# Palaeopathological and demographic data reveal conditions of keeping of the ancient baboons at Gabbanat el-Qurud (Thebes, Egypt)

**DOI:** 10.1371/journal.pone.0294934

**Published:** 2023-12-06

**Authors:** Wim Van Neer, Mircea Udrescu, Joris Peters, Bea De Cupere, Stéphane Pasquali, Stéphanie Porcier

**Affiliations:** 1 Operational Direction Earth and History of Life, Royal Belgian Institute of Natural Sciences, Brussels, Belgium; 2 Laboratory of Biodiversity and Evolutionary Genomics, University of Leuven, Leuven, Belgium; 3 ArchaeoBioCenter and Institute of Palaeoanatomy, Domestication Research, and the History of Veterinary Medicine, Ludwig Maximilian University Munich, Munich, Germany; 4 SNSB, State Collection of Palaeoanatomy Munich, Munich, Germany; 5 Labex ARCHIMEDE, UMR 5140_ASM “Archéologie des Sociétés Méditerranéennes”, Université Paul-Valéry Montpellier 3, CNRS, Montpellier, France; University of Szeged Institute of Biology: Szegedi Tudomanyegyetem Biologia Intezet, HUNGARY

## Abstract

Since predynastic times, baboons (*Papio hamadryas* and *Papio anubis*) were important in ancient Egypt for ritual and religious purposes. These species did not occur naturally in Egypt and therefore had to be imported, but little is known about their exact provenance and the conditions in which they were kept through time. Here, we analyse the skeletal remains of a collection of baboon mummies coming from Thebes (Egypt), representing a minimum of 36 individuals, from a palaeopathological and demographic point of view. The pathological cases are described, figured where relevant, and the discussion attempts to understand their aetiology. The prevalence of the different types of deformations and pathologies is compared with that of other captive baboon populations from more or less contemporary (Tuna el-Gebel and Saqqara) or older (predynastic Hierakonpolis) sites. This is combined with observations on the age and sex distribution and the proportion of hamadryas and anubis baboons to draw conclusions about the conditions of keeping, possible breeding on-site, provenance of the animals and the trade routes used for import. As in Tuna el-Gebel and Saqqara, the baboons from Gabbanat el-Qurud suffered from numerous metabolic diseases due to chronic lack of sunlight and an unbalanced diet. This and the demographic data suggest that there was a local breeding population derived from animals captured downstream from the Sudanese Nile Valley (for anubis) and from the Horn of Africa or the southern part of the Arabian Peninsula (for hamadryas). A new series of radiocarbon dates is provided, placing the baboons from Gabbanat el-Qurud between the end of the Third Intermediate Period and the beginning of the Late Period.

## Introduction

For more than a millennium, from the Saite period to the end of the Roman period (9th century BC– 4th century AD), ancient Egyptians venerated and mummified numerous animal species for religious purposes [1, 2]. So far, tens of millions of mummies have been discovered in necropolises across the country, testifying to the Egyptians’ attention to sacred or sacralised animals [3–7]. The vast majority of the mummified animals were and are mostly classified by Egyptologists as votive offerings to deities, but there was certainly wider diversity within this Egyptological category [8, 9]. Selected individuals, a minority, were considered incarnations of deities among humans, for example the Apis, Mnevis and Bouchis bulls or the Sobek crocodiles. These chosen ones, of whom there could be no more than one at a time, were mummified and buried in a more elaborate way in necropolises constructed specifically for this purpose. These sacred animals were accompanied by grave goods that resembled the inventories given to humans [3, 6, 10, 11].

**Fig 1:**
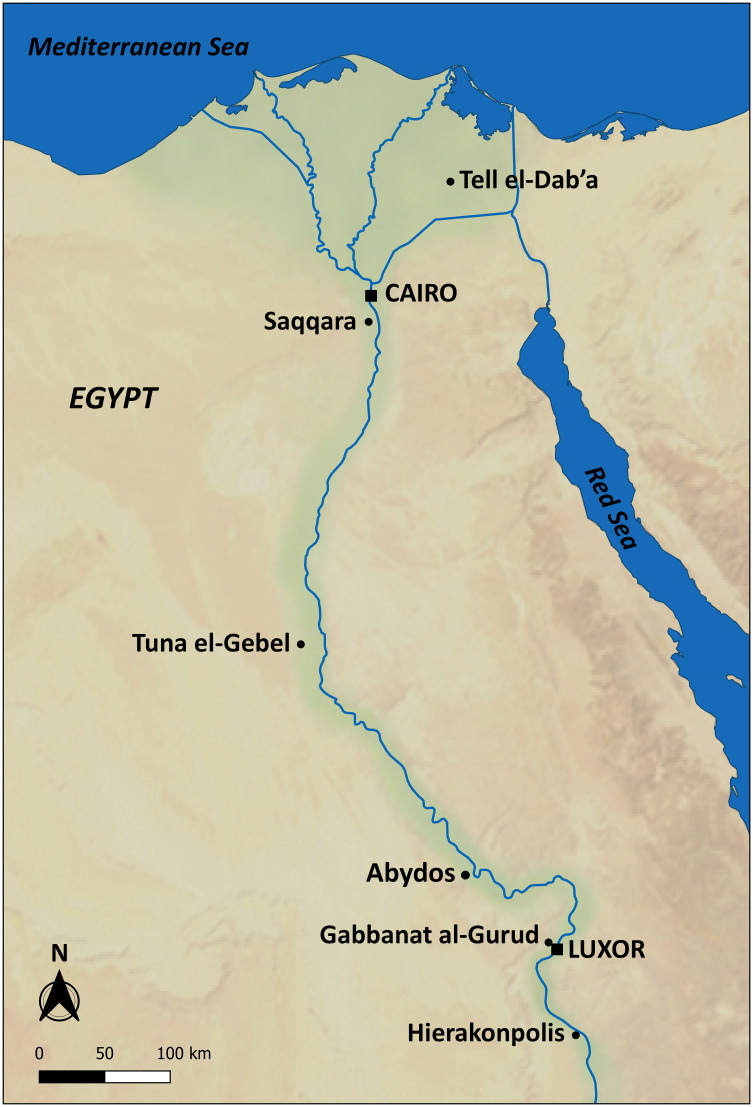
Map of Egypt showing the locations mentioned in the text.

Turning to the animal species deposited in Egyptian animal necropolises, mummies of cercopithecids (*Papio anubis*, *Papio hamadryas*, *Chlorocebus aethiops*, *Erythrocebus patas* and *Macaca sylvanus*), animals associated with the gods Thot and Khonsu and their derived forms, are very rare compared to the millions of mummies of dogs, cats, ibises and birds of prey. According to textual and archaeological evidence, monkeys were worshipped at about 12 localities [12], but the number of sites with primate mummies is limited. A ‘possible baboon’ has been reported from the Graeco-Roman period sacred falcon cemetery in the Great Wadi of North Abydos [13]. The specimen was found among five disturbed human skeletons and a stone coffin for a falcon. Larger numbers of cercopithecid mummies have been reported from three major cemeteries, namely an estimated 180 individuals at Saqqara [1–16], 247 at Tuna el-Gebel [17] and 36 at Gabbanat el-Qurud ([18, 19] and this study) (Fig 1). The bone studies, and in particular the palaeopathological observations, of the monkeys from Saqqara [14, 16] and Tuna el-Gebel [17, 20–23] provide information on the skeletal health status and living conditions of these animals.

Besides the aforementioned Late Period to Greco-Roman period cercopithecid mummies, a few finds have been attributed to the New Kingdom period on the basis of their find location. In the Valley of the Kings, near Gabbanat el-Qurud, five mummified baboons were discovered by Theodore M. Davies in three tombs without an inscription [6, 19]. These specimens, that are distributed through museums in Egypt, are better preserved than the mummies from Gabbanat el-Qurud and all belong to adult male individuals. The complete animals are still in articulating position with their skin and hair quite well preserved, and in some cases still wrapped with large quantities of linen bandages. Two additional individuals, kept in the British Museum, are said to be from Thebes, purportedly from the Temple of Khonsu [52]. From the tomb of Thutmose III, also in the Valley of the Kings, a skull of *Papio hamadryas* has been described [19].

In comparison to the elaborate studies on Tuna el-Gebel and Saqqara, the descriptions of the baboon remains from Gabbanat el-Qurud were presented a century earlier, with Lortet and Gaillard emphasising the morphological and morphometric analysis of the material. In the late 19th and early 20th centuries, well-preserved mummies ended up in art and history museums, but at the same time, numerous individuals were prepared as skeletons in natural history museums to serve as reference specimens for the study of the contemporary Egyptian fauna [24, 25]. This probably explains why the original description, which is almost 120 years old, paid relatively little attention to the skeletal deformities in the Gabbanat el-Qurud baboons. Our study reviews the results of previous work, but with a focus on palaeopathology with the aim of refining our knowledge of the conditions under which the baboons were kept. In combination with demographic parameters (age, sex, proportion of the two species), the provenance of the animals and their trade routes, age at capture and possible breeding on-site will be discussed. The observations will be compared with those from Saqqara and Tuna el-Gebel, and also with those of the much older, predynastic baboons that were held captive and buried in the elite cemetery at Hierakonpolis [26]. These earlier studies showed that traumatic lesions were abundant in Predynastic times while metabolic disorders were absent. The frequent occurrence of metabolic malformations and the rarity of trauma in Saqqara [14, 16] and Tuna el-Gebel [17] suggest that there was a diachronic evolution in the conditions of keeping. Initially, there may have been a rather violent relationship with their caretakers because of their limited dexterity in dealing with baboons. Later, the animals were kept in captivity for longer periods and under different housing conditions, resulting in a lack of sufficient sunlight and causing metabolic disorders. The age and sex distribution also suggests that baboons are now reproducing in captivity. This article will explore whether and to what extent the Gabbanat el-Qurud baboons confirm this view. Finally, a series of new radiocarbon dates will be presented that allow better chronological placement of the baboons from Gabbanat el-Qurud.

## Archaeological setting and history of discovery

The archeological site of Gabbanat el-Qurud is located in a wadi of the same name on the west bank of the Nile, about 6 km of the ancient town of Thebes (25° 43′ 46″ N, 32° 34′ 25″ E; Fig 2 in [18]). Baboon mummies from this site were first discovered by John Gardner Wilkinson in the 1830s [27] and in 1905–1906 the site was excavated by a French team from the Muséum d’Histoire Naturelle de Lyon. Several pits one to two metres deep containing mummy remains were discovered at the foot of rocky outcrops [18]. The mummies were sometimes interred in crudely carved wooden coffins, in jars or in sarcophagi made of mud bricks or terra cotta, and were accompanied by rather scarce grave goods [18]. The material was shipped to Lyon, where it was studied by Louis Lortet and Claude Gaillard, and published in a volume of the Archives du Muséum d’histoire naturelle de Lyon in 1907 and 1909, entitled ‘La faune momifiée de l’ancienne Égypte’ [18, 19]. The authors provide little contextual information on the number and the shape of pits, the number of individuals in each, the state of preservation and the grave goods of each individual.

**Fig 2:**
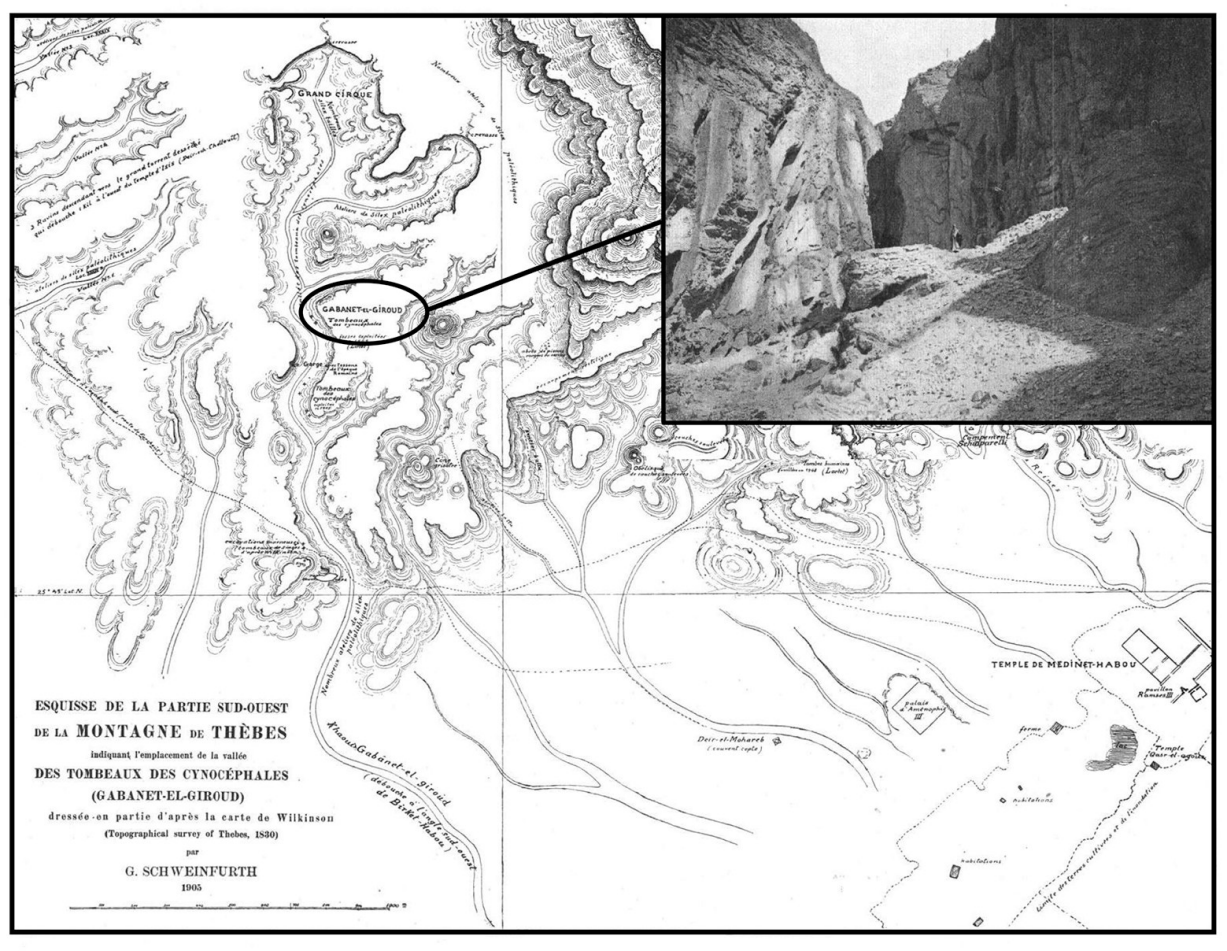
Map indicating the location of Gabbanat el-Qurud, and picture of the wadi (from [19]).

The skeletal remains collected by the French team during the first excavation campaign in 1905 in the necropolis of Thot consisted of 17 skulls and a large number of isolated bones that allowed four skeletons to be reconstructed by Lortet and Gaillard [18]. The authors mentioned that five of the skulls pertained to very young or incomplete individuals and were therefore not included in their descriptions. Of the 12 well-preserved skulls, half were classified as *Papio anubis* and the remaining half as *Papio hamadryas*. Regarding the skeletons, the authors mention one complete skeleton of a female *Papio hamadryas* that includes also the skull, while (three) other skeletons were reconstructed using skeletal elements of several individuals. In the same text passage, they mention an additional, incomplete, fifth skeleton, whose pathological bones are described separately in the same volume by Professor Poncet of the Société de médecine de Lyon.

As for the general state of preservation of the material, it is mentioned that in several tombs the bones were heavily weathered and very brittle, probably due to disturbance by torrential rains. However, the authors also report that many pieces of dry skin with tufts of long hair were discovered during the museum’s first excavation campaign in 1905 [18]. The museum’s second excavation in 1906 yielded a fairly intact mummy of a young baboon, in seated position, with its skin and hair still preserved [19]. Also found was an almost complete skeleton of a young monkey enclosed in a long jar with a very narrow neck and a wide belly. In addition, an unspecified number of more or less complete skulls were reported, as well as a large number of isolated long bones, all from primates.

## Materials

Today, the mummy remains are kept at the Musée des Confluences, the former Musée d’Histoire naturelle de Lyon. In our descriptions below, we use the registration numbers assigned to the specimens since 2005, when the material was registered in the museum’s database. Where possible, we mention the corresponding original numbers used by Lortet and Gaillard in their monograph. In the museum collections, eight clusters of bones are stored as skeletons, three of which clearly correspond to the descriptions given by Lortet and Gaillard. The five others are not mentioned in the original monograph and in three cases are composed of bones from more than one individual. In the early 1900s, attempts were made to reconstruct as many complete skeletons as possible, even using bones that did not belong to the same individual. It is worth mentioning that metal wires were used to assemble such articulated skeletons, which in some cases perforated the articular ends of the bones, leaving holes that should not be mistaken for pathologies. There are three skeletons without a skull. Five skeletons had an associated skull, but only one skull association was convincing. In two cases the association was uncertain, and in two it was clearly false. The skeleton of the intact mummy from the 1906 excavation season (MHNL 90001206) could not be analysed because the bones are still embedded in soft tissue. Moreover, we could not find the second mentioned skeleton from that same excavation season in the collections. This means that most of the bone material analysed below consists of remains recovered during the 1905 excavation. In addition to the available skeletons mentioned above, the collections produced 23 skulls (one corresponding to a skeleton), 24 mandibles (one corresponding to the same skeleton) and more than 200 isolated long bones and vertebrae, several of which show pathologies that can be diagnosed.

The baboons from Gabbanat el-Qurud have traditionally been dated to the Ptolemaic and/or Roman period based on archaeological objects found with the mummies. These include two small terracotta lamps identified by Louis Lortet and Claude Gaillard as of Greek manufacture (Fig 112 in [18]; [28]). A relatively late date has been suggested by a ceramological study of bowls (Fig 107 in [18]; MHNL 90000640), dated at the earliest to the 1st-2nd centuries AD and at the latest to the 5th-7th centuries AD ([28]; Pl. 40.6 in [29]; 104 # 1089–1116 in [30]; # T 323 in [31]); and by the presence of two amphora from the Byzantine period ([28]; Fig 109 in [18]; Pl. 29.2 in [30]; # K 737 in [31]; MHNL 90000557). A much earlier date was obtained by radiocarbon dating of textiles used to wrap a baboon (MHNL 90001206), which is recorded in the museum’s database as being of unknown provenance but must have come from the same locality: SacA40592: 2545 ± 35 BP (calibrated age 2σ: 803–544 cal. BC) [2].

To better understand and clarify the exact dating of the baboon mummies, we performed additional radiocarbon datings, this time on bone collagen. A previous attempt to date cranial bone of one the baboon mummies (MHNL 90000176) was unsuccessful (Richardin [Unpublished]) because of the poor preservation and quality of the collagen. For this reason, and in avoidance of destructive sampling of the skulls, we took bone samples from the tibiae of 13 different individuals (register number MHNL 51000326).

## Methods

Several criteria have been described to distinguish between the skulls and mandibles of the two baboon species *Papio anubis* and *Papio hamadryas* [17, 20]. We verified all these characteristics on a large series of modern skulls of both species and found that the only valid criteria are (i) the angle between the vertical plane across the orbitae and the horizontal plane along the palatal bone and (ii) the relative length of the snout. The deep external mandibular groove, described as being diagnostic for *Papio anubis*, can also be found in *P*. *hamadryas*. Identification at species level of the baboons from Gabbanat el-Qurud has therefore been limited to well-preserved skulls. Sex could be determined by the size of the upper and lower canines, which are much heavier in males, and by the third lower premolar, which is much longer in males than in females. A clear sexual dimorphism is also observed on the cranial roof: the temporal lines run parallel and are more lateral in females. In males, these lines converge to a sagittal crest in adulthood [17]. Age determination of skulls and mandibles was possible using data on tooth eruption and dental wear published for captive baboons [32, 33]. Age estimates of long bones were based on epiphyseal fusion data published by Bramblett [34]. For measuring the long bones, the protocols described by von den Driesch [35] were followed and for the skulls and mandibles, the measuring distances were used as defined in von den Driesch et al. [17]. For the skull, we added a single measurement, namely the interorbital width (width between both orbits).

The starting point of our evaluation of the pathologies observed in the ancient Egyptian baboons are modern specimens representing two groups with different living conditions, namely wild-caught *Papio anubis* and captive-reared *P*. *hamadryas* (S1 Table). The reference collection lacked skulls of wild-caught hamadryas as well as captive anubis baboons, but since both species are closely related and even interbreed in contact areas [36], this is probably a shortcoming of theoretical nature that will not affect our interpretations. The expression of the specimens served as a benchmark to evaluate the effects of ancient anthropogenic living conditions.

## Results

### New radiocarbon dates of the baboons

Only three out of 13 bone samples from the tibiae of different individuals yielded collagen of sufficient quality for dating (Table 1). The radiocarbon dates obtained range between 794 and 520 BC (95.4% probability) and are in agreement with that previously obtained on the baboon textile wrapping (MHNL 90001206), namely 803–544 cal. BC. It thus appears that the baboon mummies should be dated to the end of the Third Intermediate Period–beginning of the Late Period (23rd-26th Dynasty), which is earlier than previously assumed based on the ceramic inventories. The temporal difference between the radiocarbon dates and the age range suggested by the artefacts shows that the latter were not associated with the baboons and correspond to later phases of use of the site. Currently, there is also no known contemporaneous textual source relating to baboons in Thebes. However, between the 4th and the 2nd c. BC, members of the staff of the ‘living baboons’ of the god Khonsu are attested, to which one can probably associate the mummified monkeys of Gabbanat el-Qurud [12, 37].

**Table 1:** Calibration of the radiocarbon dates using Oxcal v4.4.4 (© Bronk Ramsey 2021), r.5, taking into account the atmospheric data by Reimer et al. [38].

Lab. code	specimen	BP	s. dev.	%C	%N	δ^13^C	δ^15^N	at C:N	68.3% probability		95.4% probability	
RICH-25387	tibia 02	2498	29	26.21	10.61	-20.0	12.7	2.9	762-744BC 690-665BC 646-550BC	8.7% 12.4% 47.1%	776-538BC 528-520BC	94.6% 0.8%
RICH-25388	tibia 04	2534	29	37.41	13.42	-19.4	11.7	3.3	786-750BC 684-667BC 635-589BC 578-574BC	27.6% 12.4% 26.7% 1.6%	794-736BC 695-662BC 649-546BC	33.0% 16.4% 46.1%
RICH-25389	tibia 12	2534	29	38.55	13.57	-18.6	13.3	3.3	786-750BC 684-667BC 635-589BC 578-574BC	27.6% 12.4% 26.7% 1.6%	794-736BC 695-662BC 649-546BC	33.0% 16.4% 46.1%

### Number of individuals, species, age and sex

After studying all the skeletal remains of baboons at the Musée des Confluences, we conclude that they belong to at least 36 individuals (minimum number of individuals, or MNI), a figure obtained when considering the available skulls and mandibles. As such, there are 23 skulls, 11 of which with their mandibles associated, and 13 isolated mandibles that do not fit any of the skulls. None of the postcranial elements produced an MNI close to the figure obtained with the cranial remains. For the sacrum, the MNI is 24. Among the long bones, the highest MNI (21) is obtained when the tibiae are considered, both the isolated specimens and those assumed to belong to a particular skeleton. In the supplementary information, all the measurements are provided that could be taken on the complete skeletons (S2 Table), on the skulls and associated mandibles (S3 Table) and on the isolated mandibles (S4 Table) and long bones (S5 Table). For the sake of completeness, we provide in those four files also the metrical data of the baboon skeletal remains with no signs of pathologies.

Below we discuss the proportion of the two baboon species at Gabbanat el-Qurud, the sex ratio and the age at death of the animals. Table 2 summarises the information obtained on the cranial remains with regard to species and sex identification and age at death of the individuals. We express age at death in dental stages following the classification of von den Driesch et al. [17], because this allows us to compare the data of Gabbanat el-Qurud with those of Tuna el-Gebel. In Table 3, dental stages are aggregated into age classes, again according to von den Driesch et al. [17].

**Table 2:** Summary of the dental stages obtained on the skulls and mandibles from Gabbanat el-Qurud, indicating taxon and sex.

				adult		subadult		adolescent			juvenile		infant	
		dental stage	?	10	9	8	7	6	5	4	3	2	1	total
*P*. *anubis*	male			2										2
*P*. *anubis*	female			2	2	1	1							6
*P*. *hamadryas*	male			2							1			3
*P*. *hamadryas*	female			2	1									3
*Papio* sp.	male		4	2	1			1						8
*Papio* sp.	female			1										1
*Papio* sp.	male?				3									3
*Papio* sp.	?			1			1	1	1		5		1	10
			4	12	7	1	2	2	1	0	6	0	1	36

The stages are those defined by von den Driesch et al. [17].

**Table 3:** Distribution of age at death of the baboons from Gabbanat el-Qurud.

		adult	subadult	adolescent	juvenile	infant	total
*P*. *anubis*	male	2					2
*P*. *anubis*	female	4	2				6
*P*. *hamadryas*	male	2			1		3
*P*. *hamadryas*	female	3					3
*Papio* sp.	male	6		2			8
*Papio* sp.	female	1					1
*Papio* sp.	male?	3					3
*Papio* sp.	?	1	1	2	5	1	10
		22	3	4	6	1	36

Based on the data of Table 2. The 4 male *Papio* sp. individuals, whose dental stage could not be determined (indicated as “?” in Table 2) because only the skull roof was preserved, are taken into account as well. They belong to 3 adults and 1 adolescent judging from the bone density and fusion state of the neurocranial bones.

Our study of the cranial remains thus shows that *Papio anubis* (MNI 8) is more numerous than *P*. *hamadryas* (MNI 6). Our results also indicate the presence of all age classes, but with a preponderance of adult individuals, several of which received attention in the first description by Lortet and Gaillard [18]. The sex ratio shows a slight preponderance of males: among the adolescent to adult animals there are 10 females and 12, possibly 15, male individuals.

The postcranial remains are not suitable for age distribution analysis, given the absence of unfused elements among the isolated long bones housed at the Musée des Confluences. Apparently, adult postcranial bones were preferentially selected for the osteological reference collections. This would help explaining the lower MNI value obtained from the postcranial elements.

### Diagnosis of the pathologies observed on the skeletons

A detailed description of the pathologies seen in the baboon remains from the Lyon collections is presented in the supporting information (S1 Text). First, observations we made on the skulls and mandibles are provided, then the more or less complete skeletons are described, and finally the isolated skeletal elements that showed pathological conditions but could not be linked to a particular individual. Only descriptions are given in the supporting information; the likely diagnoses of the observed pathologies are discussed below.

Besides four skeletons that appear to have been correctly reconstructed, two more skeletons are composed of bones from at least two individuals. One skeleton is even composed of elements from three different individuals (see also S2 Table). In addition, there are a number of specimens with pathological conditions encountered amongst the loose elements stored in the collection. It is considered useful to include all this material in the discussion, as it increases the number of observations on pathologies in the Gabbanat el-Qurud baboons. It is also worth mentioning that there is only a single skeleton with a correctly assigned skull and that in two cases the association was uncertain. For all other skeletons, there was either no associated skull at all, or the skull assigned to a particular skeleton actually belonged to another individual.

#### Healthy individuals

Of particular interest is the presence of four apparently healthy individuals lacking clear traces of metabolic disease or trauma. This applies to the female hamadryas baboon (MHNL 51000172), corresponding to skull #1 and skeleton #1 of Lortet and Gaillard. The only conditions observed are caries in a lower third molar and linear enamel hypoplasia lines in the right upper and lower first incisors. The skeleton, moreover, is characterised by heavy muscle attachments on the shafts of the humeri and, to a lesser extent, on the tibiae. However, these can be considered normal variation within healthy wild specimens. A similar condition can be seen on the tibiae, radii and ulnae of the male *Papio* sp. (MHNL 51000325) that otherwise looks completely healthy. The skeleton labelled MHNL 51000170—which in fact consists of material from two individuals—shows no pathologies at all, except for a slight exostosis on the trochanter major of a right femur.

#### Rickets and osteomalacia

As pointed out in earlier studies dealing with mummified baboon remains, metabolic bone diseases were a major health issue in captive populations. Traditionally described as distinct morphologic entities with characteristic pathogenesis and lesions, it is now increasingly common practice to subsume rickets, osteomalacia, fibrous osteodystrophy and osteoporosis under the term osteodystrophies or metabolic bone diseases. Sometimes we noted osteological markers typical of a certain disorder, allowing us to propose a diagnosis. Nevertheless, any such decision must be considered provisional because in the case of metabolic bone diseases, individuals can suffer from more than one of the aforementioned conditions, which will then create confusion diagnostically. Interpretation of osteodystrophies is further complicated by the fact that their manifestation may differ between species and that each condition, be it rickets, osteomalacia, fibrous osteodystrophy or osteoporosis, can result from more than one dietary or endocrine imbalance, a point to which we will return in the discussion.

Decreased growth rate, angular limb deformities and collapse of the subchondral bone of the humeral head are considered the typical lesions of rickets. Whereas rickets results from dietary imbalance in juvenile and adolescent individuals, osteomalacia is the continuation of the disorder in adulthood. Typical osteomalacic bones have reduced resistance to pressure and tension, and increased susceptibility to the stress of routine activities. As a result, excessive deposition of matrix occurs where mechanical stimuli are strongest, such as at insertions of tendons and fascia, places of angulation and curvature, and on stress-oriented epiphyseal trabeculae [39].

**Fig 3:**
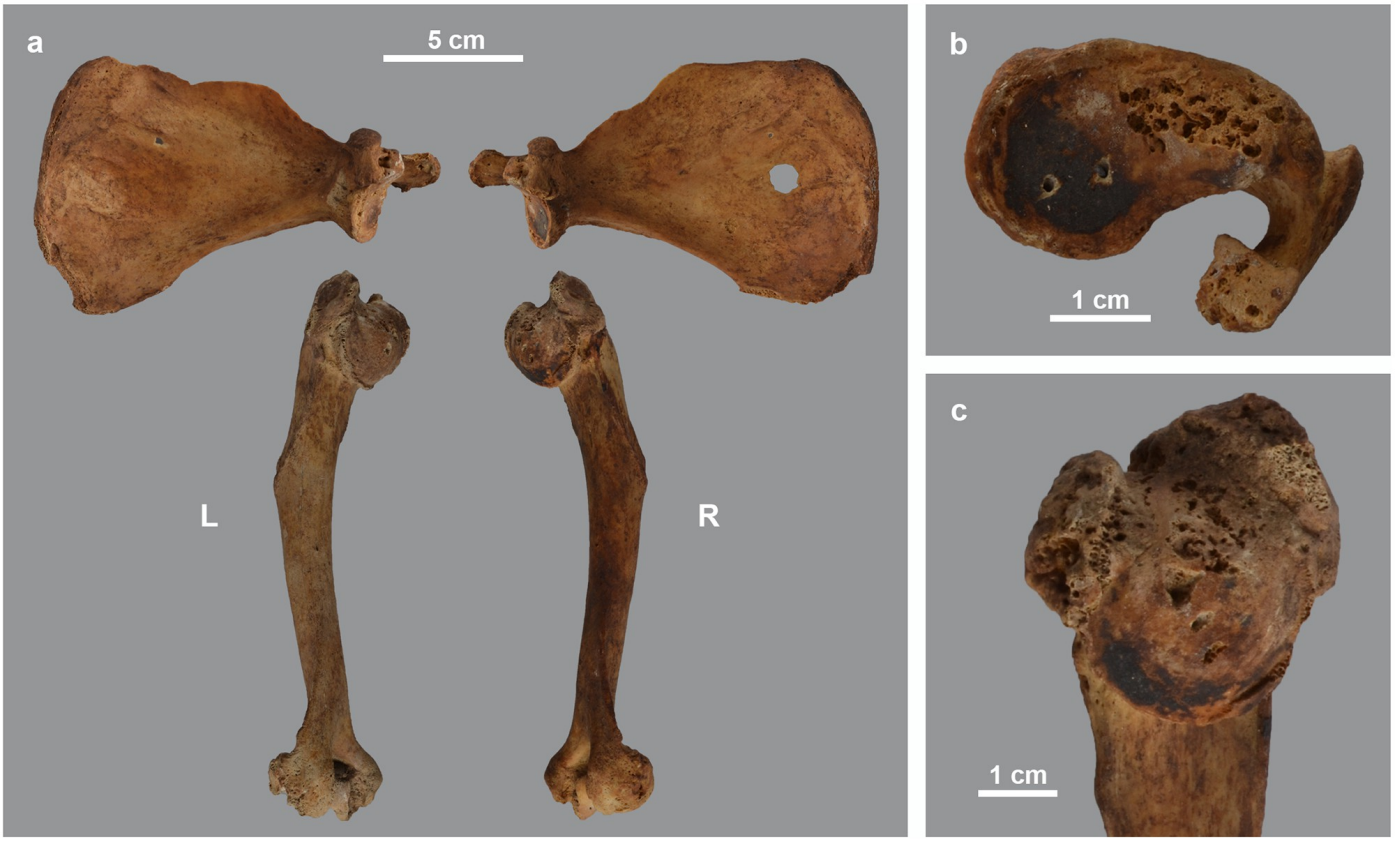
Deformations in the arm skeleton of male *Papio* sp. MHNL 51000173. a) left and right scapula and humerus showing bending of the humeri and downward tilting of humeral heads; b) right scapula showing pitting of glenoid cavity and base of acromion; c) right proximal humerus showing downward tilted articular head and circular bony margin formed by osteophytes.

**Fig 4:**
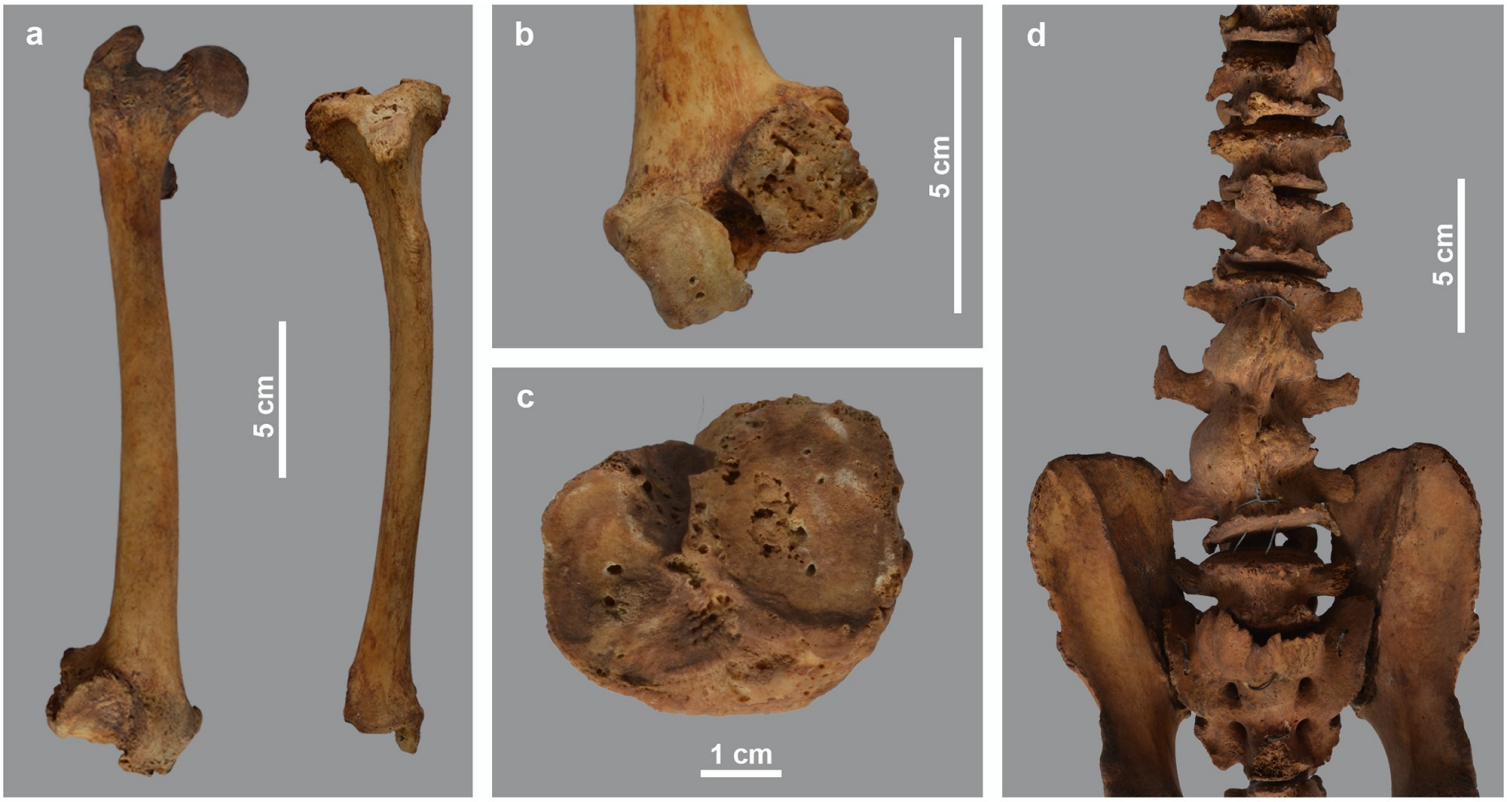
Deformations in the hind leg and vertebral column of male *Papio* sp. MHNL 51000173. a) right femur and tibia, showing the deviation of the distal epiphyses; b) distal part of right femur in distal view, showing the irregular surface of the lateral epicondyles resulting from osteophyte formation; c) left tibia in proximal view, showing osteophyte formation and lipping, as well as the pitted surface of the articulations. The circular holes are artefacts due to metal wires; d) ventral view of sacrum, lumbar vertebrae and three last thoracal vertebrae, showing ankylosing centra.

As shown in the detailed description of the pathologies (S1 Text), three individuals show this kind of bone damage. A first case is skeleton MHNL 51000173, a male baboon (*Papio* sp.). Several deformities indicate a dysfunctional scapulo-humeral joint, causing reduced mobility of the arm, with its slight curvature of the shaft and collapsed articular head (Fig 3). The same diagnosis also explains the deformities of the hind limbs (Fig 4). We identify erosive and reactive new bone formation on the entire articular surface of the distal femur and the proximal tibia. We also find a large disruption of the cortical edges of the proximal surface of the tibia. The pathologies described for the distal femur and proximal tibia indicate non-axial loading of the hind extremity due to malalignment of the consecutive limb sections. Overall mobility was hampered by arthrosis of the knee joint. Finally, this individual also suffered from ankylosing spondylosis, the aetiology of which is complex and not fully understood [40], though vitamin D deficiency has been suggested as a causative factor [41].

**Fig 5:**
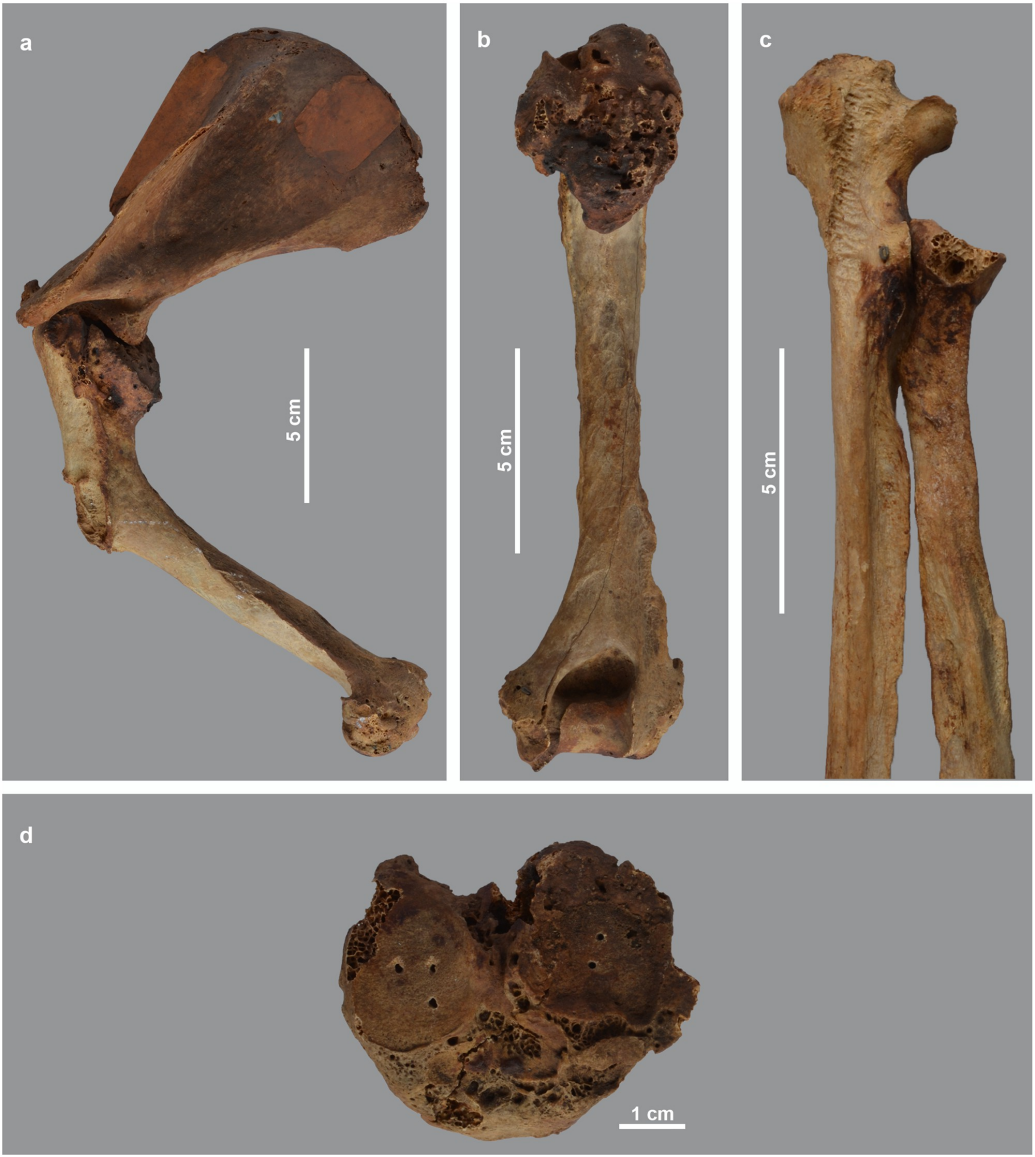
Deformations in the arm and hind leg of male *Papio* sp. MHNL 51000171. a) medial view of left scapula and humerus, showing the bowed aspect of the humeral shaft and the strong muscular insertions; b) caudal view of right humerus, showing the reticular cortical bone surface and the collapsed, downward directed head with osteophytes at the level of the articulation and around the margins. c) medial view of the right proximal ulna and radius, showing the reticulated ulnar head; d) proximal articulation of the right tibia with periarticular osteophytes. The circular holes in the two articular facets are artefacts.

**Fig 6:**
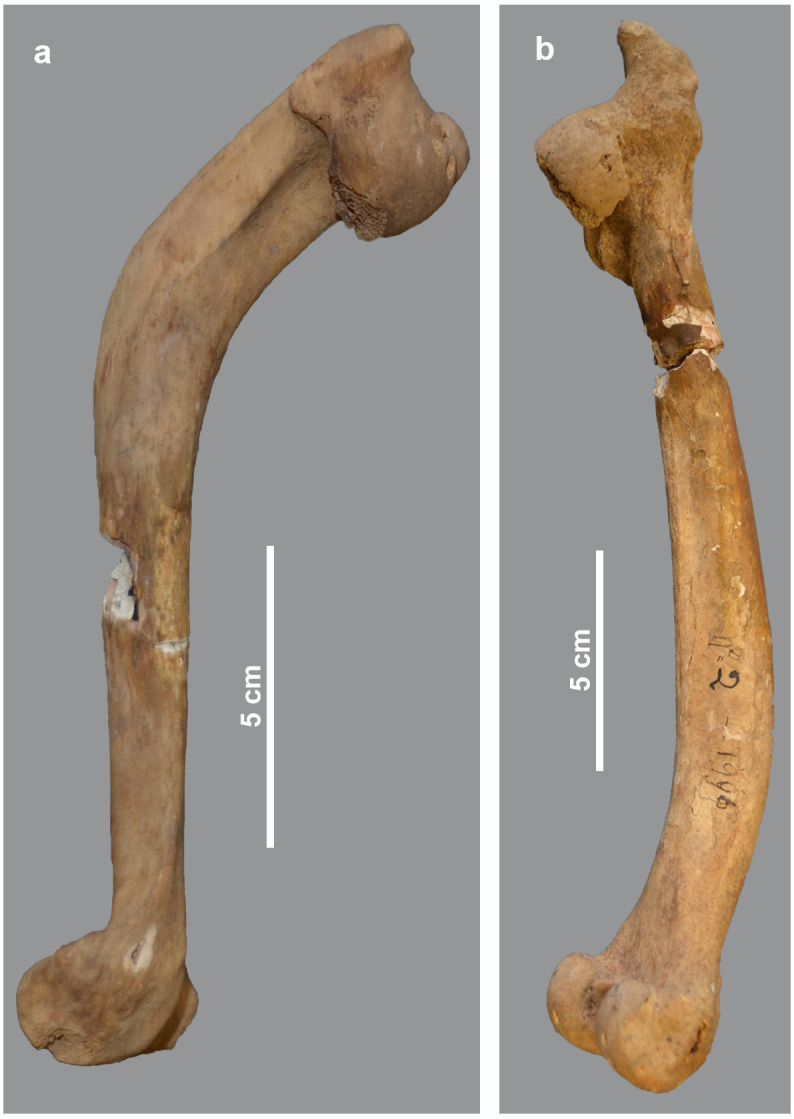
Left humerus (a) and femur (b) of *Papio* sp. female MHNL 51000328B. Shown are the bent shafts and the collapsed and downward deviated proximal articular heads.

**Fig 7:**
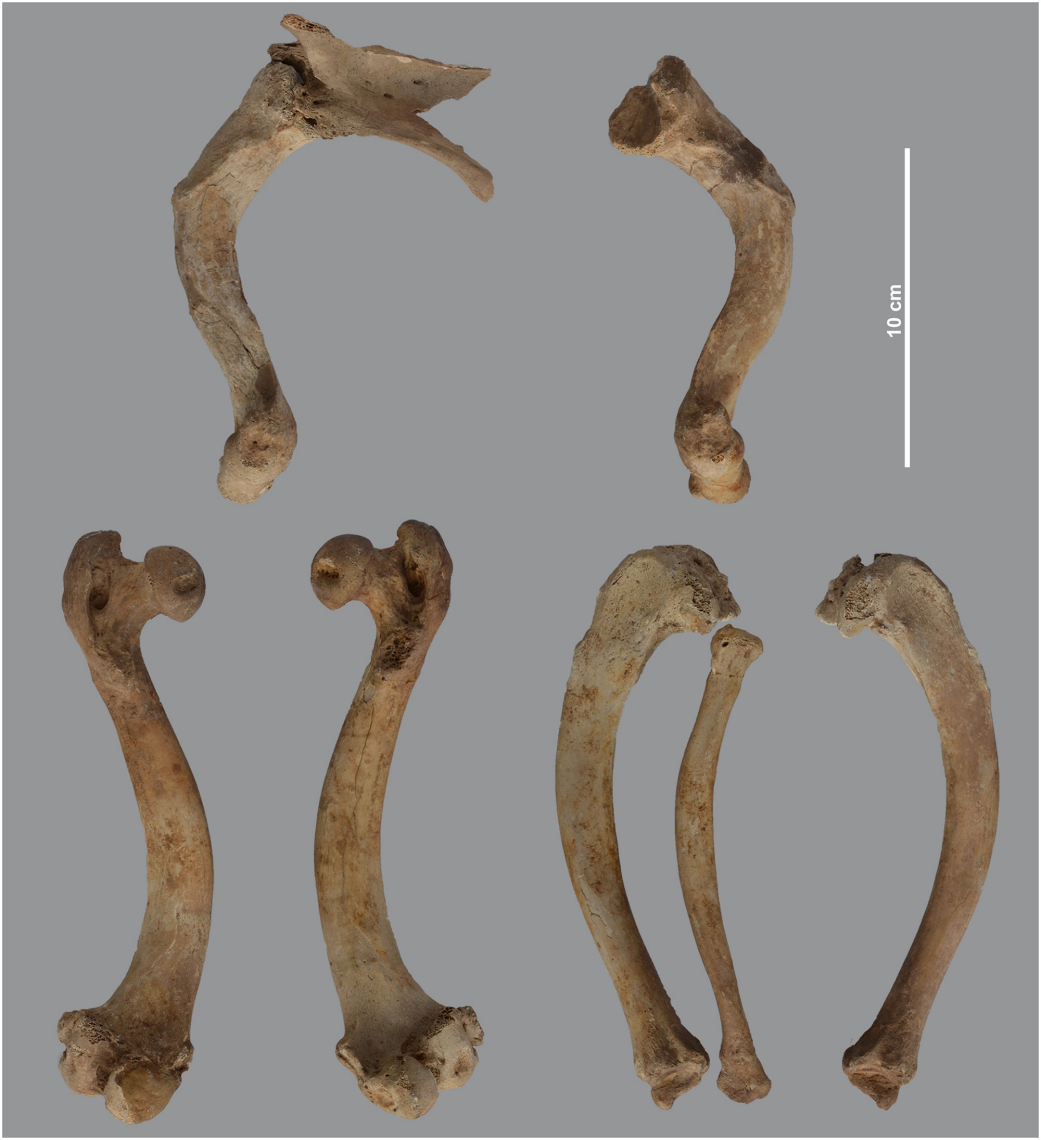
Overview of the long bones of *Papio* sp. MHNL 51000324 showing signs of rickets. Left scapula and left and right humerus (top). Left and right femur (bottom left) and left tibia and right tibia and fibula (bottom right).

**Fig 8:**
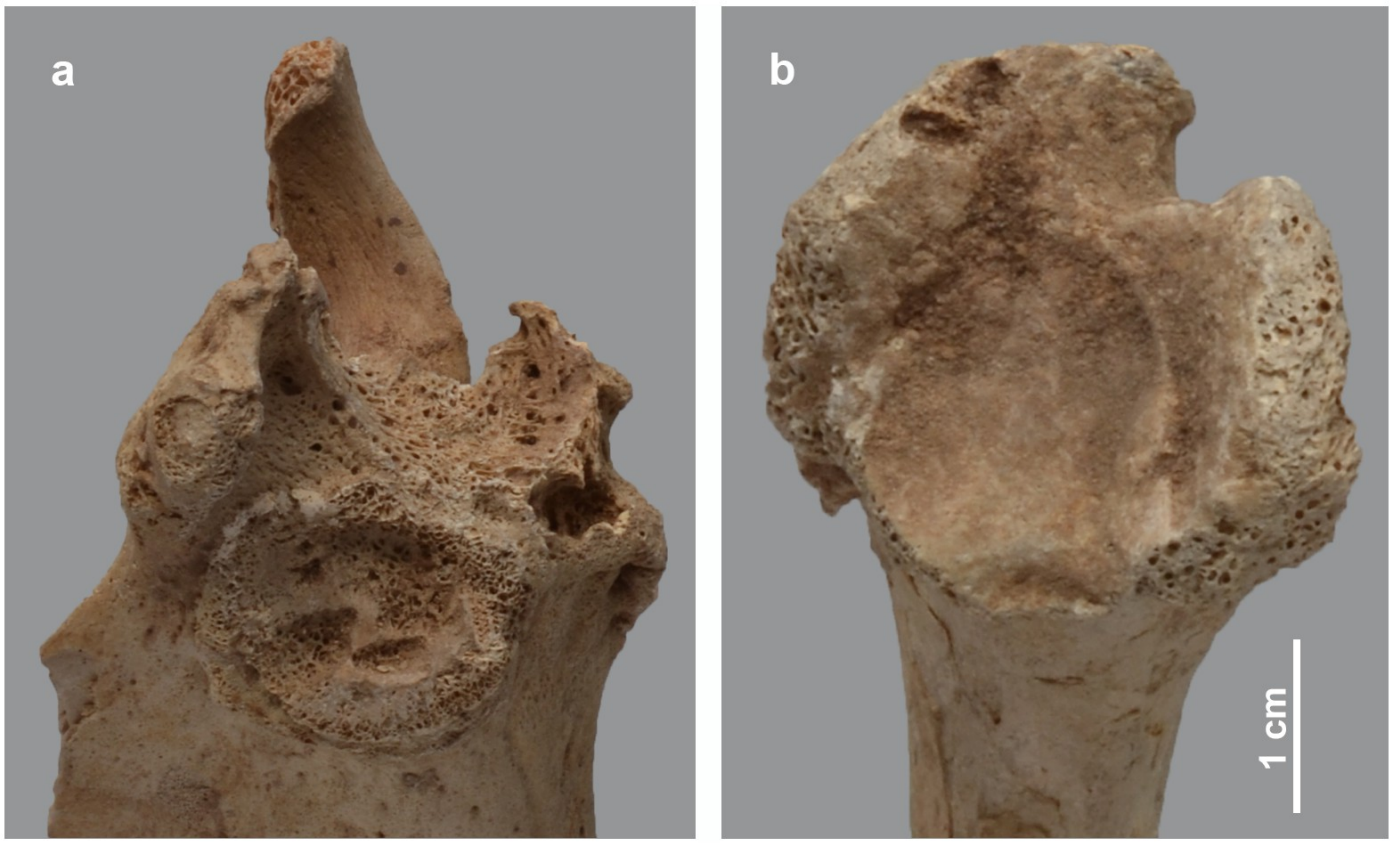
View on left scapulo-humeral joint elements of *Papio* sp. MHNL 51000324. a) scapula showing three articular facets with rugose and irregular appearance; b) proximal humerus with the articulation composed of three facet surfaces.

**Fig 9:**
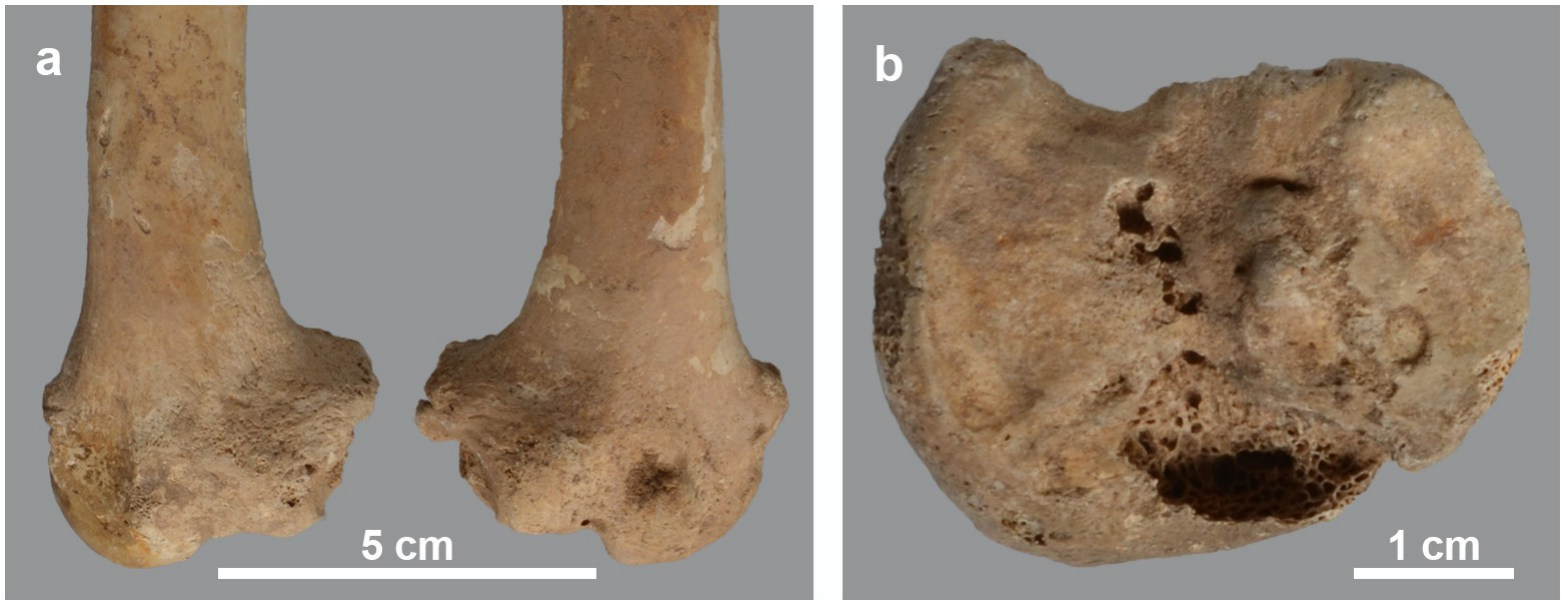
Details of hind limbs of *Papio* sp. MHNL 51000324. a) cranial view of the femurs showing the deviating distal ends and poorly indicated trochlea patellaris; b) proximal articulation of the right tibia showing erosive lesions, new bone formation around the articular surface and a pitted tibial plateau.

The post-crania of skeleton MHNL 51000171, again a male *Papio* sp., show several pathologies, including a near ankylosis of the scapulo-humeral joint, and of the coracoid process of the scapula with the coracoid (Fig 5). This means that arm movements were severely restricted in this individual. Both the collapsed joint head of the humerus and the curved shaft of the bone make the diagnosis of rickets plausible. The slight bending of the radius and the heavy muscle attachments and reticular appearance of the proximal ulna correlate with the humero-scapular joint condition mentioned above. The hind limbs are only represented by a right tibia and fibula. Although these elements show no obvious bending, the articular surface of the tibia attests to severe degeneration of the knee joint, consistent with the diagnosis proposed for the forelimbs. However, in this individual, the development of heavy muscle attachments and poor mineralisation taking place during skeletal remodelling in the radius-ulna may also indicate health problems that persisted from adolescence into adulthood and consequently a diagnosis as osteomalacia. A broadly similar situation to skeleton MHNL 51000171 can be observed in the less complete skeleton MHNL 51000328B, which belongs to a female baboon (Fig 6). The strong bending of the humeri and femora combined with the collapse and downward inclination of the respective proximal articular heads due to the severely damaged subchondral metaphysis again point to a metabolic problem in adolescence, most likely rickets. The unsexed partial skeleton MHNL 51000324, representing an unidentified species of baboon, consists of eight heavily deformed post-crania (Fig 7). Humeri, femora, tibiae and a single fibula are all heavily bent. Most notably, due to the damage to and dislocation of the humeral heads, neoarthrosis replaced the original scapulo-humeral joints on both the left and right to ensure function. This new, symmetrical articulation consisted of three separate, small surfaces in both the scapula and humerus (Fig 8). The left and right hind limbs show severe damage and joint wear as well. Also notable are the short femoral neck, the poorly pronounced, shallow patellar ridge and the clear broadening of the proximal tibia plateau (Fig 9). Taken together, these observations point to metabolic issues early in this individual’s life and rickets as the probable diagnosis.

**Fig 10:**
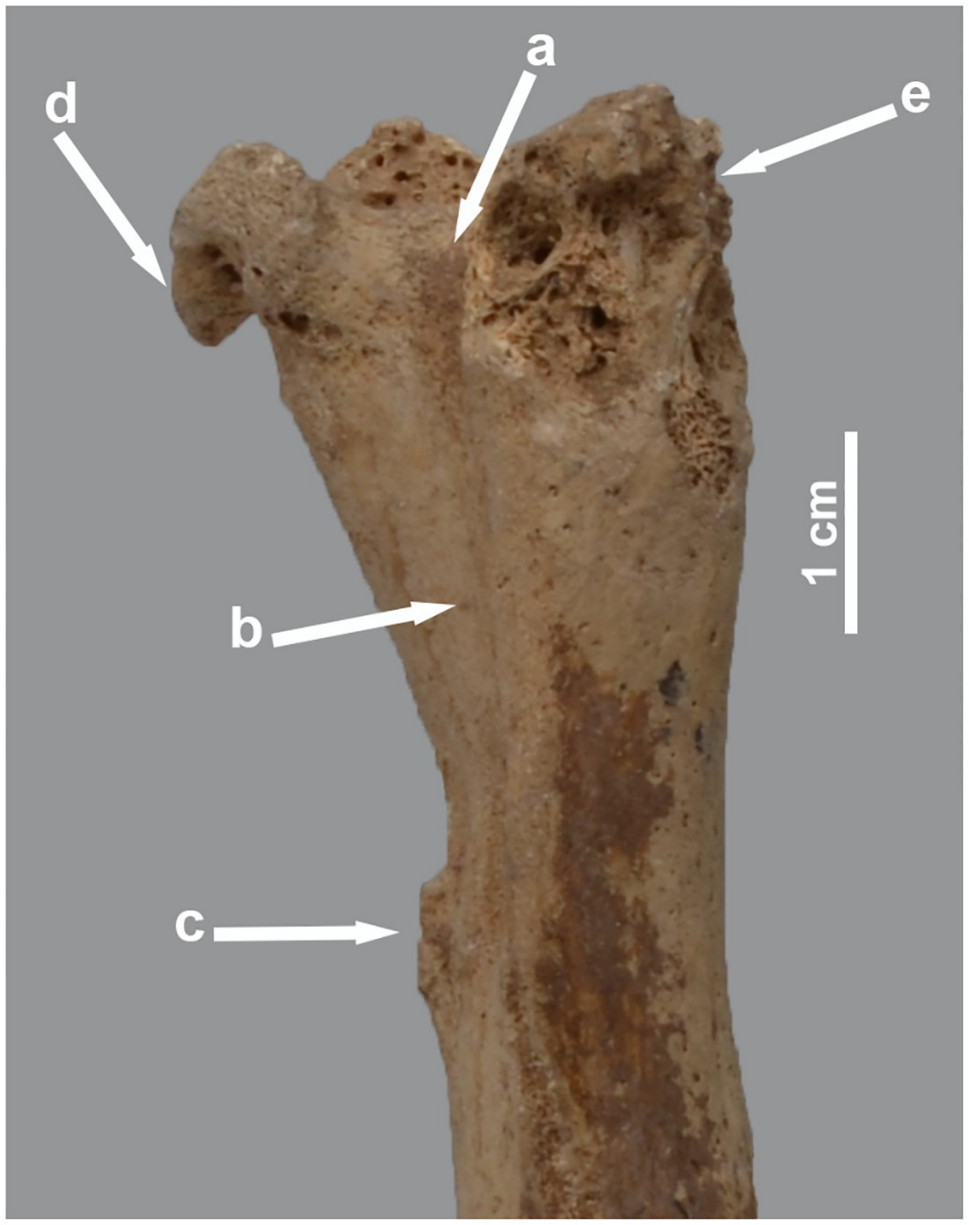
Cranial view of the proximal part of the left humerus of *Papio* sp. MHNL 51000324. Shown are the sulcus intertubercularis (a) and the bicipital groove (b), an ossified tendon (c), as well as the remaining parts of the tuberculum minus (d) and majus (e).

Parallels to the above have been observed in some of the isolated long bones as well. We therefore propose a diagnosis of rickets for three humeri (one left and right of the same individual and an additional right specimen), all with collapsed articular heads (MHNL 51000332), and two right radii with curved shafts (MHNL 51000324, MHNL 51000331). The two humeri in skeleton MHNL 51000324 are reminiscent of an additional left humerus with the same number, characterised by the absence of its articular head with exposure of the underlying spongy bone and damage to the tuberosities (Fig 10). Two lumbar vertebrae with ankylosing spondylosis belonging to the same skeleton (MHNL 51000324) presumably fit this aetiology too.

#### Neurocranial lesions

**Fig 11:**
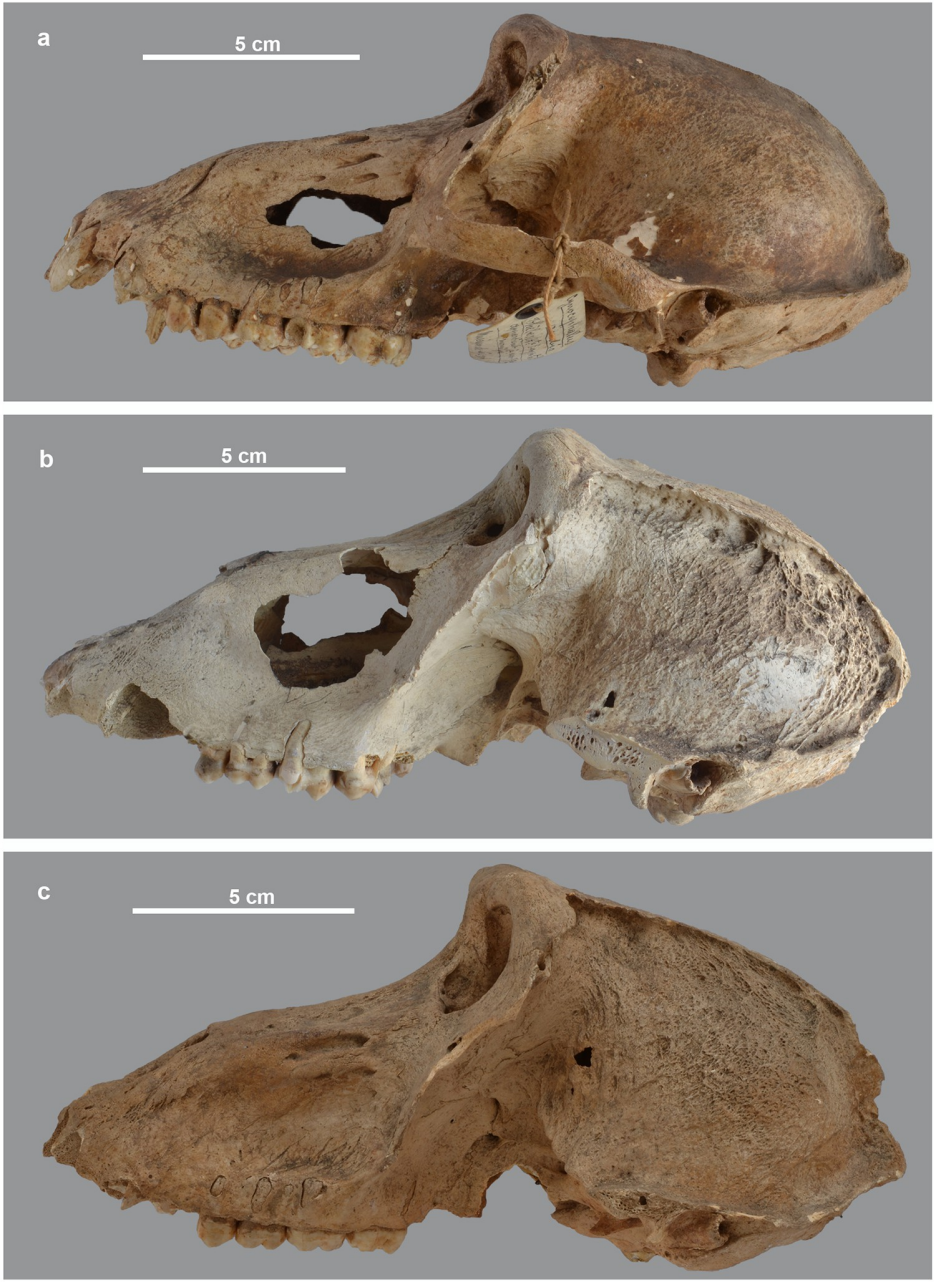
Left lateral view of the skull of three male baboons. *P*. *hamadryas* MHNL 51000171 (a), *P*. *anubis* MHNL 51000173 (b) and *P*. *hamadryas* MHNL 51000174 (c), showing the rugose or reticulated appearance of the frontal and parietal bones.

**Fig 12:**
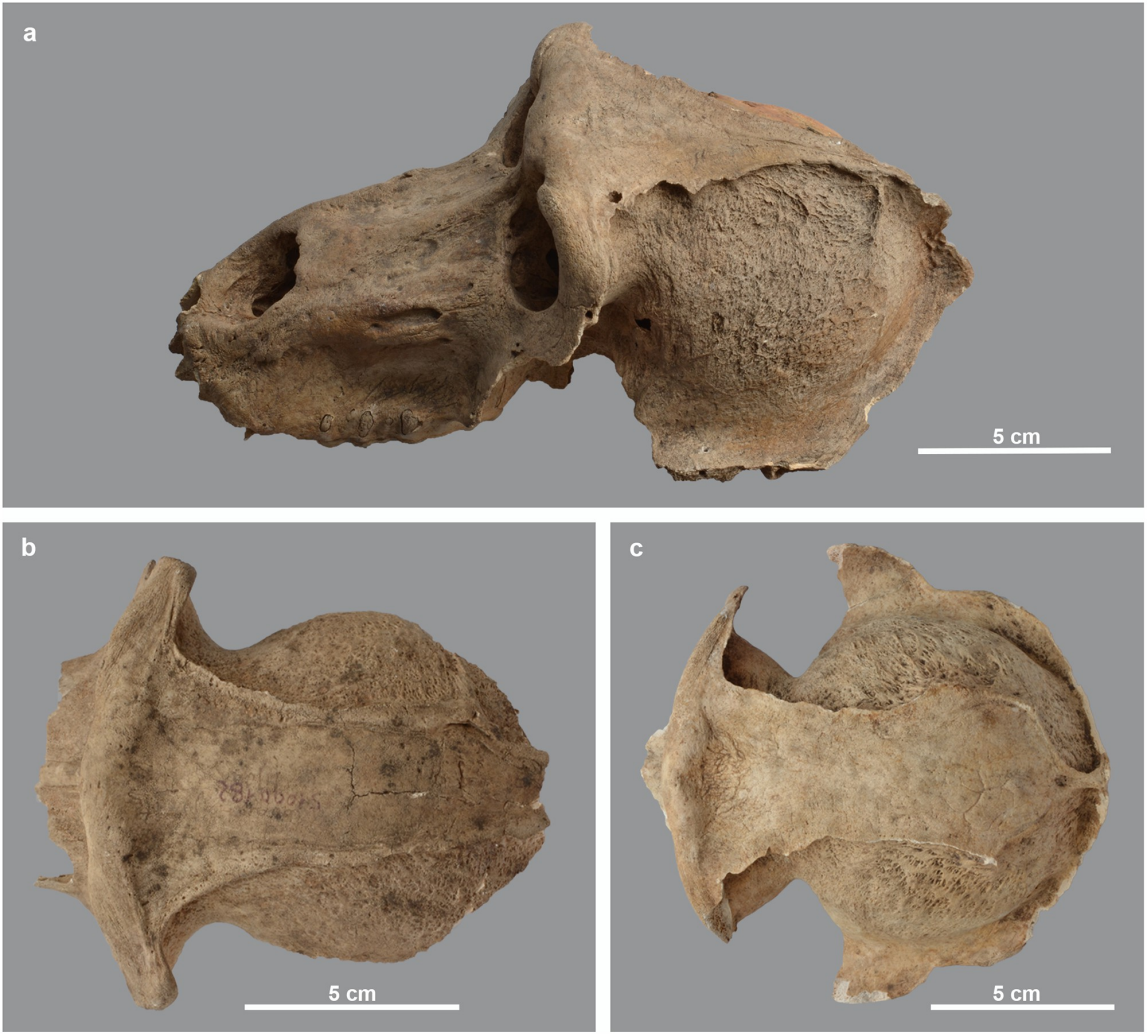
Rugose or reticulated appearance of the hind skull and laterally extended lineae temporales in three male baboons. Left superolateral view of *P*. *hamadryas* male MHNL 51000174 (a), dorsal view of male *P*. *anubis* MHNL 51000182 (b) and male *Papio* sp. MHNL 51000319 (c).

Several osteopathies observed in baboon skulls seem of metabolic origin as well, though the modifications observed vary in extent and intensity. Thus, various degrees of changes in the external aspect of the neurocranium have been observed, mostly expressed in the form of a rugose or reticulated appearance of the frontal, parietal and sometimes occipital bones. Lateral bony extensions of the lineae temporales also occur in these specimens, and the surface delimited by these structures is of variable width and generally smooth (Figs 11 and 12). The specimens concerned include one female anubis baboon (MHNL 90002001A), two male anubis baboons (MHNL 51000173 and MHNL 51000182), two male hamadryas (MHNL 51000171 and MHNL 51000174) and two unidentified males (MHNL 51000319 and MHNL 51000323A). The cranial rugosities are also mentioned by Lortet and Gaillard [18], who follow Poncet’s interpretation (p. 28 in [18]), that they do not represent a pathological condition, but are rather the result of post-mortem destruction of the skull roof causing removal of the lamina externa (= the outer compact layer of bone) and subsequent exposure of the diploe. However, rather than a taphonomic explanation, a metabolic aetiology of variable intensity seems a more likely explanation, as different stages can be observed that reflect increased modification of the bone surface.

**Fig 13:**
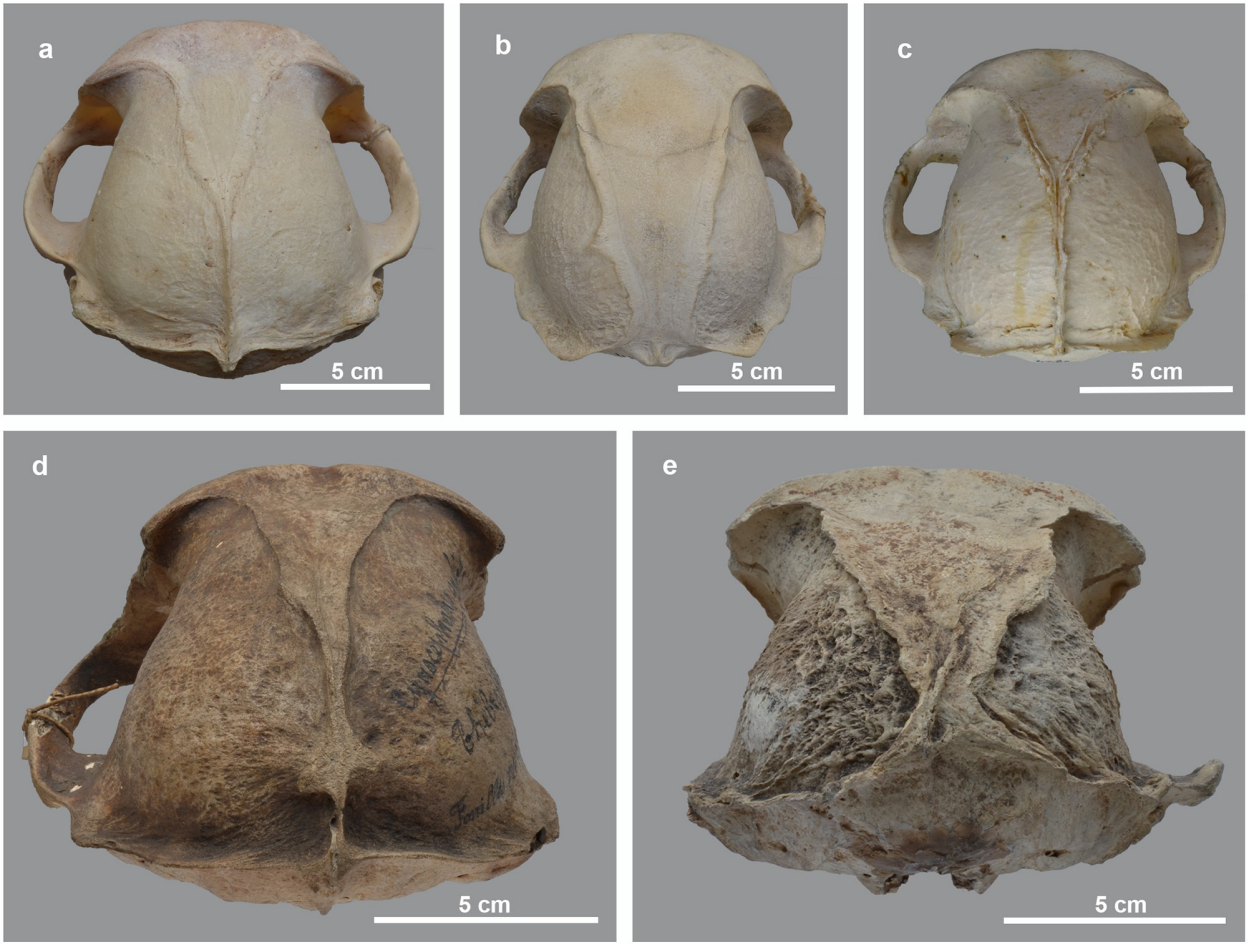
Posterior view of the skull of modern and ancient baboons. a) a modern wild-caught male *P*. *anubis*; b) modern captive male *P*. *hamadryas* and c) modern captive male *P*. *hamadryas*; compared to d) male *P*. *hamadryas* MHNL 51000171 and e) male *P*. *anubis* MHNL 51000173.

In absence of plausible parallels in the human and animal palaeopathology literature dealing with non-traumatic skull roof osteopathies, we decided to examine a large number of modern baboon skulls in the Royal Belgian Institute of Natural Sciences (RBINS) to better understand the nature and aetiology of these malformations. The large modern collection evaluated included both baboons caught in the wild and animals kept in zoos. From more than 100 specimens of *P*. *anubis* and *P*. *hamadryas*, we selected a series of skulls that provide clues about captivity as a cause of such deformities (S1 Table). We found very few specimens with a rugose or reticulated external skull roof in the wild specimens and, if present, it was always very faint. In our captive sample, however, the surface was more affected, to varying degrees, though never to the same extent as in the ancient Egyptian baboons. Fig 13 shows a series of modern and ancient skulls illustrating the continuum from wild, little-impacted specimens through slightly to moderately altered zoo specimens to heavily impacted specimens from the site. Apparently, captivity exacerbates a condition rarely seen in wild animals. Despite measures to improve housing and nutrition [23], living conditions in modern zoos at the end of the 20th century seem to have caused the aforementioned skull lesions. That said, evidence for more heavily affected individuals in our ancient Egyptian assemblage clearly implies significantly less favourable conditions of housing and feeding, an issue we will return to below.

**Fig 14:**
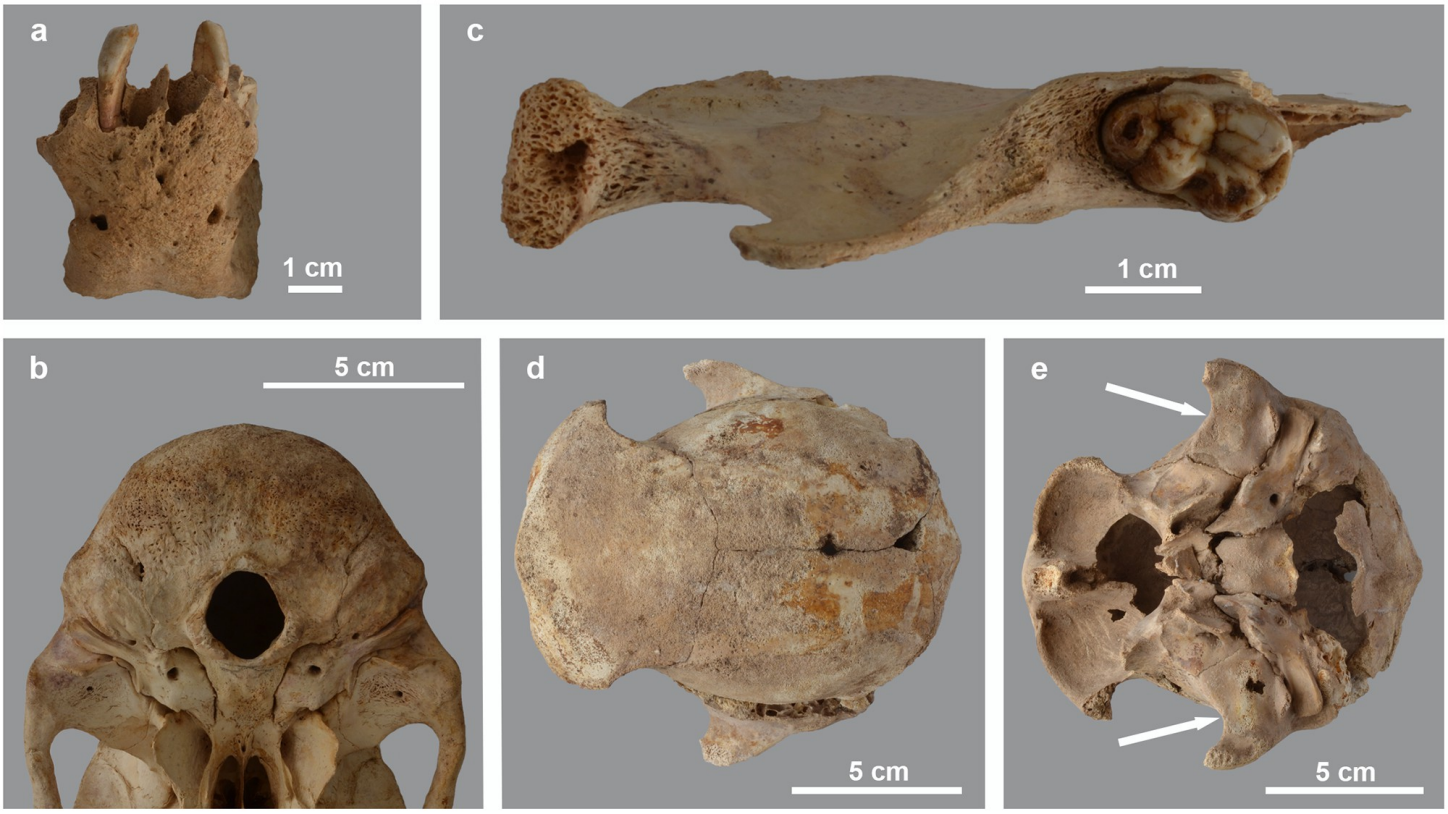
Surface modifications observed on mandibles and skulls. a) anterior view of the mandible of *P*. *hamadryas* male MHNL 51000174 with rugose surface of the chin; b) ventral view of the hind skull of *P*. *anubis* female MHNL 51000175, showing the cribriform aspect of the occipital area and the spongy aspect of the glenoid fossae (circular holes are artefacts); c) dorsal view of the right mandible half of *P*. *anubis* female MHNL 51000175, showing reticulated bone tissue posterior of the M3 and near the processus articularis; d) dorsal view of the skull of *Papio* sp. MHNL 51000318, showing the pitting of the frontals and parietals; e) ventral view of the skull of *Papio* sp. MHNL 51000318 with indication of the pitted glenoid fossae.

Of the eight skulls with neurocranial lesions collected in Gabbanat el-Qurud, only one individual, a male hamadryas (MHNL 51000174), has an associated mandible. The latter exhibited a deformation as well, namely a pronounced rugosity of the chin region (Fig 14a). Another type of surface modification is seen in a female anubis baboon (MHNL 51000175) exhibiting an irregular pitting of the occipital area (Fig 14b). The same specimen also shows reticulated bone tissue in the left and right glenoid fossae and on the maxillae behind the third molars. Moreover, on the rather completely preserved right half of the mandible, spongeous bone is visible on the horizontal and vertical rami and on the articular condyle (Fig 14c). In another case (MHNL 51000318), the macroscopic lesions observed on the skull are reminiscent of porotic hyperostosis, i.e. osteolysis of the external skull condition resulting in a fine porous, sieve-like or coral-like appearance (cf. Photo 30 in [42]) (Fig 14d and 14e).

**Fig 15:**
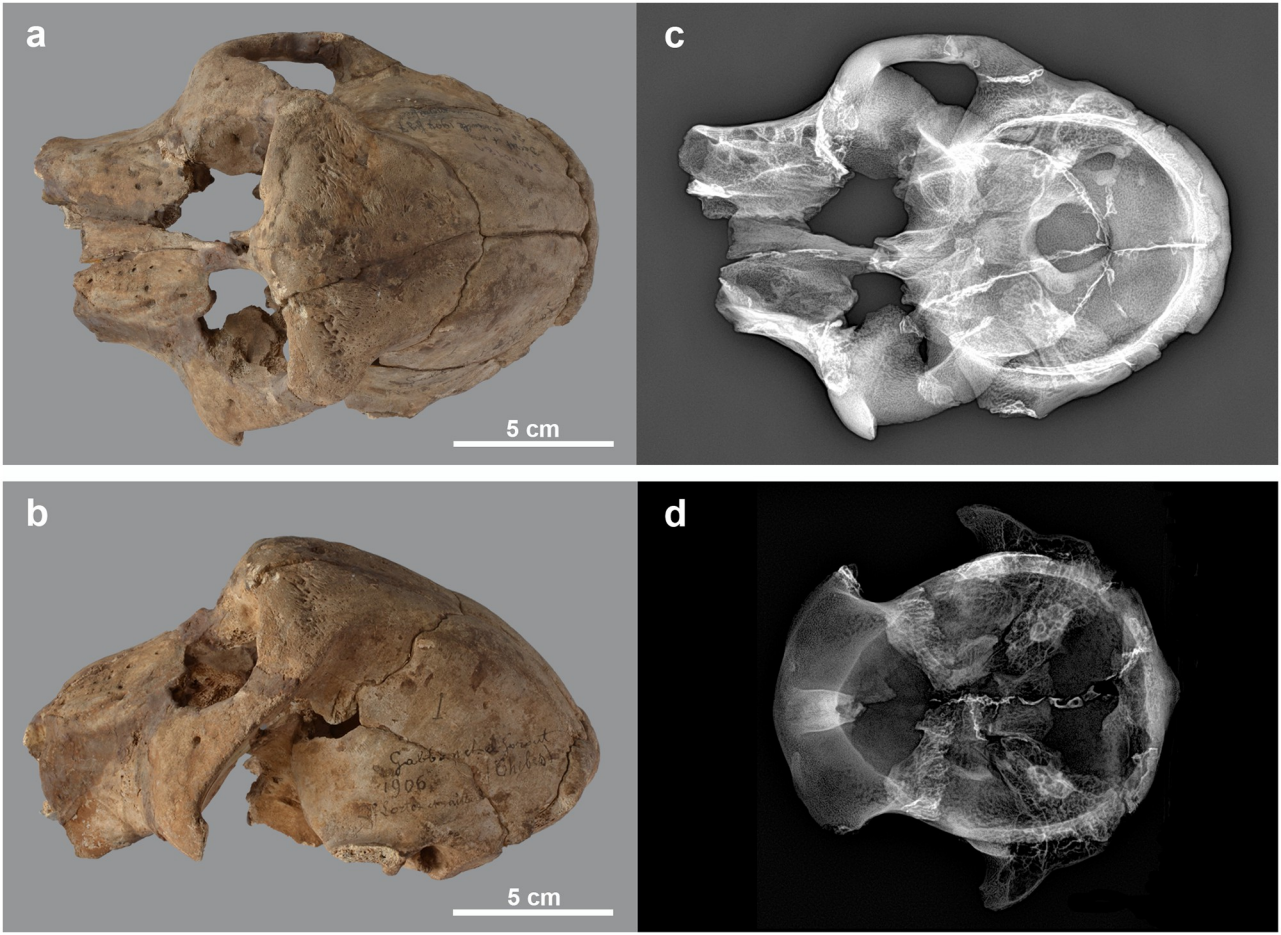
Thickening of the fronto-parietal region in two baboon skulls. Dorsal (a) and left lateral (b) view of skull of *Papio* sp. MHNL 51000181. X-ray in dorso-ventral view of the skull of *Papio* sp. MHNL 51000181 (c) and *Papio* sp. MHNL 51000318 (d), showing the (X-Rays: R. Lichtenberg).

Another cranial osteopathy observed in the assemblage concerns the marked thickening of the skull bones in specimen MHNL 51000181, diagnosed as Paget’s disease by Dr. Poncet (in [19]) (Fig 15). The thickening of the frontal and parietal bones, considered typical of the sclerotic, i.e. final stage of the condition, is apparent on X-ray (Fig 15c). A milder case of thickening, shown in Fig 15d, was observed in a young individual (MHNL 51000318). Possibly, we are dealing here with the intermediate, so-called mixed stage of the same condition [43]. Theoretically, histological sections under polarised light showing a chaotic arrangement of collagen fibres would be very helpful for the diagnosis of Paget’s disease, but the state of preservation of this ancient Egyptian specimen is not very promising for this approach.

**Fig 16:**
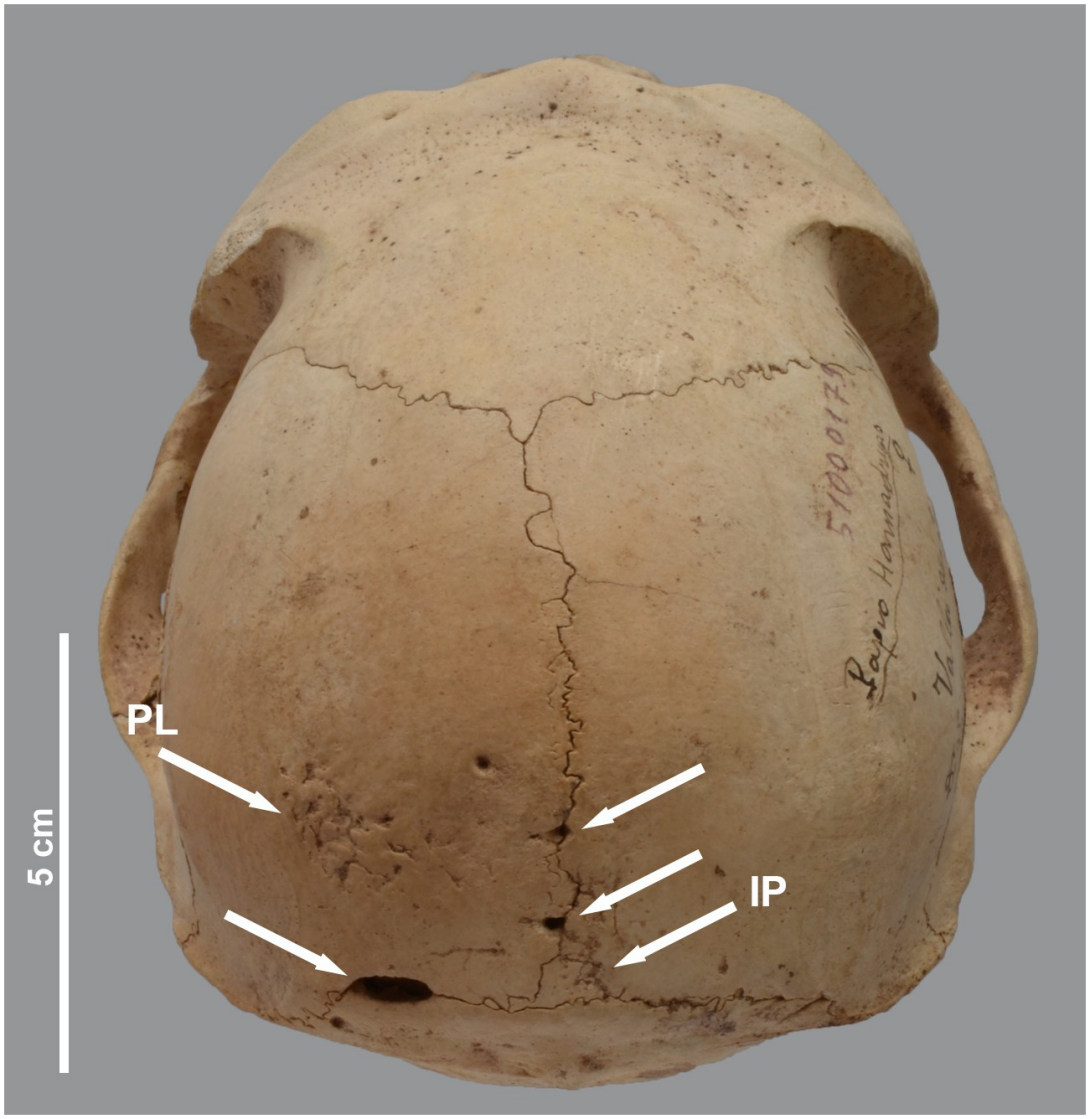
Pathologies in the skull of *P*. *hamadryas* female MHNL 51000179. Posterior view, showing a lesion (PL) on the left parietal and three perforations along the parietal sutures due to osteomalacia, and the presence of an interparietal (IP).

Finally, the skull of a female *P*. *hamadryas* (MHNL 51000179) shows modifications that likely can be diagnosed as osteomalacia (Fig 16). Three perforations occur along the parietal suture and there is a lesion on the left parietal.

#### Osteopathies in the craniofacial region and mandible

**Fig 17:**
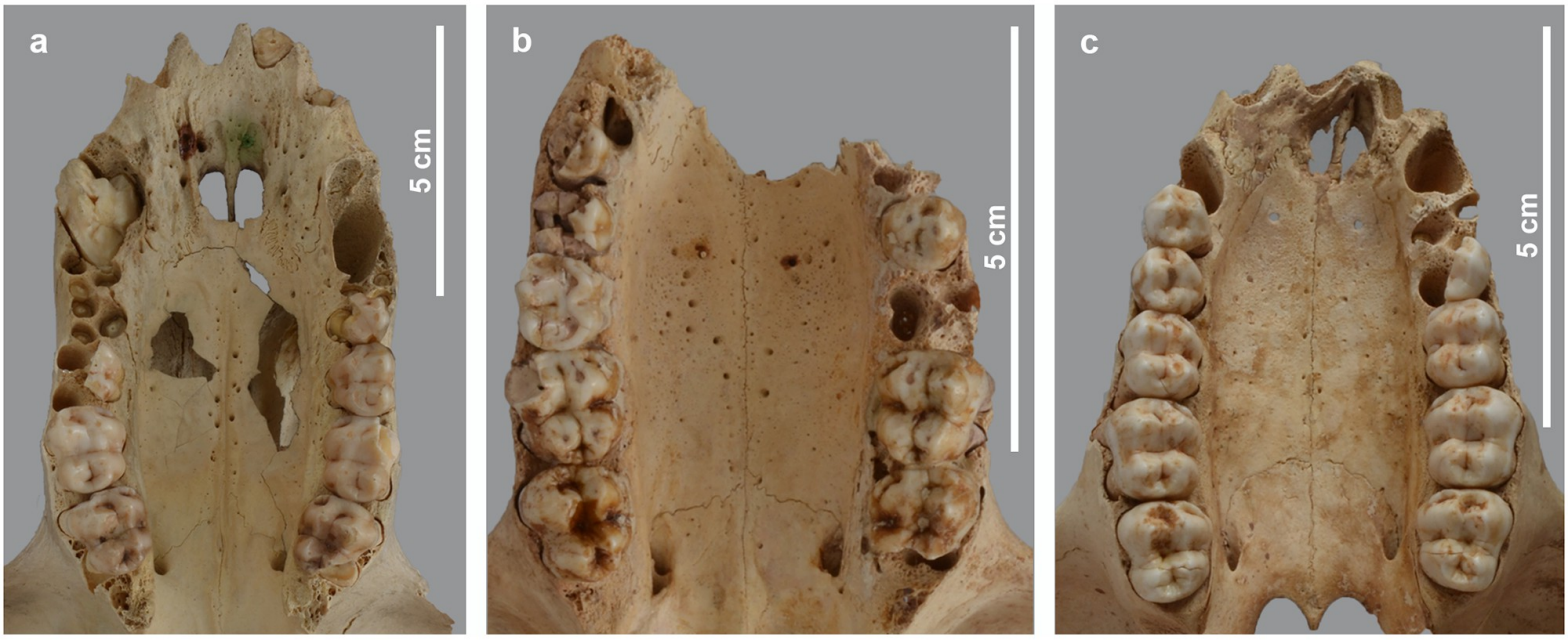
Irregular surface and pitted aspect of the palatine in three baboons. Ventral view of the viscerocranium of *P*. *anubis* male MHNL 51000173 (a), *P*. *anubis* female MHNL 51000175 (b) and *P*. *hamadryas* female MHNL 51000179 (c) showing the irregular surface and pitted aspect of the palatine. The swelling of the buccal rim delimiting the maxillary dentition is best seen in (c). The snout of this individual is distorted to the left.

**Fig 18:**
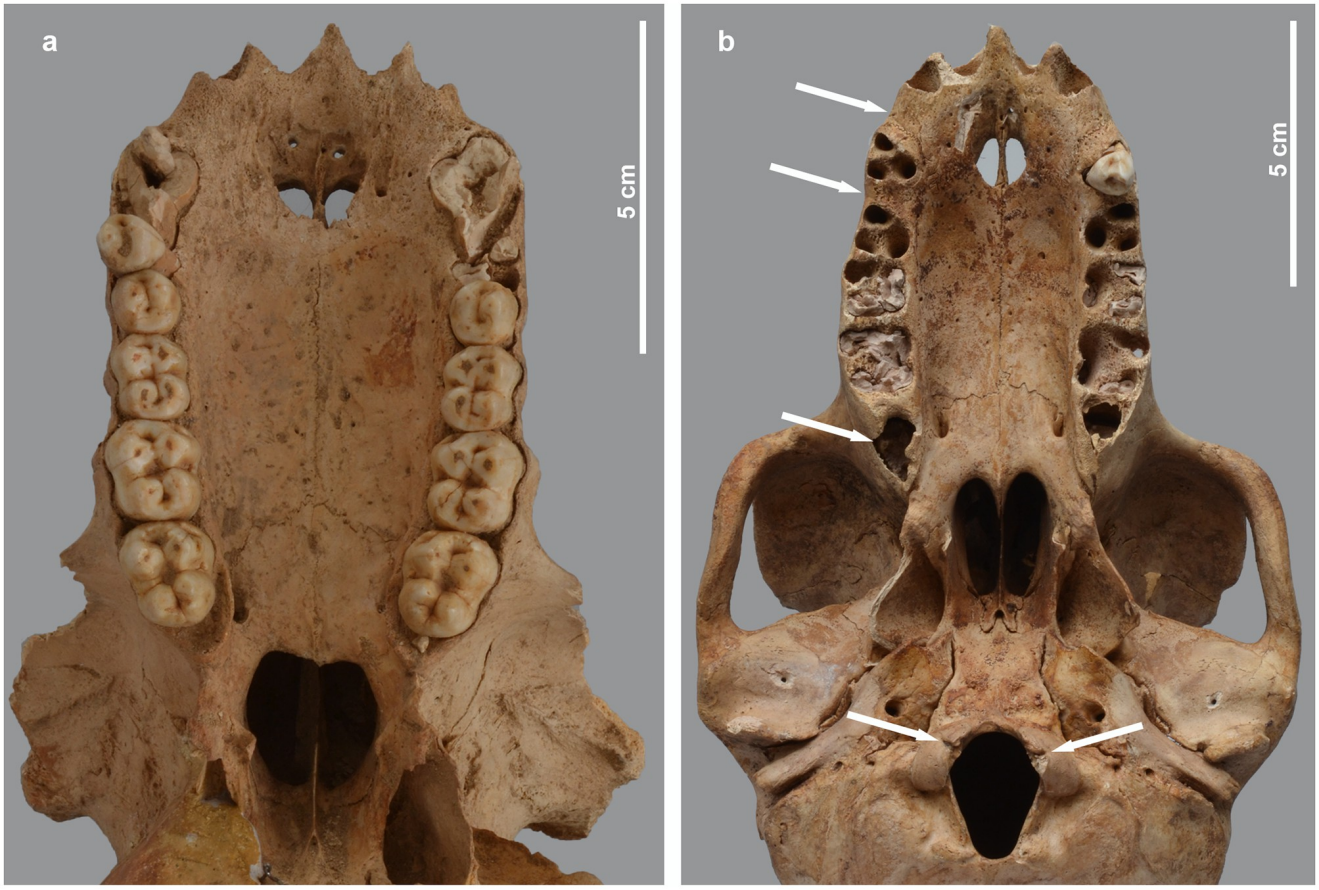
Osteopathies in the craniofacial region of two baboons. Ventral view of the skull of a) *P*. *hamadryas* male MHNL 51000174, showing the shortened viscerocranium, the rugose surface of the palatine, as well as the poorly developed P3; and of b) *P*. *anubis* female MHNL 51000320c, showing a clear gap between the I2 and the P3, as well as between the P3 and P4. The poorly developed M3 are still in their crypts. The occipital condyles show a fissure.

**Fig 19:**
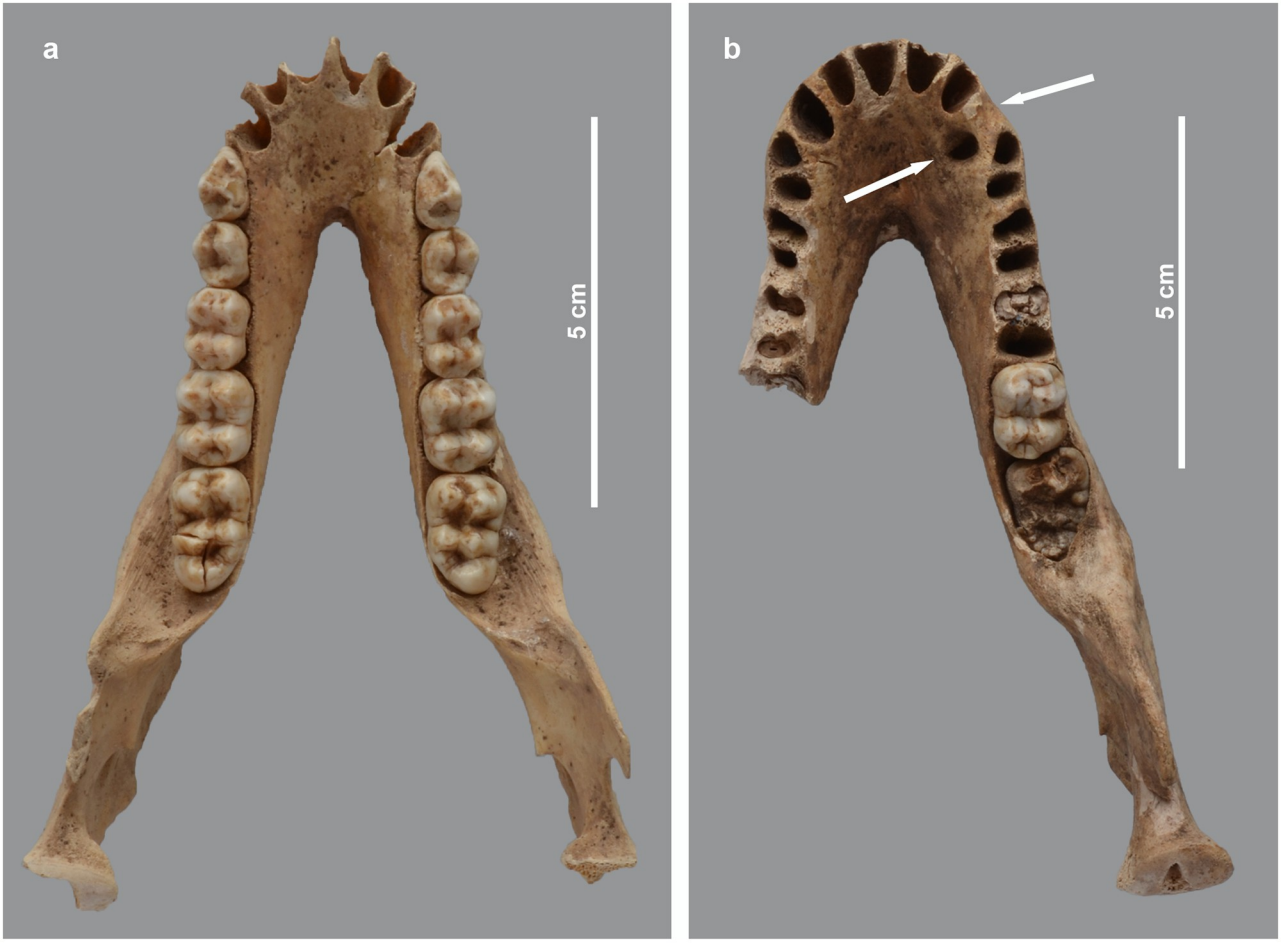
Osteopathies in the mandible of two baboons. Dorsal view of the mandible of a) *P*. *hamadryas* female MHNL 51000179, showing the asymmetry in the rostral part, and b) *P*. *anubis* female MHNL 51000320c, showing the location where the right canine is missing, as well as the large perforation lingually of the tooth row. The defective crown of the right M3 is also clearly visible.

As with the neurocranium, metabolic conditions likely affected the craniofacial region as well, and in this case both species and sexes are involved. We consider the varying degrees of pitting or rugose appearance of the palatine surface (in MHNL 51000173, 51000174, 51000175 and 51000180) and the swelling of the buccal rim delimiting the maxillary dentition (MHNL 51000173, 51000179 and 51000180) to be manifestations of such metabolic disorders (Fig 17). Moreover, two individuals had their snout distorted to the left (MHNL 51000179, MHNL 51000183) (Fig 17c), while two individuals suffered from shortened craniofacial regions (MHNL 51000174, MHNL 51000320C) (Fig 18). The first shows a poorly developed left and right P3. In the second, an adult female anubis baboon, the upper canines are not visible and the upper M3s are still in in their crypts. Presumably, the germs of the canines did not develop further and remained in their sockets. We think this explanation–an idea suggested by the microdonty observed in the upper M3 –is more plausible than a diagnosis as agenesis or extraction. It is known from well-documented examples in the literature ([44], Fig 3d and 3e in S1 Text) that in the latter case, regenerative bone tissue would be visible at the surface of the maxilla at the level where the canines were positioned initially. In our specimen, however, the corresponding surface does not show any such formation, it is completely smooth. Thus, the absence of any reactive tissue explains why we abandoned the idea that the baboon keepers had extracted the canines. Moreover, in the mandible, an alveolus for the left canine is present, but on the opposite side this tooth was not present and there is a gap between the alveolus of the I2 and anterior alveolus of the P3 (Fig 19). However, in this right lower jaw, a cavity is observed lingually of the anterior alveolus of the P3. This opening may correspond to the location where the tip of the canine could eventually protrude.

The occipital condyles of the same individual show a distinct fissure between the basi- and exoccipital bones, either the consequence of a metabolic or a congenital disorder [17].

Some of the conditions observed in the craniofacial region have their counterpart in the mandible. Specifically, the mandible of specimen MHNL 51000179 is asymmetrical, consistent with the aforementioned distortion of the snout (Fig 19a). The mandible of MHNL 51000174 shows a rugose chin but has no dental defects (Fig 14a). The right canine of MHNL 51000320C is absent and its alveolus is lingually misaligned. In addition, the only observable M3 is not erupted and the development of its crown does not appear to be fully completed (Fig 19b).

**Fig 20:**
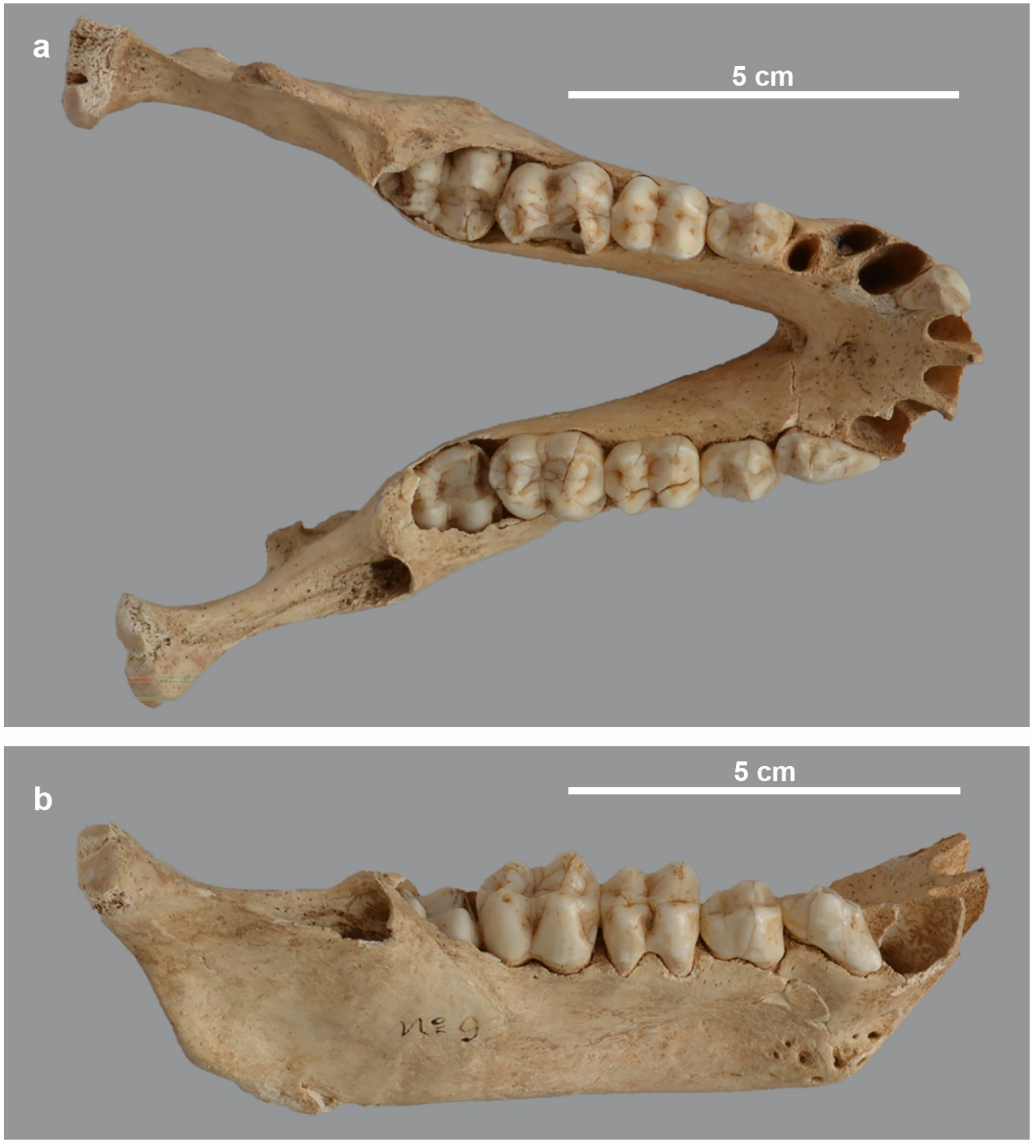
Asymmetry of the mandible of *P*. *anubis* female MHNL 51000176. Dorsal (a) and dorso-lateral (b) view showing the absence of the coronoid process and the distinctive osteocavitation in the right mandible half, posterior of the M3.

Another case of asymmetry was observed in the female anubis baboon MHNL 51000176, in which the right processus coronoideus is missing, causing asymmetric development of the glenoid fossae (Fig 20). Lortet and Gaillard [18] also mention this anomaly and believe that the processus coronoideus broke when the individual was young. Subsequently, the retractor muscles of the mandibles must have exerted pressure on the processus coronoideus, preventing this apophysis from fusing with the vertical ramus. After some time, it must have slid into the cavity that is visible behind the last molar. We think an alternative explanation is possible, namely that the processus coronoideus was embedded in the temporal muscle throughout the animal’s entire life and that it was lost post-mortem. This loss may have occurred when the soft tissues were decomposed and degraded thus exposing the bone fragment, which was overlooked when the skull was excavated due to its small size and unspecific shape.

**Fig 21:**
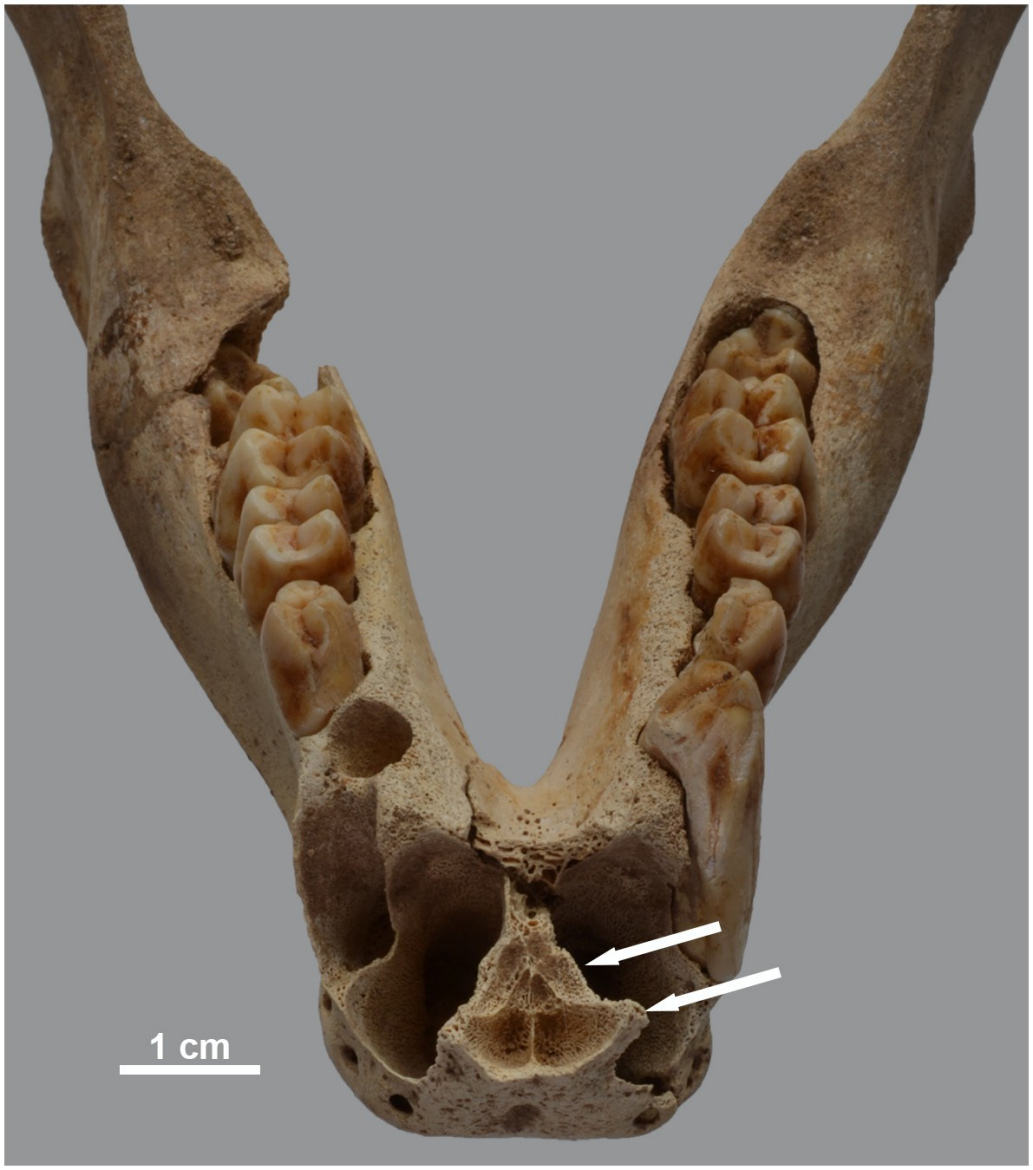
Dorso-anterior view of the mandible of *Papio* sp. male MHNL 51000315 + 504. Two large and two small alveoli for the incisors are present.

In an isolated mandible of a male *Papio* sp. (MHNL 51000315 + 504), dental misalignment was observed (Fig 21). The alveoli of the incisors align pairwise behind each other instead of side-by-side.

#### Caries, linear enamel hypoplasia, metopism and tumor

**Fig 22:**
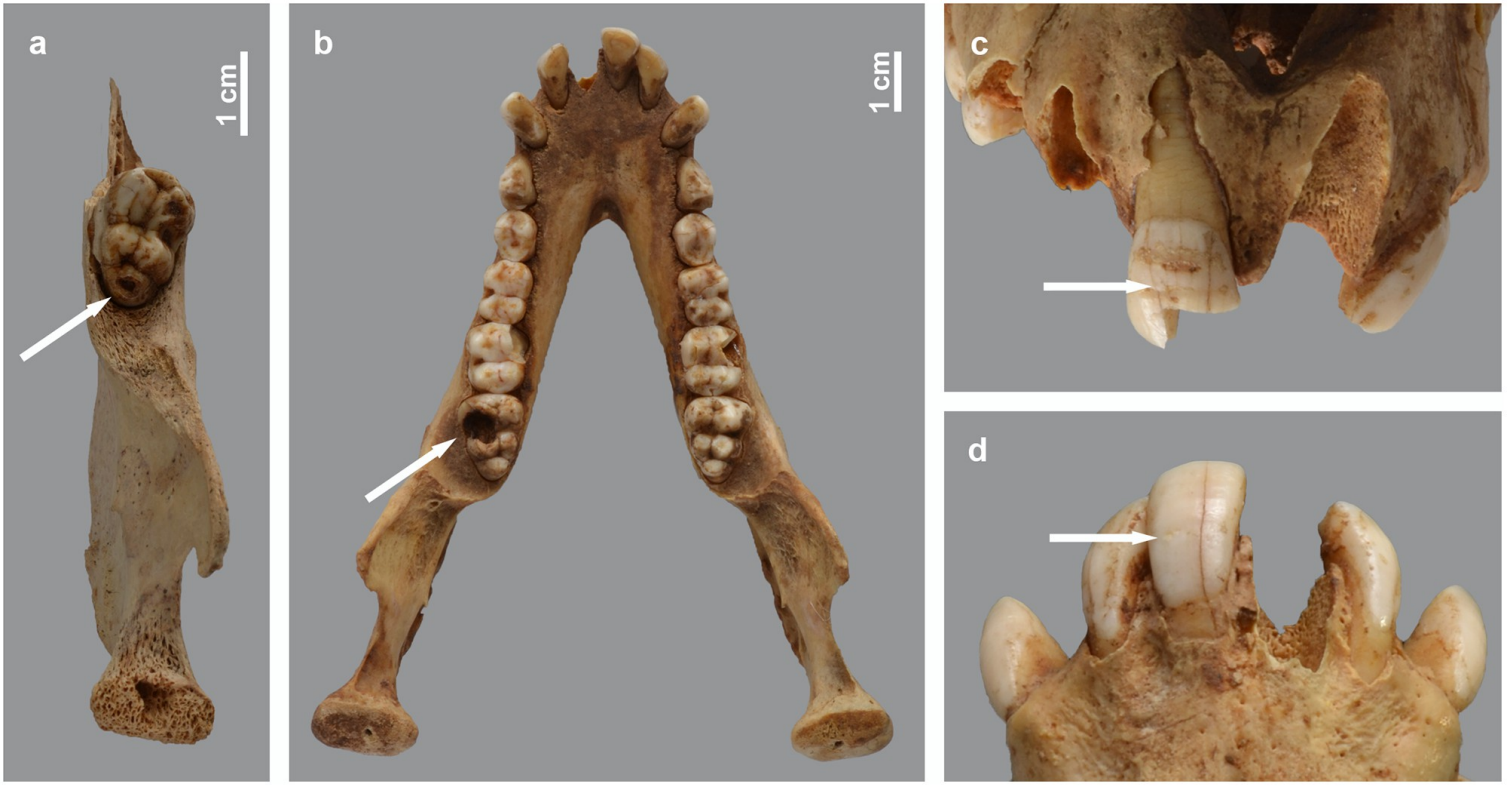
Non-metabolic dental pathologies observed in the baboons. Caries in the right lower M3 of *P*. *anubis* female MHNL 51000175 (a) and in the left lower M3 of *P*. *hamadryas* female MHNL 51000172 (b). Hypoplasia lines in the right upper (c) and lower (d) first incisor of *P*. *hamadryas* female MHNL 51000172. Above the hypoplasia line of the upper incisor is a linear deposition of dental calculus.

Besides the aforementioned pathologies considered the result of metabolic disorders, relatively few pathologies with a distinct aetiology have been observed. Dental problems unrelated to metabolic diseases involve two cases of caries, the first in a right lower M3 of a female anubis baboon (MHNL 51000175) and the second in a left lower M3 of the aforementioned female hamadryas (MHNL 51000172) (Fig 22a and 22b). The latter individual is also the only baboon with linear enamel hypoplasia (in the right lower and upper I1) in an otherwise healthy animal (Fig 22c and 22d).

**Fig 23:**
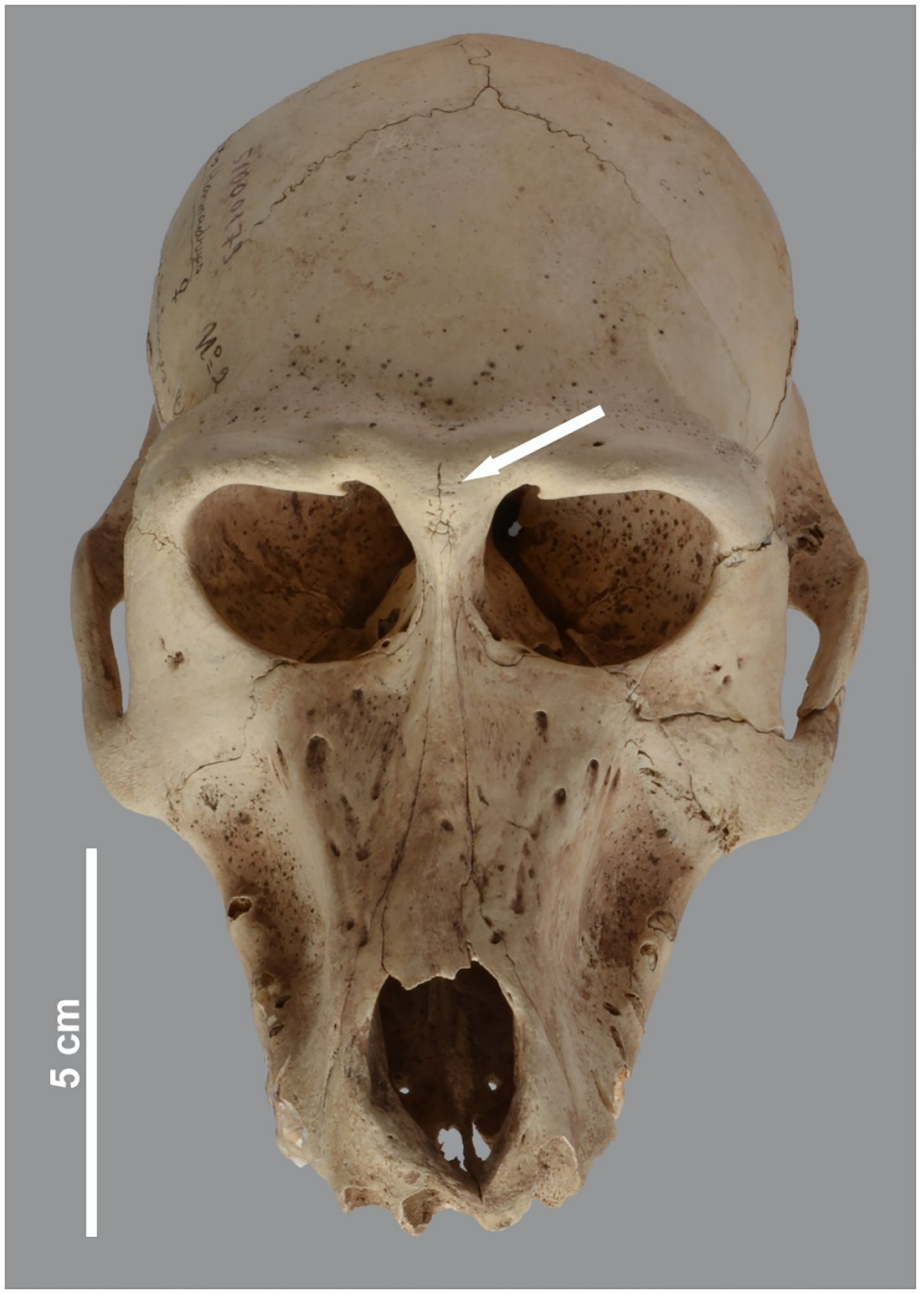
Non-metabolic pathologies observed in the skulls. *P*. *hamadryas* female MHNL 51000179 in dorso-frontal view, showing the partial metopic suture at the level of the glabella.

We also noted six cases of persistent metopic sutures, two of which were partial (Fig 23). One of these specimens with a partial mesotopic suture, a female hamadryas baboon (MHNL 51000179), has an interparietal bone (Fig 16). Presumably, the incomplete fusion of the parietal sutures, which show several holes, is related to this anatomical particularity. Interestingly, a study discussing the two phenomena in humans suggests a correlation between the occurrence of persistent metopic suture and evidence for the interparietal bone [45].

**Fig 24:**
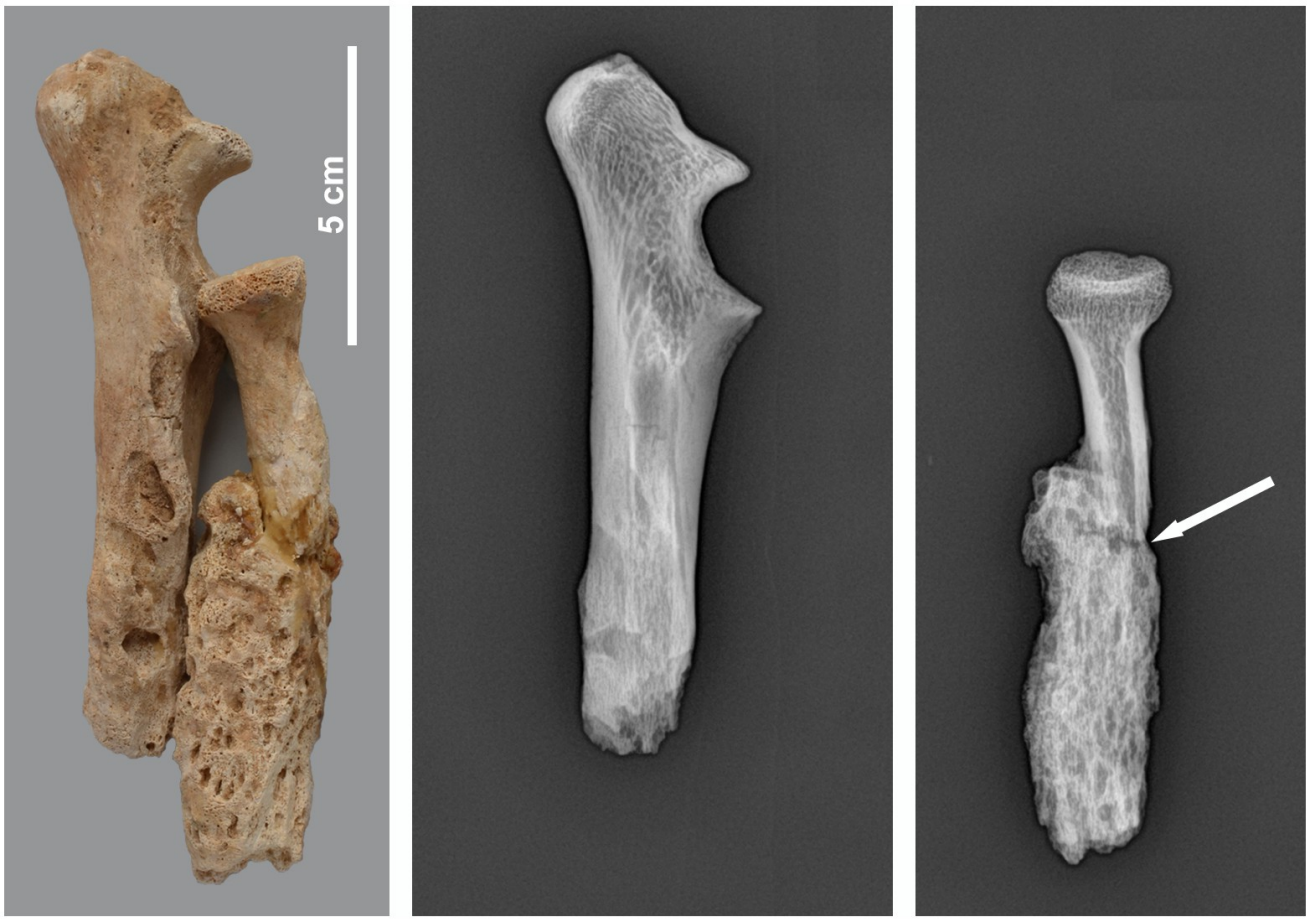
Lateral view of the right radius and ulna of *Papio* sp. MHNL 51000324. The pathology was diagnosed as a ‘sarcome périostique ossifiant’ in [19]. The glue that was used to mend the recent break of the radius can be clearly seen. On the X-ray, the arrow seems to indicate a transversal fracture of the radius but is in fact an artefact, due to a recent break.

The pathology on a right radius and ulna (MHNL 51000324) (Fig 24) was diagnosed by Poncet (in [18]) as being a ‘sarcome périostique ossifiant’. Nerlich et al. [46] suggested that it could be an ‘exuberant (post-traumatic) callus formation rather than a malignant tumor’. We made a radiograph that at first sight seems to indicate the presence of a fracture underneath the callus. The general, outer appearance of the ulna and in particular the radius is very irregular and, if one prefers to diagnose it as a callus, the fracture must have occurred shortly before the baboon’s death. However, the radiograph that we made shows no evidence for a fracture. In fact, the disruption visible on the image is caused by a fresh, modern break that can be seen with the naked eye and that has been glued. This specimen was mostly likely broken during or after the excavation.

#### Trauma

**Fig 25:**
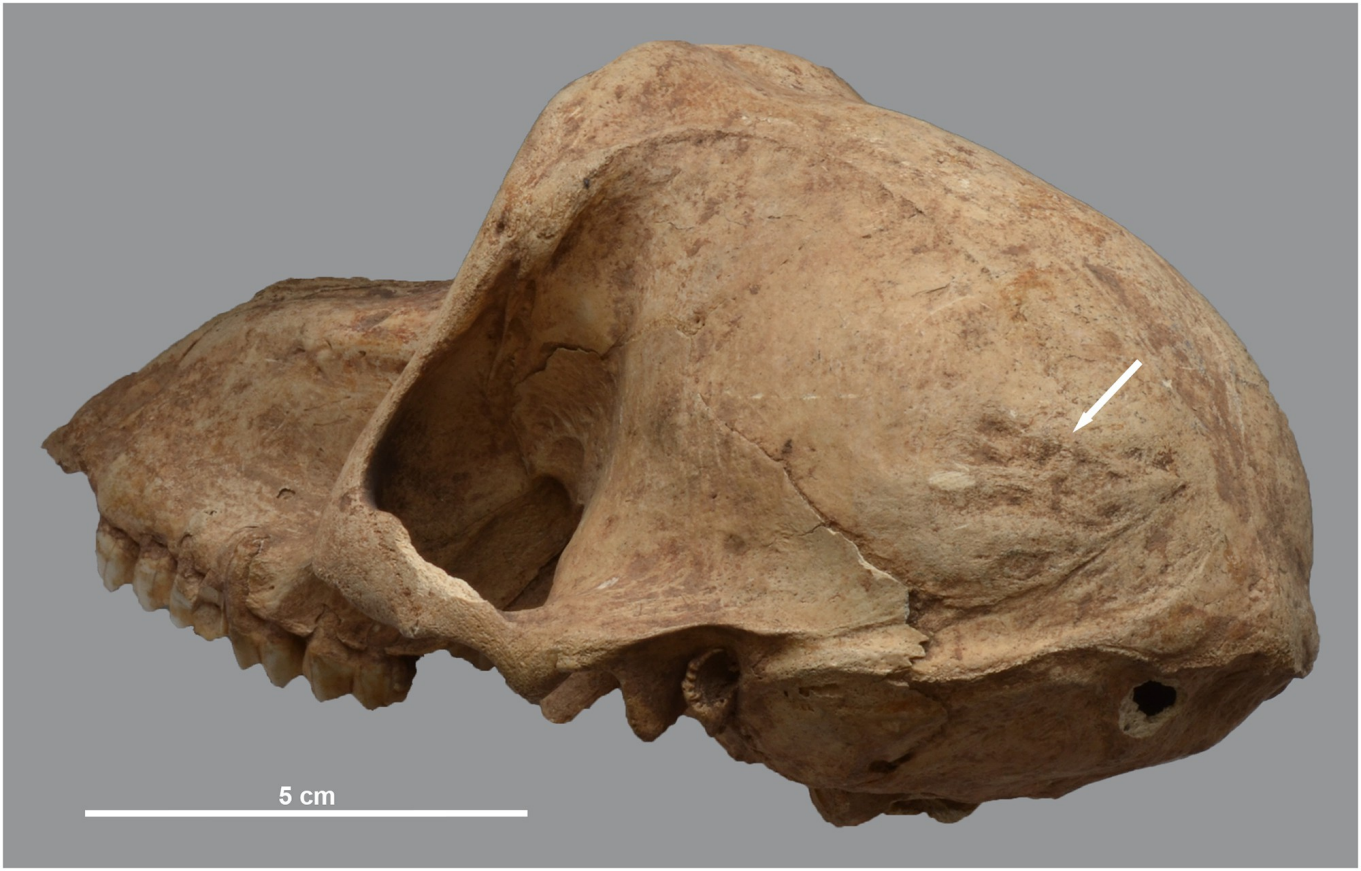
Traumatic lesion on skull. Localized lesion on the left parietal of *Papio hamadryas* MHNL 51000170.

The only case of trauma observed is a localized lesion on the left parietal bone, perhaps a result of violent blows received, in a female hamadryas baboon (MHNL 51000170) (Fig 25). No indication of trauma was found on the postcranial material.

## Discussion

In view of the incidence of bone pathologies in the Gabanat el-Qurud assemblage, aetiology and pathogenesis characterizing the different disorders will be discussed first. We then compare the incidence of metabolic and traumatic osteopathies to similar findings reported from archaeological sites elsewhere in Egypt, more precisely in the animals from Predynastic Hierakonpolis, Saite to Late Ptolemaic Tuna el-Gebel and Ptolemaic Saqqara. Based on the age and sex distribution of the baboons, we then address the potential role of long-distance trade *versus* local breeding of animals at these sites and conclude with speculations about the possible origin of those baboons that were ultimately destined to be mummified and deposited in Egyptian animal cemeteries.

### Feeding and keeping baboons

From our observations, it becomes clear that several adverse factors affected skeletal health in the baboons from Gabbanat el-Qurud. Because bone growth and maturation are complex processes in which genetic factors, local and systemic hormones, dietary nutrients and mechanical forces interact, any imbalance in the availability or ratio of chemical elements, vitamins and hormones [39, 44, 47] or limitations imposed by life in captivity (e.g. [48, 49]) can cause osteopathological disorders. Mode and degree of expression may vary, though, depending on the individual’s age, phase of skeletal development and duration and intensity of the imbalance. It follows that the range of skeletal disorders is considerable, with even single causes varying markedly in their manifestation [39]. To complicate matters, post-mortem examinations mostly ignore the skeleton, because maceration is very time-consuming and cost-intensive and thus limited to those cases wherein skeletal health issues have been diagnosed during a patient’s clinical history. That said, current medical research may help determine the aetiology and pathogenesis of past skeletal disorders, for example in the elderly where long-term severe vitamin D deficiency causes secondary hyperparathyroidism. This is visible in high bone tissue turnover and demineralisation that can no longer be compensated by new bone formation, thus resulting in osteomalacia (e.g. [50]), although reversal is possible [51]. Another major limiting factor for our retrospective study of disorders of ancient Egyptian baboons is, the ‘dry’ nature of our materials, which excludes valuable companion studies such as histology of fresh bone.

Contrary to healthy bone tissue showing metabolic activity directed toward maintenance of ionic equilibrium, repair of damaged structures and reaction to external stimuli, the changes observed in diseased bone are largely due to alterations in the processes by which they are formed [47]. Individuals can even suffer from several disorders simultaneously, whereby each of these can be caused by more than one nutritional or endocrine imbalance. In practical terms, lesions reported in rickets and osteomalacia may overlap with those noted in other metabolic bone disorders and vice versa. Metabolic bone diseases moreover seldom result from a deficiency of a single dietary nutrient, but rather from a deficiency of several nutrients or a dietary imbalance in the ratio of calcium to phosphorus [39]. Modern textbooks on pathology therefore place conditions such as rickets, osteomalacia, fibrous osteodystrophy and osteoporosis under the heading metabolic bone diseases. This takes into account that some aetiologies can be responsible for various metabolic bone diseases [39]. For example, deficiency of vitamin D, known to cause rickets in adolescents and osteomalacia in adults, will also lead to fibrous osteodystrophy, due to reduced calcium absorption from the intestine. The latter disorder as well as osteoporosis will develop in case of persistent dietary deficiency of calcium, but the degree of expression depends on age and species. Prolonged calcium deficiency may also cause dental abnormalities including delayed eruption and increased susceptibility to wear resulting from mild enamel hypoplasia. Whereas a deficiency of phosphorus causes rickets and osteomalacia, the element’s chronic deficiency may evoke a combination of osteomalacia and osteoporosis in adult individuals. Excess dietary phosphorus in turn may cause nutritional secondary hyperparathyroidism and fibrous osteodystrophy. Persistent severe undernutrition may go along with osteoporosis, i.e. reduced bone formation and increased resorption. The same cause also effects tooth development, expressed by delayed formation and eruption of the whole dentition, with a relatively greater delay in the growth of the jaws, though, causing overcrowding of the teeth, especially the permanent molars. This results in malocclusion and malalignment and even partial or complete elimination of the diastemata between incisors and canines and between canines and deciduous molars [39].

All in all, the challenges faced by the caretakers of the baboons in Gabbanat el-Qurud were multifaceted, and since our study can only assess the skeletal remains, other health issues remained largely hidden, such as parasitoses and zoonoses. Consequently, our study can only provide a glimpse of how problematic primate keeping must have been in reality. Serious nutritional and endocrine disequilibria help explain the metabolic bone disorders observed, with calcium and vitamin D deficiencies as prominent factors. Since the latter and its metabolites play a key role in activating transcription factors that regulate calcium metabolism and bone formation, insufficient exposure to sunlight (UV B) is probably the key to most problems. In wild baboons sun-exposure is a widespread behaviour that certainly promoted vitamin D synthesis, but such a scenario seems questionable when dealing with captive populations. As baboons are good climbers, and at the same time aggressive animals and therefore dangerous in captivity, high-walled and roofed enclosures were likely essential to keep them indoors. Walled corridors of large temple buildings, for example, offered shady conditions and limited exposure to sunlight. An unbalanced, inadequate diet and painful skeletal disorders may have promoted aggression in captive baboons, and it is possible that the lesion observed on one of the skulls is due to injuries inflicted with a stick.

In summary, the metabolic bone disorders observed in the ancient Egyptian baboon population presented here point to a combination of nutrient and hormone imbalances, chronic malnutrition and the adverse conditions of a life in captivity offering poor opportunities for sun exposure.

### Comparison of the findings at Gabbanat el-Qurud with other places of baboon worship

High frequencies of similar metabolic disorders are observed at Saqqara and Tuna el-Gebel. Looking at the baboons from Tuna el-Gebel, it appears that in the oldest, Saite period, 15% of the animals were affected by metabolic bone disorders. This number increased to 26% in the Late Dynastic/Early Ptolemaic periods, and to 46% in the Late Ptolemaic period [17]. It has been suggested that this significant increase in pathologies may be due to a higher degree of inbreeding necessitated by difficulties in obtaining more wild animals (see below). For Saqqara, a diachronic breakdown of the pathologies has not been presented, but no fewer than 82% of the skulls obviously reflected metabolic disorders [14]. At Gabbanat el-Qurud, 56% of the individuals reveal evidence of metabolic deformities, a proportion between those in Saqqara and in Late Ptolemaic Tuna el-Gebel.

Compared to the metabolic disorders, incidences of trauma at Gabbanat el-Qurud, Saqqara and Tuna el-Gebel are relatively low. At the first site, a lesion on the hind skull points to trauma, possibly caused by interactions of the animals with their caretakers. At Tuna el-Gebel, only 5 out of the 227 baboons had suffered from injuries that left traces on the skeleton: a healed fracture was observed on a mandible and on the supra-orbital margin of a skull; on another skull a perforation was found showing traces of healing. As for post-crania, healed injuries have been noted in a scapula and a clavicula [17]. Traumatic incidences are also low among the estimated 180 monkeys at Saqqara. The only healed fracture observed concerns a humerus. Three skulls (two of females, one of a male) show healed injuries due to blows the baboons received, while three additional skulls exhibit damage presumably self-inflicted after osteomalacia had softened the bone [16].

Very little information is available on the actual physical characteristics of the structures in which the baboons may have been confined, but little direct sunlight in the places where the animals were kept may have been the rule. For Gabbanat el-Qurud, there is no textual or archaeological evidence for any buildings or other structures. In the case of Saqqara, six monkey obituaries written in demotic and dated to the second century BC are available in which the Temple of Ptah-under-his-Moringa-Tree is mentioned in connection with the baboons [52]. Mummification took place in this subsidiary temple in Memphis, but apparently baboons also lived and reproduced here. However, archaeological evidence for such structures is lacking. At Tuna el-Gebel, in a building connected to the Osiris-Baboon temple, a small room measuring 2 by 3 metres has been found that was described as an ‘animal cage’ [53]. This small structure, made of limestone slabs and only roughly datable to the Roman period, had a small lockable window through which the enclosed animal(s) could be observed and fed. The authors mention that either sacred ibises or a small baboon may have been kept in there. Another indication as to how baboons were housed can be found in a Thutmosid palace at Tell el-Dabʿa, where an in situ burial of an adult male baboon was discovered [54]. This chamber was connected to another room and there was also an exit outside the palace that must have allowed the animal(s) to move around.

The conditions of baboon keeping in all sites were probably also dictated by their behaviour. This may not have been easy because they may become very aggressive, as illustrated by the filed down canines of the Tel el-Dab’a male [54]. A comparable measure may have been the extraction of canines mentioned for three hamadryas baboons from the royal tombs in the Valley of the Kings [44]. When animals were captured young, or when they were born in captivity, it may have been easier to handle them.

All observations from Gabbanat el-Qurud, Saqqara and Tuna el-Gebel are in marked contrast to what is seen in the baboons found in burials of the elite cemetery HK6 at predynastic Hierakonpolis. A high number of healed fractures is noted there and no evidence of metabolic problems is found [26]. This suggests the animals represented direct imports from the south and that no local breeding occurred. No injuries to the head were observed, but skulls are poorly represented and evidence of blows to the head may therefore be missing. Among the mandibles, however, occurs a specimen with a healed fracture and several other lower jaws had lost teeth in vivo, possibly due to blows received or because of gum infections. Two individuals showed a parry fracture in their left forearm and there was one case of a healed fracture on a clavicula. All the other injuries observed occur on the hands and feet. In the oldest burial (HK6, Tomb 12), 45 healed fractures were observed on baboon extremities. No less than 14.2% of the preserved metapodals, first and second phalanges showed healed fractures. In two younger graves, namely Tomb 17 (MNI 1) and Tomb 53 (MNI 6), many fewer such fractures occur (4.5% and 2.7% respectively). It is worth noting that in all three tombs, metacarpals were more often fractured than metatarsals, indicating that the hands were most frequently targeted. The higher incidence of trauma in the oldest burial (Tomb 12) compared to that in the probably younger Tombs 17 and 53 suggests that people became more dexterous or humane in dealing with baboons over time. None of the 75 metapodals and 12 first phalanges from Gabbanat el-Qurud, available in the collections of the Musée des Confluences, had a healed fracture. The description of the baboon skeletal material from Saqqara or Tuna el-Gebel does not mention metapodials or phalanges, but we assume that if pathological specimens were present, this would have been reported. The low incidence of healed fractures in baboon skeletons from the Late Period onwards shows that violent contacts decreased, possibly because of the different housing that allowed reducing direct contact with the animals. This is suggested for instance by the ‘cage’ at Tuna el-Gebel mentioned above.

Although it is suggested that some of the metabolic deformities may be partly due to inadequate diet, we have little direct evidence for the kind of foodstuffs available to the baboons. It has been argued that qualitatively, the food must have been chronically unbalanced, compared to the extremely varied diet in the wild, and that if the baboons received leftovers from the kitchen, old, hard bread would have been a major component of their diet [17]. The carbon and nitrogen stable isotope data obtained on the three baboons from Gabbanat el-Qurud (Table 1) range between -18.6‰ and -20.0‰ (mean -19.1‰±0.7‰) for carbon and between 11.7‰ and 13.3‰ (mean 13.2‰±1.0‰) for nitrogen. These values fall within the range of those from a large number of human bone samples (n = 16) from the Nile Valley, dating between the Predynastic and the 30th Dynasty: between -18.4‰ and -20.8‰ for carbon and between 10.7‰ and 14.8‰ for nitrogen [55]. This suggests that the food the baboons received may have been comparable to that of the humans and that the diet was thus less monotonous than supposed in von den Driesch et al. [17]. An omnivorous diet is also suggested by the oral microbiome preserved in the dental calculus of the Gabbanat el-Qurud baboons [56].

### Import and local breeding

With the exception of four individuals that appear healthy, all other animals from Gabbanat el-Qurud have suffered from metabolic disorders. Although Brandon-Jones and Goudsmit [16] argue that the deformities such as rickets or osteomalacia may develop during the lives of baboons that were captured young, we consider it equally possible that they occur in next-generation individuals and that the pathological specimens are therefore offspring of animals raised in captivity. The four individuals at Gabbanat el-Qurud that show no pathologies probably represent ‘fresh’ wild-caught animals. Of particular interest among them is the female hamadryas baboon MHNL 51000172. The complete mitogenome of this individual was reconstructed and compared well with that of hamadryas from Eritrea, Ethiopia and eastern Sudan, although an origin from the Arabian Peninsula cannot be completely ruled out [57].This animal shows linear enamel hypoplasia (LEH) lines about halfway the crown of the first upper and lower incisors. This marker may well correspond to the stress that the animal underwent when it was captured and translocated immediately afterwards. Certainly, the deepest LEH lines in primates are observed in individuals born in the wild that had been transferred to captivity [58]. The first permanent incisors of baboons erupt at 33–35 months of age [32], but at that time the crown is already completely formed. Observations on two modern female baboons show that mandibular first incisors start developing at around 0.6 years of age and that crown formation is completed around 3–3.3 years of age [59]. Given the position of the lines in the specimen from Gabbanat el-Qurud, we suggest the animal was almost two years old when it was captured. Judging from the attrition stage of its molars, this baboon died when it was about 8 years of age. The animal developed normally, with the exception of a large caries in a lower third molar. No osteomalacia had affected this baboon.

**Fig 26:**
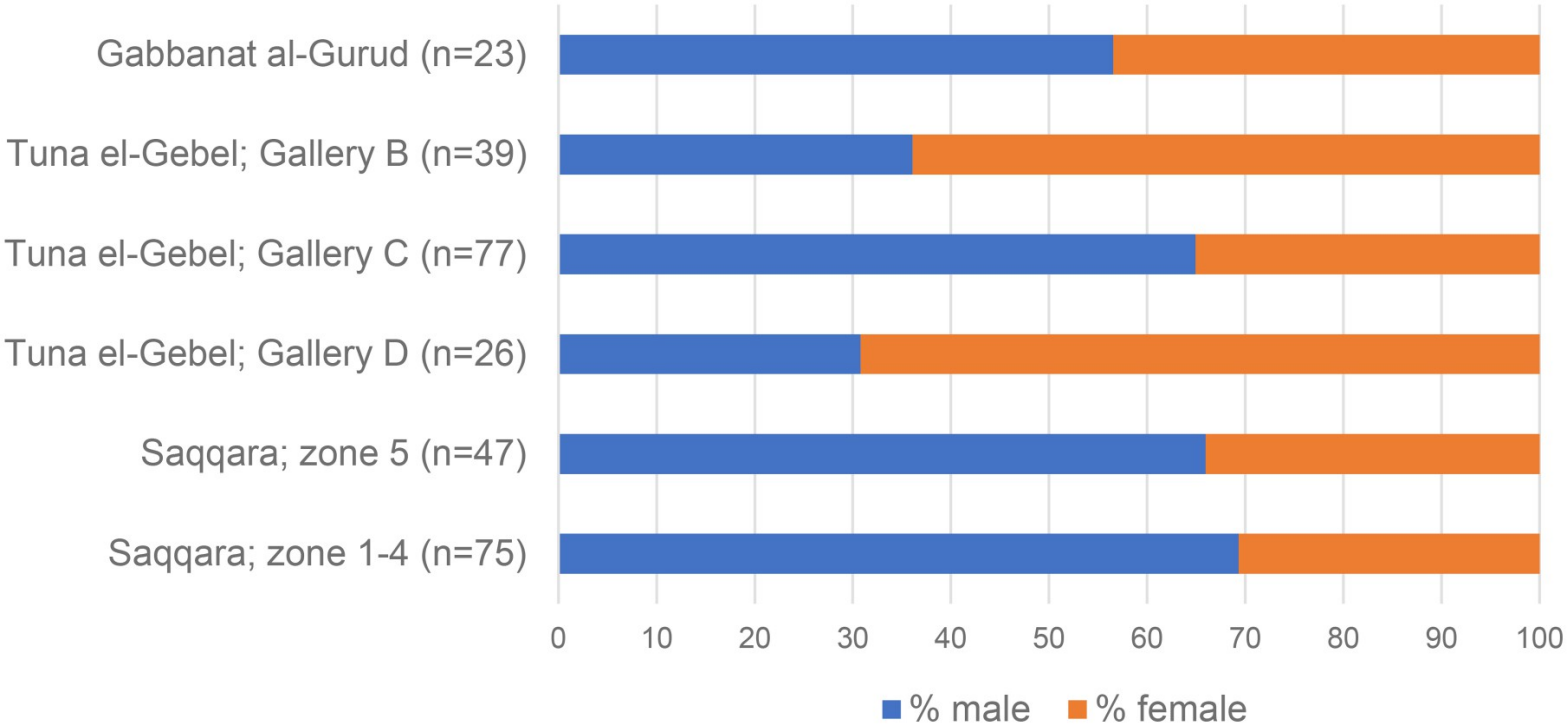
Sex ratio of the baboons at Gabbanat el-Qurud, compared to those of the Upper Galleries (zones 1–4) and Lower Gallery (zone 5) at Saqqara, and of the galleries B, C, and D at Tuna el-Gebel. The figures from Tuna el-Gebel are taken from Fig 37 of von den Driesch et al. [17], wherein no distinction was made between the animals from the Persian and Early Ptolemaic Periods in Gallery C.

The frequent occurrence of metabolic disorders in the other animals at Gabbanat el-Qurud is an indication that these baboons suffered from inadequate housing and food during most of their lives, and suggests that the animals were born in captivity. Other arguments for local breeding are the more or less equal proportions of males and females, as well as the presence of infants and juveniles [17]. At Gabbanat el-Qurud the sex ratio, based on the cranial remains, shows a slight predominance of males among the adolescent to adult animals: 10 of them are females and 12, possibly 15, are male individuals (Table 2). The somewhat higher proportion of males compared to females at Gabbanat el-Qurud is also seen at Saqqara and in Gallery C of Tuna el-Gebel (Fig 26).

**Fig 27:**
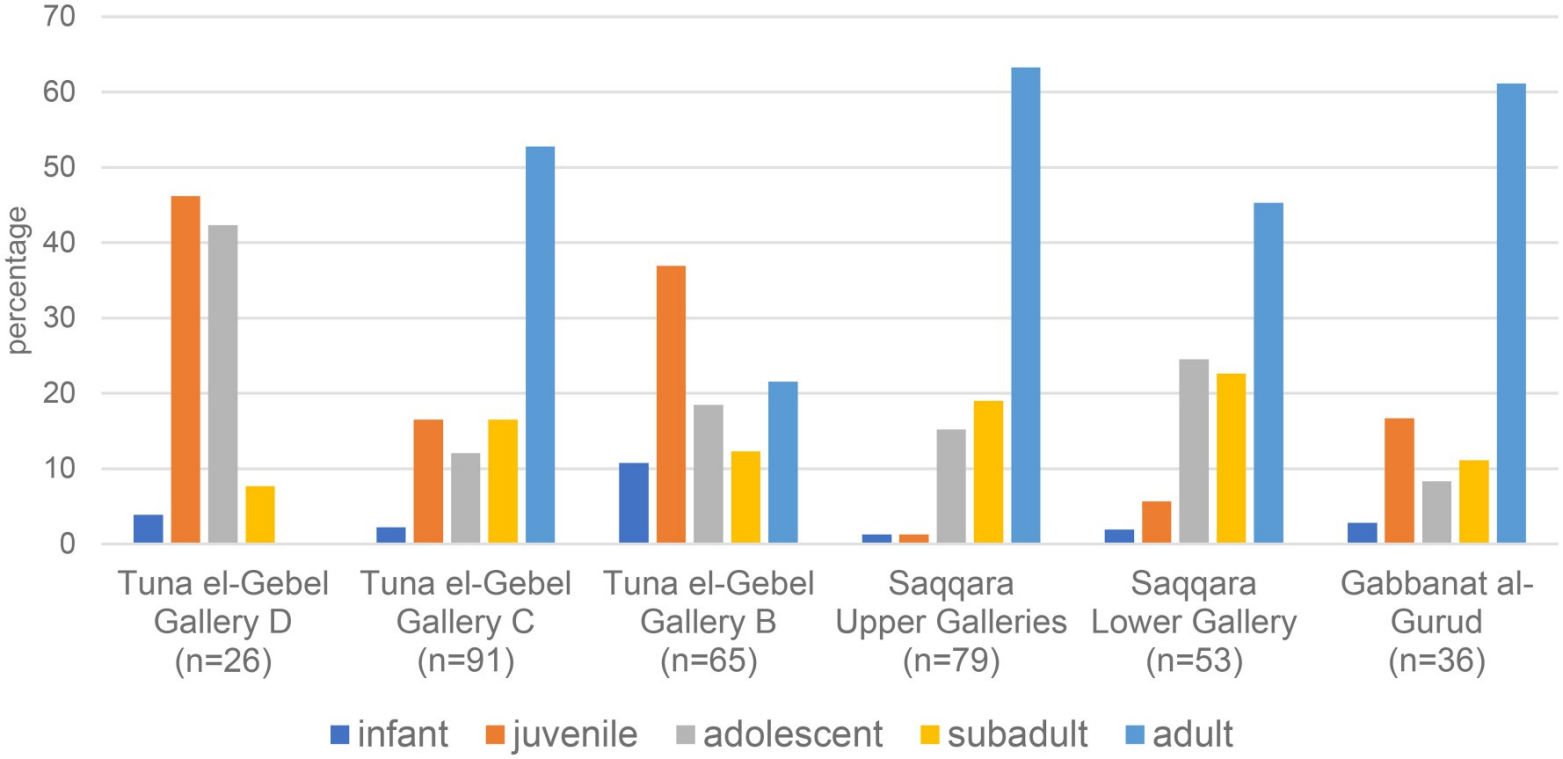
Proportion of the age classes of the baboons at Gabbanat el-Qurud, in the Upper and Lower Galleries at Saqqara, and in the three galleries at Tuna el-Gebel. The figures from Tuna el-Gebel are taken from Fig 36 of von den Driesch et al. [17] in which no distinction was made between the animals from the Persian and Early Ptolemaic Periods in Gallery C.

In Fig 27 the proportion of the different age classes are shown for the baboons of Gabbanat el-Qurud and for those of the three galleries at Tuna el-Gebel. It appears that the age distribution seen in gallery C of Tuna el-Gebel compares well to that of Gabbanat el-Qurud, with infants and juveniles representing about one fifth of the population. The proportion of infants and juveniles at Saqqara is low, an observation that led Brandon-Jones and Goudsmit [16] to the suggestion that the more fragile nature of their remains may have caused this bias.

### Provenance of the baboons

**Fig 28:**
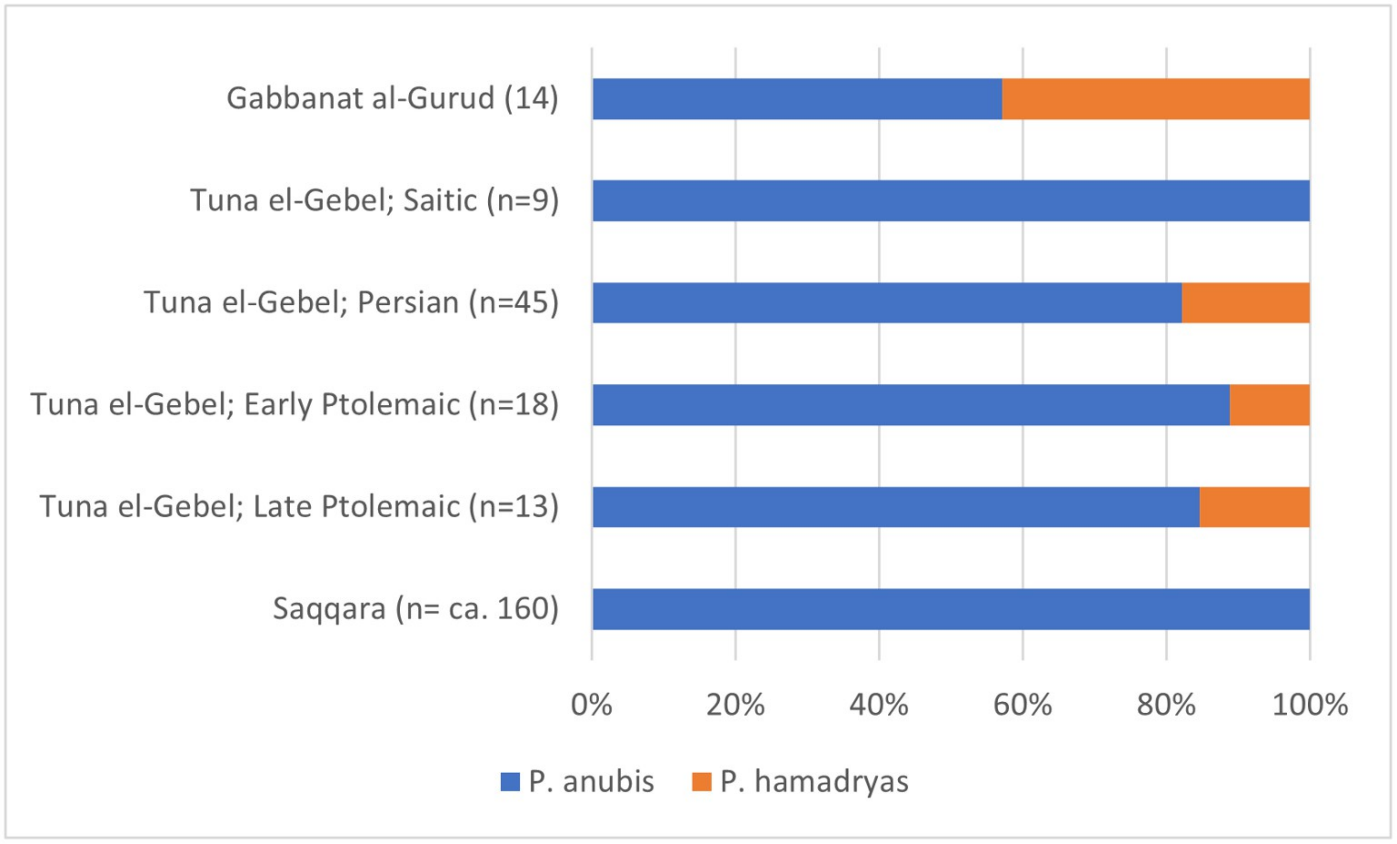
Proportion of anubis and hamadryas baboons at Gabbanat el-Qurud, Saqqara and the four periods at Tuna el-Gebel.

In Gabanat el-Qurud, 14 of the 36 individuals could be identified to species, showing that there was a preponderance of 8 anubis against 6 hamadryas baboons (Fig 28). In the assemblage from Saqqara, dated between about 400 and 30 BC, anubis was the only baboon species believed to be represented, but the skull of a baboon, labelled as “possibly *Papio cynocephalus*”, is also depicted [16]. Judging from the angle between the vertical plane across the orbitae and the horizontal plane along the palatal bone, this specimen seems to pertain to *P*. *hamadryas*, though. A reanalysis of the material from Saqqara is desirable to check whether this impression is correct and if this assemblage contains additional hamadryas individuals. At Tuna el-Gebel, both species have been identified, but from the publications, we cannot tell the exact criteria that were used to identify the various specimens. Individuals with poorly preserved skulls may well have been identified on the basis of the fossa mandibularis which turned out to be an invalid criterion according to our observations on a large series of modern baboons. For the comparisons below, we retain the published identifications of Tuna el-Gebel, but for this material as well, a reanalysis might be useful. Due to these uncertainties related to species determination, the results discussed below should be assessed with some reservations.

Among the estimated 180 primates at Saqqara, there were also 21 *Macaca sylvanus* (Barbary macaque) and 2 *Chlorocebus aethiops* (green monkey) [16]. The material from Tuna el-Gebel represents a diachronic series [17] that allows documenting the proportions between the various species, the age distribution and the sex ratios over time. In Table 4, we summarize the species occurrences that we could deduce from the tables, figures and descriptions in the latter publication series. In Gallery D, corresponding to the earliest phase, i.e. the Saite Period (from ca. 650 BC), 4 *Chlorocebus aethiops* and 9 *Papio anubis* have been found, besides 22 individuals only identifiable as *Papio* sp.; evidence for *Papio hamadryas* is lacking. In Gallery C, passage C-D, dated to the Persian Period (525–332 BC), a single *Chlorocebus aethiops* and *Erythrocebus patas* (patas monkey) were found. *Papio anubis* (n = 37) is the most common species, but the proportion of *P*. *hamadryas* (n = 8) is higher than in the other galleries. Sixteen baboons could not be identified to species. Passages C-C, C-B and C-A of Gallery C, dating to the Early Ptolemaic Period (until 250 BC), yielded 16 *P*. *anubis*, 2 *P*. *hamadryas*, 35 unidentified baboons, and 3 *C*. *aethiops*. In the Late Ptolemaic Gallery B, dating after 250 BC, there were 11 *P*. *anubis*, 2 *P*. *hamadryas*, 9 *C*. *aethiops* and 1 *Macaca sylvanus*.

**Table 4:** Occurrence of the different primates identified from the various galleries in Tuna el-Gebel (based on [17]).

			*P*. *anubis*	*P*. *hamadryas*	*Papio* sp.	*E*. *patas*	*C*. *aethiops*	*M*. *sylvanus*	
Saite	gallery D		9	-	22	-	4	-	35
Persian	gallery C	passage C-D	37	8	16	1	1	-	63
Early Ptolemaic	gallery C	passage C-C	11	1	22	-	-	-	34
Early Ptolemaic	gallery C	passage C-B	4	1	1	-	1	-	7
Early Ptolemaic	gallery C	passage C-A	1	-	12	-	2	-	15
Late Ptolemaic	gallery B	passage B-A	4	-	6	-	1	-	11
Late Ptolemaic	gallery B	passage B-C	1	-	9	-	-	-	10
Late Ptolemaic	gallery B	passage B-E	6	2	53	-	8	1	70
			73	12	141	1	17	1	245

Information on the chronology kindly provided by D. Kessler.

Looking at the zoogeographical distribution of the primates, inferences can be made about the provenance of the animals and the possible trade routes through which they were brought to the sites. In the case of anubis baboons [60], it can be safely assumed that the animals came from the south, namely the Sudanese Nile Valley. The green monkey, found at both Saqqara and all periods of Tuna el-Gebel, lives in the same region and may have been captured there as well. The same holds true for the single patas monkey found in a Persian period context at Tuna el-Gebel. Transport of anubis baboons, and occasionally green monkeys or patas monkeys, from Sudan towards the north was probably facilitated by the currents of the Nile and was effective already since predynastic times, around 3700 BC, as shown by the finds from Hierakonpolis. Access to hamadryas baboons was clearly a later development, with the earliest evidence coming from the New Kingdom finds in Thebes. They occur in Late Period Gabbanat el-Qurud and Persian period Tuna el-Gebel; in the earliest, Saite phase of Tuna el-Gebel, the species is lacking but the sample size for that period is small. If the absence of hamadryas in the oldest phase of Tuna el-Gebel is not due to sampling bias, then the earlier presence of this species of baboon in Gabbanat el-Qurud is consistent with the rare sources attesting to the use of the ancient roads to the Red Sea and contacts with the land of Punt during the Saite period [61–67]. Hamadryas baboons inhabit arid sub-desert and steppe environments in mountainous areas bordering the Red Sea, from Suakin in the Sudan southwards to Eritrea, Somalia and Ethiopia. They also occur in the southwestern part of the Arabian Peninsula [68]. The provenance of hamadryas baboons has been often discussed and the Land of Punt was mentioned in pharaonic times as the region from which these monkeys were obtained. The exact definition of where Punt was located is still ongoing and both the Horn of Africa as the southern part of the Arabian peninsula have been named [44, 69–75].

## Summary and conclusions

The analysis of the skeletal remains, both cranial and postcranial material, of the mummified baboons from Gabbanat el-Qurud showed that the collections from the Musée des Confluences consist of a minimum of 36 individuals. Radiocarbon dating was attempted on 13 individuals, but only three could be dated and all indicate that they belong to the end of the Third Intermediate Period and the beginning of the Late Period, which is older than suggested by textual evidence and archaeological material that was believed previously to be associated with the mummies. Of the 14 individuals that could be identified to species, 8 belonged to *Papio anubis* and 6 to *Papio hamadryas*. The cranial remains show that all age categories–from infants to adults–are present with a slight predominance of males among the adolescent to adult animals. This, combined with the abundant evidence of metabolic disorders, suggests that a local breeding population was established to fulfill local demand. With the exception of a small number of healthy specimens that likely represent direct imports from the wild, it appears that most animals were born and raised in captivity, suffering from chronic lack of sunlight and imbalanced nutrition. The main pathologies observed were rickets, neurocranial lesions and deformations in the craniofacial region and mandible. However, no traumatic lesions were observed on the bones showing that the animals underwent relatively little direct violent treatment.

Comparison of the pathologies and of the age and sex distribution with similar data from Saqqara and Tuna el-Gebel shows many similarities, indicating that the mode of keeping them was largely similar in space and time. The biggest differences observed concern the occurrence of hamadryas baboons. Unlike the anubis baboons, which were translocated downstream from the Sudanese Nile Valley, the supply of hamadryas baboons was less straightforward as they were traded from further afield—the Horn of Africa or the southern part of the Arabian Peninsula—along the Red Sea and then via desert roads. In Tuna el-Gebel, hamadryas baboons start to be found slightly later than in Gabbanat el-Qurud, namely from the Persian period onwards. Despite the great distance from the source area, hamadryas baboons may have been present at Saqqara as well, but a reanalysis of the material is needed to confirm this.

The baboon collection from the Musée des Confluences still provides scope for further analyses that could provide more details about the keeping conditions and the provenance of the animals. Ongoing work includes dental microwear analysis which, combined with the available carbon and nitrogen stable isotope data, could further refine our knowledge concerning the diet of the baboons. These data will allow comparison with existing information from predynastic Hierakonpolis [76]. A series of molars, sequentially sampled for strontium isotope analysis, is under study to try to distinguish between allochthonous individuals and those animals born locally. As shown by previous work [44], the isotopic signature could also provide information on the animals’ provenance where—in the case of the hamadryas baboons—it is hoped to find out whether the Horn of Africa or rather the southern part of the Arabian Peninsula played a role. Previous ancient DNA research has shown that genetic material is very poorly preserved, but mitochondrial DNA could also help determine provenance [57, 77]. In addition, nuclear DNA could ascertain whether crossbreeding occurred between anubis and hamadryas baboons kept in captivity. This phenomenon is known from populations kept in zoological gardens, but has also been observed in the wild [78].

## Supporting information

S1 Text Description of the pathologies. (PDF)

S1 Table List of modern baboon specimens consulted for comparison. Indicated are the registernumbers of the Royal Belgian Institute of Natural Sciences (RBINS), the species, sex and provenance of the animals.
(PDF)

S2 Table Measurements, in mm, of the long bones of seven baboon skeletons from Gabbanat el-Qurud. Indicated are the MHNL registration numbers, the corresponding numbers of Lortet and Gaillard [19], the species name and the sex. Measuring distances and their abbreviations are as in von den Driesch [36].The skeletons MHNL 51000172, MHNL 51000173 and MHNL 51000325 each have measurements that are coherent for one individual. As skeleton MHNL 51000328B is from a rachitic individual, measurements of the greatest length of humerus and femur are not provided. The three other skeletons are clearly reconstructed from more than one individual, but for completeness, we also give the measurements of these bones. In the case of skeleton MHNL 51000170, the dimensions of the arms show that bones from at least one additional individual were used to compose a more or less complete skeleton. Specimen MHNL 51000171, is partly composed of bones from a pathological specimen whose measurements are indicated in bold. MHNL 90002100, a skeleton thought to belong to a baboon found in a jar, is also composed of skeletal elements from more than one individual, as shown by the differences in left and right values for several bones. The distal epiphysis of the left radius has not yet fused, unlike all other long bones. This means that this skeleton consists of at least three individuals.
(PDF)

S3 Table Measurements, in mm, of the baboon skulls and associated mandibles from Gabbanat el-Qurud. Indicated are the MHNL registration numbers, the corresponding numbers of Lortet and Gaillard [19], the species name, the sex, the last erupted tooth and the attrition stage of the dentition. Measuring distances, including the numbers assigned to them, are those of von den Driesch et al. [17]. The only additional measuring distance, defined by us, is indicated with an asterisk. Measurements in brackets are approximate. The attrition stages are according to Reed [33, 34]. NA: not available.
(PDF)

S4 Table Measurements, in mm, of baboon mandibles from Gabbanat el-Qurud that could not be associated with a skull. Indicated are the MHNL registration numbers, the species name, the sex, the last erupted tooth and the attrition stage of the dentition. Measuring distances, including the numbers assigned to them, are those of von den Driesch et al. [17]. Additional measuring distances defined by us are indicated with an asterisk. Measurements in brackets are approximate. The attrition stages are according to Reed [33, 34].
(PDF)

S5 Table Measurements, in mm, of isolated long bones (humerus, radius, ulna, femur and tibia) of baboon (*Papio* sp.) from Gabbanat el-Qurud. Indicated are the MHNL registration numbers and, in the case of the tibiae, the numbers assigned by us to the elements sampled for radiocarbon dating. Measuring distances and their abbreviations are as in von den Driesch [36].
(PDF)
