# Supplementary material for: Palaeopathological and demographic data reveal conditions of keeping of the ancient baboons at Gabbanat el-Qurud (Thebes, Egypt)
Source: PLoS One. 2023 Dec 6;18(12):e0294934. doi: 10.1371/journal.pone.0294934 (PMC10699651; doi:10.1371/journal.pone.0294934)
Supplement: S1 Text — (PDF) [file pone.0294934.s001.pdf]

S1 Text. Description of the pathologies

Below we describe the baboon remains that were available in the Lyon collections, starting with the skulls and mandibles, then the more or less complete skeletons, and finally the isolated skeletal elements showing pathological conditions but that could not be linked to a particular individual. Only descriptions are given in this section, the aetiology of the observed pathologies is covered in the main text of the article. For the sake of completeness we also depict some of the rather well preserved few cranial remains that show no pathologies.

Contents

1. Skulls ... 4

Skull MHNL 51000170 *Papio hamadryas* female; Lortet & Gaillard # 3 ... 5

Skull MHNL 51000171 *Papio hamadryas* male; not in Lortet & Gaillard ... 8

Skull MHNL 51000172 *Papio hamadryas* female; Lortet & Gaillard # 1 ... 12

Skull MHNL 51000173 *Papio anubis* male; Lortet & Gaillard # 8 ... 15

Skull MHNL 51000174 *Papio hamadryas* male; not in Lortet & Gaillard ... 19

Skull MHNL 51000175 *Papio anubis* female; Lortet & Gaillard # 5 ... 24

Skull MHNL 51000176 *Papio anubis* female; Lortet & Gaillard # 9 ... 28

Skull MHNL 51000177 *Papio anubis* female; Lortet & Gaillard # 10 ... 32

Skull MHNL 51000178 *Papio anubis* female; Lortet & Gaillard # 6 ... 35

Skull MHNL 51000179 *Papio hamadryas* female; Lortet & Gaillard # 2 ... 38

Skull MHNL 51000180 *Papio* sp. male juvenile; Lortet & Gaillard # 12 ... 43

Skull MHNL 51000181 *Papio* sp.; (Lortet & Gaillard 1909: 7) ... 45

Skull MHNL 51000182 *Papio anubis* male; not in Lortet & Gaillard ... 50

Skull MHNL 51000183 *Papio hamadryas* male; Lortet & Gaillard # 7 ... 52

Skull MHNL 51000318 *Papio* sp.; not in Lortet & Gaillard ... 55

Skull MHNL 51000319 *Papio* sp. male; not in Lortet & Gaillard ... 58

Skull MHNL 51000320c *Papio anubis* female; Lortet & Gaillard # 4 ... 59

Skull MHNL 51000321 *Papio* sp.; not in Lortet & Gaillard ... 64

Skull MHNL 51000323A *Papio* sp. male; not in Lortet & Gaillard ... 66

Skull MHNL 90002100A *Papio anubis* female; Lortet & Gaillard # 11 ... 67

Mandible MHNL 51000315 + 504 *Papio* sp. male; not in Lortet & Gaillard ... 69

2. Skeletons ... 71

Skeleton MHNL 51000172 *Papio hamadryas* female; Lortet & Gaillard skeleton # 1 ... 71

Skeleton MHNL 51000173 *Papio* sp. male; Lortet & Gaillard skeleton # 8 ... 73

Scapulo-humeral joint ... 73

Femur ... 76

Tibia and fibula ... 79

Vertebral column ... 80

Skeleton MHNL 51000325 *Papio* sp. male; not in Lortet & Gaillard ... 82

Skeleton MHNL 51000170 *Papio* sp. male; not in Lortet & Gaillard ... 83

Shoulder girdle and upper arm ... 85

Radius and ulna ... 87

Tibia ... 88

Skeleton MHNL 51000328B *Papio* sp. female; not in Lortet & Gaillard ... 89

Skeleton MHNL 51000324 *Papio* sp.; not in Lortet & Gaillard ... 91

Scapula and humerus 93
Femur 96
Tibia and fibula 98
3. Isolated pathological bones 99
Right radius MHNL 51000324 99
Left humerus MHNL 51000324 99
Two lumbar vertebrae MHNL 51000324 100
Vertebral column MHNL 51000171 101
Right radius & ulna MHNL 51000324 101
Humeri MHNL 51000332 102
Radii and ulnae 102
Femurs and tibiae 103
Hand and foot bones 103
Mandibles 103
Femur MHNL 51000171 103
4. Bibliography 106

1. Skulls

In many of the skulls described below, part of the roots are exposed on the lateral side of the maxillae, well above the alveoli. The apical part of the molar roots is visible, but this is not due to periodontitis, which is a deformation starting at the level of the cemento-enamel junction. We are clearly dealing with a resorption of bone tissue of the thin-walled maxillae. This condition occurs not only in the baboon mummies investigated here, but also in the modern skull collections of the RBINS, in both captive and wild-caught animals. Most skulls and some mandibles have fairly well-developed calculus that has been sampled for oral bacteria analysis (Ottoni et al. 2019). Neither condition is mentioned in the descriptions below.

Skull MHNL 51000170 *Papio hamadryas* female; Lortet & Gaillard # 3

This skull and the corresponding mandible are basically intact. Of the maxillary dentition, the right C-M2 and the left P3-M3 are still in place; in the lower jaw, all the teeth, except for the right I1, are present. The sole deformation that is observed on this skull is on the left parietal bone. The external bone surface is somewhat irregular and thickened across an area of about 1 to 2 cm.

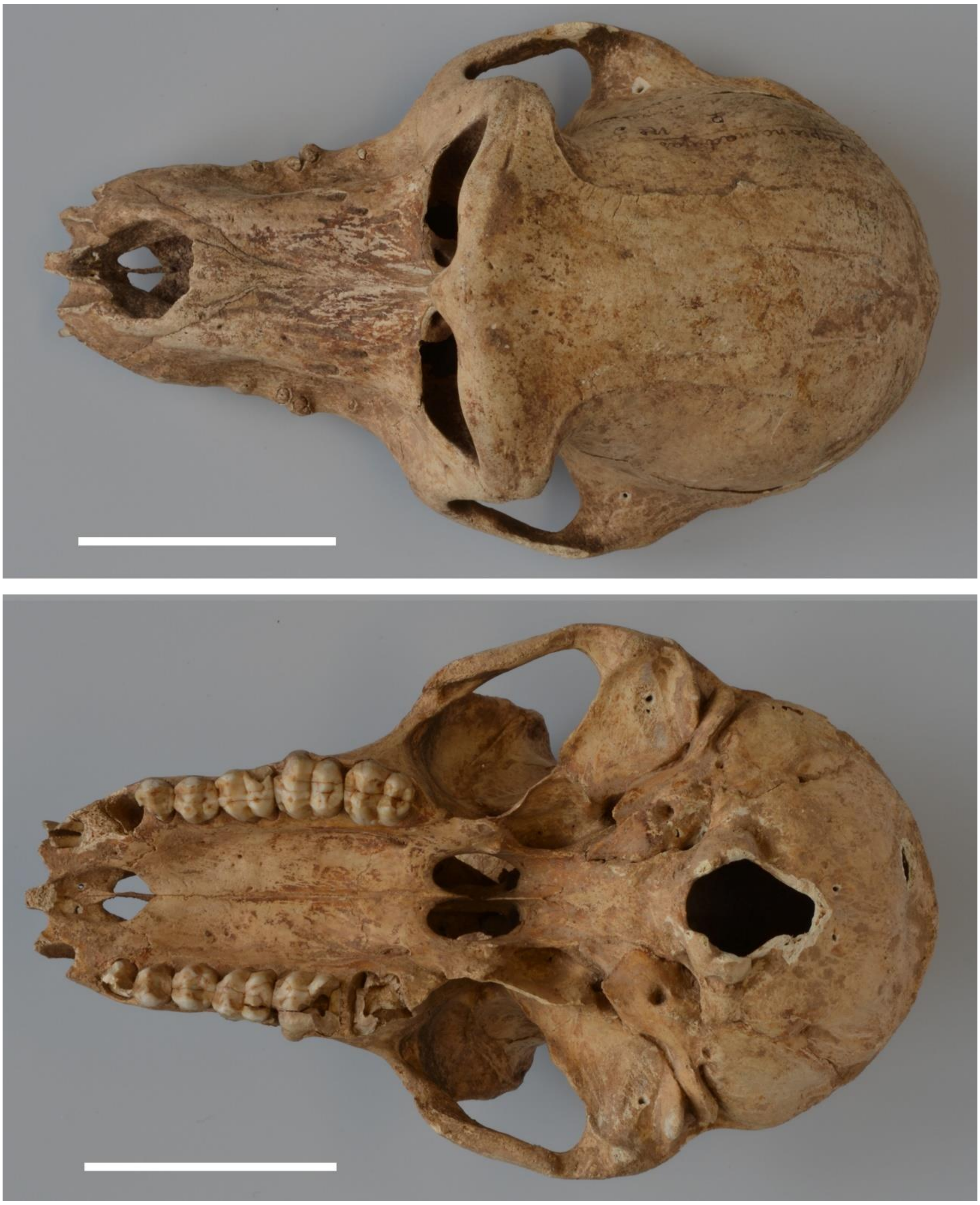

Figure SI-1: Dorsal and ventral view of the skull of *P. hamadryas* female MHNL 51000170. Scale bars are 5 cm.

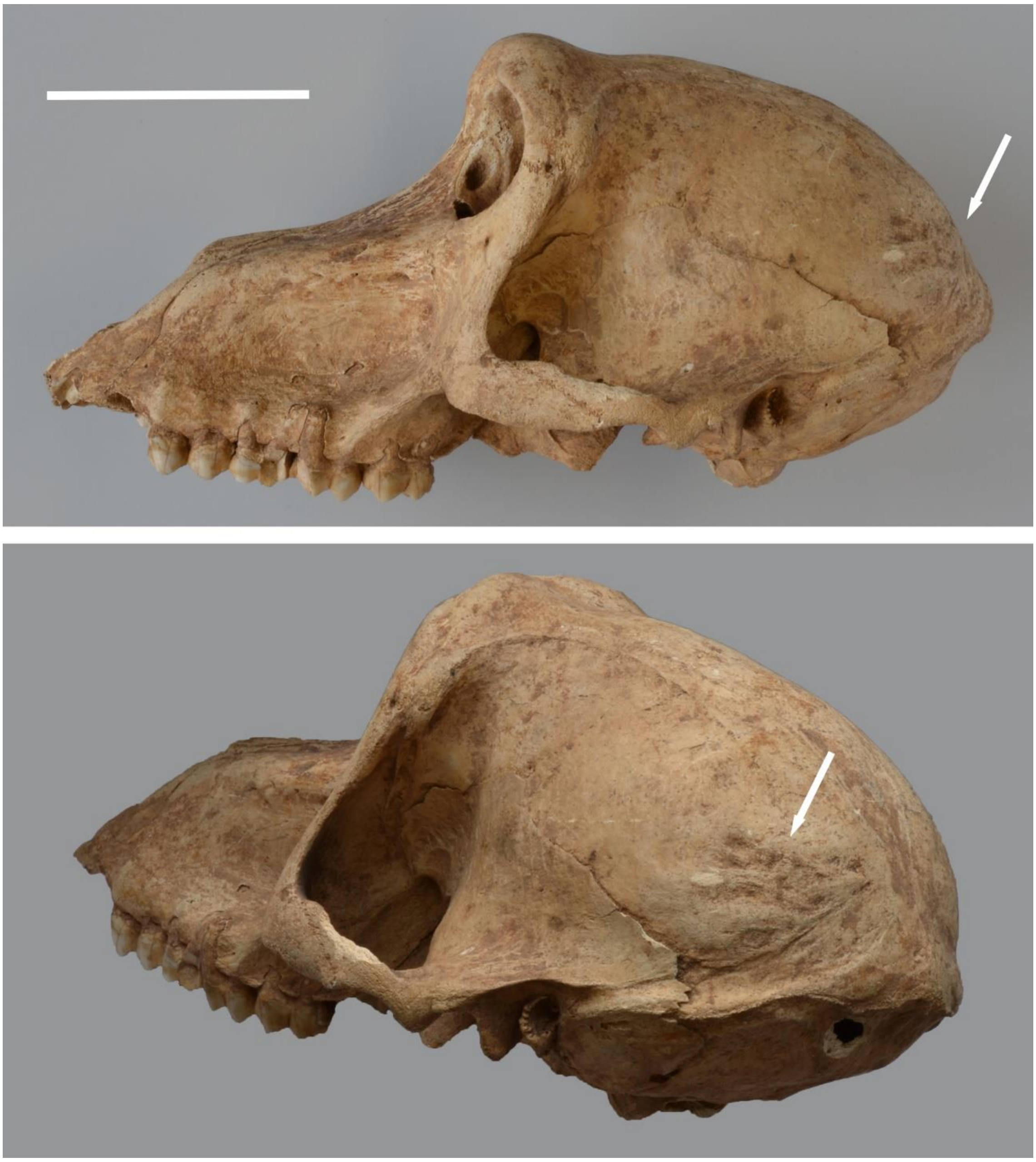

Figure SI-2: Left lateral and posterolateral view of the skull of *P. hamadryas* female MHNL 51000170. The irregular bone surface of the left parietal is indicated with an arrow. Scale bars are 5 cm.

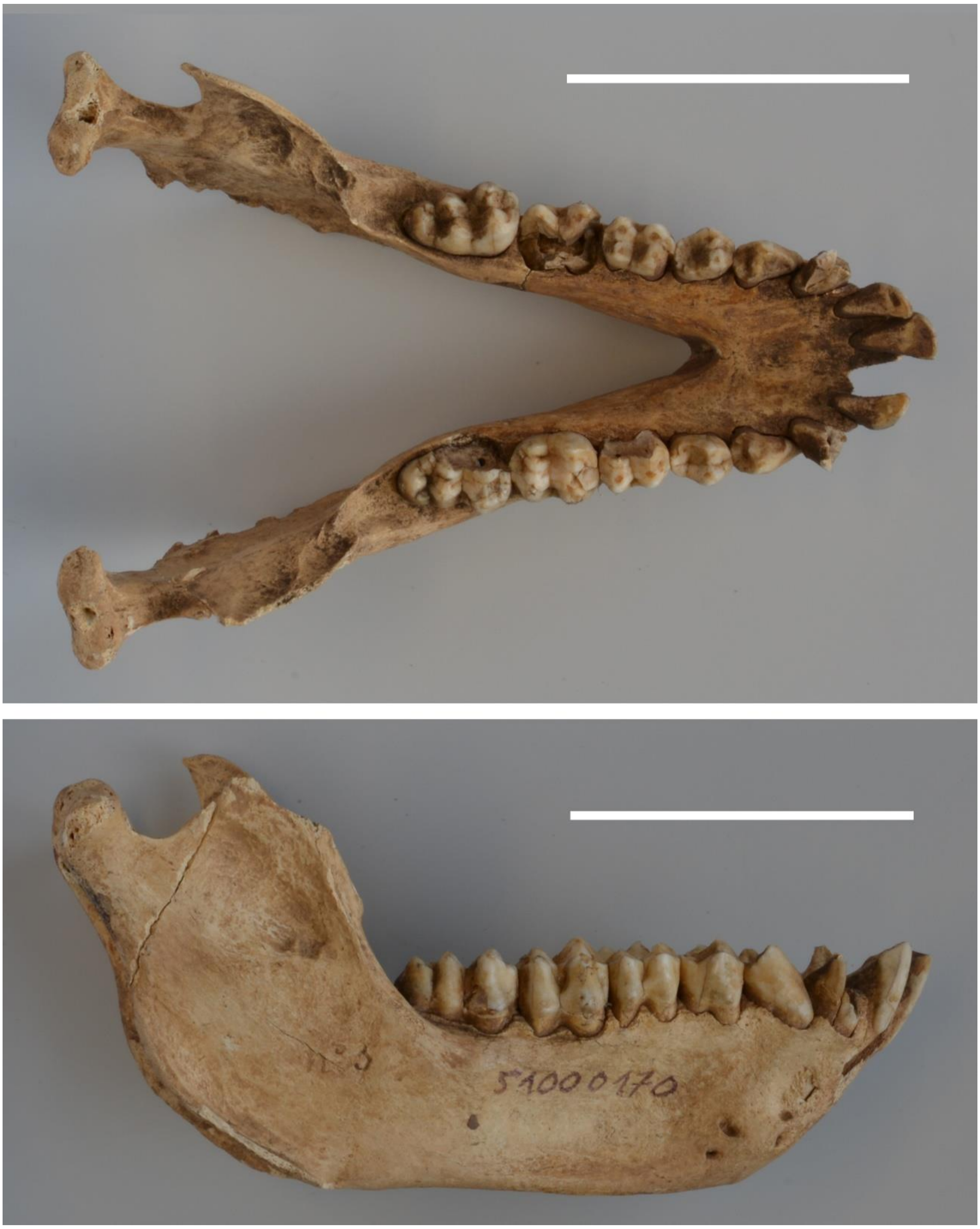

Figure SI-3: Dorsal and right lateral view of mandible of *P. hamadryas* female MHNL 51000170. Scale bar is 5 cm.

Skull MHNL 51000171 *Papio hamadryas* male; not in Lortet & Gaillard

The skull and the mandible of this male individual are available and all the teeth are in situ, albeit some damage to the canines and some of the incisors was noted.The parietals and the lateral parts of the frontals exhibit a slightly rugose, reticulated surface. The upper, medial part of the skull – between the left and right linea temporalis – is smooth. The sagittal crest shows an irregular, sinusoidal course towards the linea nuchalis. There are small left and right lateral extensions at the temporal lines. The surface of the palatine is uneven and shows pitting. This skull and mandible were labeled *Cynocephalus anubis* by Lortet and Gaillard, but the angle between the vertical plane across the orbitae and the horizontal plane along the palatal bone is typical of *Papio hamadryas*.

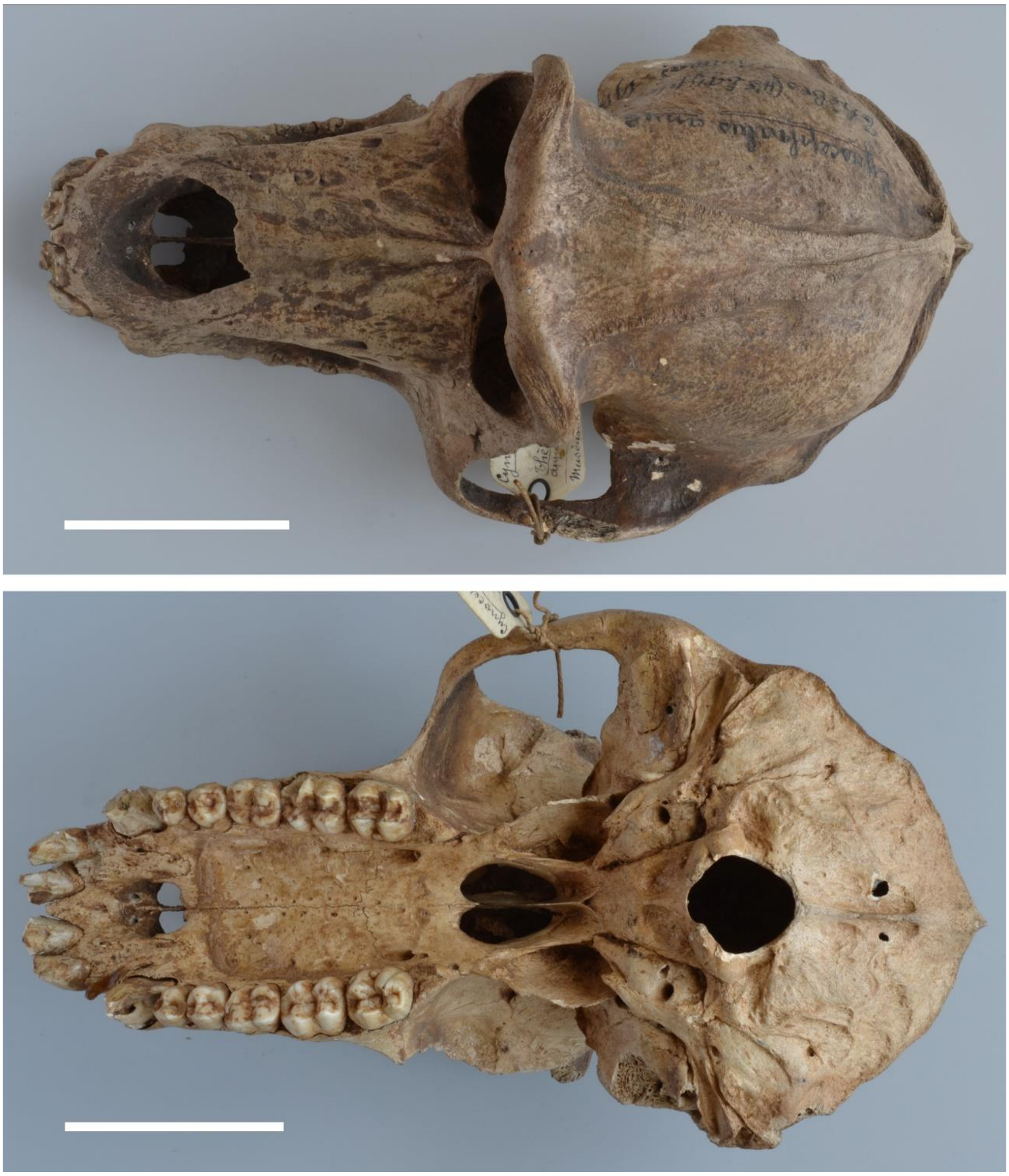

Figure SI-4: Dorsal and ventral view of the skull of *P. hamadryas* male MHNL 51000171. Scale bars are 5 cm.

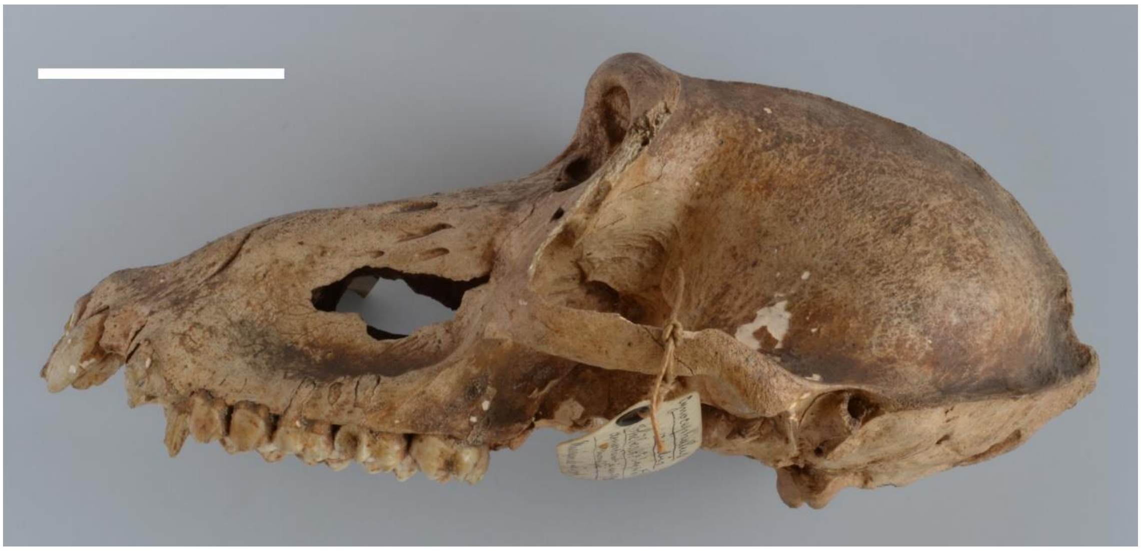

Figure SI-5: Left lateral view of the skull of *P. hamadryas* male MHNL 51000171 with indication of the rugose surface of the parietal. Scale bar is 5 cm.

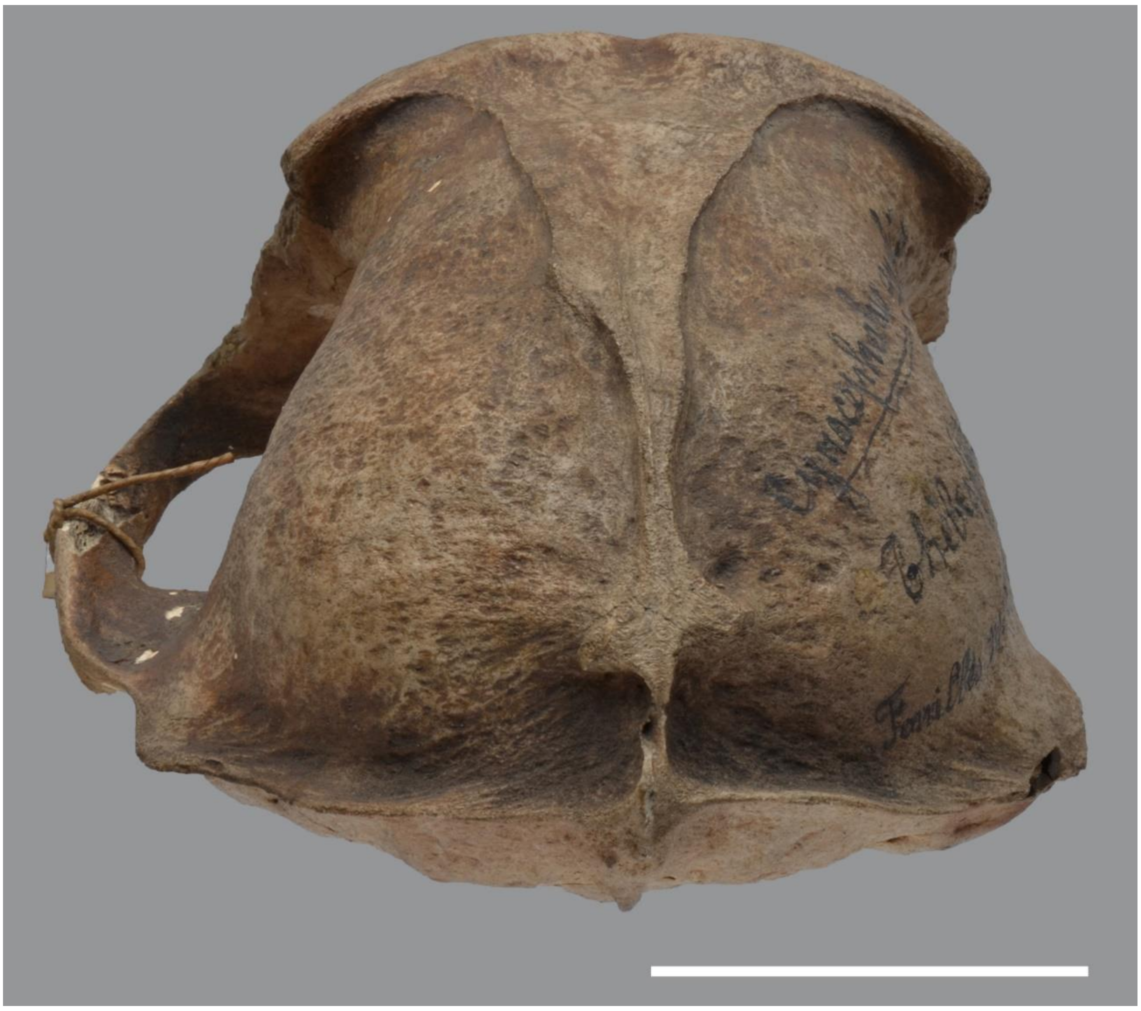

Figure SI-6: Posterior view of the hind skull of *P. hamadryas* male MHNL 51000171 with indication of the rugose surface and the irregular crista sagittalis. Scale bar is 5 cm.

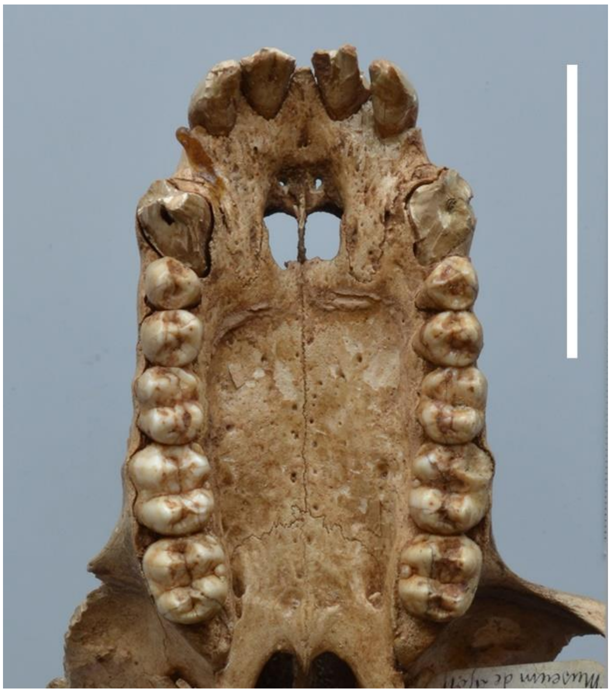

Figure SI-7: Ventral view of the viscerocranium of *P. hamadryas* male MHNL 51000171 showing the pitting and irregular surface of the palatine. Scale bar is 5 cm.

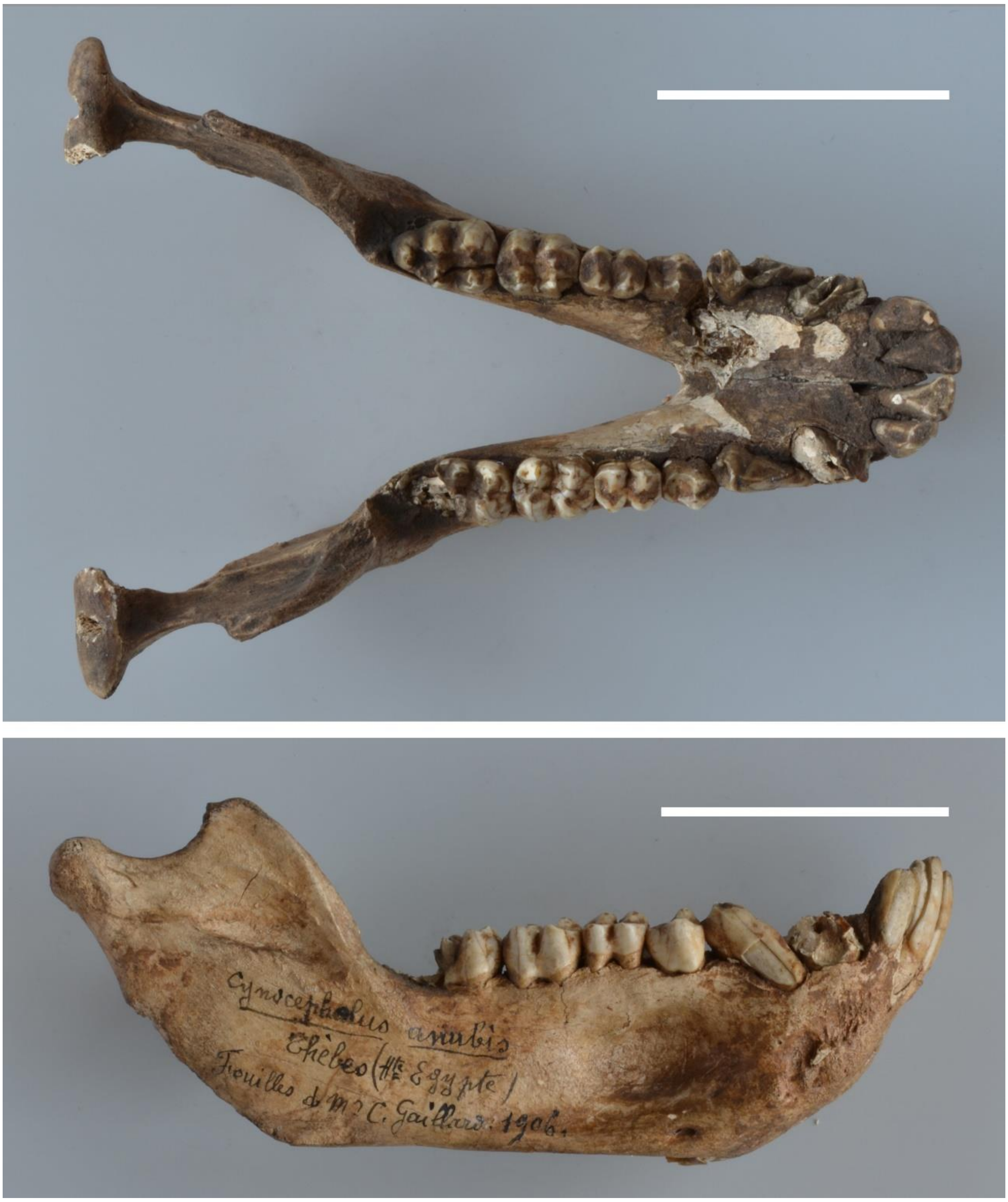

Figure SI-8: Dorsal and right lateral view of the mandible of *P. hamadryas* male MHNL 51000171. Scale bars are 5 cm.

Skull MHNL 51000172 *Papio hamadryas* female; Lortet & Gaillard # 1

The skull and mandible of this individual are very well preserved, but the dentition is incomplete due to post-mortem loss. Maxillary teeth in place include: right I1 and P3-M3; and left I2 and P3-M3. All mandibular teeth are present except for the left I1. This specimen shows some dental pathologies: the left lower M3 has a large caries on its buccal side. Moreover, hypoplasia lines were observed about halfway the crown in the right upper and lower first incisors.

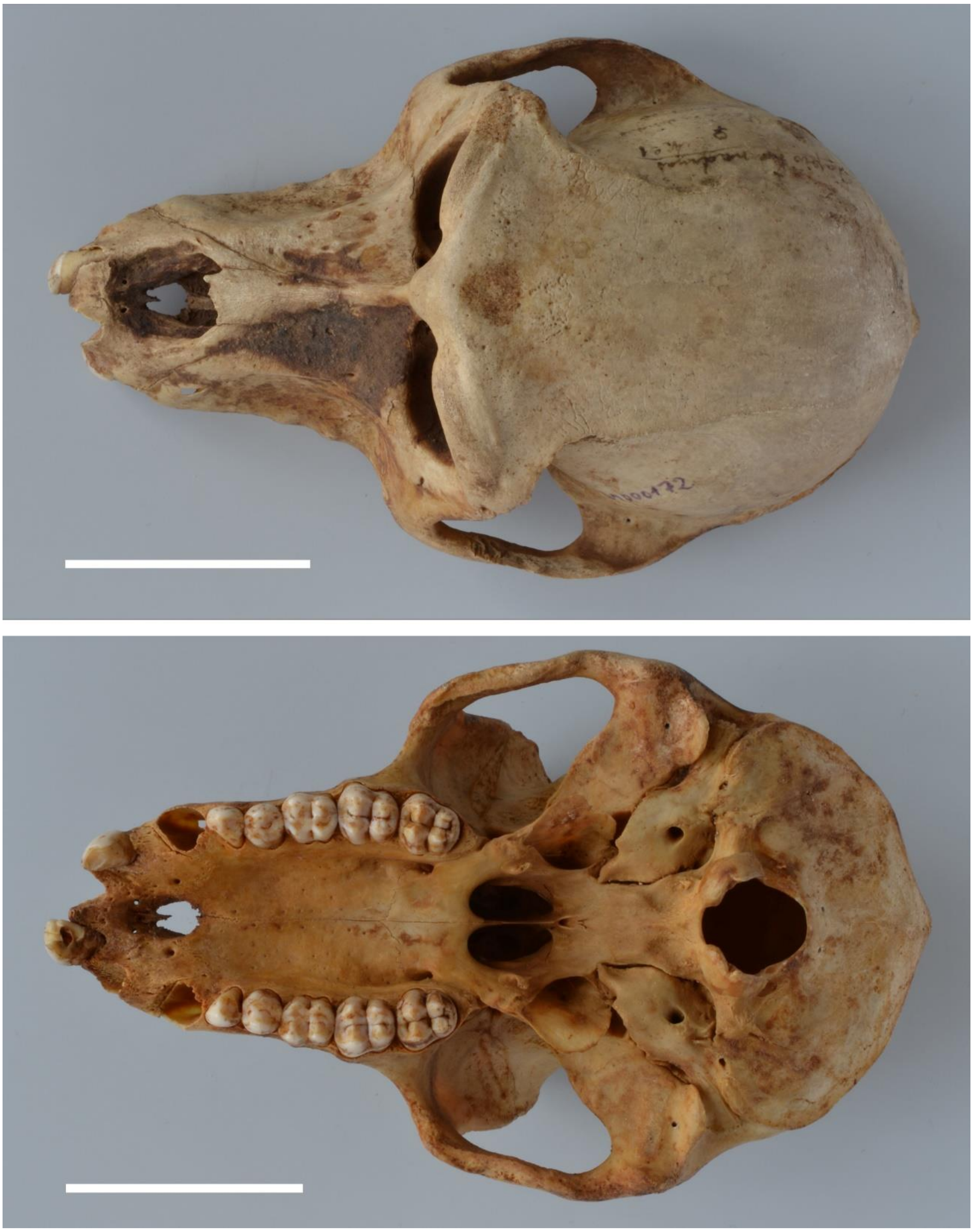

Figure SI-9: Dorsal and ventral view of the skull of *P. hamadryas* female MHNL 51000172. Scale bars are 5 cm.

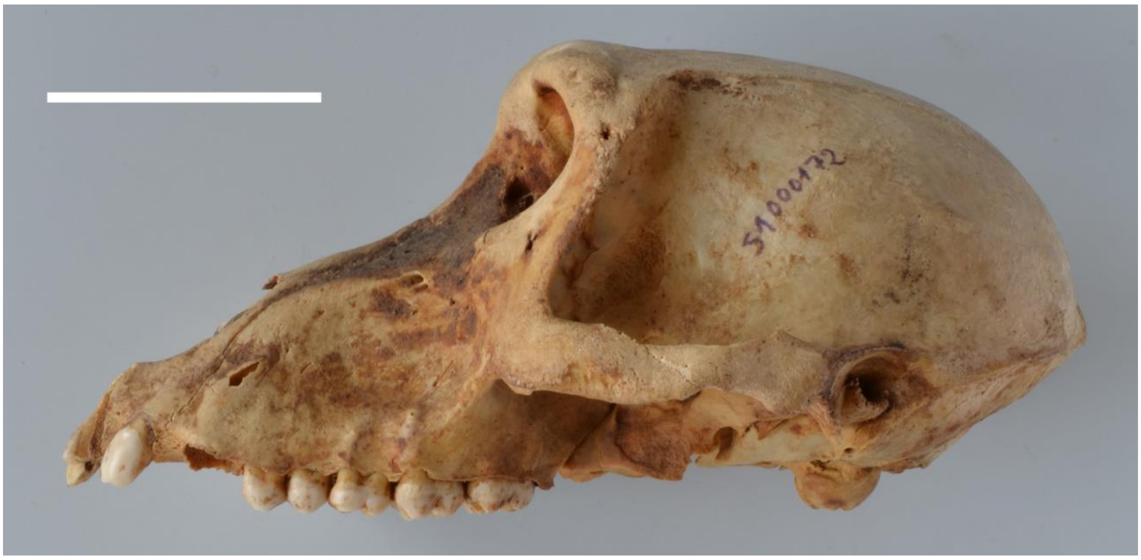

Figure SI-10: Left lateral view of the skull of *P. hamadryas* female MHNL 51000172. Scale bar is 5 cm.

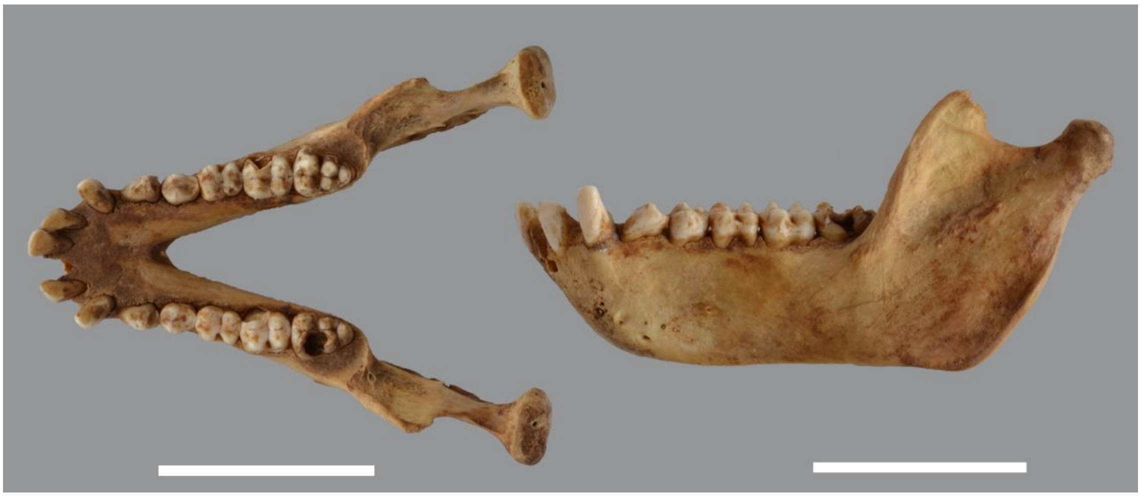

Figure SI-11: Dorsal and left lateral view of the mandible of *P. hamadryas* female MHNL 51000172. Large dental caries is visible in the left M3. Scale bars are 5 cm.

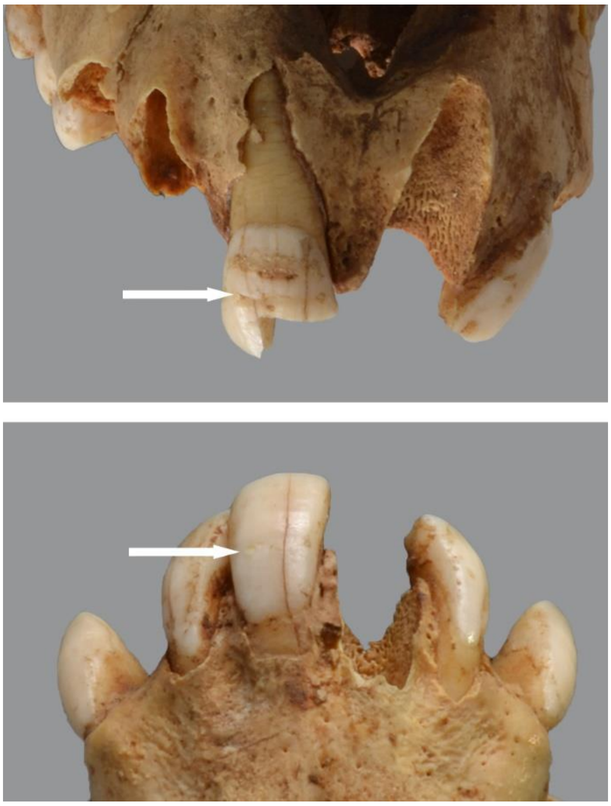

Figure SI-12: Hypoplasia lines in the right upper and lower first incisor of *P. hamadryas* female MHNL 51000172. Above the hypoplasia line of the upper incisor is a linear deposition of dental calculus.

Skull MHNL 51000173 *Papio anubis* male; Lortet & Gaillard # 8

The jugal arches of the skull are severely damaged and the lateral sides of both the left and right maxilla exhibit large holes. Of the maxillary dentition, only the right M2 and M3, and the left P4 (partial) and M1-3 are still in place. The right half of the mandible is well preserved, the left half broke off at some point and was poorly mended. Instead of being flat and smooth, the surface of the palatine is irregular. It also shows some pitting and the buccal margins are somewhat arched upwards. The lateral part of the frontals and the almost entire parietals have a granular, rugose surface. The occipital area has a similar appearance, whereas the surface between the lineae temporales is wide and comparably smooth. Their edges project laterally. The upper nuchal line exhibits an irregular course and the external occipital protuberance is asymmetrical shape.

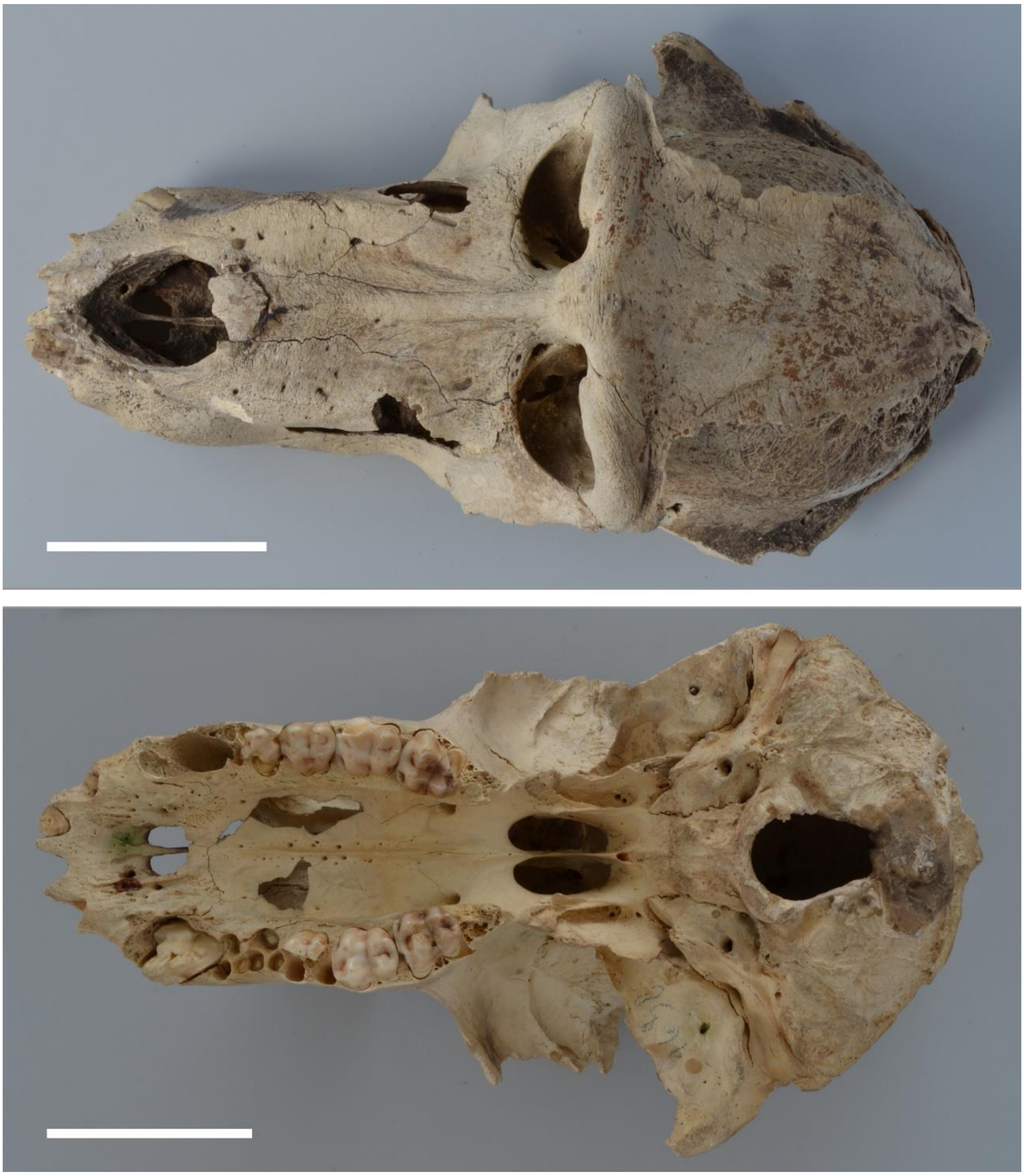

Figure SI-13: Dorsal and ventral view of the skull of of *P. anubis* male MHNL 51000173. Scale bars are 5 cm.

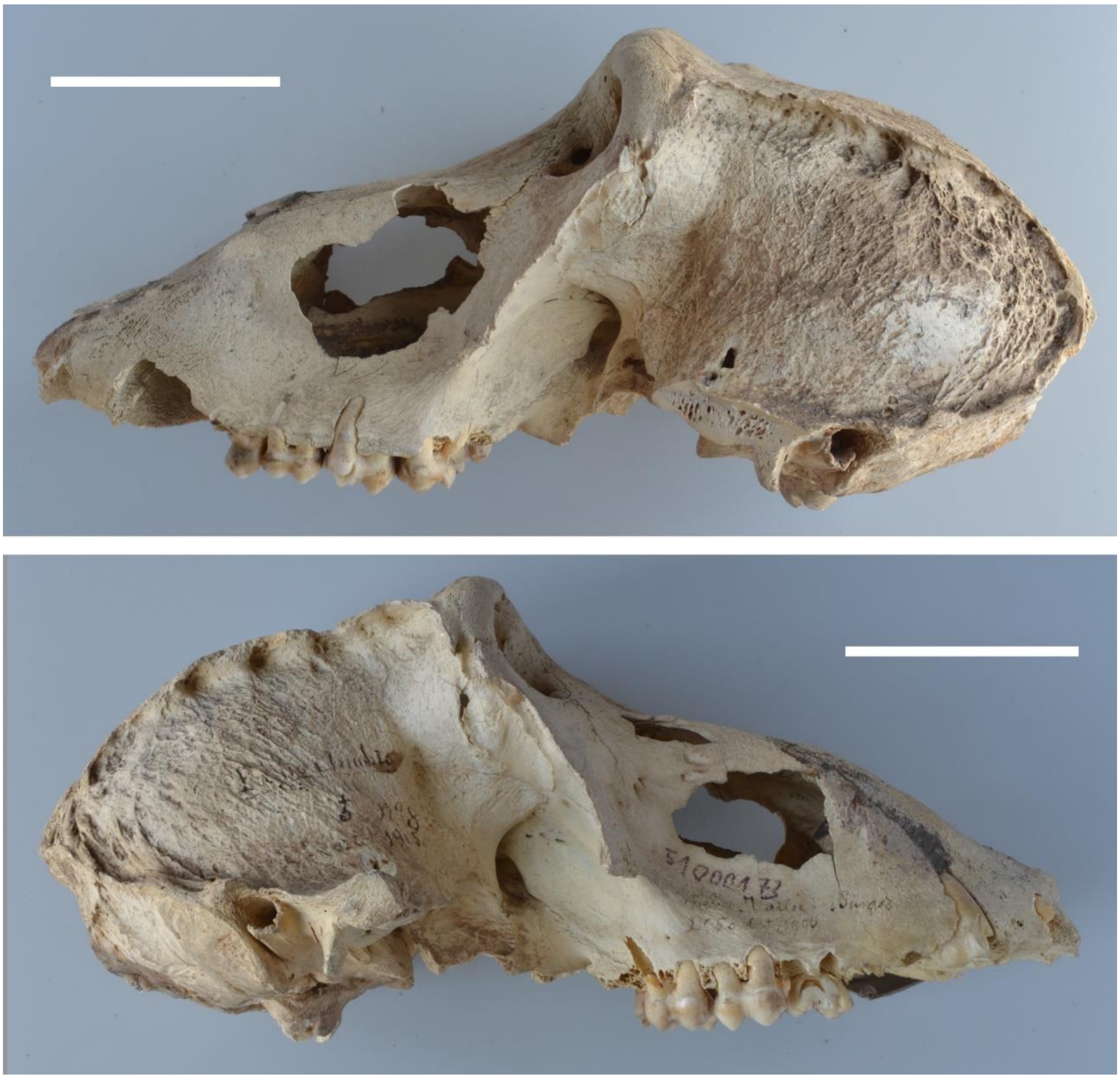

Figure SI-14: Left and right lateral view of the skull of of *P. anubis* male MHNL 51000173 showing the rugose surface of the parietal bones. Scale bars are 5 cm.

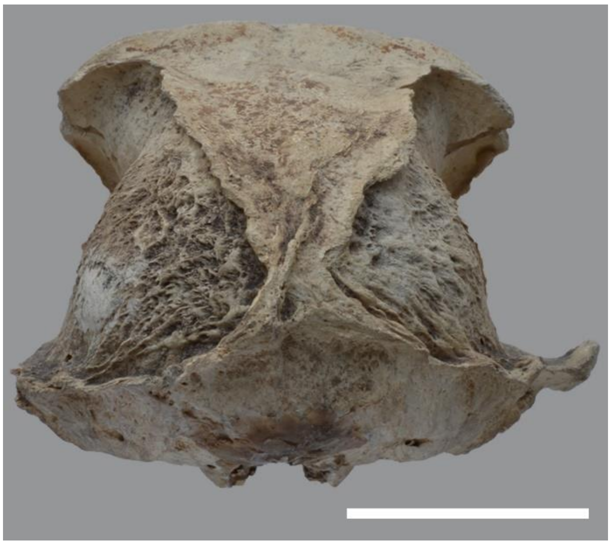

Figure SI-15: Posterior view of the hind skull of *P. anubis* male MHNL 51000173 showing the rugose surface of the parietal bones, the laterally extended lineae temporales, the irregular course of the superior nuchal line, and the asymmetrical shape of the external occipital protuberance. Scale bar is 5 cm.

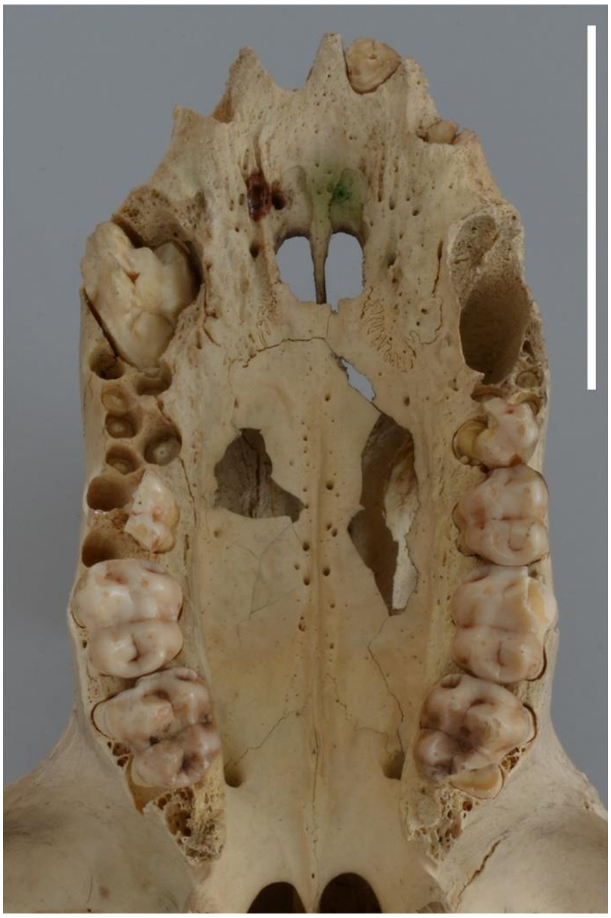

Figure SI-16: Ventral view of the viscerocranium of *P. anubis* male MHNL 51000173. The palatine has an irregular surface and is pitted. The holes are due to post-mortem damage. Scale bar is 5 cm.

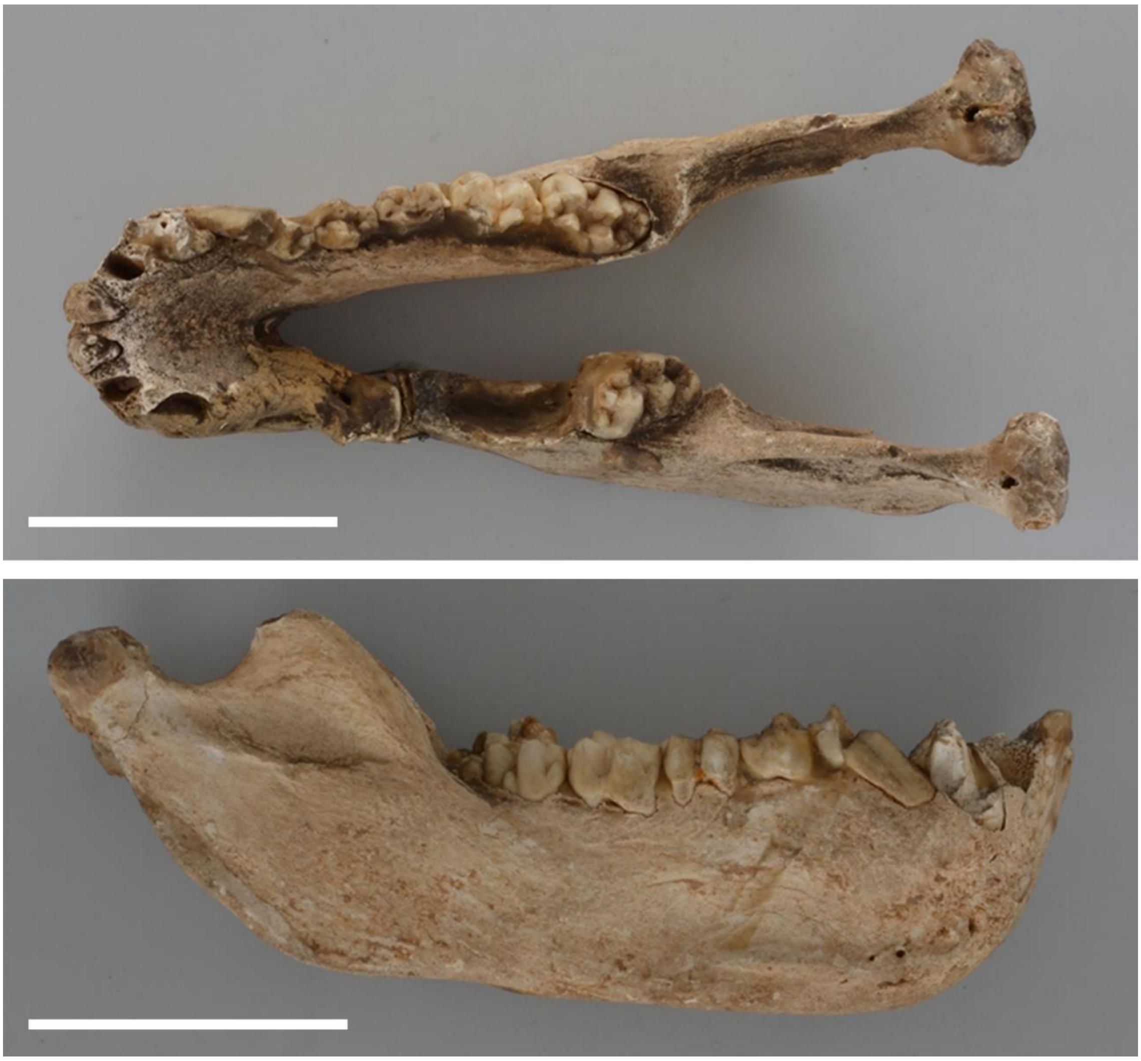

Figure SI-17: Dorsal and right lateral view of the mandible of *P. anubis* male MHNL 51000173. Scale bars are 5 cm.

Skull MHNL 51000174 *Papio hamadryas* male; not in Lortet & Gaillard

The skull of this specimen is incomplete, the jugal arches are missing as well as the left lateral side of the hind skull (mainly the parietal and part of the frontal bones) that was repaired with plaster. All the upper teeth are present, except the incisors that were lost post-mortem. The left mandible is complete and all teeth preserved, except for the I1 and the crown of the canine. Of the right mandible only the symphyseal part with the I2 is preserved. This specimen was not depicted by Lortet and Gaillard who apparently only identified this skull at genus level. The old label indicates “Crâne de Cynocéphale » to which, instead of *Papio* sp., papio papio cynocephalus (*sic*) was added together with the new register number 51000174. We believe this animal can be identified as *Papio hamadryas* on the basis of the angle between the osseous palate and the orbits as well as the relatively short snout (Figure SI-23).

Despite the fact that the neurocranium is damaged, we note that larger sections of the lateral part of the frontal and the parietal bones exhibit a reticulated surface. There is a flat, smooth area behind the orbitals, as in specimen MHNL 51000171, but it is less wide. The lineae temporales converge into a sagittal crest, and in the anterior part they are projecting laterally. The anterior part of the viscerocranium seems deformed: the entire premaxilla has a somewhat rugose surface, both externally and at the level of the palatine. It appears moreover that the viscerocranium as a whole is shortened and that the incisive fossa is asymmetrical. The chin is rugose as well. It is also worth noting that both left and right upper P3 are poorly developed.

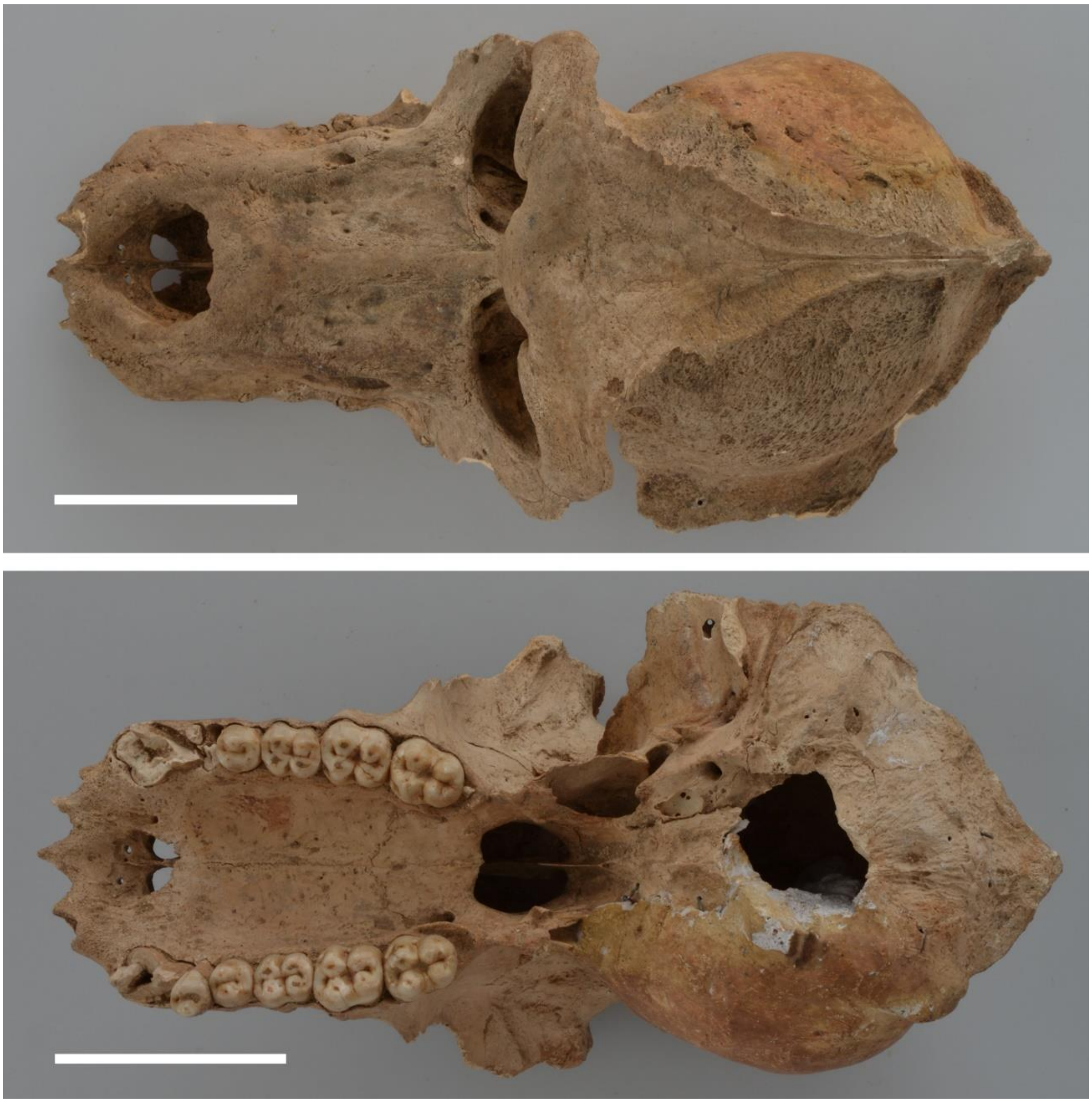

Figure SI-18: Dorsal and ventral view of the skull of *P. hamadryas* male MHNL 51000174. Scale bars are 5 cm.

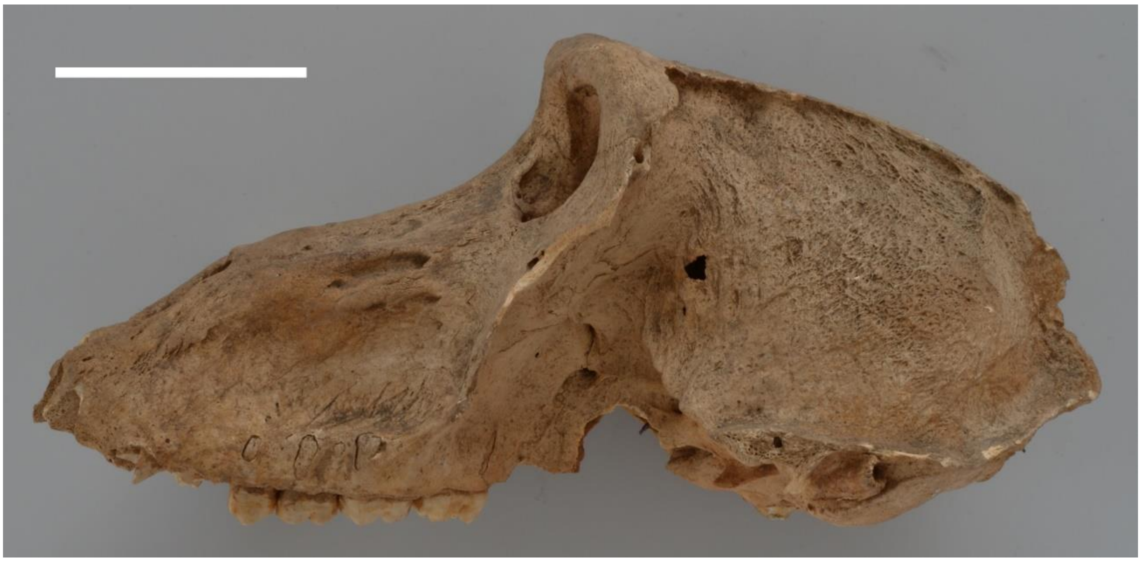

Figure SI-19: Left lateral view of the skull of *P. hamadryas* male MHNL 51000174. Scale bar is 5 cm.

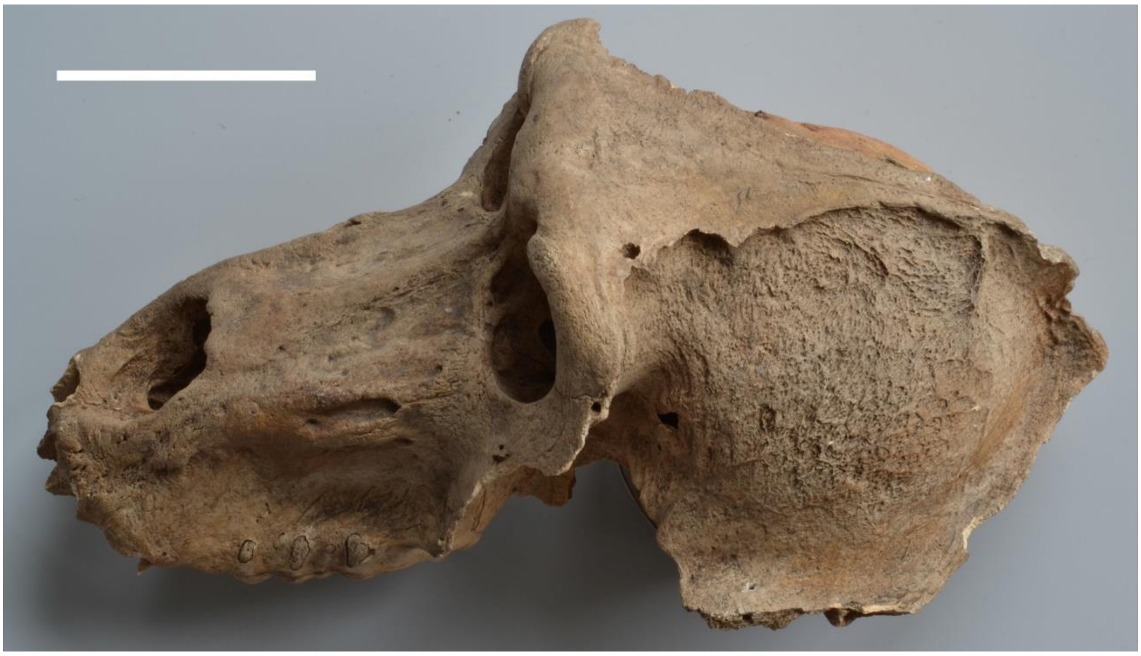

Figure SI-20: Left superolateral view of the skull of *P. hamadryas* male MHNL 51000174 showing the laterally extended lineae temporales and the rugose surface of the left parietal. Scale bar is 5 cm.

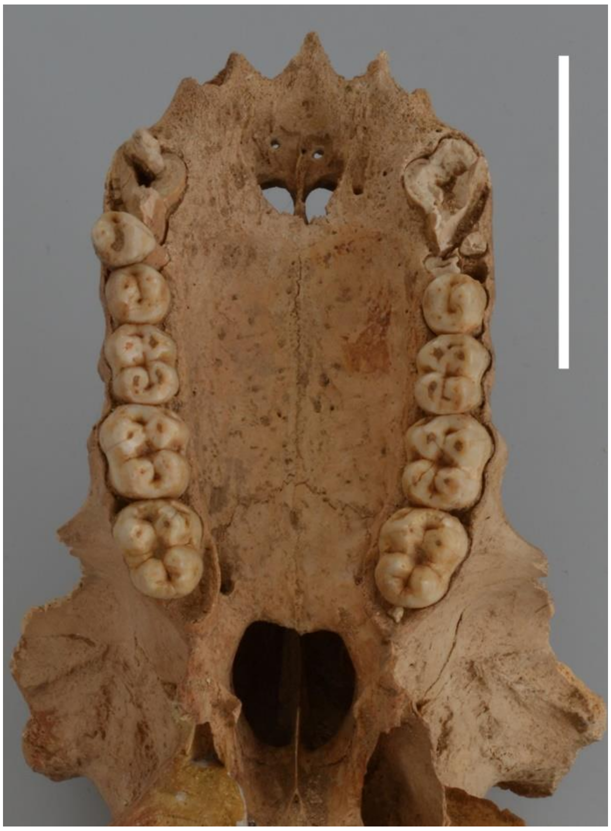

Figure SI-21: Ventral view of the skull of *P. hamadryas* male MHNL 51000174, showing the shortened viscerocranium, the rugose surface of the palatine, as well as the poorly developed P3. Scale bar is 5 cm.

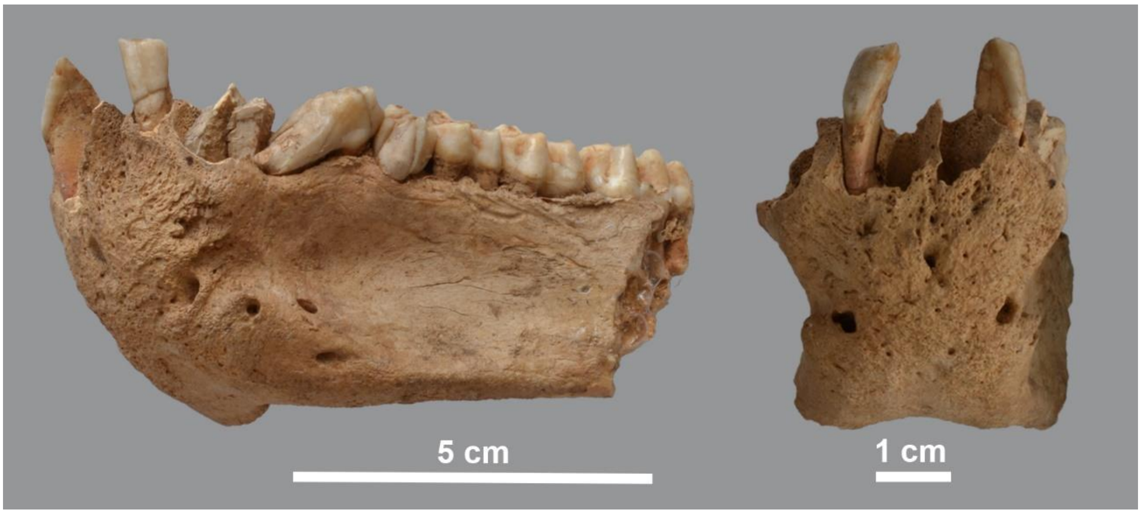

Figure SI-22: Left lateral and anterior view of mandible of *P. hamadryas* male MHNL 51000174. In the anterior view the rugose surface of the chin is visible.

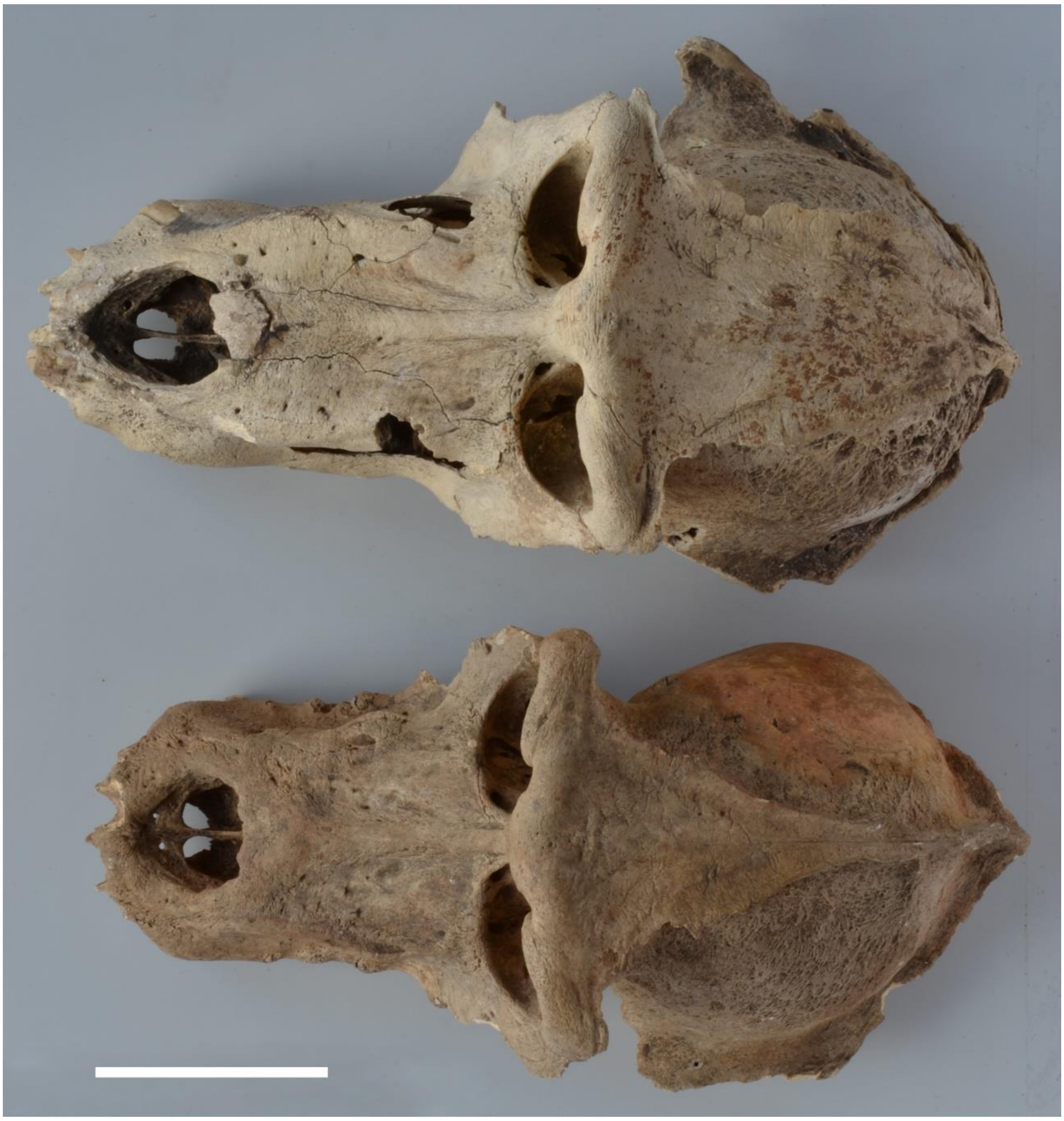

Figure SI-23: Dorsal view of *P. hamadryas* male MHNL 51000174 (below) and *P. anubis* male MHNL 51000173 (above). Scale bars is 5 cm.

Skull MHNL 51000175 *Papio anubis* female; Lortet & Gaillard # 5

The skull is well preserved, except for the damaged premaxillae and left maxilla. The upper right C-M3 are present, in the left upper jaw only the P4, M2 and M3 are preserved. All other maxillary teeth are missing due to post-mortem loss. In the left mandible, of which the vertical ramus is missing, the following teeth are still in place: I1, I2, C (root only), P3, M2 and M3; in the quite complete right mandible, the I1, I2, C, P3 and M3 are present.

The palatine shows pitting. The whole occipital region has a very striking cribriform aspect. Both glenoid fossae also exhibit a spongy aspect, both on the basilar part of the occipital bone and on the maxillae posterior to the third molars. In addition, on the rather completely preserved right half of the mandible, spongy bone occurs on the vertical ramus, near the processus articularis and on the horizontal ramus posterior of the third molar. This third molar shows caries at the level of the posterior cusp. In the right maxilla, a small hole is observed in front of the third premolar. Obviously, the canine is still in its alveolus, since its tip is visible in the crypt.

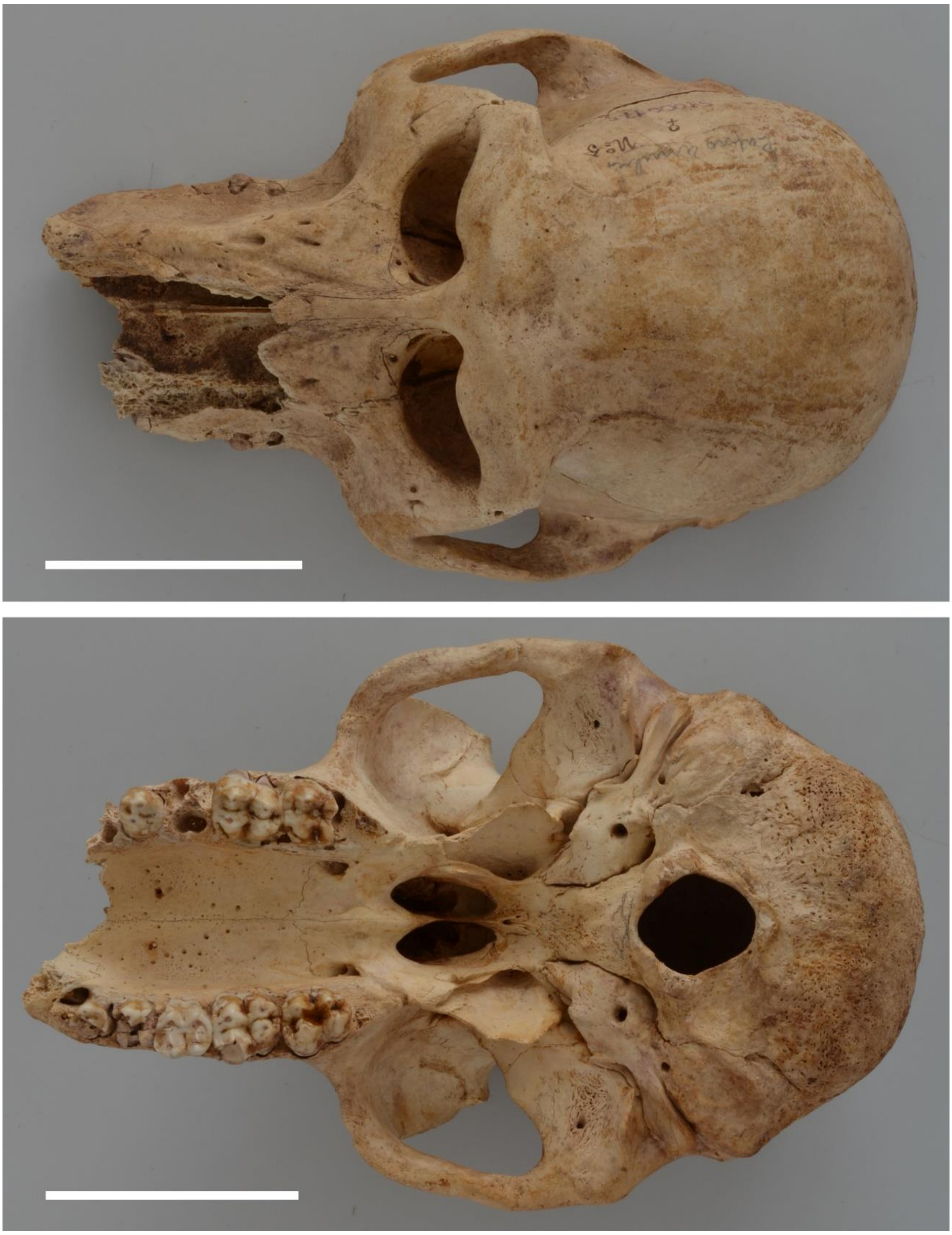

Figure SI-24: Dorsal and ventral view of the skull of *P. anubis* female MHNL 51000175. Scale bars are 5 cm.

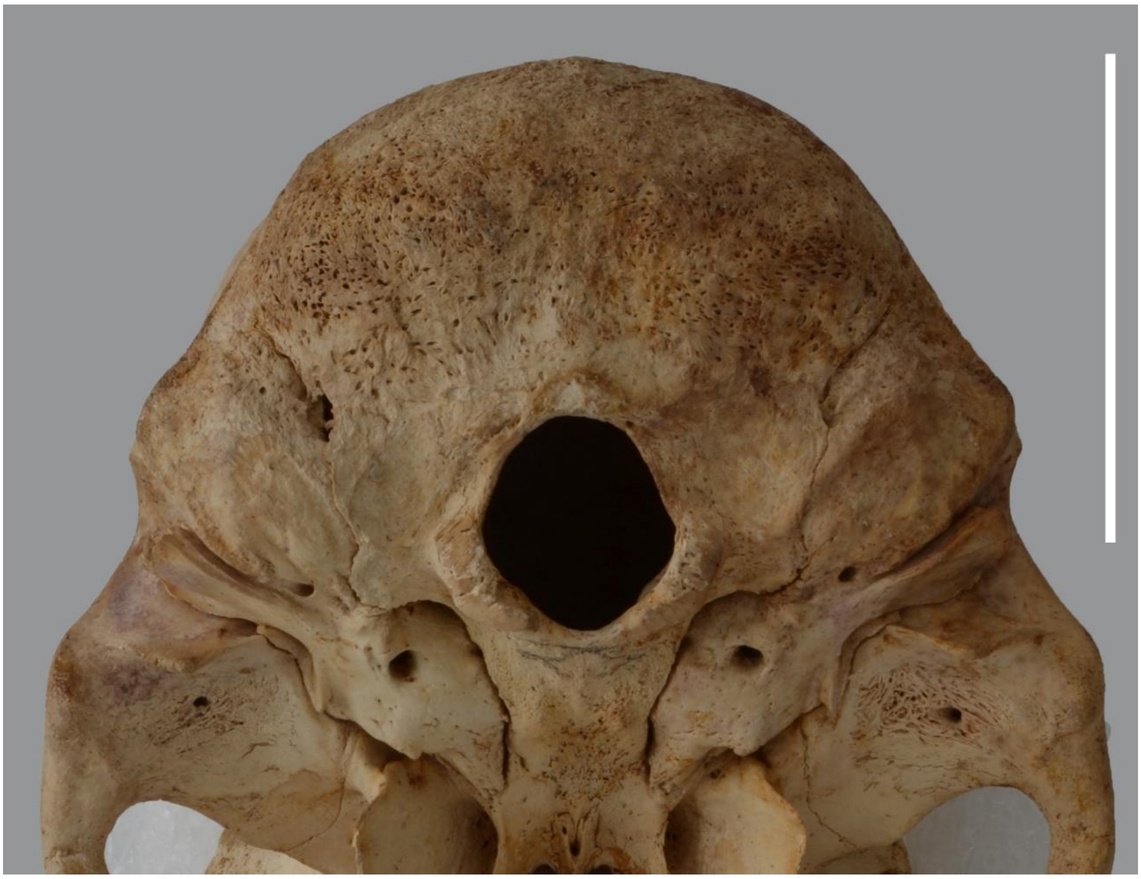

Figure SI-25: Ventral view of hind skull of *P. anubis* female MHNL 51000175, showing the cribriform aspect of the occipital area and the spongy aspect of the glenoid fossae. Scale bar is 5 cm.

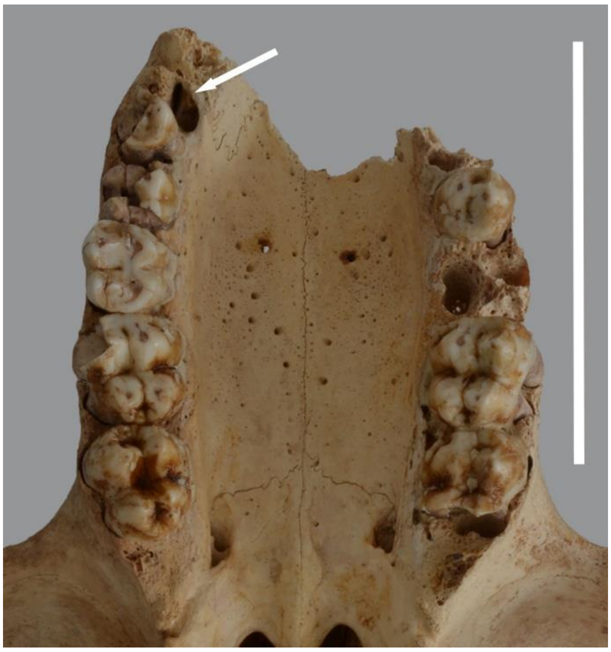

Figure SI-26: Ventral view of the viscerocranium of *P. anubis* female MHNL 51000175 showing the pitted palatine and the crypt with the right canine (arrow). Scale bar is 5 cm.

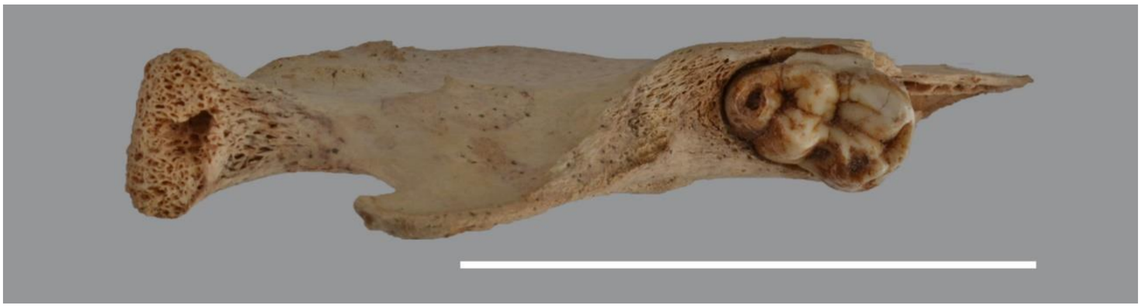

Figure SI-27: Dorsal view of the right mandible of *P. anubis* female MHNL 51000175 showing caries in the posterior lobe of the M3 and the reticulated bone tissue posterior of the M3 and near the processus articularis. Scale bar is 5 cm.

Skull MHNL 51000176 *Papio anubis* female; Lortet & Gaillard # 9

The skull and the mandibles of this individual are intact, except for part of the left jugal arch that was previously sampled for dating purposes. There is also some post-mortem loss of a number of teeth, mainly incisors and canines. The following teeth are still present: in the upper jaws the right P3-M3 and left P4-M3; and in the mandibles the right P3-M3 and the left P3-M3. The third molars are visible, but not yet erupted, indicating that this is a subadult individual.

The skull of this animal shows a metopic suture at the level of the glabella. Furthermore, it is noted that the left and right glenoid fossae are asymmetrical: the left articular surface is much more developed in an oral-aboral direction.

At the buccal side of the right mandible, an elongate lesion of about 2 cm long and 7-8 mm wide is found posterior to the M3. There is no indication at all for an infectious origin of this destructive alteration of the jaw: no sign of caries is observed in the right M3 or any other tooth that could be related to the development of the lesion. Moreover, there is no asymmetry in the mandibular arches, and there is no particular coloration of the area that could be a sign of an ancient hypervascularisation. With no additional parameters that can be evaluated, we conclude that we are dealing with an osteocavitation of unspecific aetiology. In addition, it appears as if the right coronoid process is missing, an anatomical anomaly that was also mentioned by Lortet and Gaillard (1907: 23).

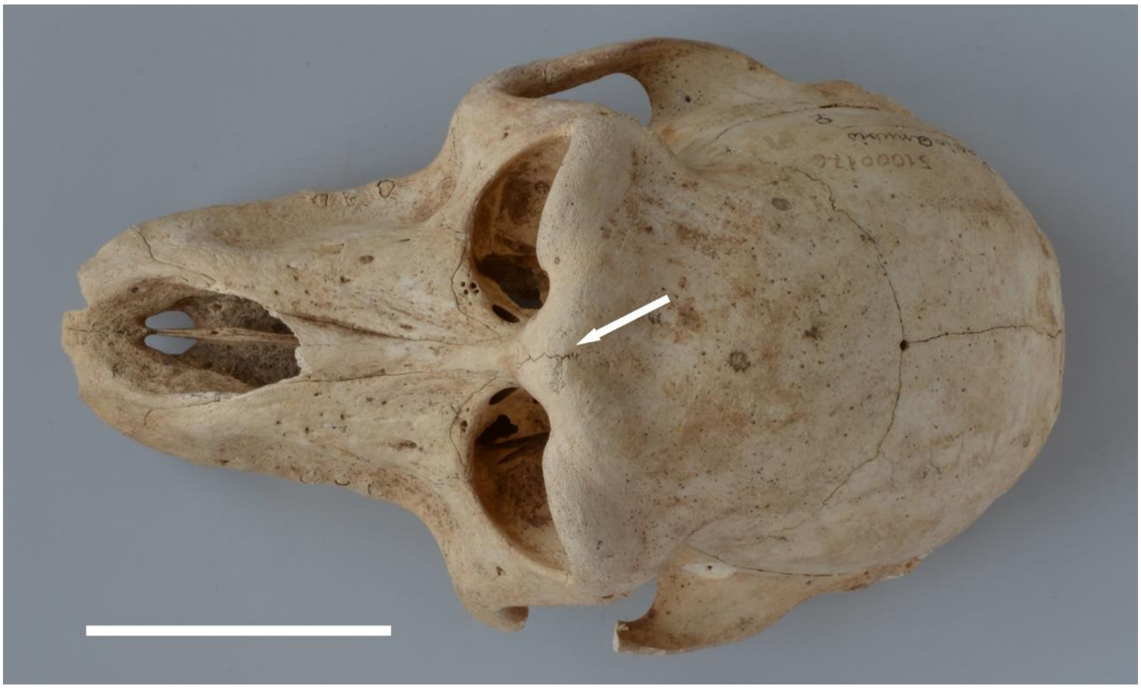

Figure SI-28: Dorsal view of *P. anubis* female MHNL 51000176, showing the metopic suture at the level of the glabella. Scale bar is 5 cm.

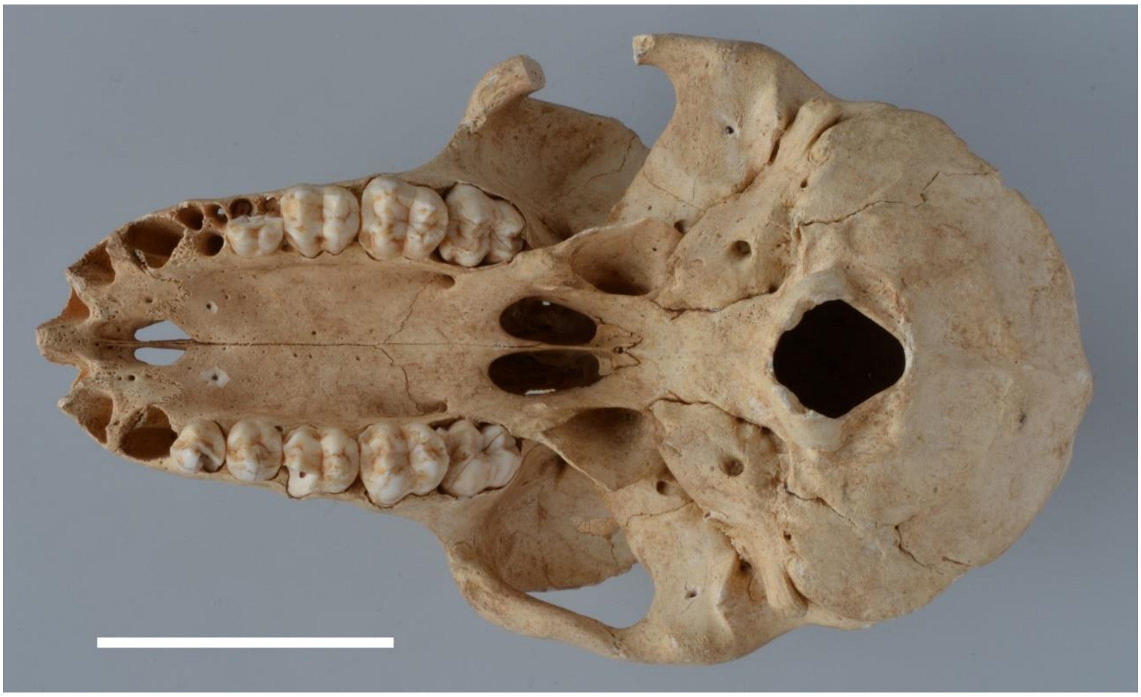

Figure SI-29: Ventral view of skull of *P. anubis* female MHNL 51000176, showing the unequally developed left and right glenoid fossae. Scale bar is 5 cm.

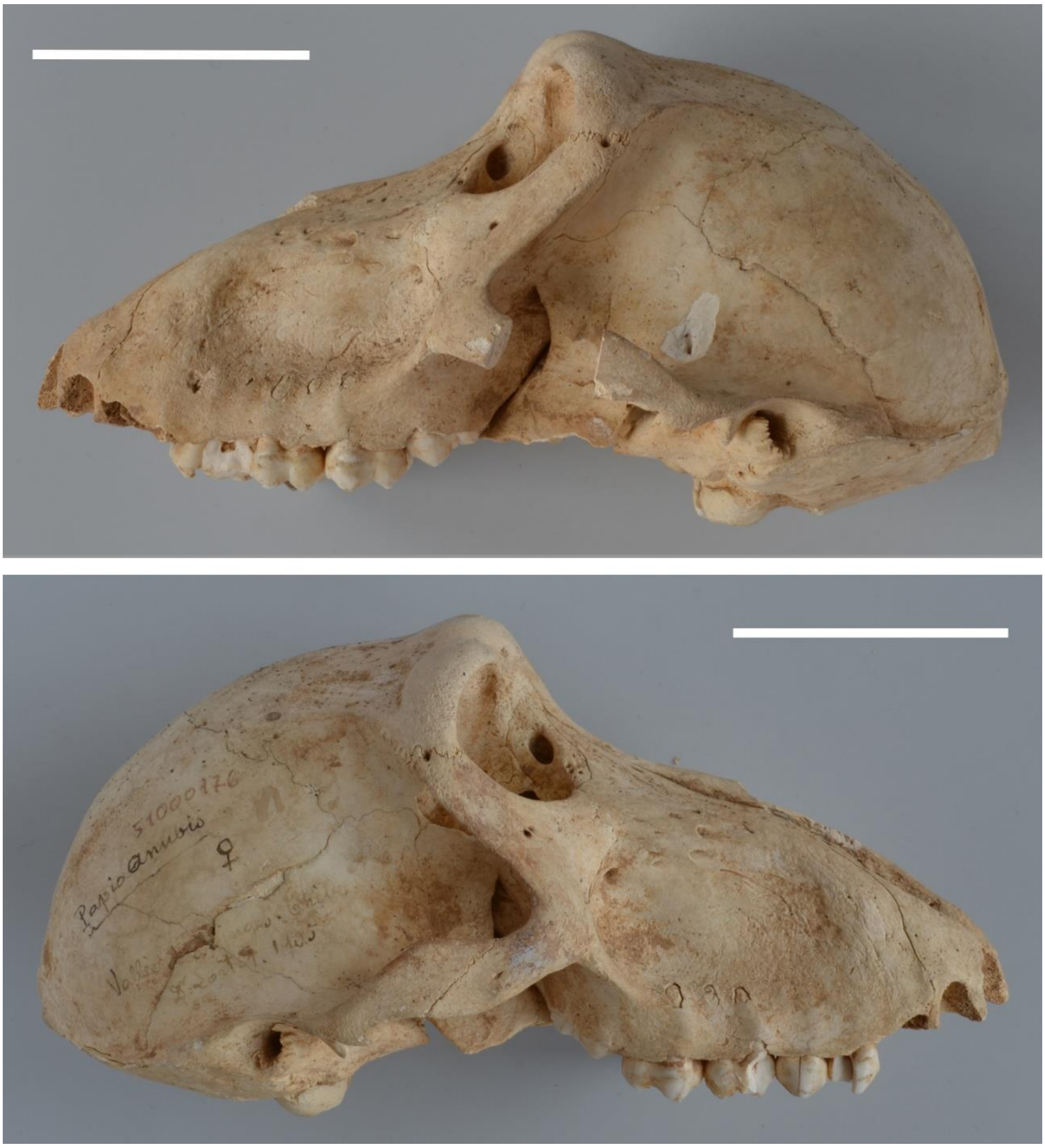

Figure SI-30: Left and right lateral view of the skull of of *P. anubis* female MHNL 51000176. Scale bars are 5 cm.

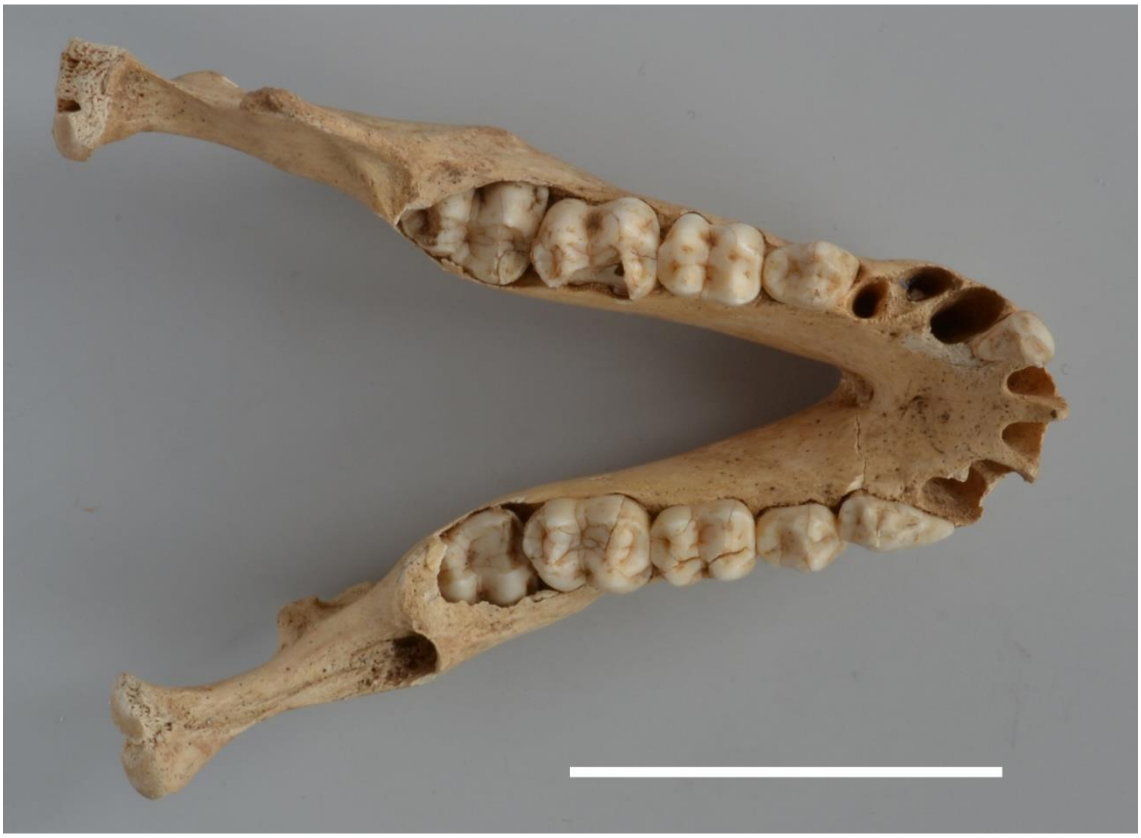

Figure SI-31: Dorsal view of mandible of *P. anubis* female MHNL 51000176, showing a distinctive osteocavitation in the right mandible half, posterior of the M3. Scale bar is 5 cm.

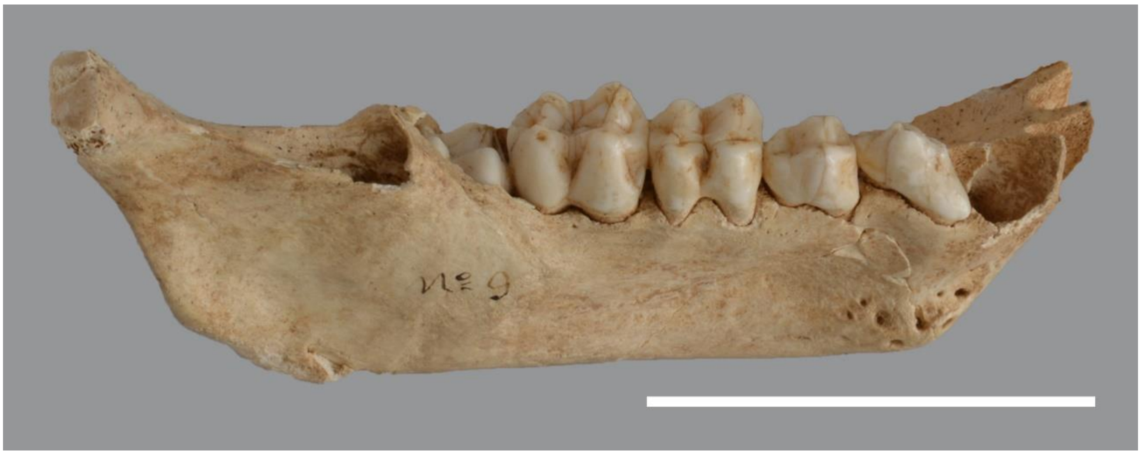

Figure SI-32: Dorso-lateral view of the right mandible half of *P. anubis* female MHNL 51000176 showing the absence of the coronoid process and the distinctive osteocavitation. Scale bar is 5 cm.

Skull MHNL 51000177 *Papio anubis* female; Lortet & Gaillard # 10

The skull of this individual is intact with only some post-mortem loss of teeth. The following teeth are present: right M1 and M2; left P3-M3. Lortet and Gaillard (1907: 24) mention that both mandibles are broken and that the dentition of the right one is almost completely missing. We only found the horizontal ramus of the left mandible with the C, P3, M1 and M2 in place. The palatine of this individual seems pitted and the rostral part is rugose.

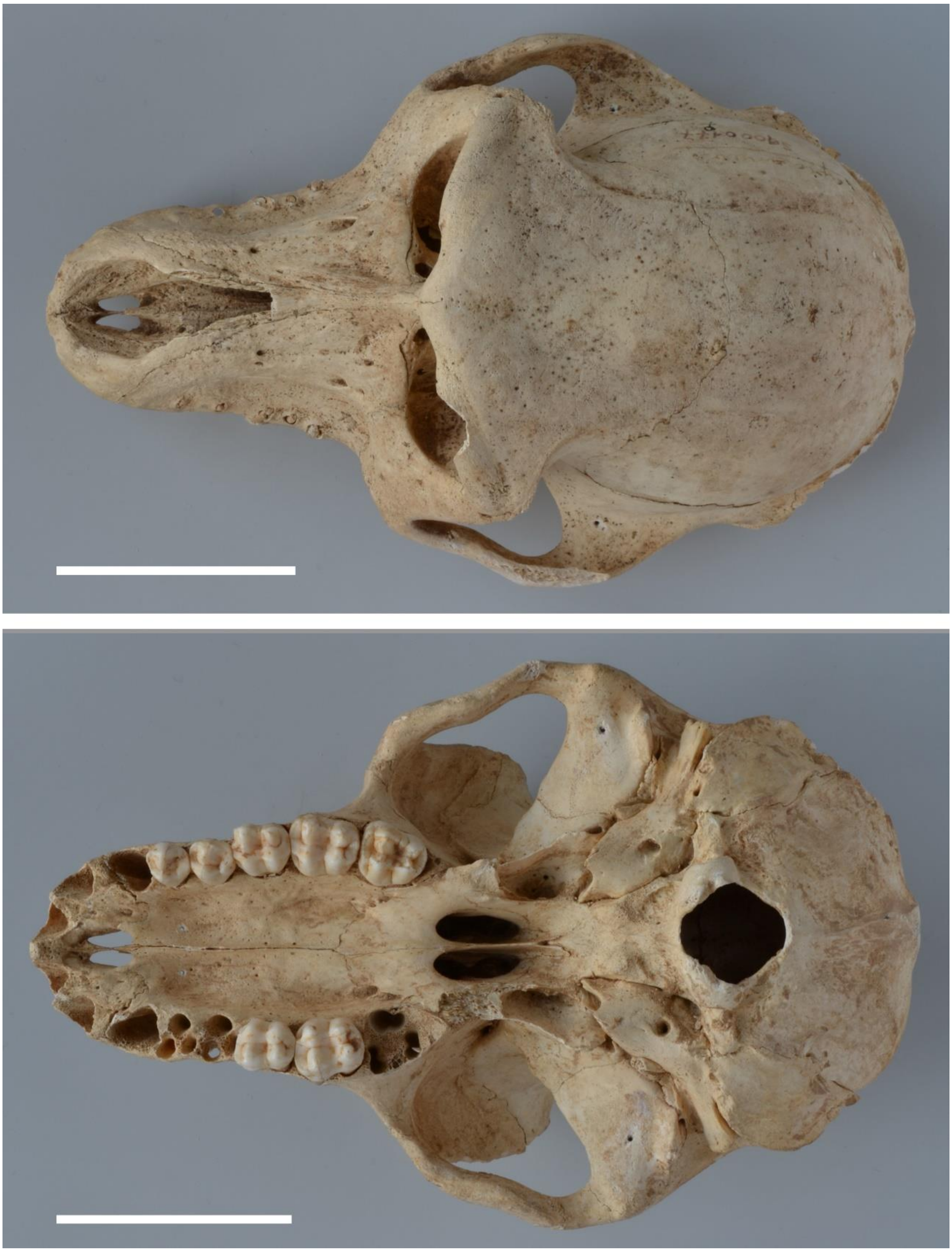

Figure SI-33: Dorsal and ventral view of the skull of *P. anubis* female MHNL 51000177. Scale bars are 5 cm.

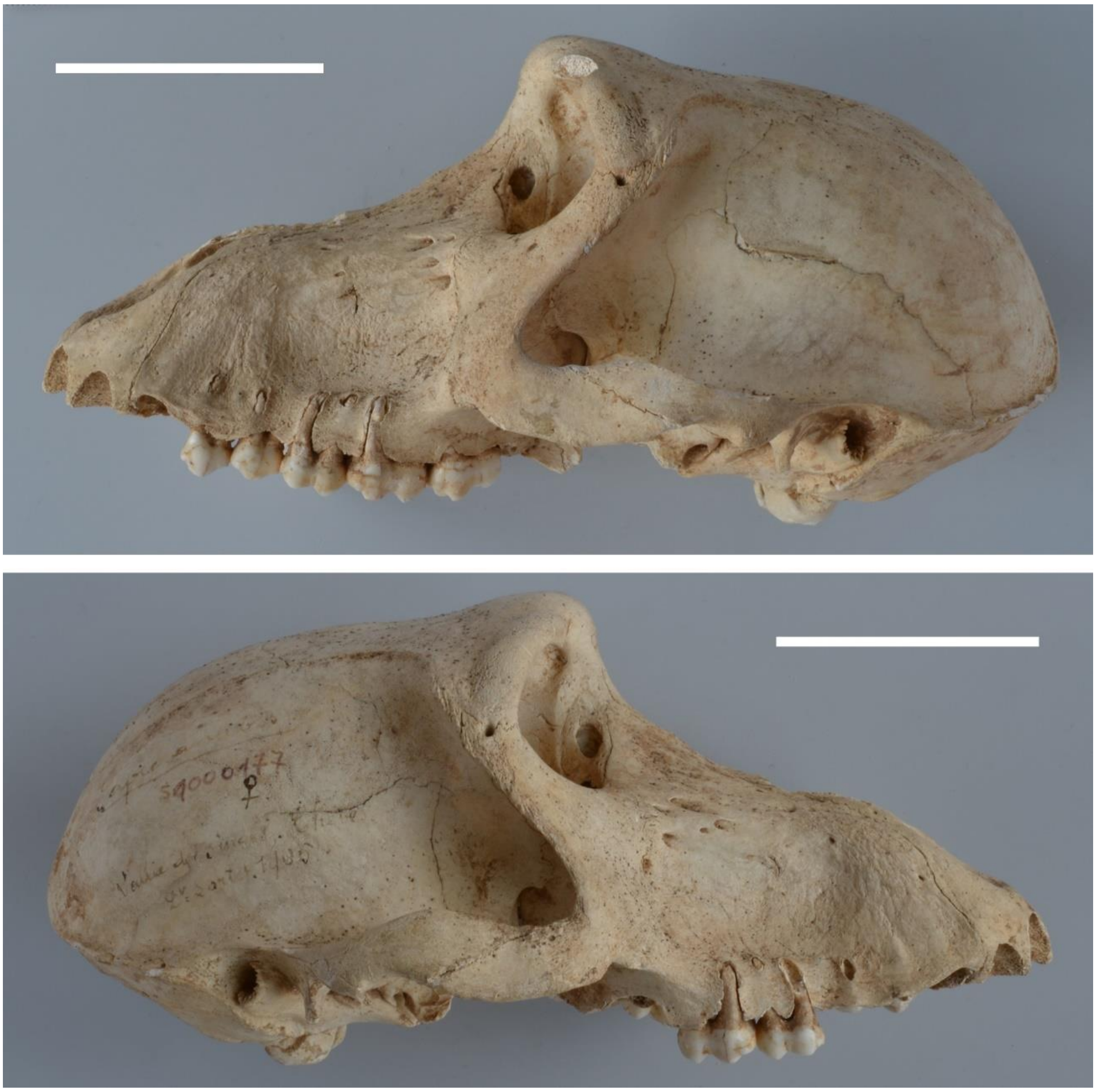

Figure SI-34: Left and right lateral view of the skull of *P. anubis* female MHNL 51000177. Scale bars are 5 cm.

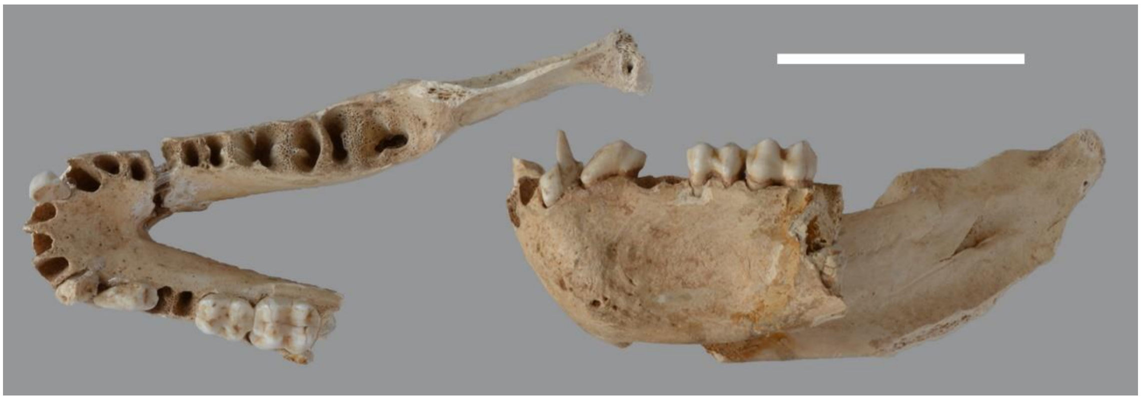

Figure SI-35: Dorsal and left lateral view of the mandible of *P. anubis* female MHNL 51000177. Scale bar is 5 cm.

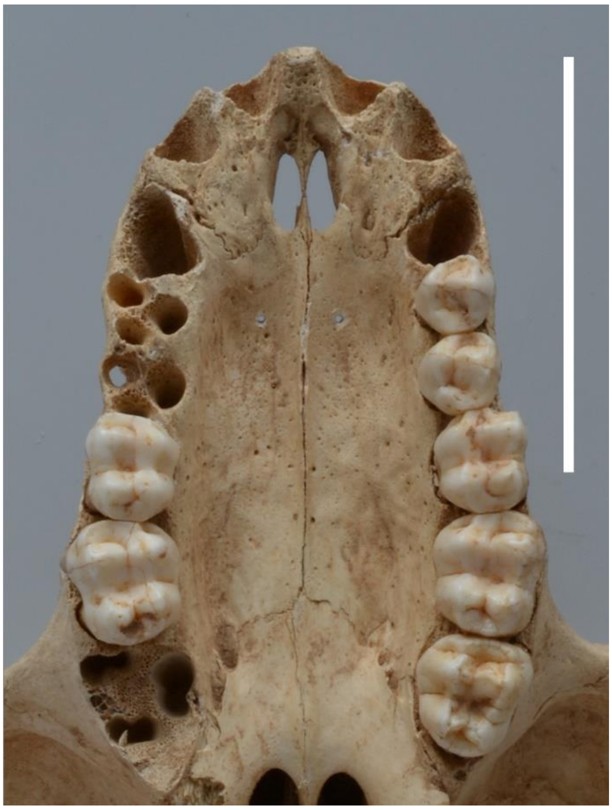

Figure SI-36: Ventral view of the viscerocranium of *P. anubis* female MHNL 51000177, showing the pitting and the undulating surface of the palatine. The two holes with a fresh appearance are artefacts. Scale bar is 5 cm.

Skull MHNL 51000178 *Papio anubis* female; Lortet & Gaillard # 6

Of this individual only the skull is available. The specimen is intact except for two holes in the occipital region, and the post-mortem loss of several teeth. The following dental elements are preserved: right C-M3; left partial canine and M2-3. We also noted the rugose appearance of the basioccipital region.

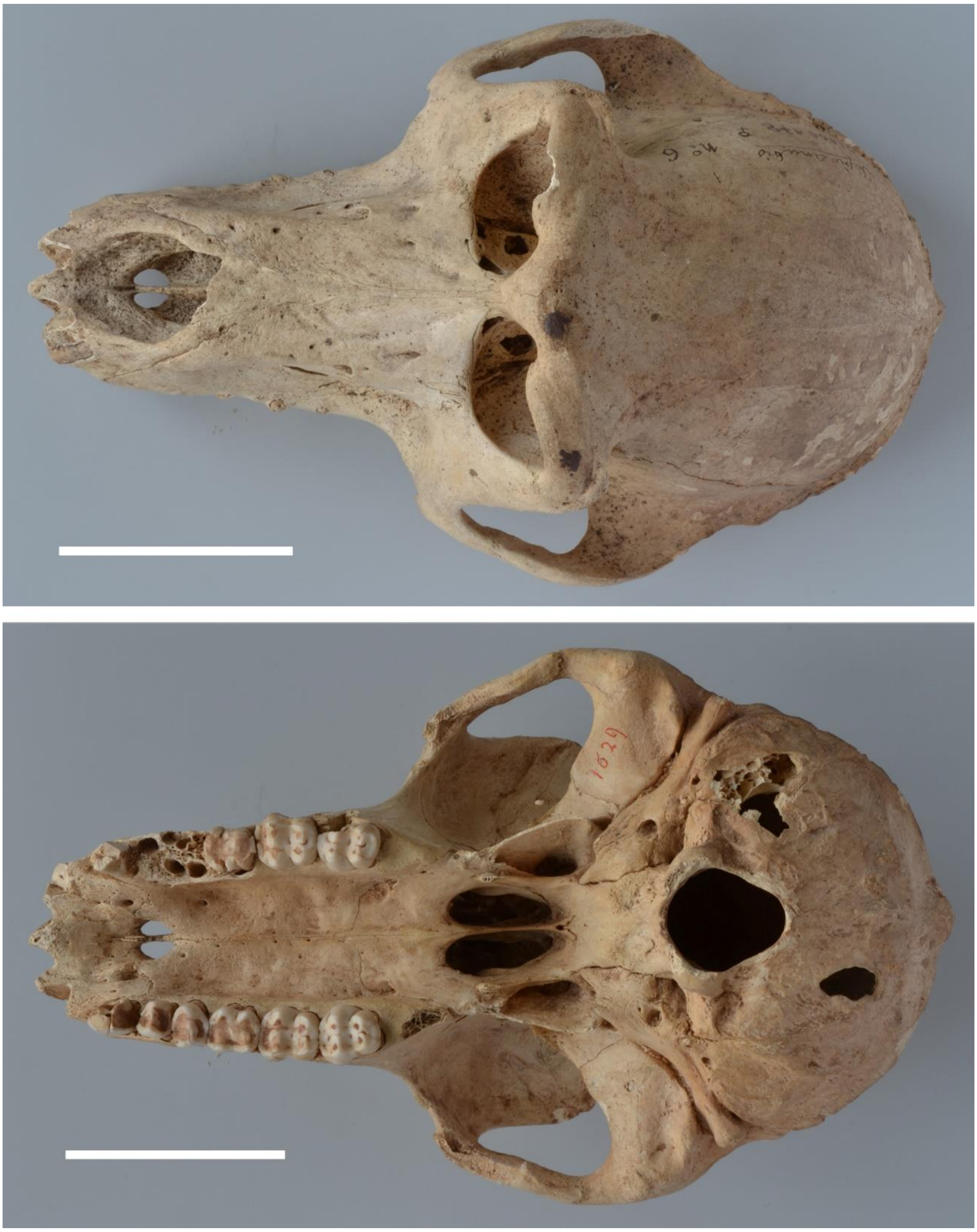

Figure SI-37: Dorsal and ventral view of the skull of *P. anubis* female MHNL 51000178. Scale bars are 5 cm.

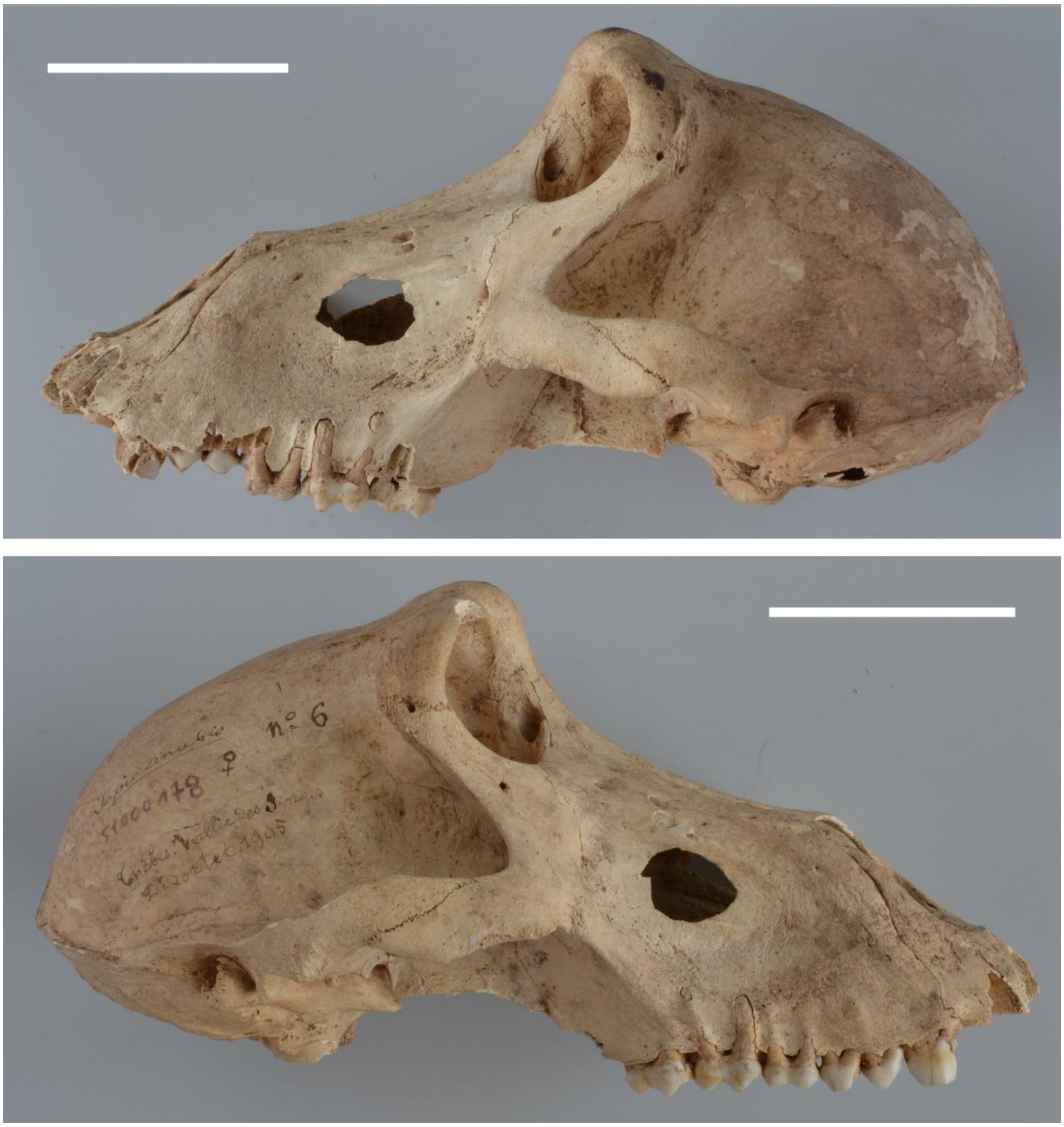

Figure SI-38: Left and right lateral view of the skull of *P. anubis* female MHNL 51000178. Scale bars are 5 cm.

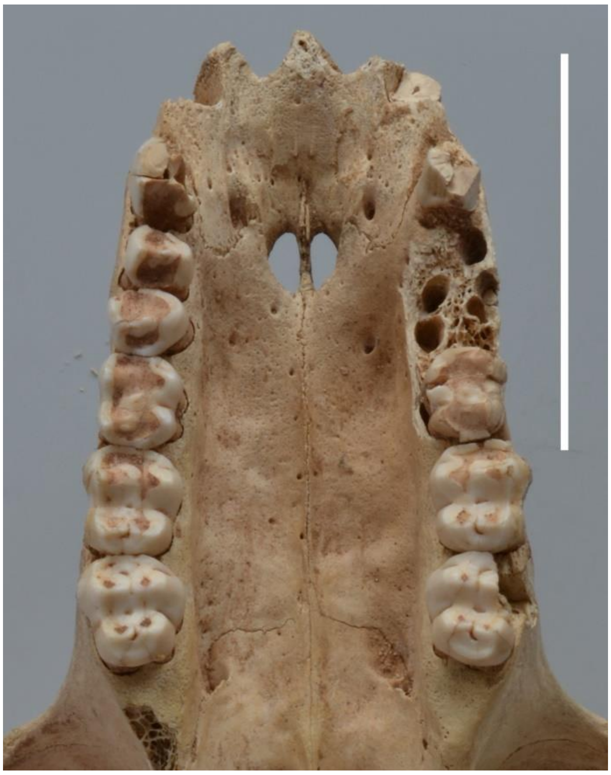

Figure SI-39: Ventral view of the viscerocranium of *P. anubis* female MHNL 51000178, showing the pitting and the undulating surface of the palatine. Scale bar is 5 cm.

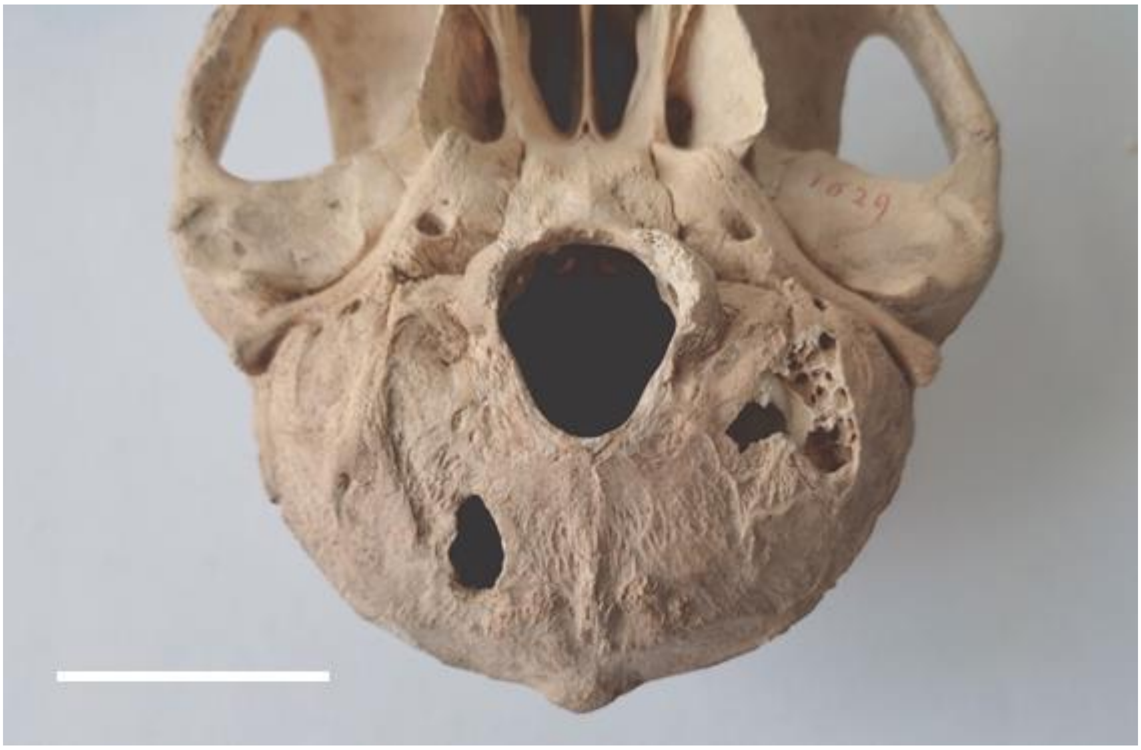

Figure SI-40: Ventral view of hind skull of *P. anubis* female MHNL 51000178, showing the rugose surface of the basioccipital area. Scale bar is 5 cm.

Skull MHNL 51000179 *Papio hamadryas* female; Lortet & Gaillard # 2

The skull is well preserved and the following maxillary teeth are still in situ: right P3-M3; left M1-3. Both left and right mandible are complete including their dentitions, except for the incisors and canines that were lost port-mortem.

At the level of the glabella, the skull exhibits a partial metopic suture. The anterior part of the viscerocranium is asymmetrical, as mentioned also by Lortet and Gaillard (1907: 16). In dorsal view, pitting of the nasal region is obvious. In ventral view, it can be seen that the margins of the palatine – where they meet the lingual side of the teeth – are elevated. On the left side of the skull, an incisor was missing as shown by the alveoli. The nasal septum is not straight but deviated in a left lateral direction. The left premaxilla is only partially developed compared to its right homologue. Both the asymmetry and the missing incisor are indicative of a metabolic disorder rather than a traumatic event. This condition may have affected the germ of the incisor and thus hampered full development of the tooth.

In dorsal view, it can be clearly seen that the left and right half of the mandible are not equally developed: the rostral part of the left half of the lower jaw is more heavily developed producing a marked asymmetry.

A wormian bone - the interparietal or lambdoid bone - is present between the parietals and the occipital. Slightly above the interparietal, two small perforations of about 1 mm diameter are seen along the parietal suture; in addition, a small perforation occurs on the left parietal. On the left side of the interparietal, another rather elliptic perforation of 9 mm long and 3.5 mm high is located on the suture connecting the parietal with the occipital.

In addition, we observed an irregular surface about 15 mm wide and 12 mm high on the left parietal, just medial to the linea temporalis.

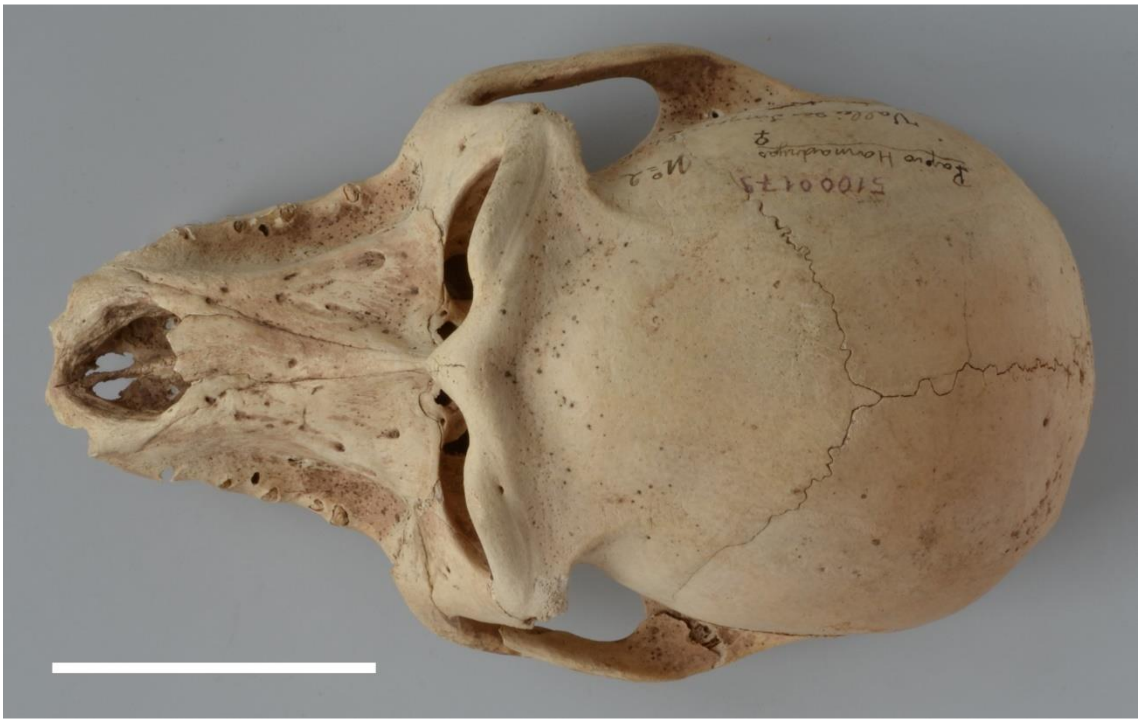

Figure SI-41: Dorsal view of skull of *P. hamadryas* female MHNL 51000179, showing the short and asymmetrical viscerocranium. The pitted nasal region is also clearly visible. Scale bar is 5 cm.

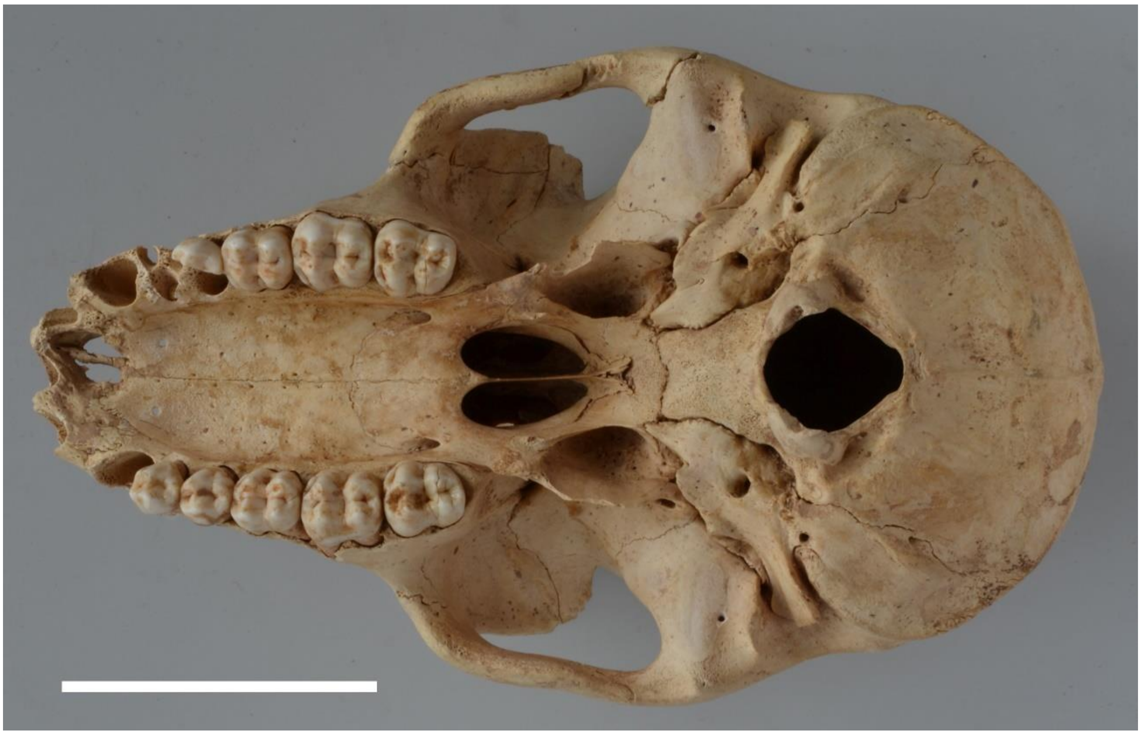

Figure SI-42: Ventral view of the skull of *P. hamadryas* female MHNL 51000179, showing the asymmetrical viscerocranium. Scale bar is 5 cm.

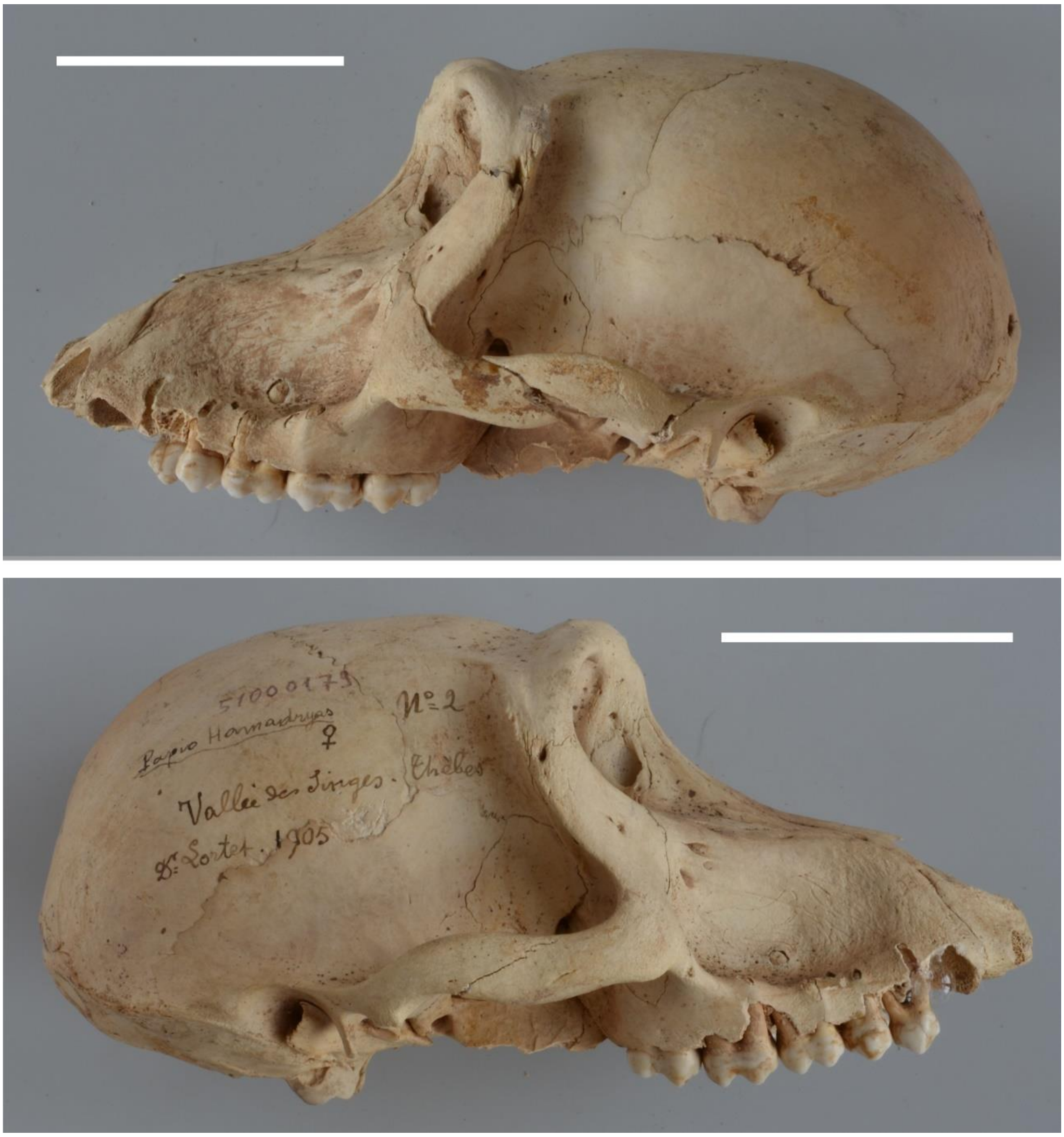

Figure SI-43: Left and right lateral view of the skull of *P. hamadryas* female MHNL 51000179. Scale bars are 5 cm.

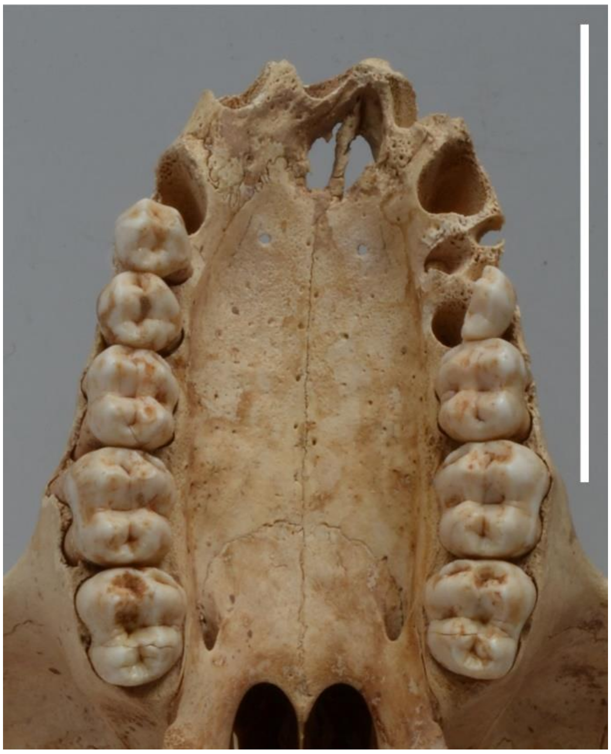

Figure SI-44: Ventral view of the viscerocranium of *P. hamadryas* female MHNL 51000179, showing the asymmetrical nasal septum and the absence of a left incisor. The margins of the palatine are elevated. Scale bar is 5 cm.

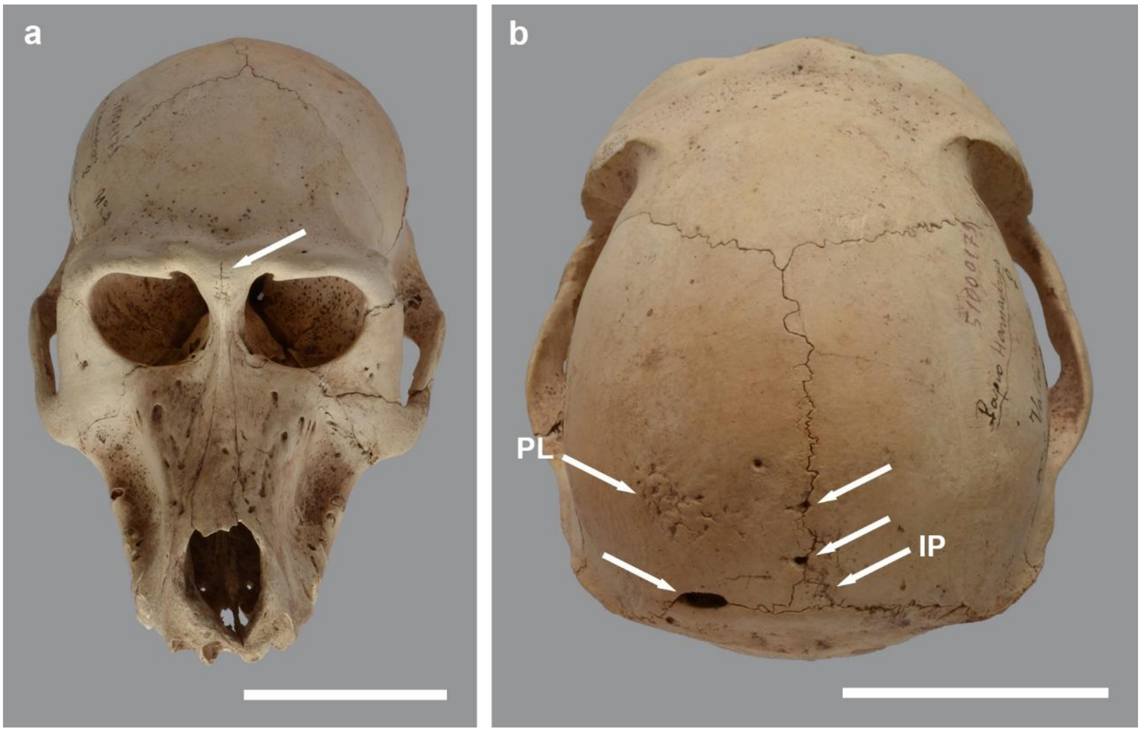

Figure SI-45: a) Dorso-frontal view of skull of *P. hamadryas* female MHNL 51000179, showing the pitted nasal region and the partial metopic suture at the level of the glabella. b) Posterior view of the skull of *P. hamadryas* female MHNL 51000179, showing the presence of an interparietal (IP), a lesion (PL) on the left parietal, and three perforations along the parietal sutures. Scale bars are 5 cm.

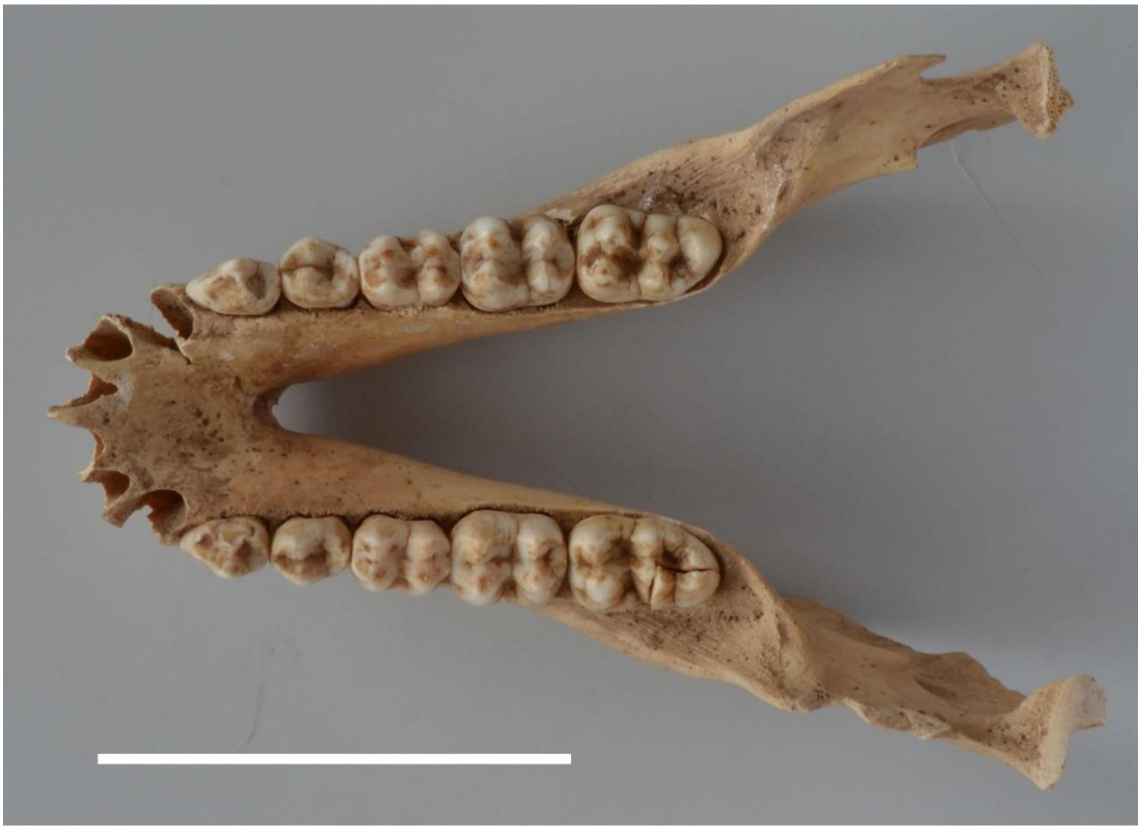

Figure SI-46: Dorsal view of the mandible of *P. hamadryas* female MHNL 51000179, showing the asymmetry in the rostral part. Scale bar is 5 cm.

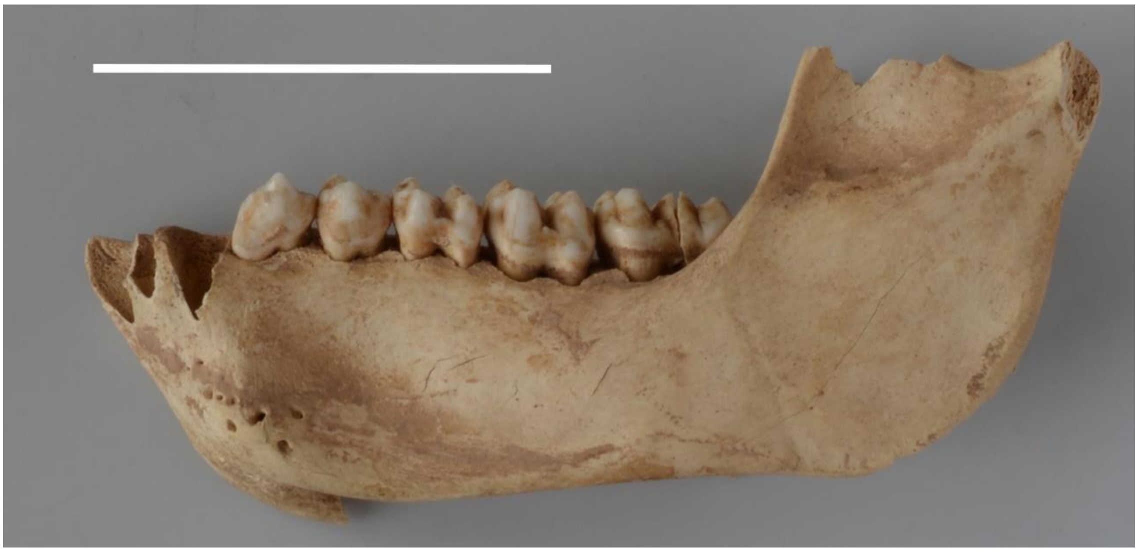

Figure SI-47: Left lateral view of the mandible of *P. hamadryas* female MHNL 51000179. Scale bar is 5 cm.

Skull MHNL 51000180 *Papio* sp. male juvenile; Lortet & Gaillard # 12

The skull of this juvenile animal is damaged on its left lateral side and the vertical rami of both mandibles are incomplete. In the right maxilla the P3 and P4 are still in their crypt, no erupted teeth are in place, but the posterior-most alveoli are those for the M2. In the lower jaws, the M2s are erupting. These dental stages suggest an age at death around 4 years (Reed, 1965, 1967). Despite the young age of the animal, it is obvious that this is a male individual. Its skull is larger than the adult females of the same collection. This skull was identified as *P. hamadryas* by Lortet and Gaillard (1907) but because of the young age of this individual we prefer classifying it as *Papio* sp.

On the skull, a partial metopic suture is observed at the level of the glabella. The palatine is raised near the tooth rows and in the middle part of the palate, several small perforations occur.

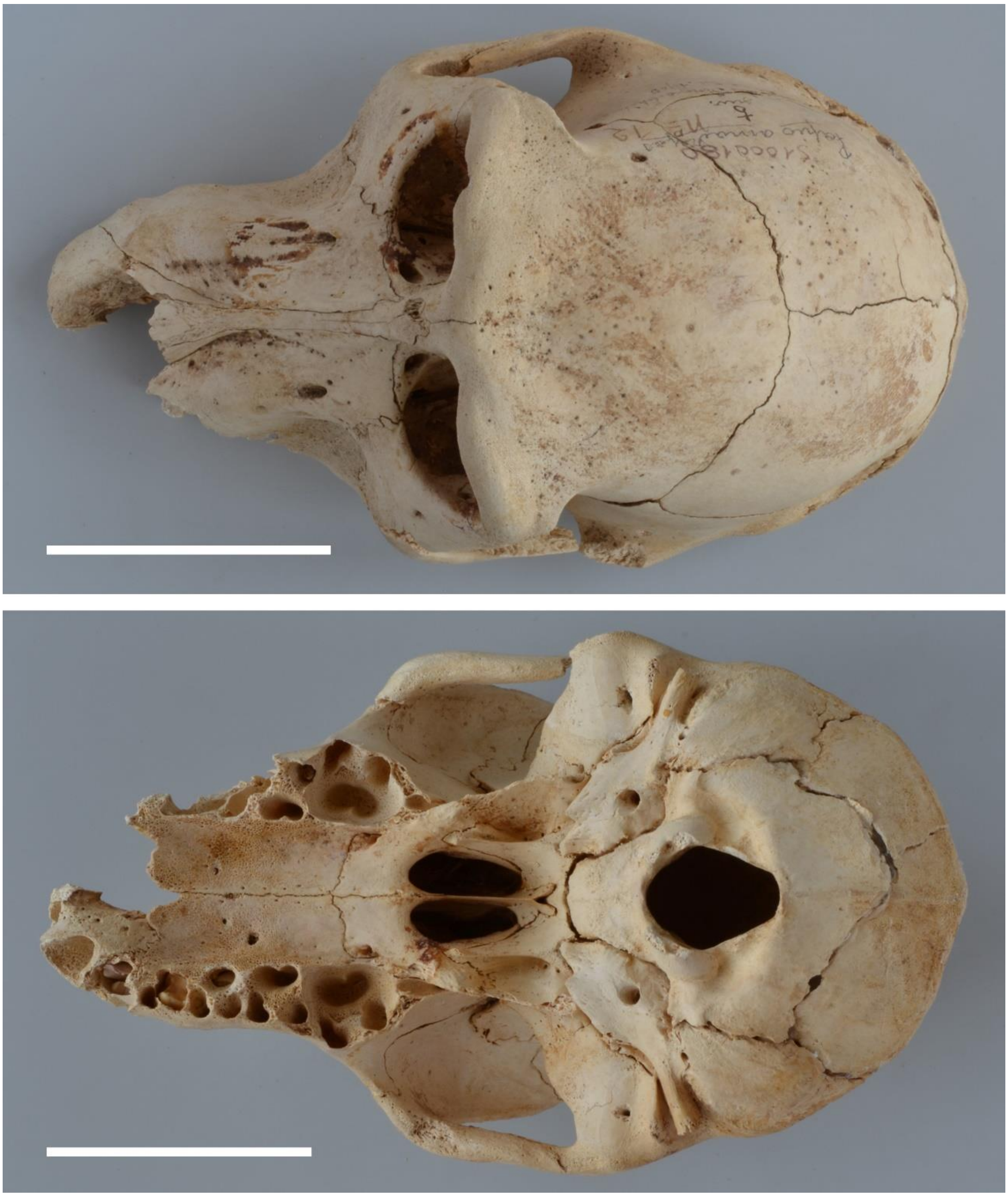

Figure SI-48: Dorsal and ventral view of skull of young *Papio* sp. male MHNL 51000180. Scale bars are 5 cm.

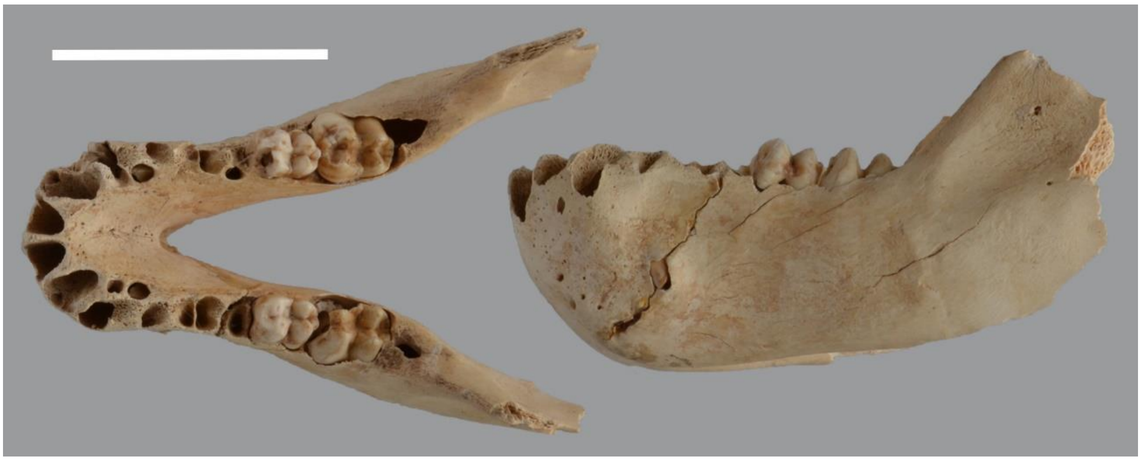

FigureSI-49: Dorsal and left lateral view of mandible of young *Papio* sp. male MHNL 51000180. Scale bars are 5 cm.

Skull MHNL 51000181 *Papio* sp.; (Lortet & Gaillard 1909: 7)

This skull is depicted in Lortet and Gaillard (1909: 7) and described separately from those mentioned above. The specimen has undergone some damage since its first description as most of the snout and the left jugal arch has disappeared since then. The animal was identified as *Papio hamadryas*, an identification that we find hard to confirm using the partial skull. We could not verify if the angle between the vertical plane across the orbitae and the horizontal plane along the osseous palate corresponds to what is expected in *P. hamadryas*.

No maxillary teeth are present, but the alveoli for M1-M3 can be seen. The entire skull is thickened and the surface of the frontals is rugose. The sutures of the parietals with the frontals and the occipital are not closed and there is a furrow between the left and right parietal. The sutures of the left and right frontals are closed, but those between the parietals and the squamosa are not. There is also a metopic suture at the level of the glabella. The heavy skull bones, the deep sutures, and the incompletely fused cranial bones (parietals, occipital, squamosal) caused Dr Poncet (in Lortet and Gaillard 1909: 6-8) to diagnose this specimen as a case of Paget’s disease, a diagnosis that is confirmed by the X-ray we took. It can be seen clearly that the thickening is most pronounced in the fronto-parietal region.

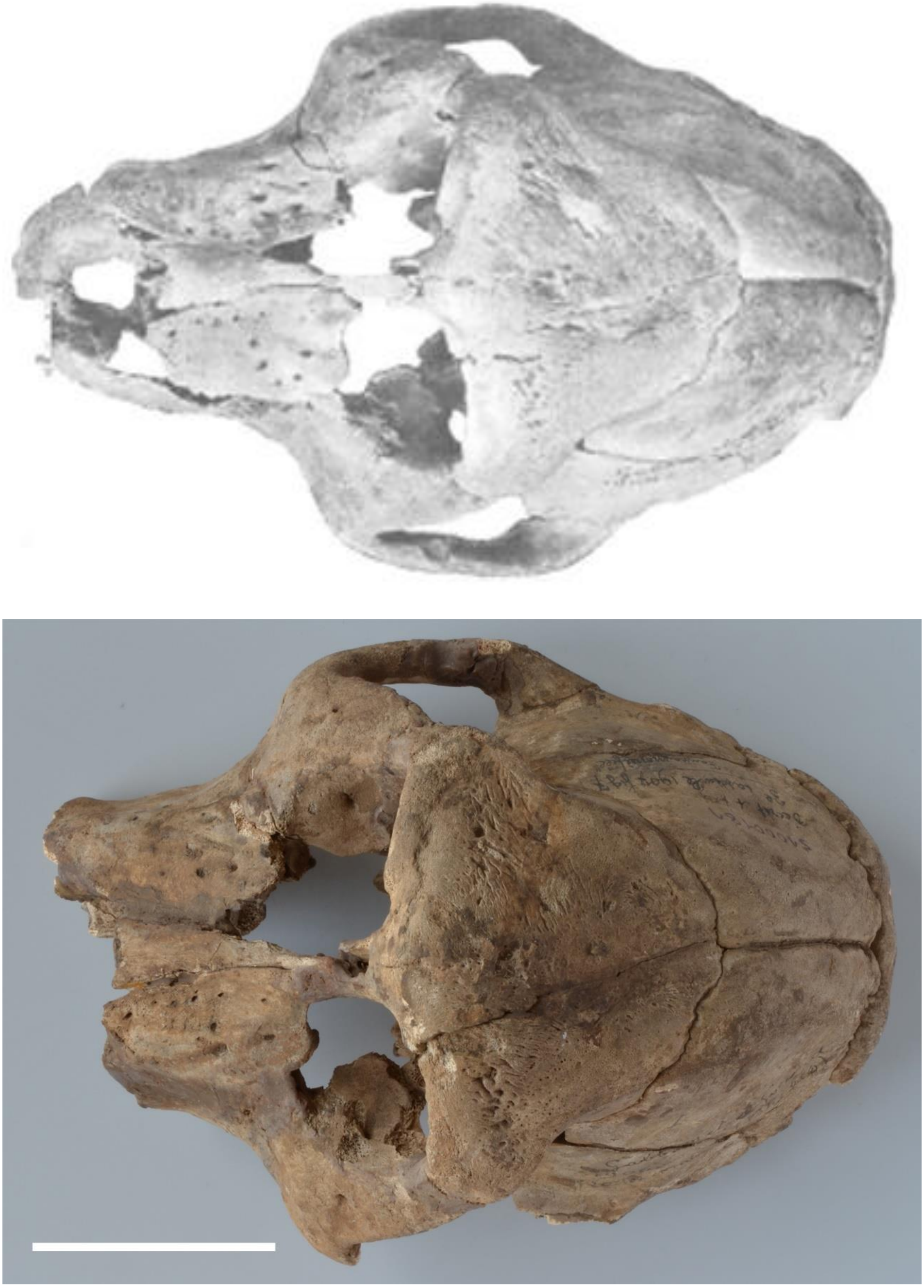

Figure SI-50: Dorsal view of skull of *Papio* sp. MHNL 51000181 compared to the depiction in Lortet & Gaillard (1909, p. 7, fig 7). Scale bar is 5 cm.

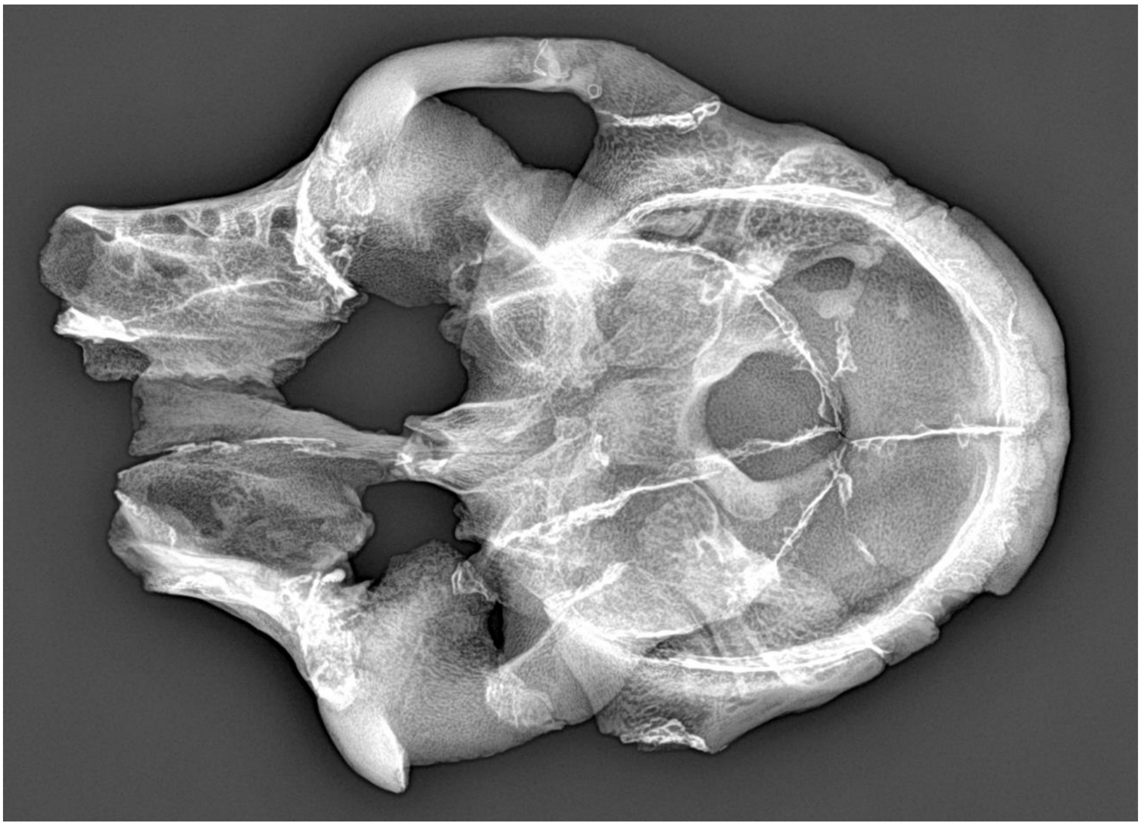

Figure SI-51: X-ray in dorso-ventral view of the skull of *Papio* sp. MHNL 51000181, showing the thickening of the fronto-parietal region (X-Ray : R. Lichtenberg).

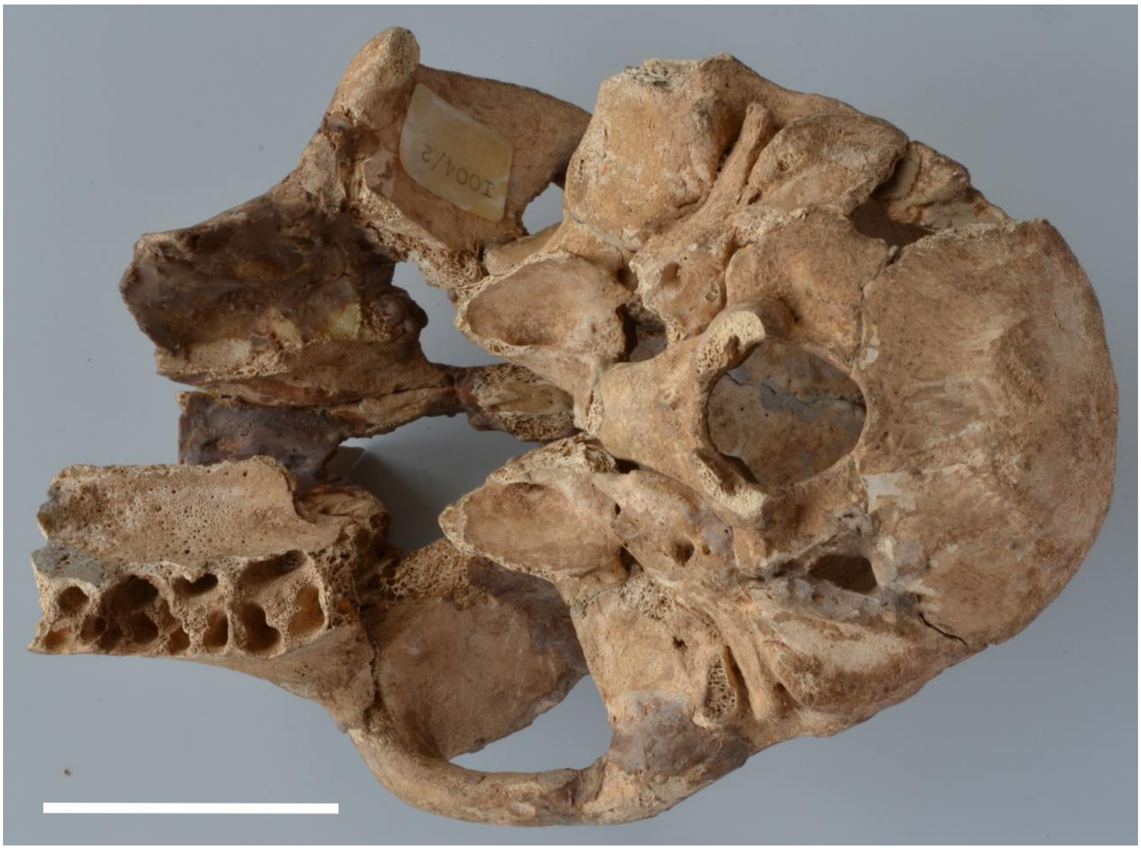

Figure SI-52 : Ventral view of skull of *Papio* sp. MHNL 51000181. Scale bar is 5 cm.

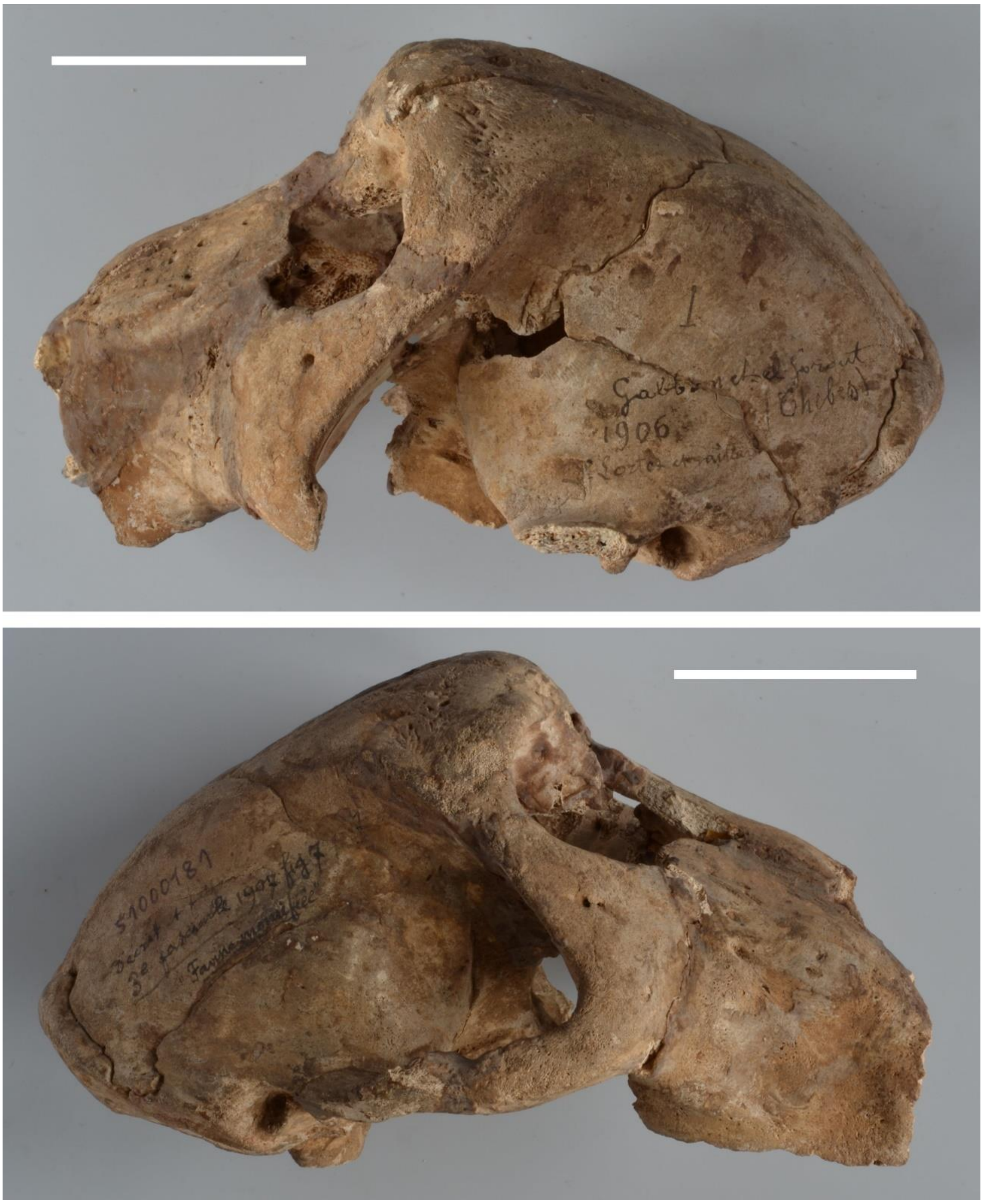

Figure SI-53: Left and right lateral view of skull of *Papio* sp. MHNL 51000181. Scale bars are 5 cm.

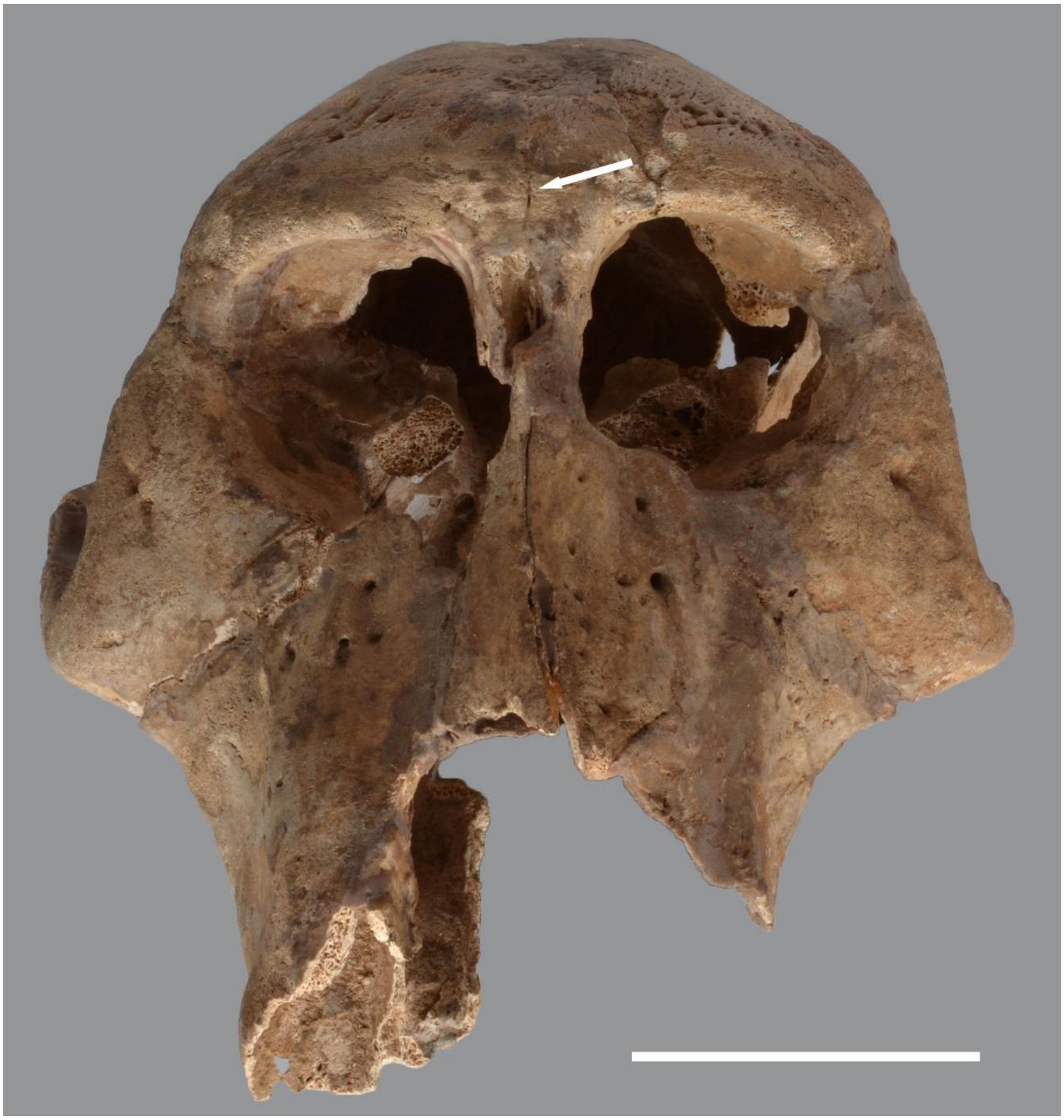

Figure SI-54: Dorsofrontal view of skull of *Papio* sp. MHNL 51000181 the partial metopic suture at the level of the glabella. Scale bar is 5 cm.

Skull MHNL 51000182 *Papio anubis* male; not in Lortet & Gaillard

Only a small part of this skull is preserved, namely the orbital area, the frontals and part of the parietals. On the lateral side of the skull it is noticed that the surface is rugose. The lineae temporales project laterally and the area between them is wide and smooth. Towards the back of the skull, the temporal lines start converging, suggesting that this is a male individual. This is confirmed by the overall size of the skull and the heavy supraorbital ridges.

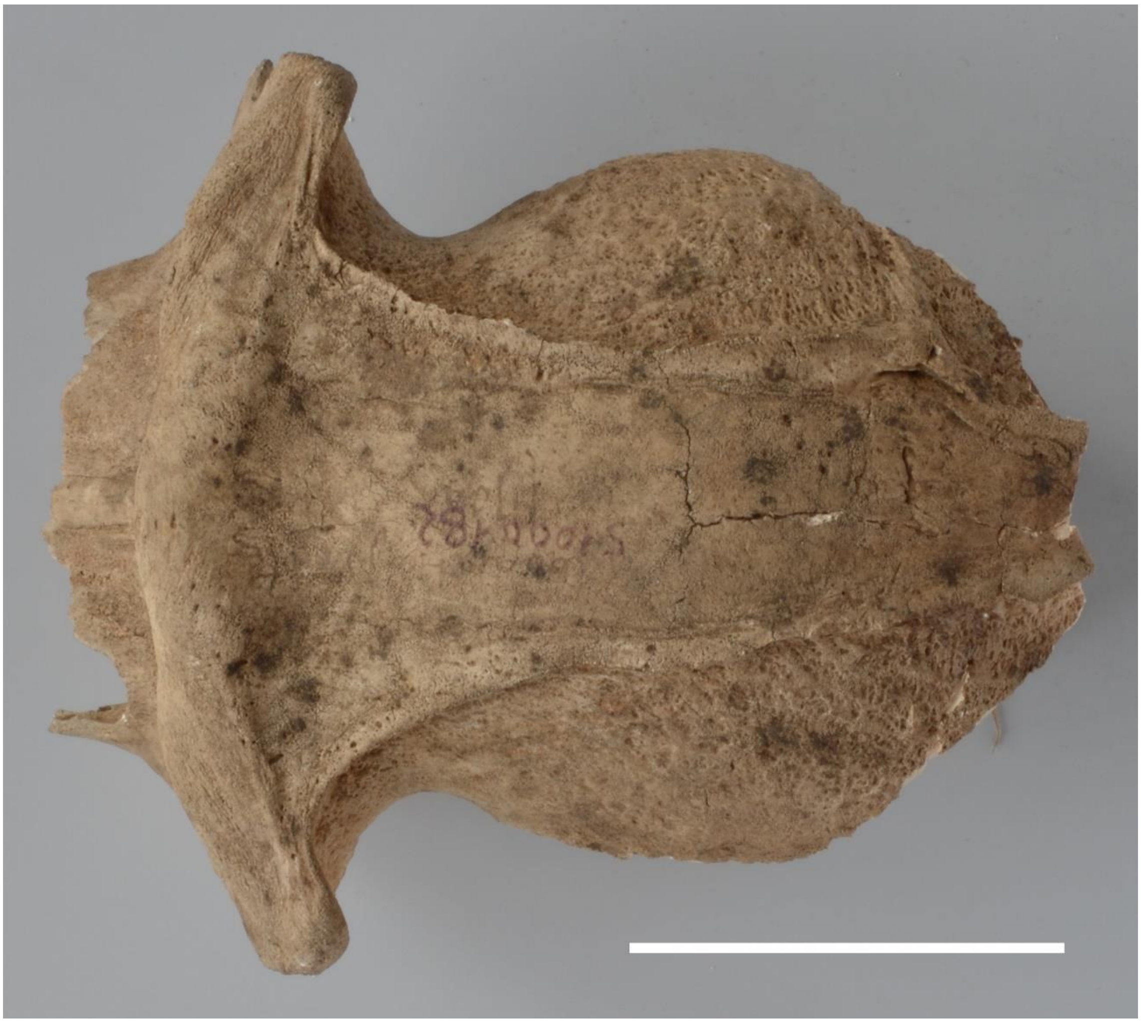

Figure SI-55: Dorsal view of skull of *P. anubis* male MHNL 51000182, showing the wide, flat area between the lineae temporales, and the rugose surface of the parietals. Scale bar is 5 cm.

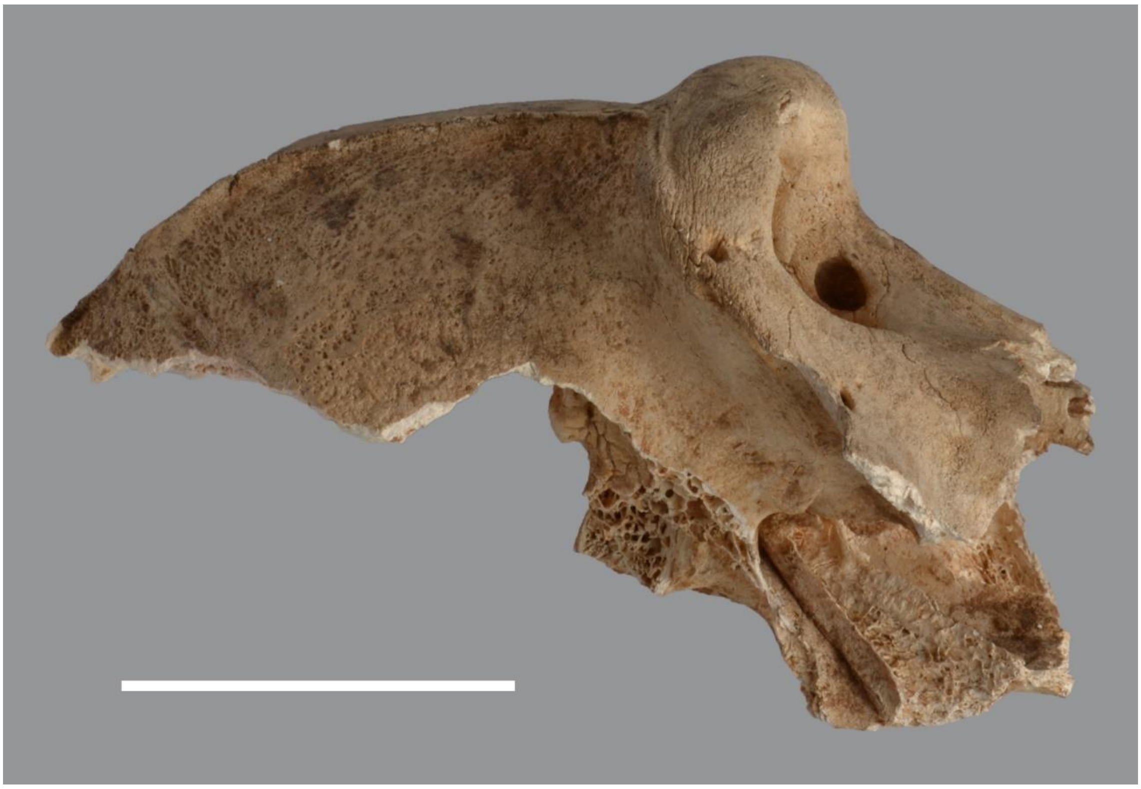

Figure SI-56: Right lateral view view of skull of *P. anubis* male MHNL 51000182. Scale bar is 5 cm.

Skull MHNL 51000183 *Papio hamadryas* male; Lortet & Gaillard # 7

The skull is rather well preserved, except for some damage in the right orbital area and the absence of the right jugal arch. There has been some post-mortem tooth loss, but the following teeth are still in situ: right P4, M1 and M3; left P4-M2. The mandibles of this individual are missing.

In ventral view, it is noticed that the incisive fossae have irregular margins and that the bone surface of the ossa incisiva is pitted. It also appears that the snout is slightly distorted in a left direction, as reflected by the difference in distance between the canine and second incisor: the right diastema is more spacious compared to the left one.

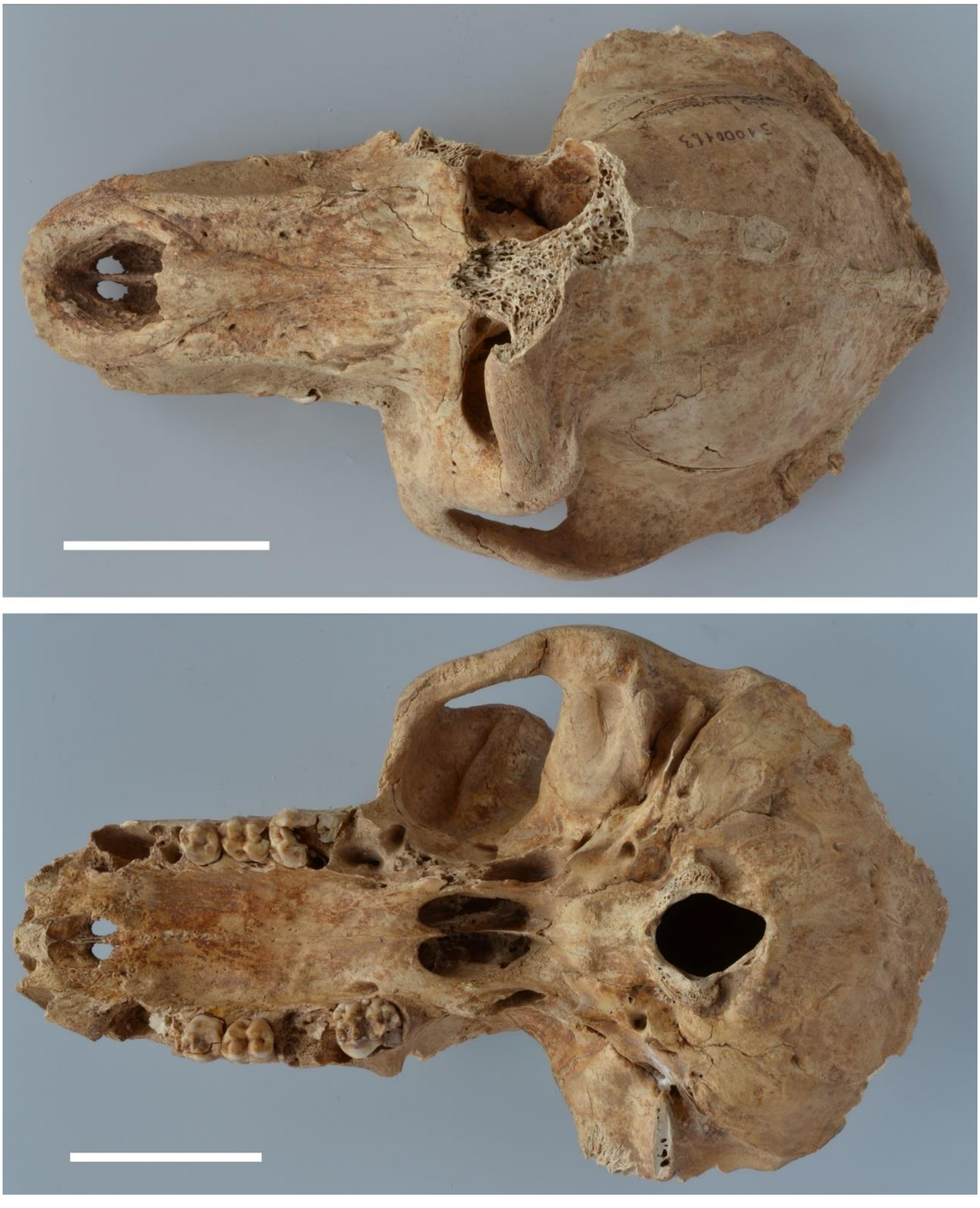

Figure SI-57: Dorsal and ventral view of the skull of *P. hamadryas* male MHNL 51000183. Scale bars are 5 cm.

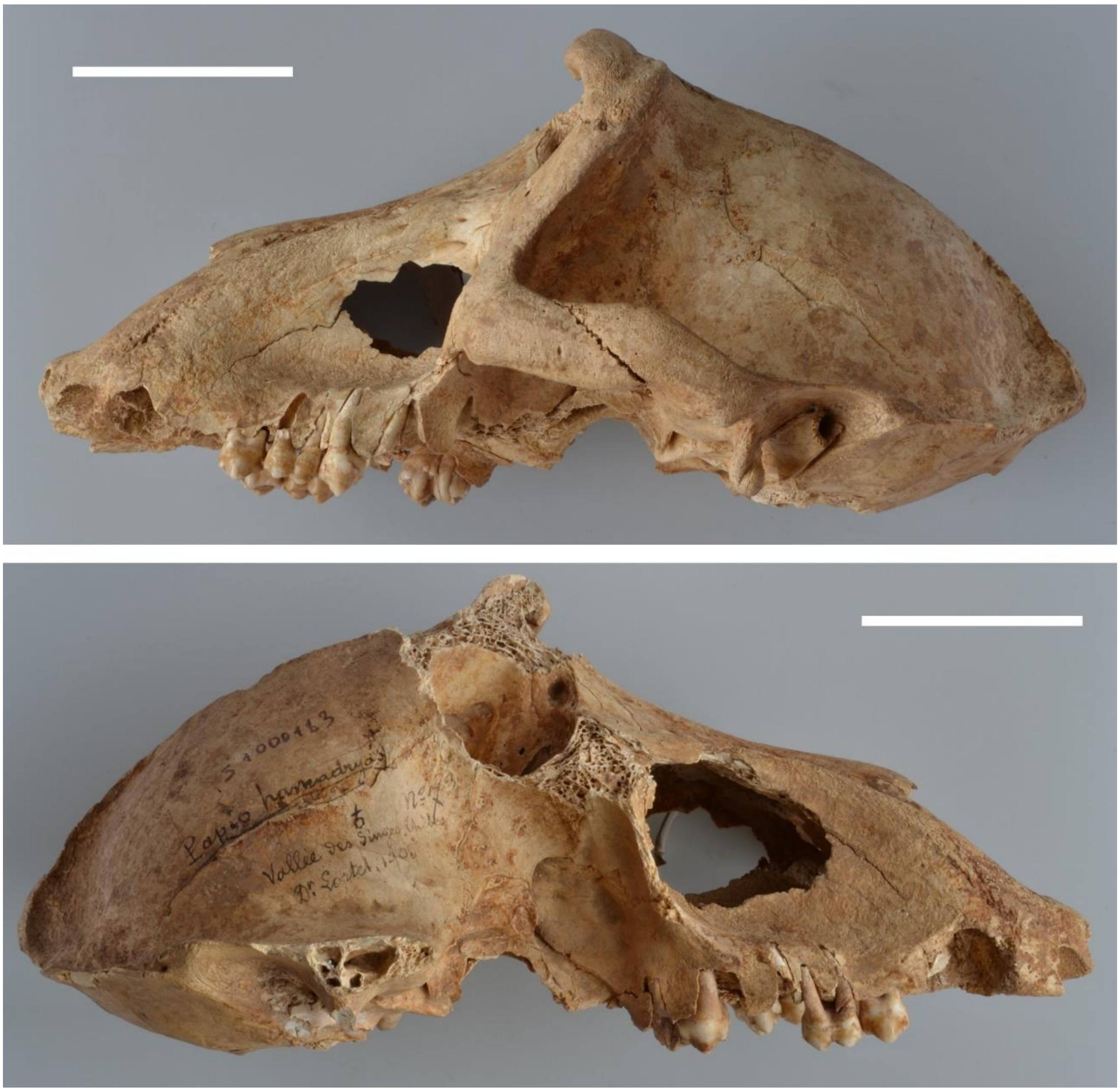

Figure SI-58: Left and right lateral view of the skull of *P. hamadryas* male MHNL 51000183. Scale bars are 5 cm.

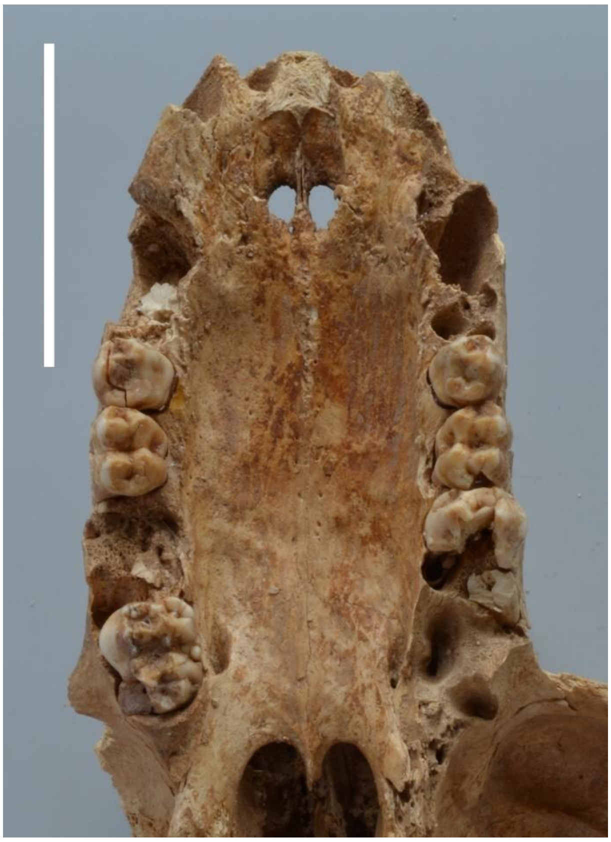

Figure SI-59: Ventral view of the viscerocranium of *P. hamadryas* male MHNL 51000183, showing the distorted snout and the irregular surface of the incisive area. Scale bar is 5 cm.

Skull MHNL 51000318 *Papio* sp.; not in Lortet & Gaillard

Only the hind skull of this young individual is preserved. The frontals and the parietals have an irregular surface that is not smooth but somewhat pitted. The bones also seem rather thick. On the X-rays, the beginning of a thickening is observed. Both the right and left glenoid fossa have an irregular porous appearance, and the surface seems to be slightly protruding from the articular surface forming a faint medio-lateral ridge.

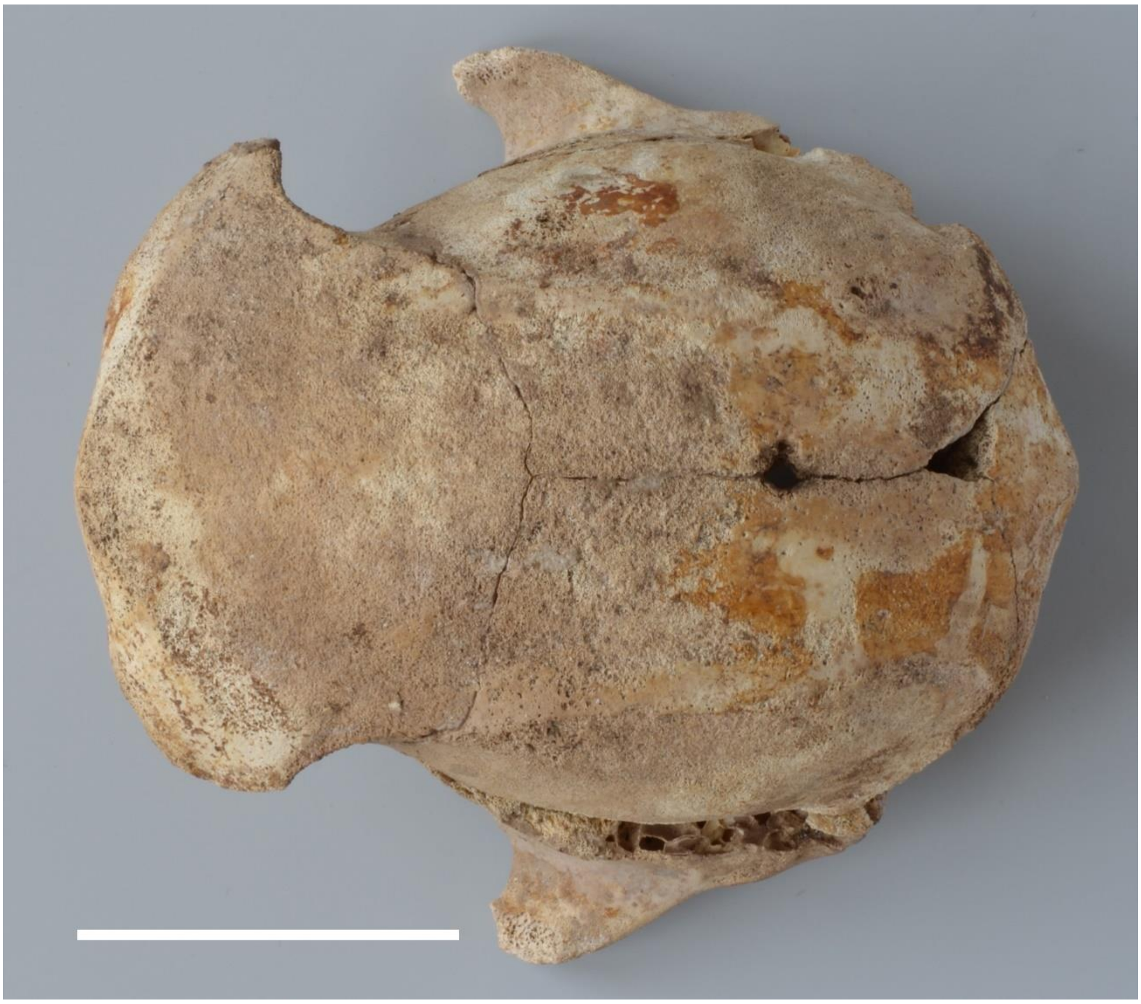

Figure SI-60: Dorsal view of the skull of *Papio* sp. MHNL 51000318, showing the pitting of the frontals and parietals. Scale bar is 5 cm.

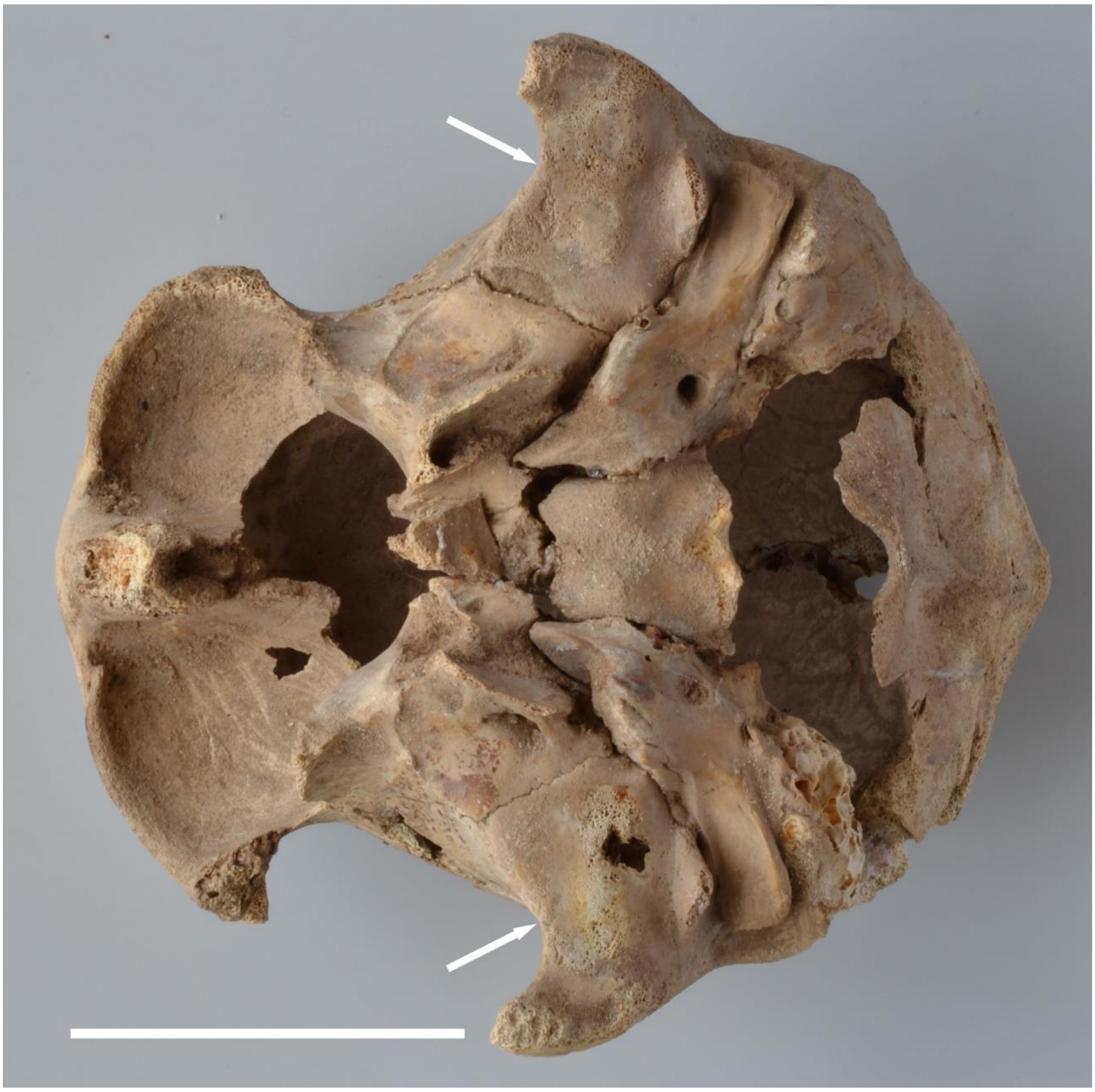

Figure SI-61: Ventral view of the skull of *Papio* sp. MHNL 51000318 with indication of the pitted glenoid fossae. Scale bar is 5 cm.

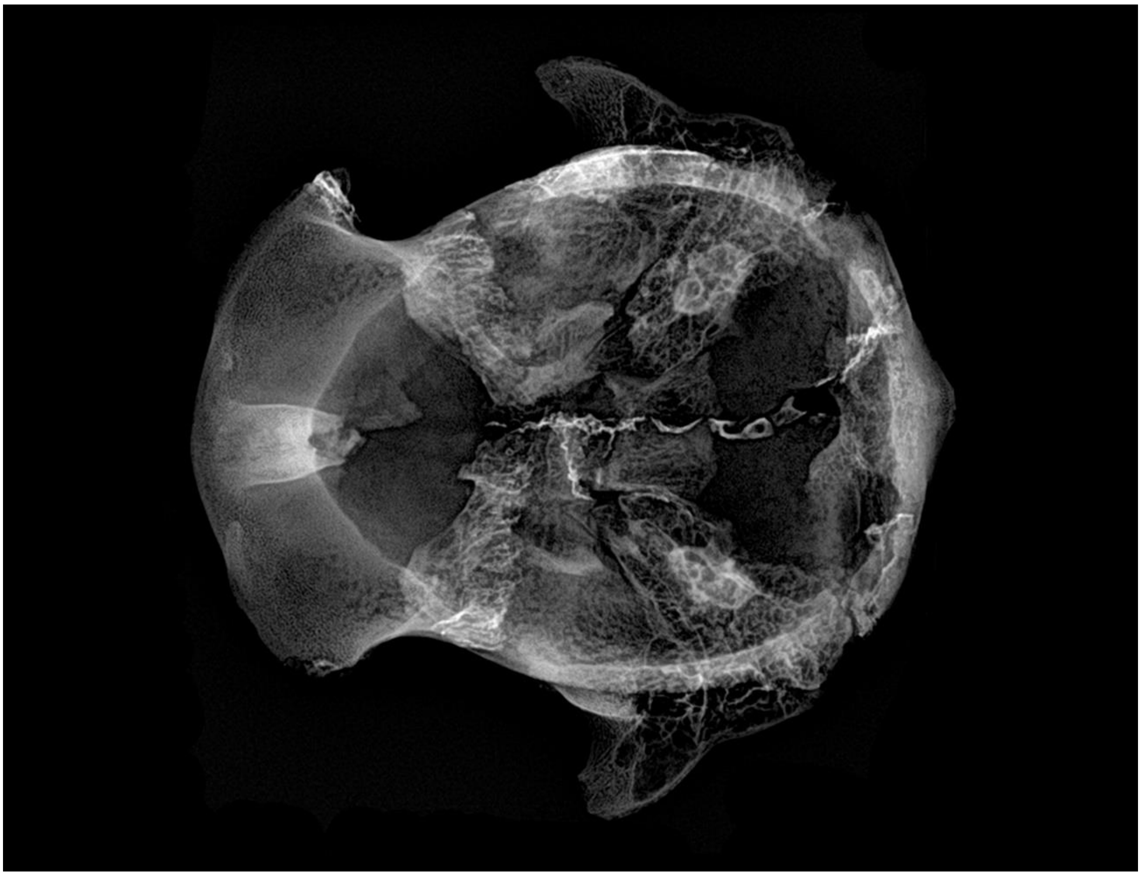

Figure SI-62: X-ray in dorso-ventral view of the skull of *Papio* sp. MHNL 51000318, showing the thickening of the fronto-parietal region. (X-Ray : R. Lichtenberg).

Skull MHNL 51000319 *Papio* sp. male; not in Lortet & Gaillard

Only the hind skull of this apparently adult animal is preserved. The lateral parts of the frontals and the parietals have an irregular, rugose surface. The lineae temporales are laterally extended and the area delineated by them is smooth. Only towards the posterior end of the skull – in the last fifth of the structure – both lines converge into a single crest.

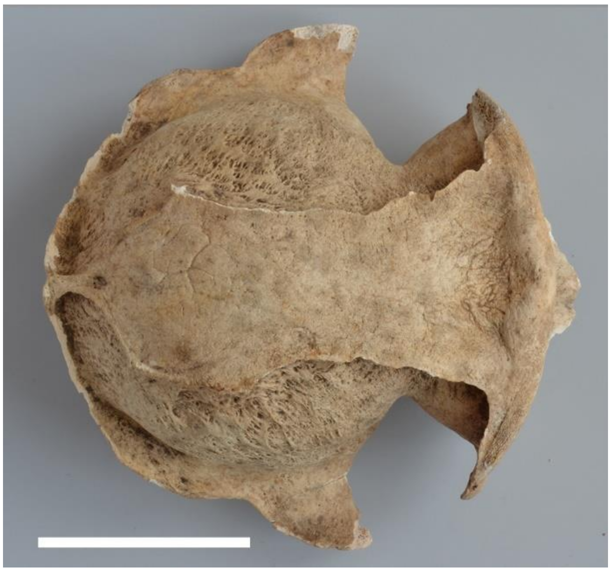

Figure SI-63: Dorsal view of the hind skull of *Papio* sp. male MHNL 51000319, showing the rugose surface of the lateral parts, as well as the laterally extended lineae temporales and the wide, smooth surface between them. Scale bar is 5 cm.

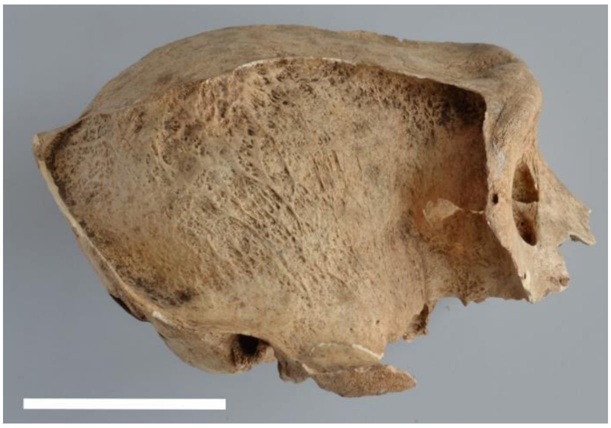

Figure SI-64: Right lateral view of the hind skull of *Papio* sp. male MHNL 51000319, showing the rugose surface of the parietals. Scale bar is 5 cm.

Skull MHNL 51000320c *Papio anubis* female; Lortet & Gaillard # 4

The skull of this subadult individual is more or less intact. The left P3 is the sole erupted tooth that is completely preserved. The left and right upper M3 are still in their crypt. The left posterior half of the mandible is missing and only the right M2 and M3 that are still completely preserved. The last lower molar is still erupting.

This skull shows a slight metopic suture at the level of the glabella. A fissure can be seen in the occipital condyles, evidencing that the fusion between the basi- and exoccipital bone was not complete.

Several abnormalities are observed in the dentition. The left and right upper third molars are still in their tooth sockets.The cusps do not seem to be developed properly, a characteristic that is also – and better – observed in the right lower M3. In the skull, the left and right canines are not visible, and the area where they can be expected has a swollen appearance. This suggests that the canines may not have been fully developed and remained inside their sockets. We think this explanation – an idea suggested by the microdonty observed in the upper M3 – is more plausible than a diagnosis as agenesis. Because of the absence of the canines there is a rather wide bony space between the I2 and the P3. In the maxillae it is moreover noticed that there is a gap between the P3 and P4.

In the mandible, an alveolus for the left canine is present, but on the opposite side this tooth was not present and there is a gap between the alveolus of the I2 and anterior alveolus of the P3. However, in this right mandible, a cavity is observed lingually of the anterior alveolus of the P3. Measuring approximately 4mm in diameter, this opening may correspond to the location where the tip of the canine could eventually protrude.

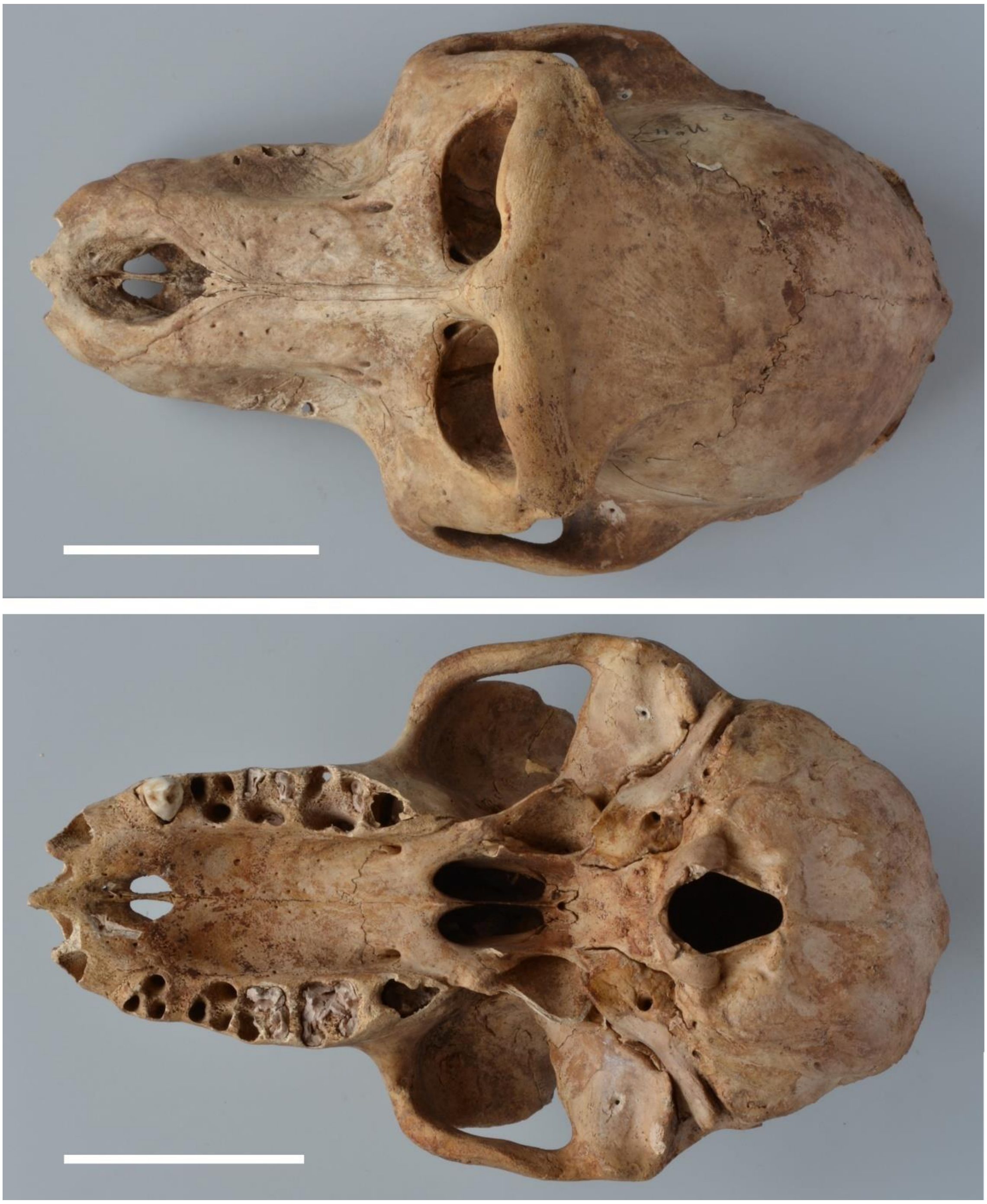

Figure SI-65: Dorsal and ventral view of the skull of *P. anubis* female MHNL 51000320c. Scale bars are 5 cm.

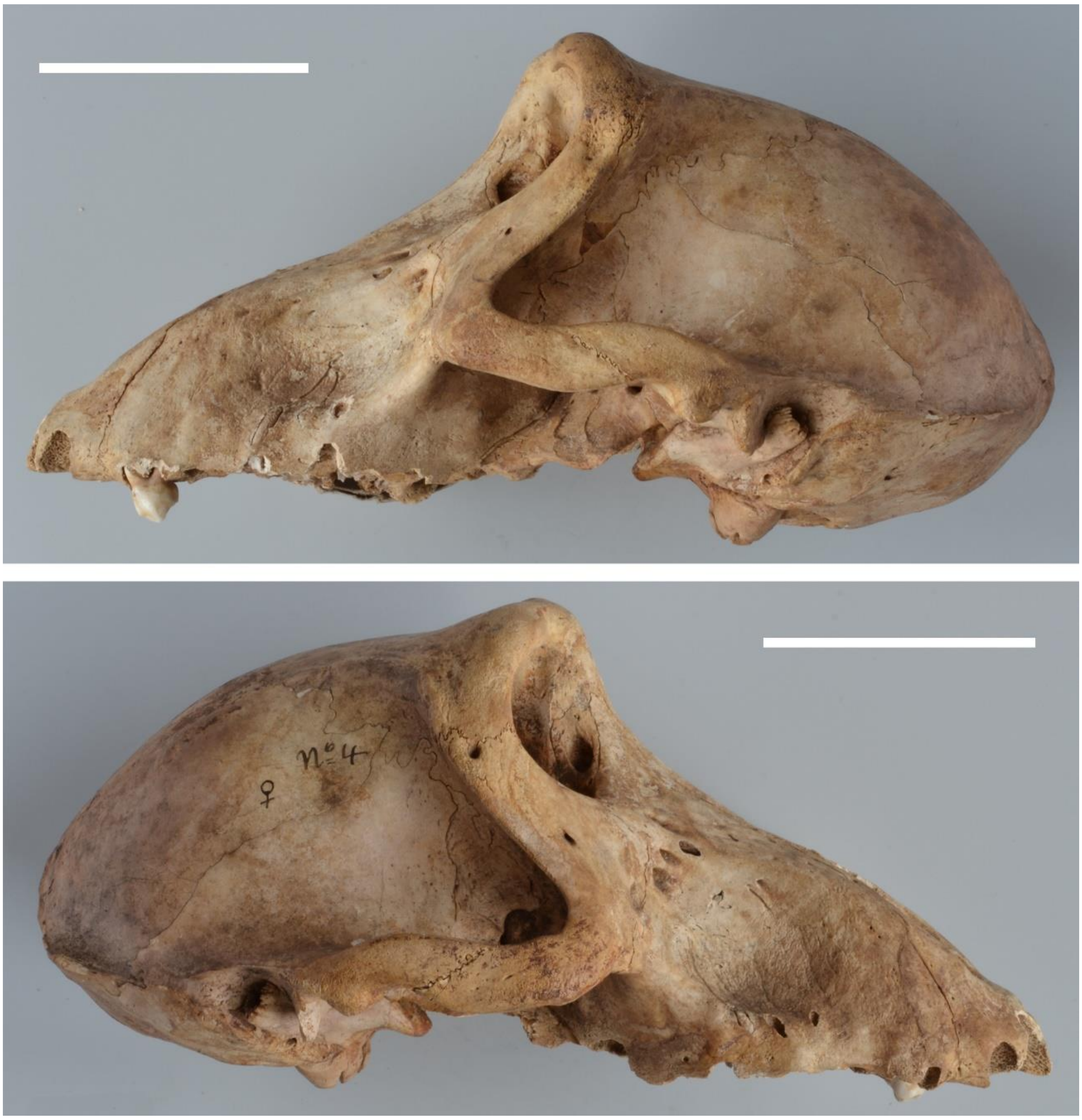

Figure SI-66: Left and right lateral view of the skull of *P. anubis* female MHNL 51000320c. Scale bars are 5 cm.

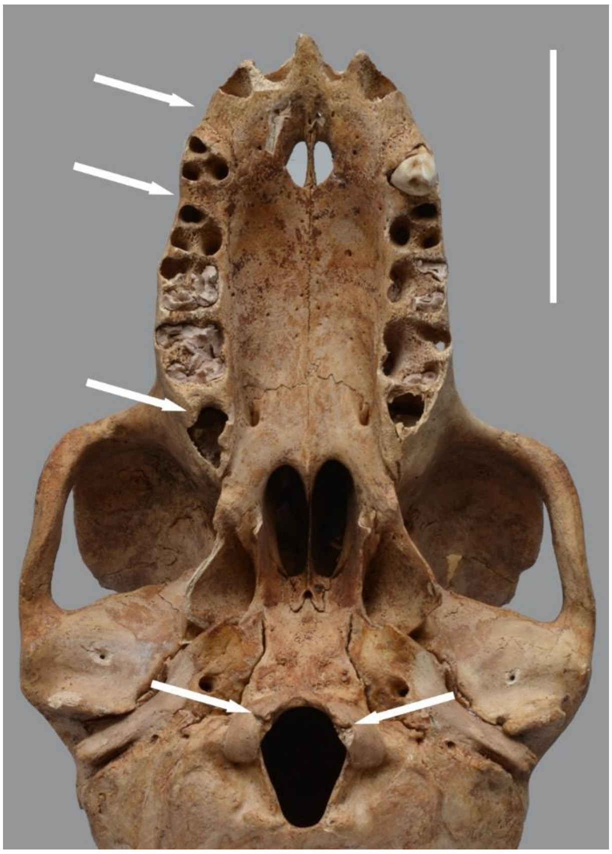

Figure SI-67: Ventral view of the skull of *P. anubis* female MHNL 51000320c, showing the fissure in the occipital condyles. A clear gap is visible between the I2 and the P3, as well as between the P3 and P4. The poorly developed M3 are still in their crypts. Scale bar is 5 cm.

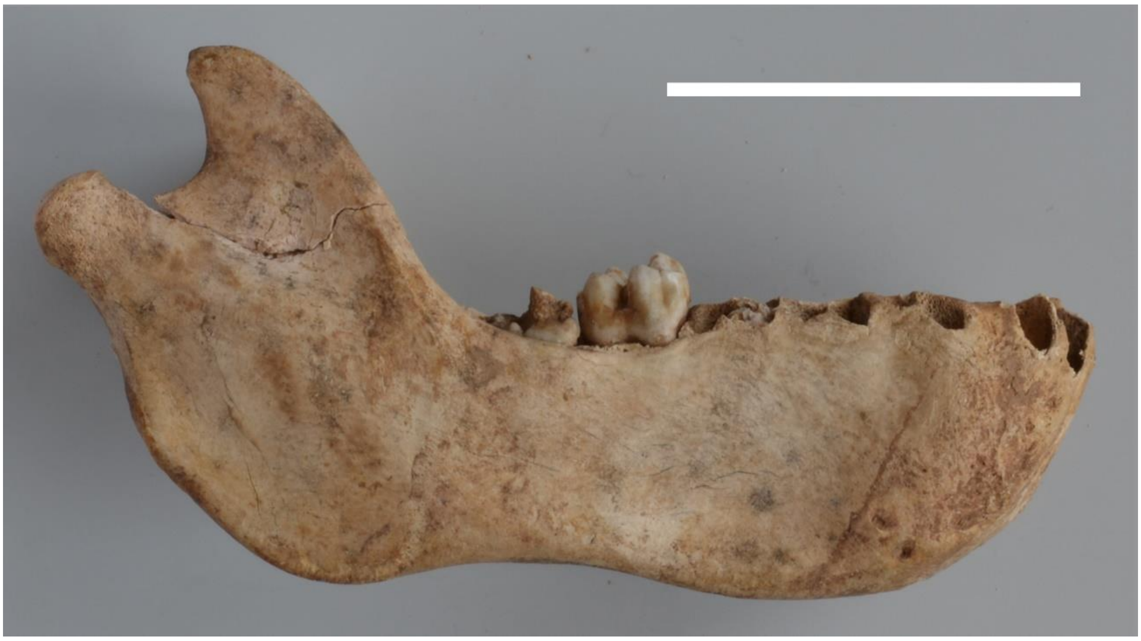

Figure SI-68: Right lateral view of the mandible of *P. anubis* female MHNL 51000320c. Scale bar is 5 cm.

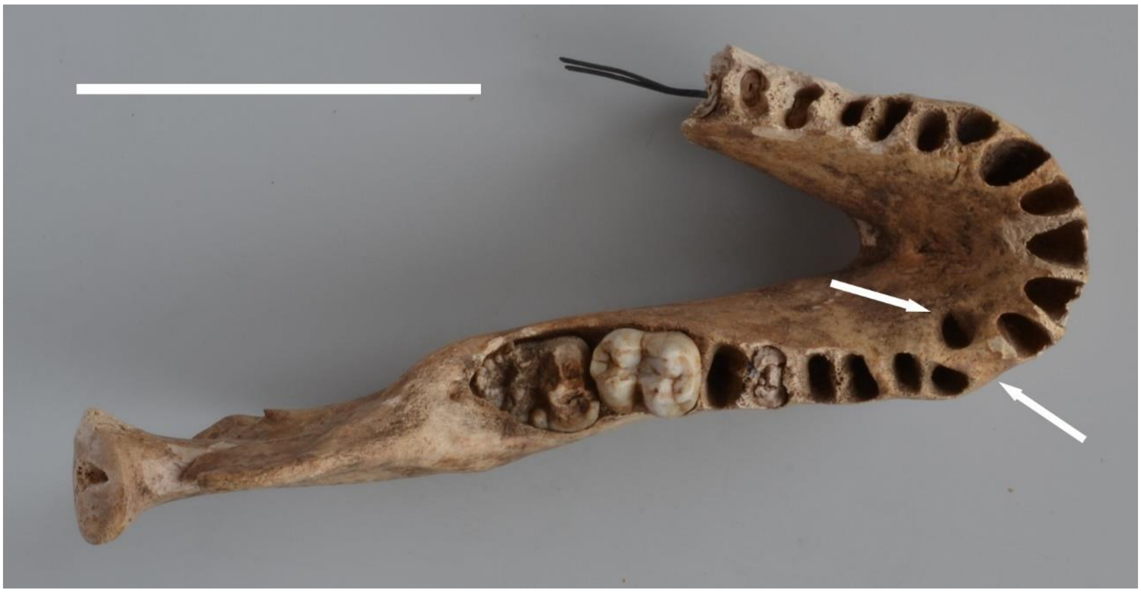

Figure SI-69: Dorsal view of the mandible of *P. anubis* female MHNL 51000320c, showing the location where the right canine is missing, as well as the large perforation lingually of the tooth row. The defective crown of the right M3 is also clearly visible. Scale bar is 5 cm.

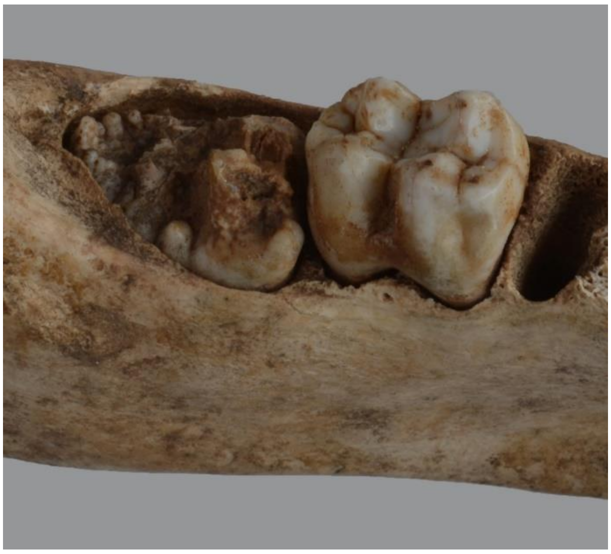

Figure SI-70: Detail of the two last lower right molars of *P. anubis* female MHNL 51000320c showing the deviant tooth crown pattern of the M3.

Skull MHNL 51000321 *Papio* sp.; not in Lortet & Gaillard

The basal part of the hind skull is missing and there has been considerable post-mortem tooth loss. In the right maxilla, the P4 and M1 are in place and the M2 is visible in its crypt. On the left side, only the M2 is present in its crypt. Based on the dental stage, this individual is estimated to be around 4 years of age Evidence for pathologies is lacking. In lateral view the skull resembles *P. anubis* but because of the young age of the individual we retain identification only at the genus level.

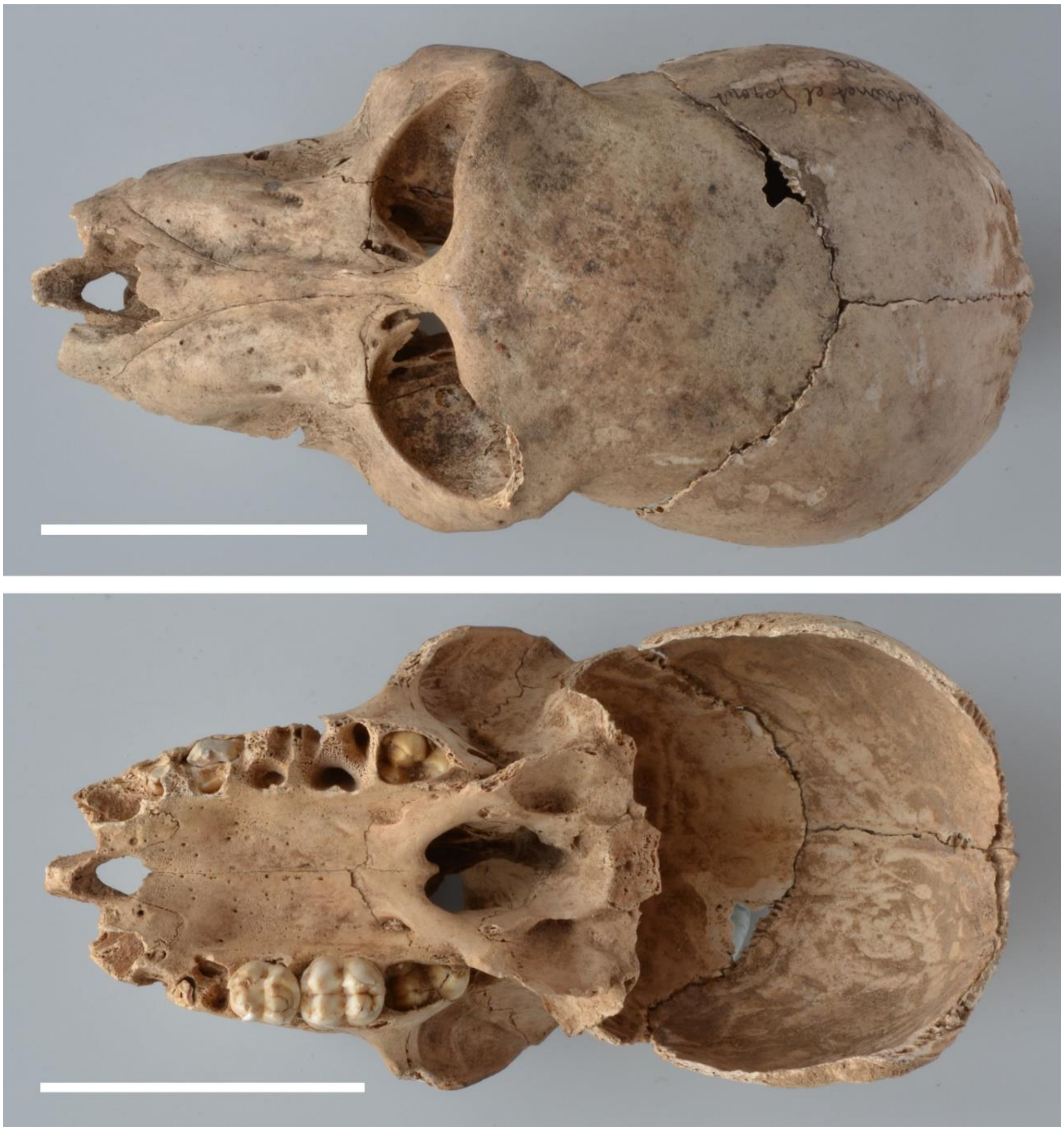

Figure SI-71: Dorsal and ventral view of the skull of *Papio* sp. MHNL 51000321. Scale bars are 5 cm.

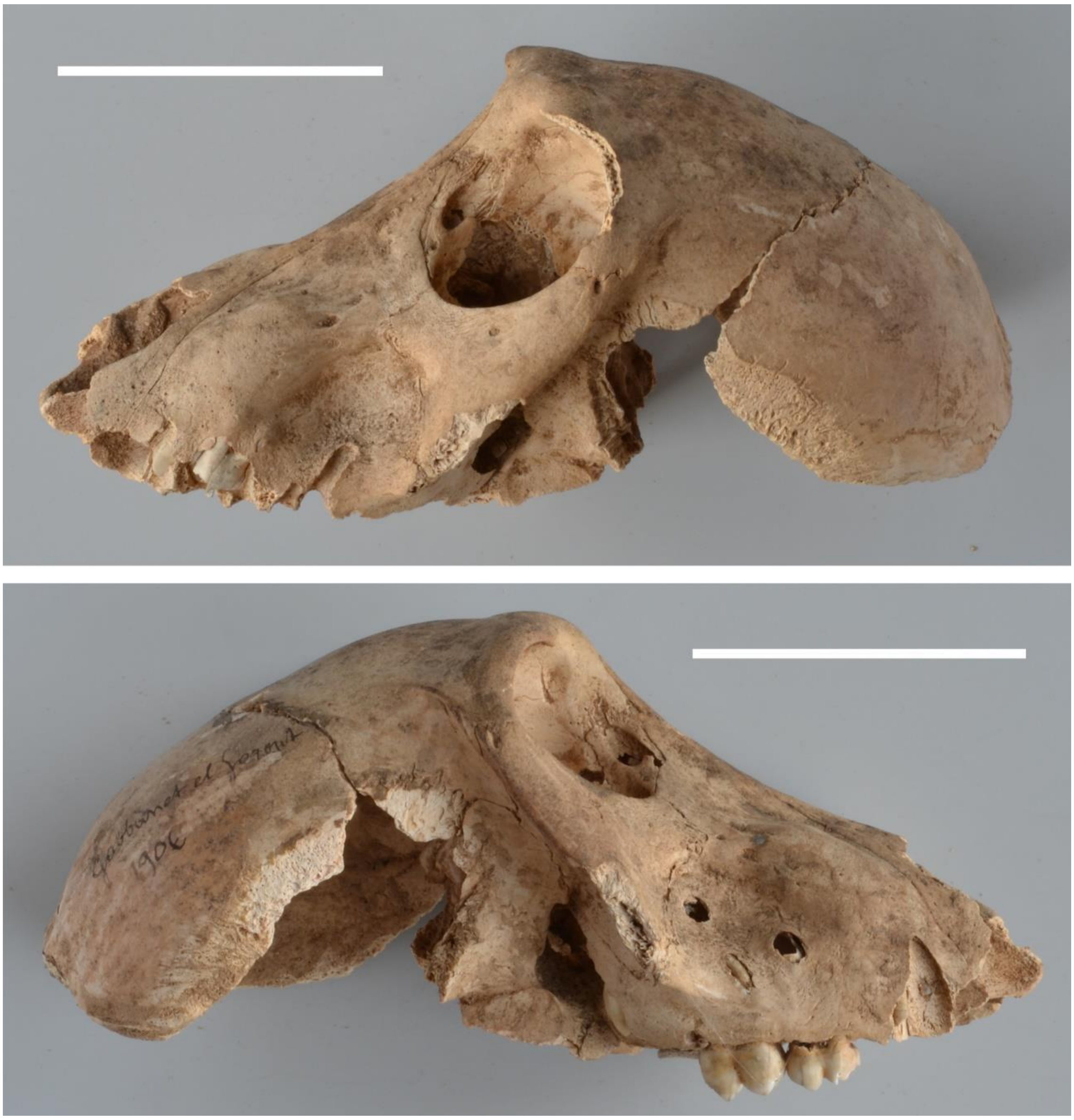

Figure SI-72: Left and right lateral view of the skull of *Papio* sp. MHNL 51000321. Scale bars are 5 cm.

Skull MHNL 51000323A *Papio* sp. male; not in Lortet & Gaillard

Of this specimen, only the skull roof is preserved. The lineae temporales converge towards the posterior part of the skull and they only form thin lines that are not protruding laterally. A rugose surface is noted on both parietals but only over a limited area towards the occipital part of the skull.

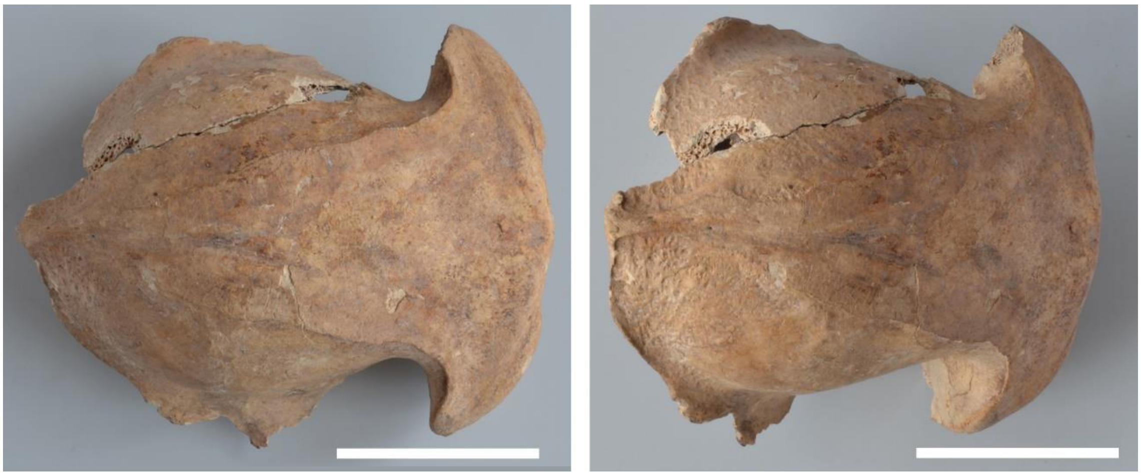

Figure SI-74: Dorsal and dorso-posterior view of hind skull of *Papio* sp. male MHNL 51000323A. Scale bars are 5 cm.

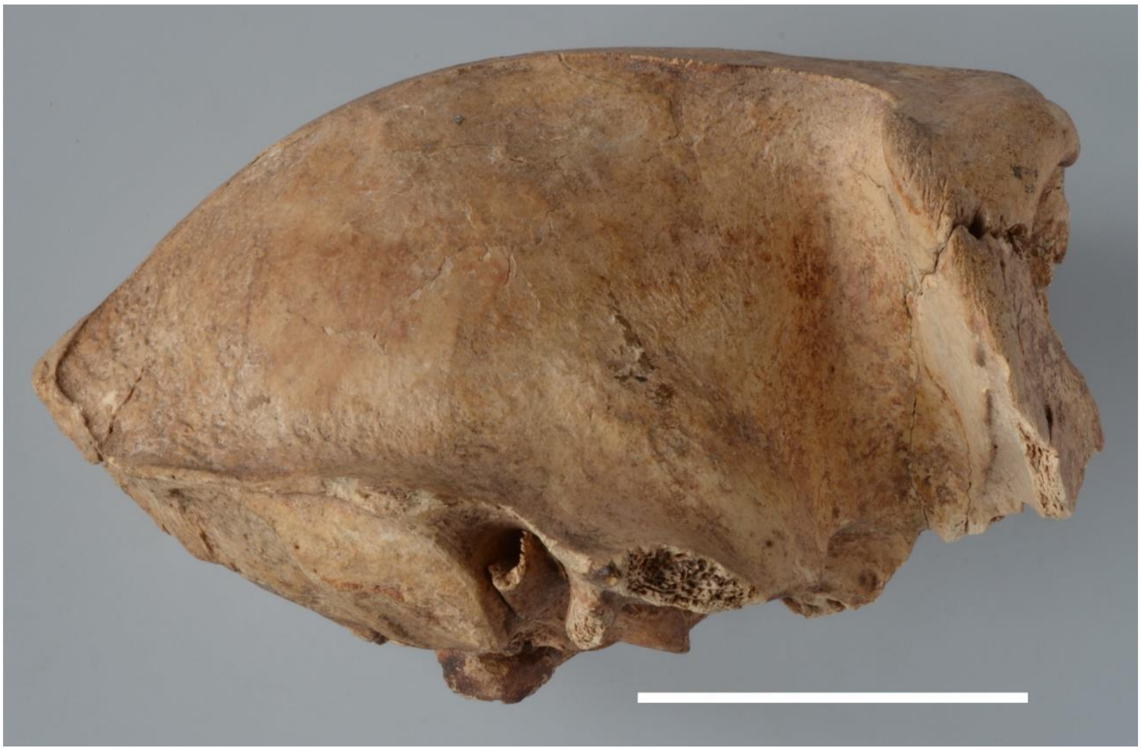

Figure SI-74: Right lateral view of hind skull of *Papio* sp. male MHNL 51000323A. Scale bar is 5 cm.

Skull MHNL 90002100A *Papio anubis* female; Lortet & Gaillard # 11

Apart from limited damage to the left jugal arch and some post-mortem tooth loss, the skull of this individual is intact. The following teeth are still present: right P4-M2; left P3 (partial), P4 and M1. The vertical rami of the mandibles are broken off. In the right half only the I2 is still present, in the left mandible the P3-M3 are in place.

This skull has a metopic suture at the level of the glabella. The posterior end of the skull is slightly rugose.

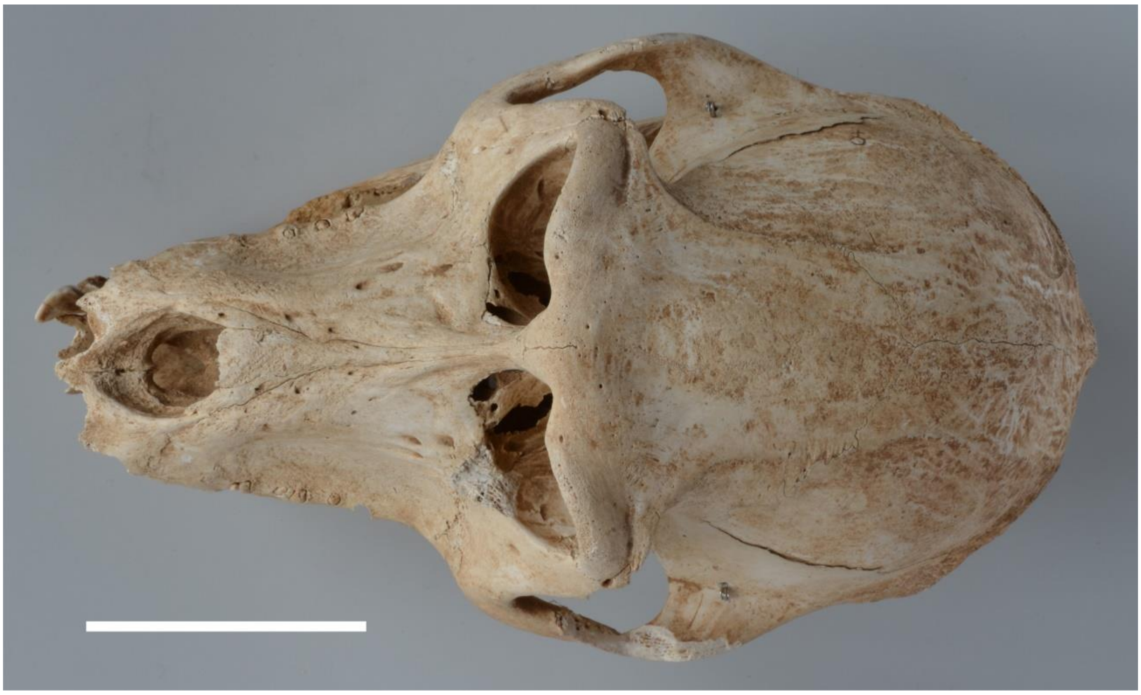

Figure SI-75: Dorsal view of the skull of *P. anubis* female MHNL 90002100A, showing the metopic suture in the glabella and the slightly rugose surface of the parietals. Scale bar is 5 cm.

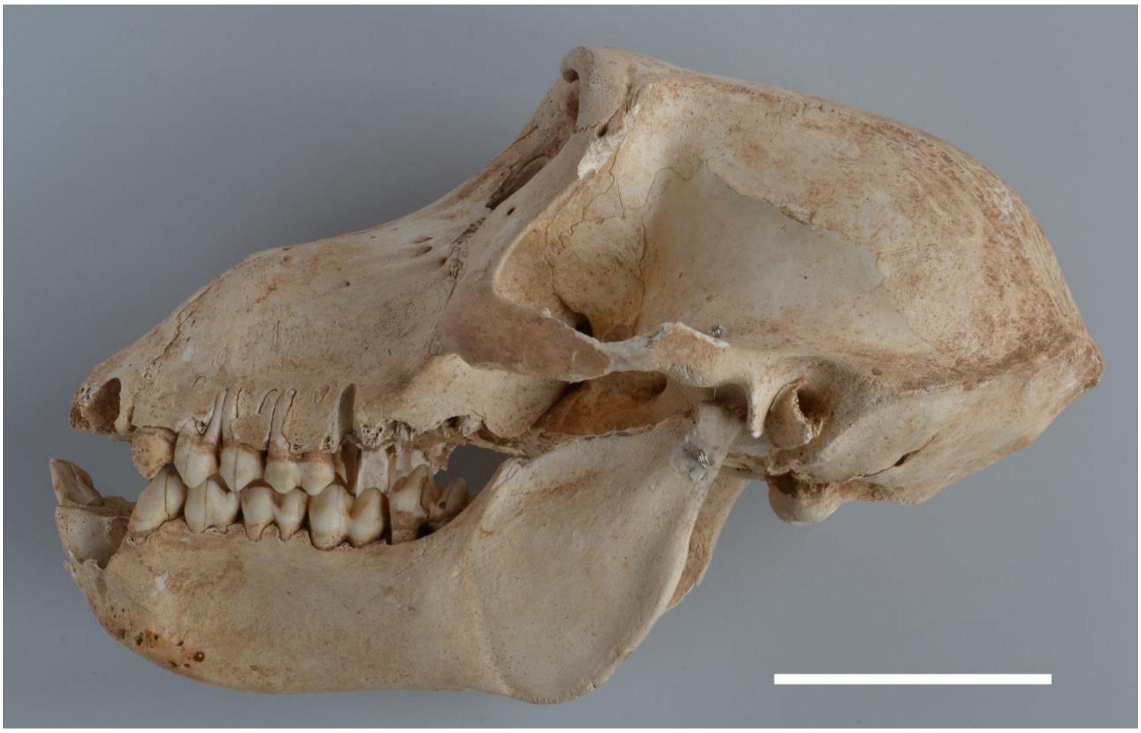

Figure SI-76: Left lateral view of the skull of *P. anubis* female MHNL 90002100A, showing the slightly rugose surface of the parietals. Scale bar is 5 cm.

Mandible MHNL 51000315 + 504 *Papio* sp. male; not in Lortet & Gaillard

Besides the 12 mandibles described above with their respective skulls, the Lyon collections also contain an additional 16 isolated lower jaws in variable state of preservation. In only 12 cases, measurements could be taken (see S4 Table, supplementary information). Here we describe the sole specimen that shows a pathology.

This mandible is well preserved with all the teeth still in situ except for the incisors, the canines and the right P3. The last erupted molar is the M2, the M3 are visible in their crypts. The elongated P3 and the large alveoli for the canines show that we are dealing with a male individual. In front of the alveoli for the canines, there seem to be only two large alveoli for the incisors instead of the expected four. However, two minor depressions are seen aborally of the large alveoli, that may have contained the germs of the two other incisors.

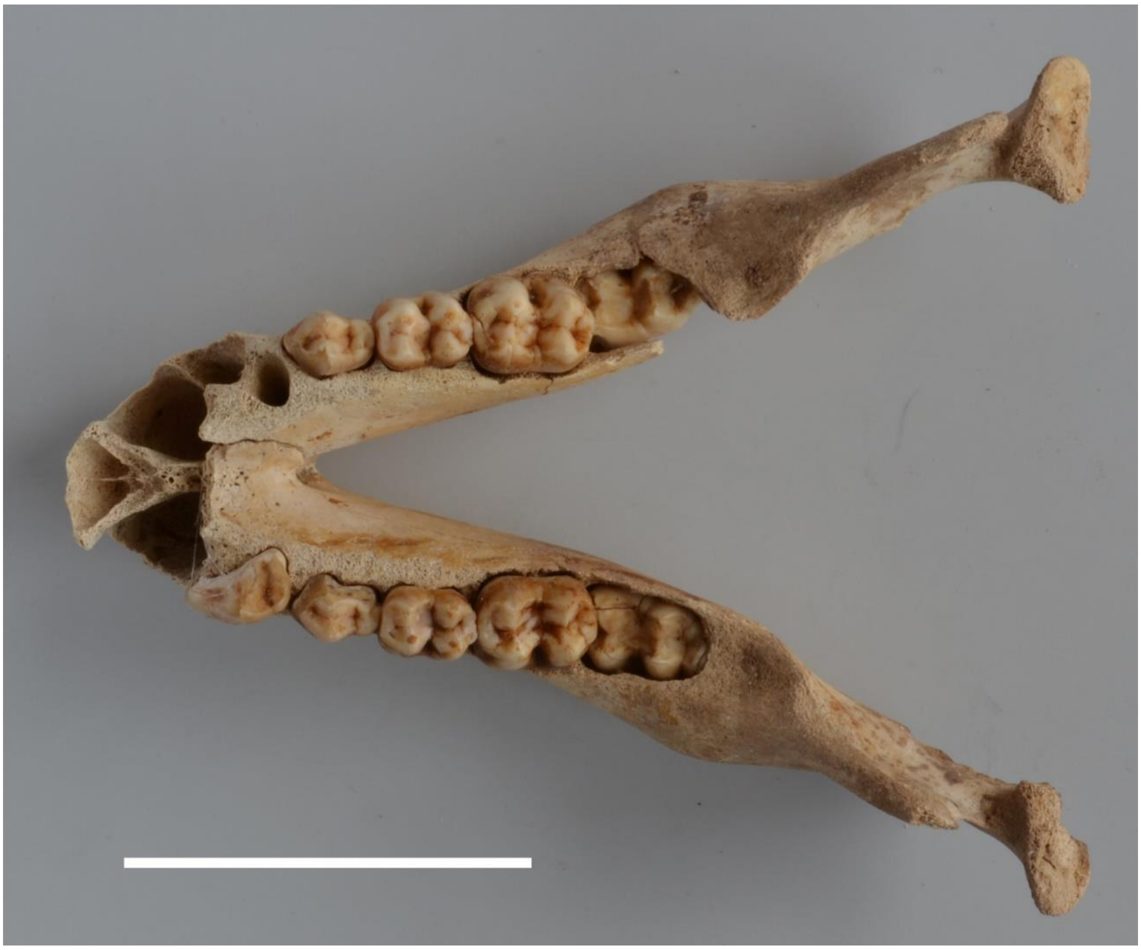

Figure SI-77: Dorsal view of the mandible of *Papio* sp. male MHNL 51000315 + 504. Scale bar is 5 cm.

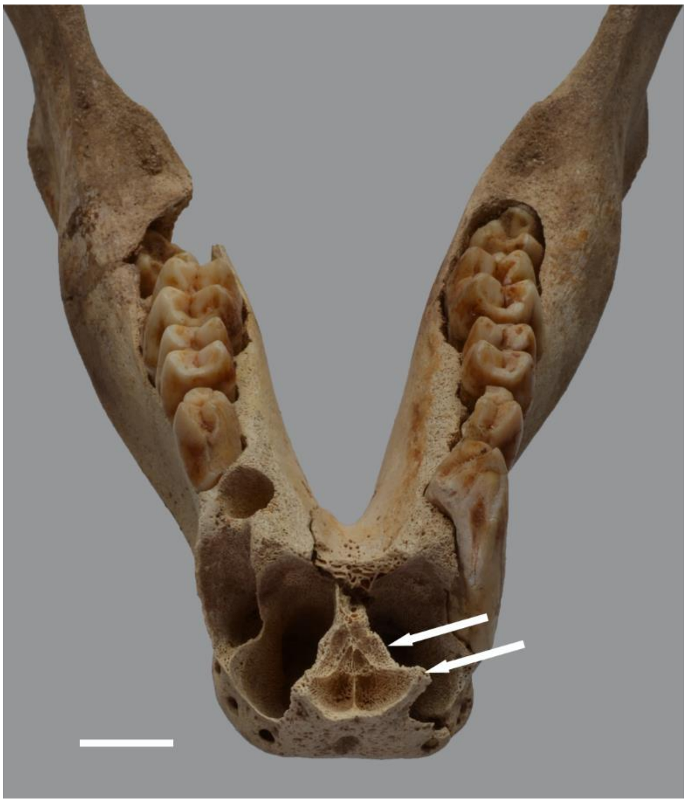

Figure SI-78: Dorso-anterior view of the mandible of *Papio* sp. male MHNL 51000315 + 504, showing the presence of two large and two small alveoli for the incisors. Scale bar is 1 cm.

2. Skeletons

Skeleton MHNL 51000172 *Papio hamadryas* female; Lortet & Gaillard skeleton # 1

An almost complete skeleton is available that was labeled as ‘squelette n°1’ by Lortet and Gaillard (1907: 15) and of which we can confirm that the atlas fits perfectly with the skull n°1 that was later on given the same register number MHNL 51000172. It also appears that the preservation state of the skull and the skeleton are the sarme, making it very likely that both belong to the same individual. Among the postcranial remains, that were used to make a mounted specimen (Figure SI-79), it was noticed that the left scapula was from another individual. The measurements of that bone are therefore not given in the supplementary information. No particular pathologies were observed on the postcranial remains. The only striking features that were noticed on this skeleton are the heavy muscular attachments seen on the shafts of the humeri and, to a lesser extent, on the tibiae.

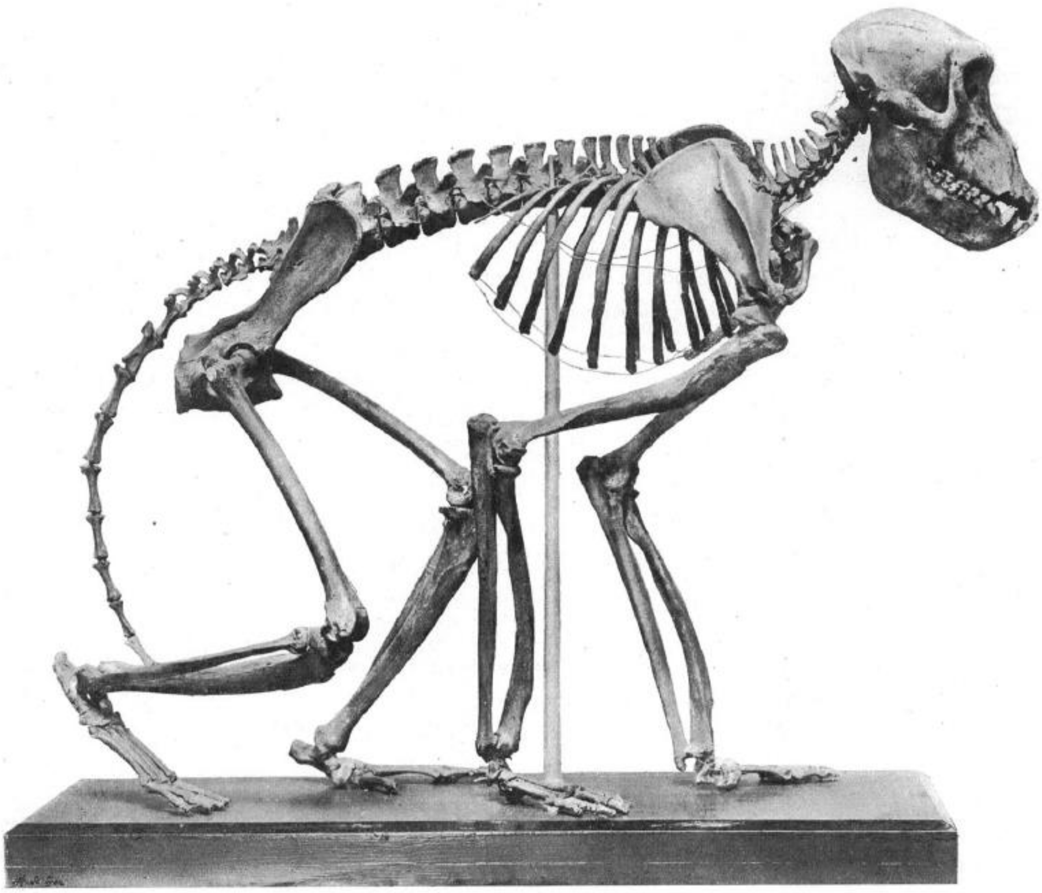

Fig. 83. — *Papio hamadryas*. Vallée des singes, à Thèbes. (1/4 gr. nat.).

Figure SI-79: The sole articulated baboon skeleton figured in Lortet and Gaillard (1907: 14), corresponding to the present-day register number MHNL 51000172.

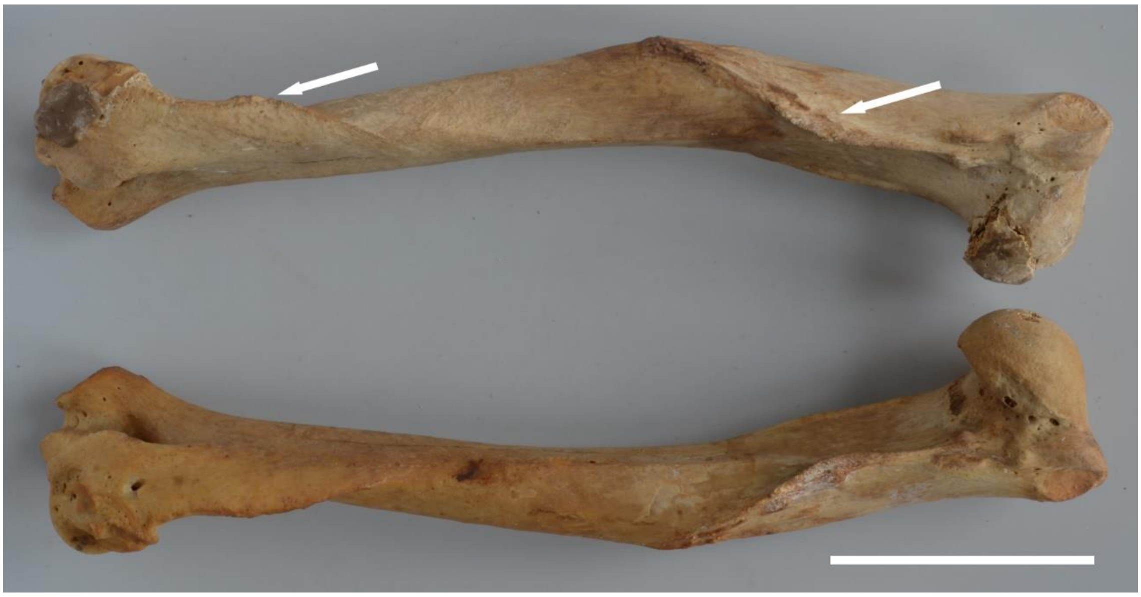

Figure SI-80: Medial view of the left (upper) and right (bottom) humerus of *P. hamadryas* female MHNL 51000172, showing the pronounced crista m. deltoidea and crista epicondyli lateralis. Scale bar is 5 cm.

Skeleton MHNL 51000173 *Papio* sp. male; Lortet & Gaillard skeleton # 8

Another almost complete skeleton is registered at the Musée des Confluences under the same number MHNL 51000173 as a skull of a male *Papio anubis* labeled as skull n°8 by Lortet and Gaillard (1907: 22 and 27). They also mention a skeleton n°8 that is said to belong to a male *Papio anubis* other than that corresponding to the skull n°8. We checked both the skeleton and skull with number MHNL 51000173 and can confirm - judging from their state of preservation - that they clearly represent two different individuals. However, we are less confident in attributing a species and sex identification to the skeleton. Possibly Lortet and Gaillard had cranial remains of this individual at their disposal, which allowed them to label the skeleton as male *P. anubis*. However, this cannot be verified since, as mentioned in the introduction, their notes are no longer available. Our examination of the skeleton, in particular the state of preservation of the bones and the consistency of the observed pathologies, allow us, however, to be confident that all the postcranial remains belong to a single individual. Regarding the sex of the specimen, there are indications indeed that this may have been a male individual despite its relatively small size. The comparably large ala ossis ilii (the ilium wings) compared to their homologue in the female skeleton #1 supports such classification as well.

Scapulo-humeral joint

The articular surface of the scapula is irregular and pitted on about one third of the glenoid cavity and at the base of the acromion. In both humeri, the head is tilted downwards. Around the humeral head, numerous exostoses have resulted in the formation of a circular bony margin. The bicipital groove is rather deep and the crista of the tuberculum major is heavily developed. Both humeri show a slight bending of the shaft. The distal ends do not show obvious deformations.

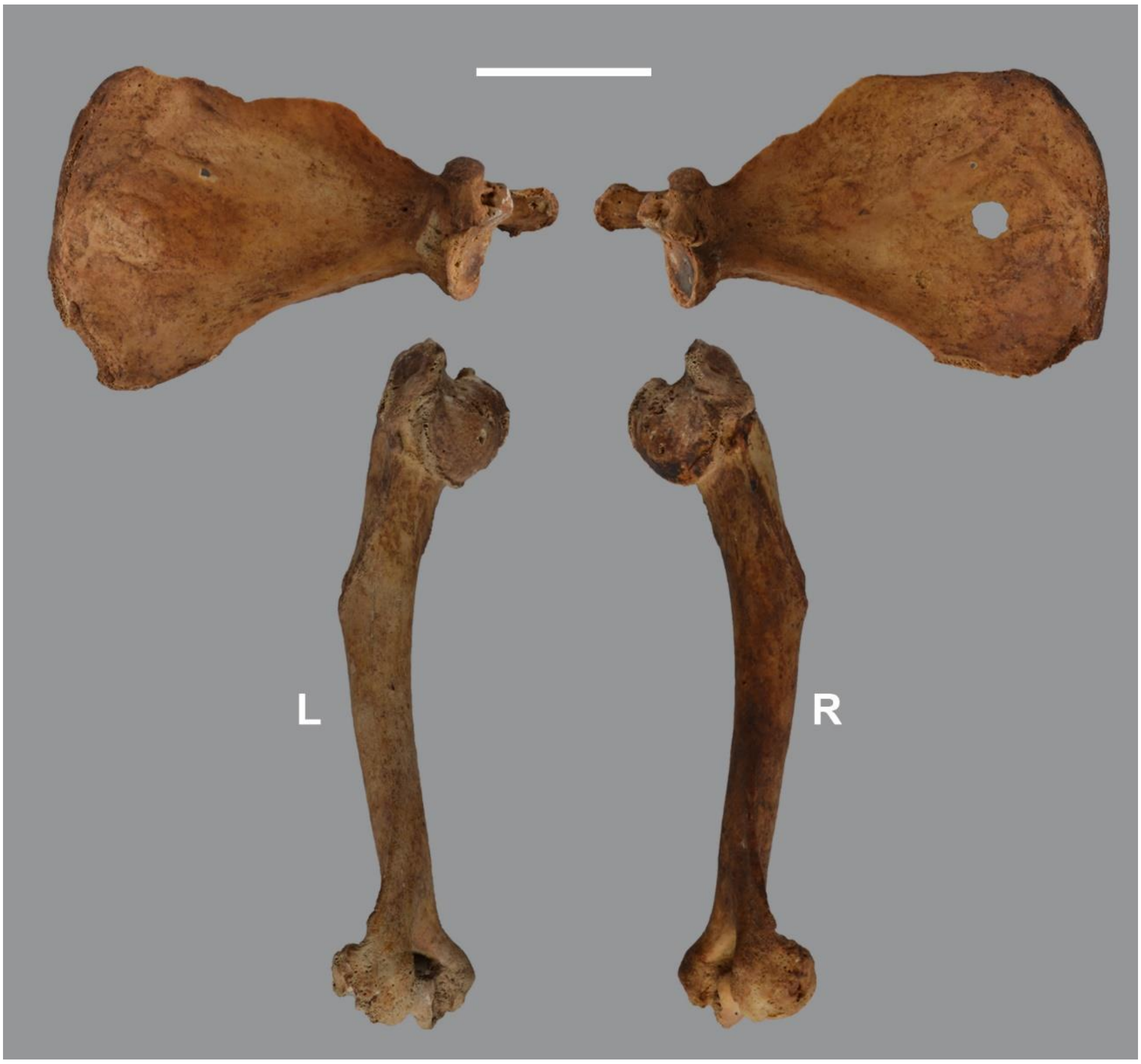

Figure SI-81: The left and right scapula and humerus of *Papio* sp. male MHNL 51000173, showing the slight bending of the humeri and the downward tilting of the humeral heads. Scale bar is 5 cm.

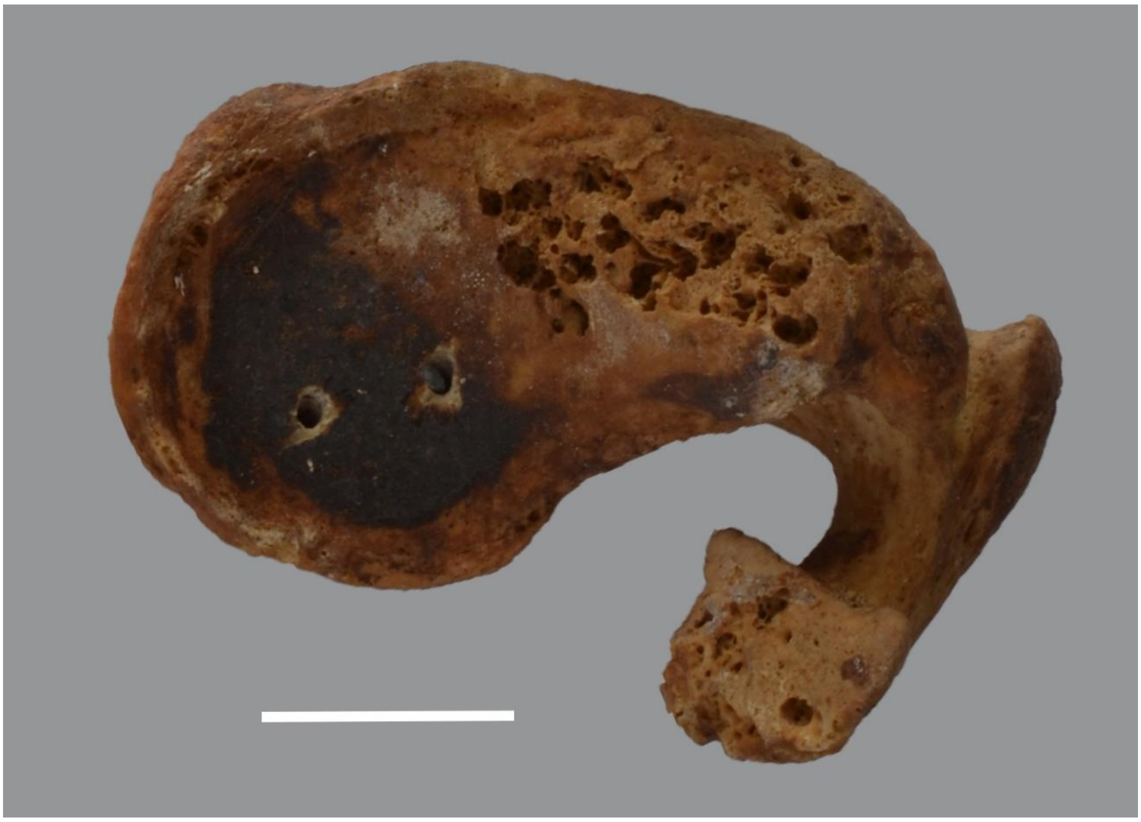

Figure SI-82: Distal view of the right scapula of *Papio* sp. male MHNL 51000173, showing the pitting of glenoid cavity and the base of the acromion. The two holes in the articular surface are artefacts. Scale bar is 1 cm.

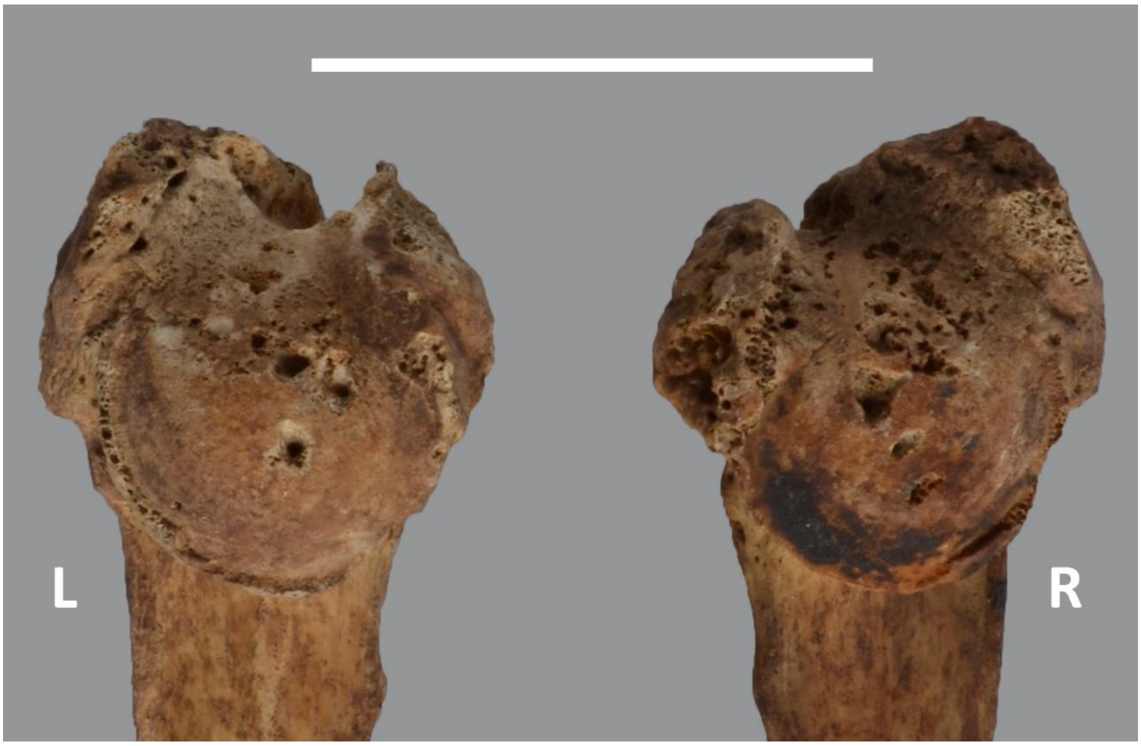

Figure SI-83: Caudal view of the left and right humerus of *Papio* sp. male MHNL 51000173, showing the downward tilted humeral heads as well as their circular bony margins formed by osteophytes. Scale bar is 5 cm.

Femur

The proximal ends of the femurs are asymmetrical. It appears that the sulcus of the right femur is more open than that of its left homologue. In addition, the collum of the left femur is more sturdy and shows additional bone tissue at its surface. The transition of the column towards the femoral head seems eroded in the left femur and is more sharply delineated in the right specimen.

The distal ends show in both cranial and caudal view a marked deviation of the epiphysis. Both medial condyles are morphologically unaltered, unlike the lateral condyles of both femurs that are surrounded by marginal osteophytes. The trochlea of the right femur is modified by the numerous periarticular exostoses. The left femur exhibits loss of compacta with pitting on the medio-cranial edge of the patellar groove and a line at the articular surface pointing to a tilting of the main axis of the patella.

In caudal view, one observes that the epicondylus medialis is more or less normally developed, whereas the epicondylus lateralis has an irregular surface in both the left and right femur. This bone proliferation is a result of osteophyte formation to secure functioning of the knee joint.

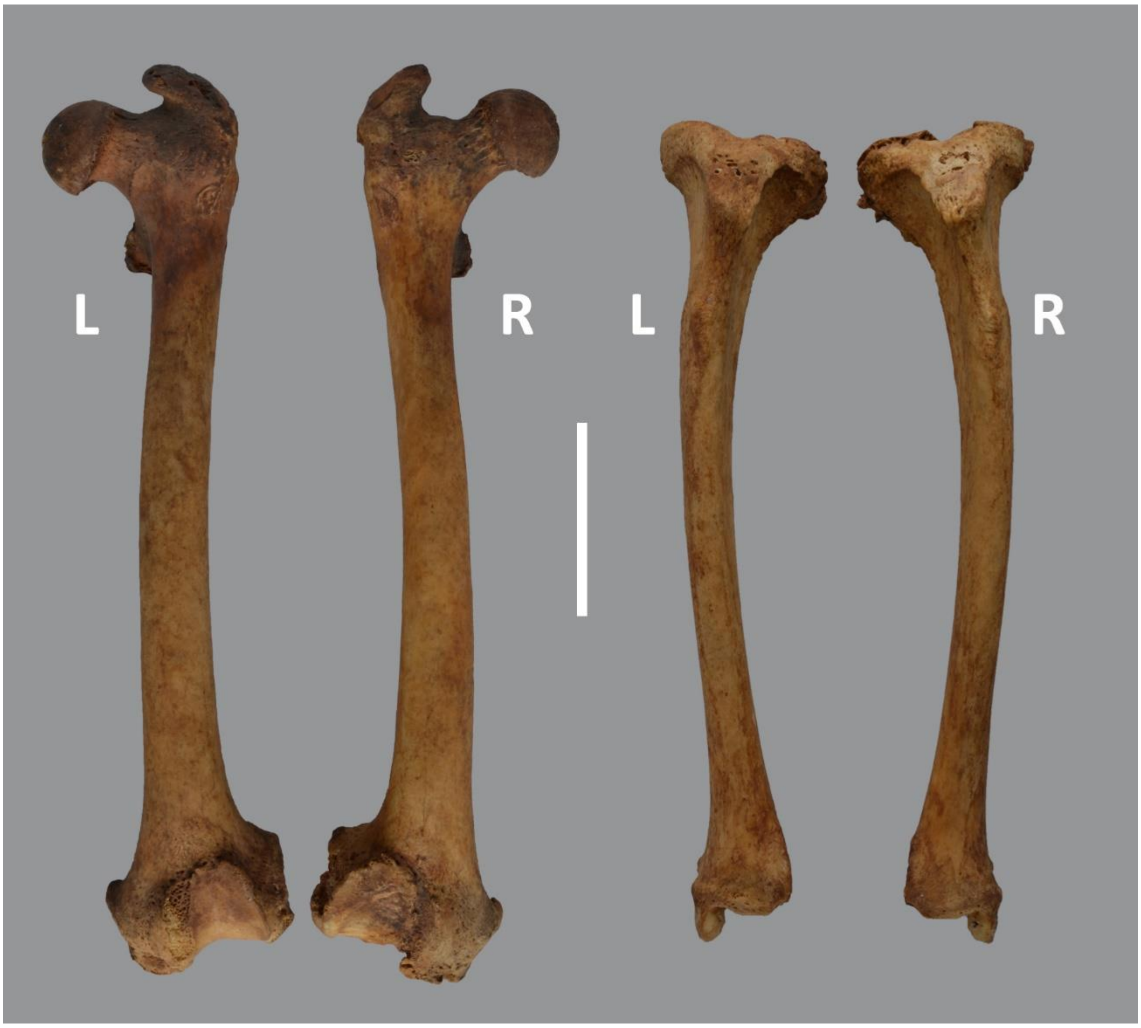

Figure SI-84: Cranial view of the left (L) and right (R) femur and dorsal view of the tibia left (L) and right (R) of *Papio* sp. male MHNL 51000173, showing the deviation of the distal epiphyses. Scale bar is 5 cm.

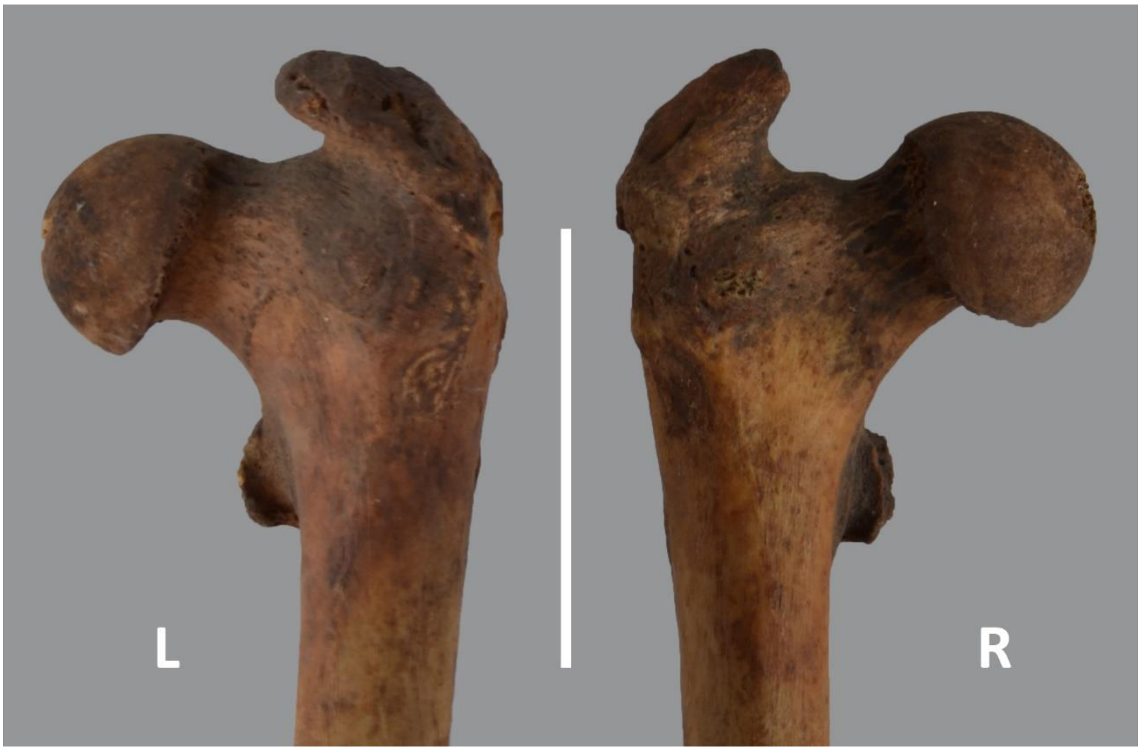

Figure SI-85: Cranial view of the proximal part of the left (L) and right (R) femur of *Papio* sp. male MHNL 51000173, showing differences in the sulcus, in the development of the collum, and the presence of additional bone tissue in the left bone. Scale bar is 5 cm.

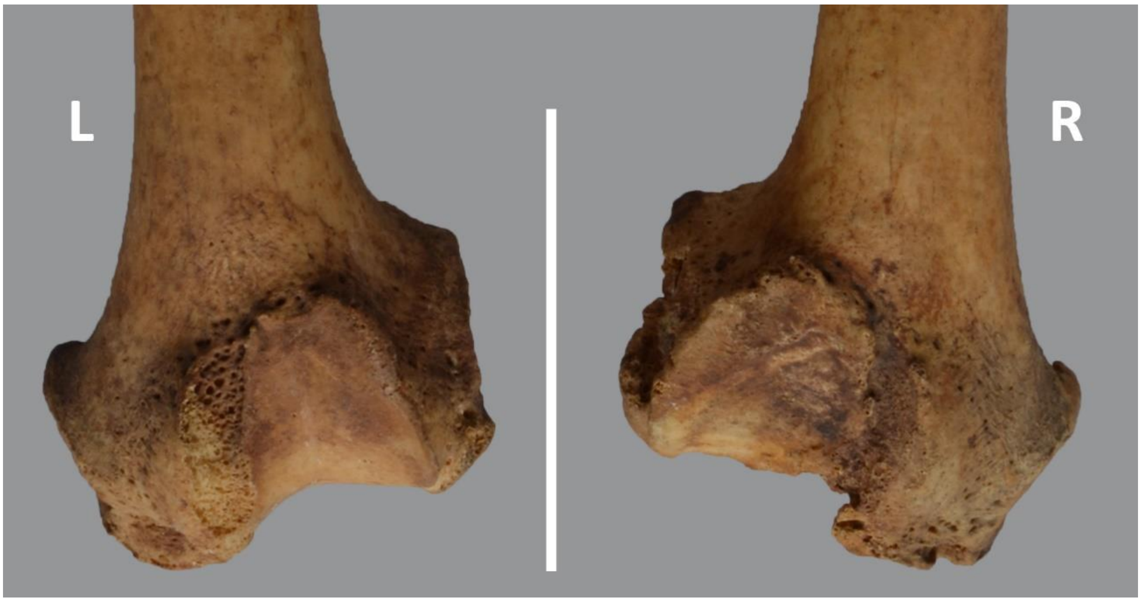

Figure SI-86: Cranial view of the distal part of the left (L) and right (R) femur of *Papio* sp. male MHNL 51000173, showing the presence of marginal osteophytes on the lateral condyles, and deformations at the level of the trochleae. Scale bar is 5 cm.

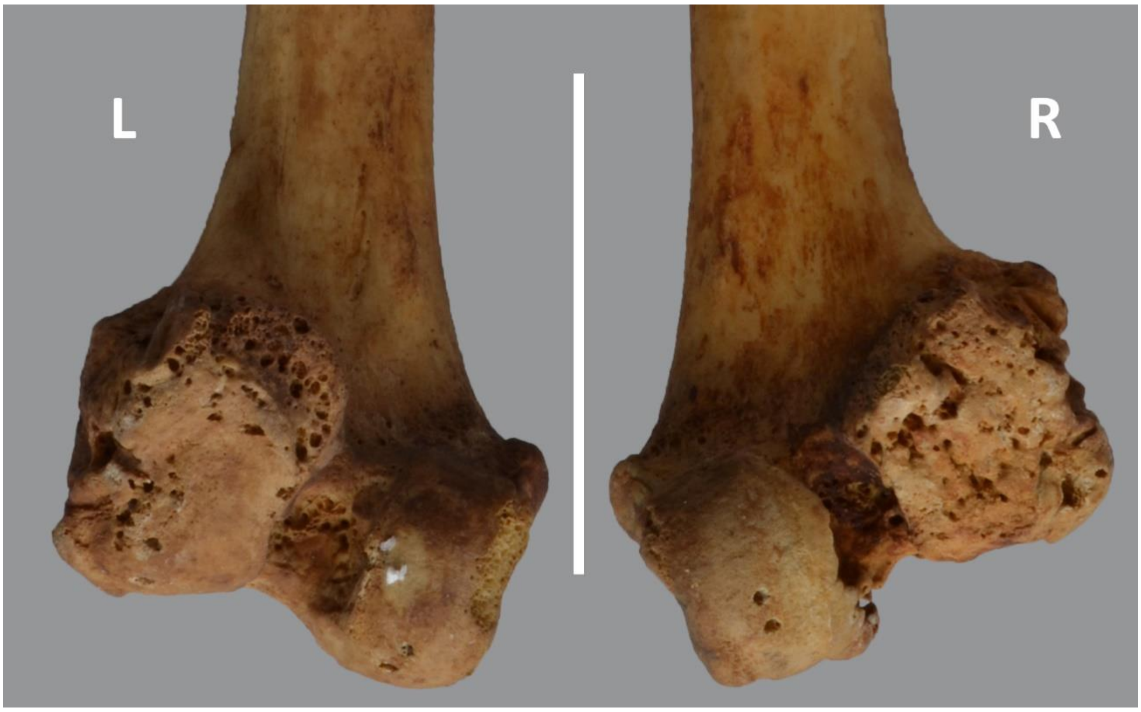

Figure SI-87: Caudal view of the distal part of the left (L) and right (R) femur of the *Papio* sp. male MHNL 51000173, showing the irregular surface of the lateral epicondyles resulting from osteophyte formation. Scale bar is 5 cm.

Tibia and fibula

The shafts of the tibiae and the sole preserved fibula are slightly bend. Around the two proximal articulations, in particular of the right tibia, numerous osteophytes and lipping are observed. In addition, the articular surfaces themselves have a cribriform aspect. The proximal end of the right fibula is in the process of fusing to the tibia as shown by the bony spurs. The left fibula is missing, but from the proximal tibia, it can be seen that there was no fusion on that side.

The erosive and reactive new bone formation observed in the entire articular surface of the distal femur and the proximal tibia as well as the large disruption of the cortical margins of the proximal surface of the tibia are a result of non-axial load due to articular misalignment.

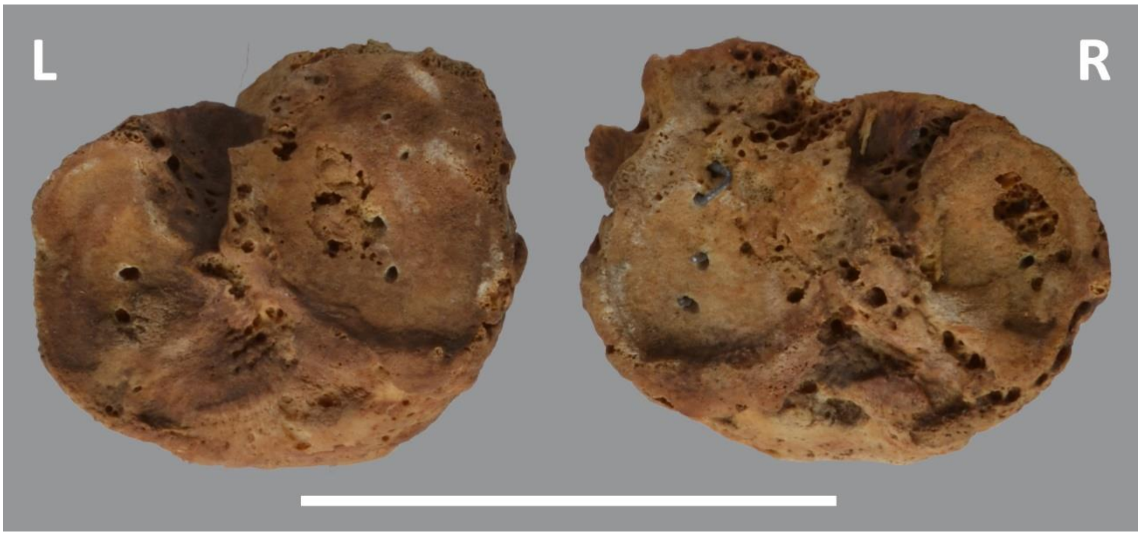

Figure SI-88: Proximal view of the tibiae of *Papio* sp. male MHNL 51000173, showing osteophyte formation and lipping, as well as the pitted surface of the articulations. The circular holes in the articular surface are artefacts due to metal wires. Scale bar is 5 cm.

Vertebral column

Pathologies are observed in the lumbar region: on their ventral sides the L2, L3 and L4 are completely fused by bony bridges, whereas at the level of the L1 and the last three thoracic vertebrae, bony spurs occur that possibly correspond to additional bony bridges that may have been damaged after death. At the level of the fourth and third lumbar vertebrae, the bony bridge appears asymmetrical indicating some kind of dislocation between the two vertebrae. Because the column is normal in dorsal view, this ventral fusion should not be considered the result of a trauma at this level.

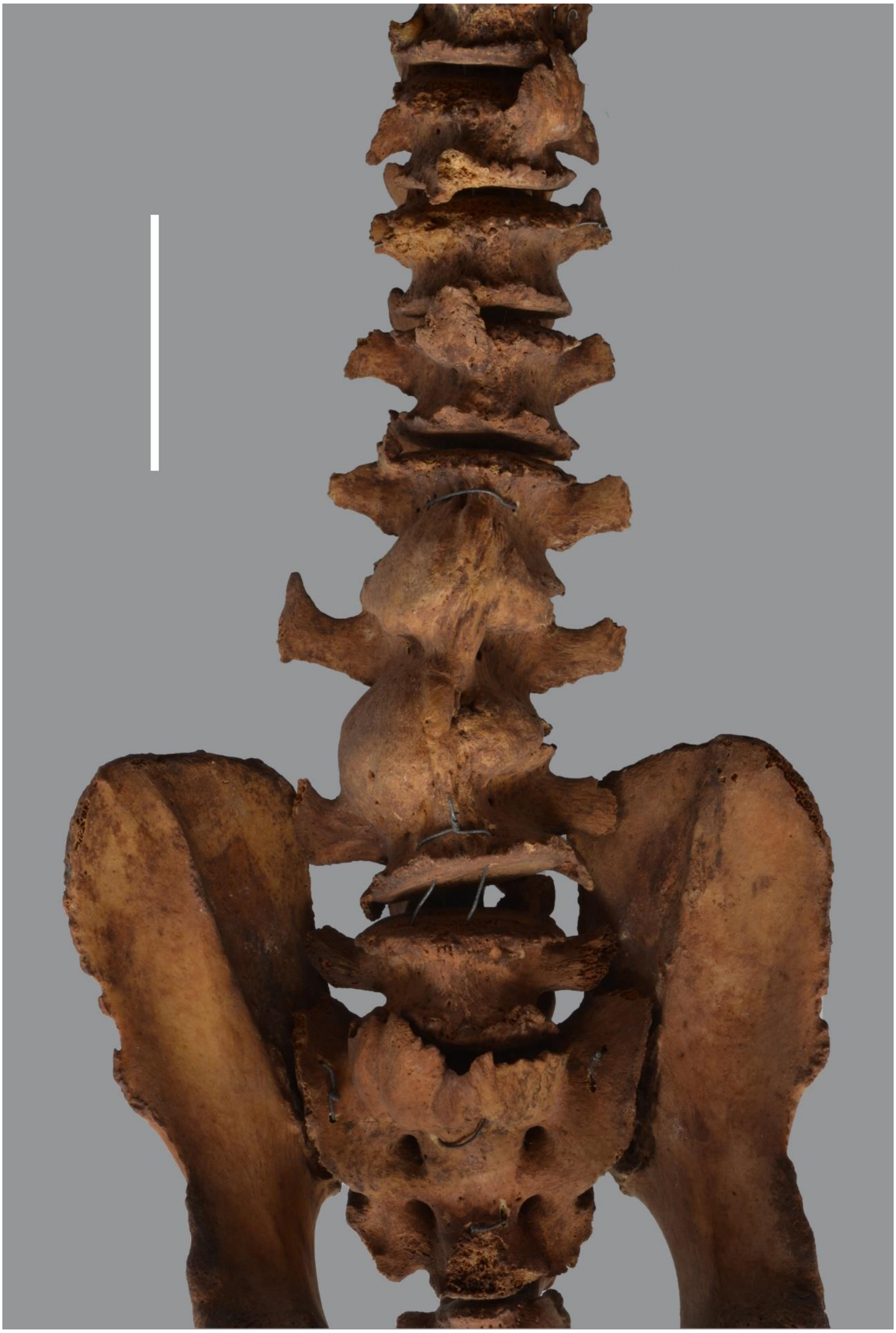

Figure SI-89: Ventral view of the sacrum, the lumbar vertebrae and the three last thoracal vertebrae of *Papio* sp. male MHNL 51000173, showing ankylosing centra. Scale bar is 5 cm.

Skeleton MHNL 51000325 *Papio* sp. male; not in Lortet & Gaillard

A partial skeleton of a large baboon is registered under this number. Because of its sturdiness and the large size of the long bones, they must pertain to a male individual. Also the size and shape of the posterior part of the ischium points in that direction. No vertebral column (except for the sacrum) or skull are associated to these bones. The left and right pelves and the hind legs and feet are present. No scapula and only a right humerus could be found in the collections, while the rest of the arms and hands are complete. Not a single clear pathology has been observed on this assemblage. The only feature that we want to mention here are the heavy ridges for muscular attachments seen on the lateral side of the shaft of the tibiae and on the radius and ulna.

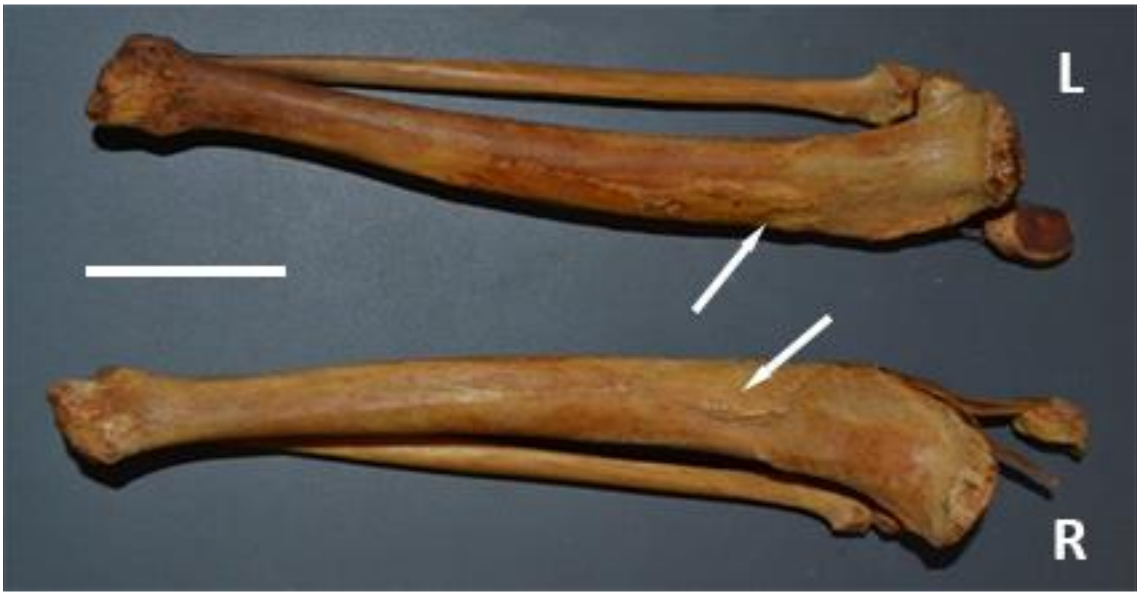

Figure SI-90: Medial view of the left (L) and right (R) tibia, fibula and patella of *Papio* sp. male MHNL 510000325 showing the well-developed attachment sites for the *Musculus tibialis anterior*. Scale bar is 5 cm.

Skeleton MHNL 51000170 *Papio* sp. male; not in Lortet & Gaillard

The collection number MHNL 510000170 refers to the skull of a female *Papio hamadryas* that Lortet and Gaillard labeled as ‘crâne 3’. The same number MHNL 510000170 has also been given to a skeleton. The atlas of this skeleton is too large for the skull of the female *Papio hamadryas* with the same number. We found that the atlas articulates well with several of the male skulls in the collection, but were not able to identify with certainty the species or the individual. For that reason we propose to label the vertebral column of this specimen as ‘male *Papio* sp.’. The shape and size of the posterior part of the ischium also indicate that we are dealing with a male individual. When analysing the extremities, however, we realised that the legs do not fit to the vertebral column and pelves. The femur heads are much too small for the acetabula. The left and right arms are both relatively small and roughly belong to the same size class as the legs, but it is clear that the left and right side are composed of bones from two different individuals, as can be seen from the measurements that we have nevertheless retained for information in the supplementary tables (Table S2). Worth mentioning is that these two individuals, used to compose a single skeleton, do not show any pathologies except for a slight exostosis at the level of the trochanter major of the right femur.

Skeleton MHNL 51000171 *Papio* sp. male; not in Lortet & Gaillard

The Lyon collection number MHNL 51000171 was given to a skull of *Papio hamadryas* – described above – to which apparently no number was attributed by Lortet and Gaillard but of which the old label mentions “appartient à un squ momifié”.

The same number MHNL 51000171 was also given to what looks at first sight as an apparently rather complete skeleton. The atlas of this animal was missing making it impossible to verify if it would match the skull with the same number. We are therefore not sure if the skeleton and the skull represent a single individual. The ischial tuberosities are large suggesting that the pelvis and vertebral column are from a male individual. However, it appears clearly that the skeleton labelled as MHNL 51000171 is composed of the bones of several individuals.

The left and right scapula and humerus are pathological and, judging from their morphology and size, clearly belong to the same animal. In the case of the radius and ulna, the dimensions of the left and right bones are rather similar, but those from the left do not show any of the pathological deformations seen in the right bones. It appears also that the state of preservation of left and right lower arms is different. The lower extremities do not seem to belong to a single individual either: the left femur, tibia and fibula are not pathological and they are also much larger than the corresponding – pathological – bones of the right side. Moreover, it will be shown below that the dimensions of the pathological right femur cannot be of the same animal as the right radius and tibia. It is therefore described separately – after the skeletons – as was done for a number of other isolated bones that are pathological. Our reanalysis thus allows concluding that at least three individuals were used to compose the mounted skeleton, registered under number MHNL 51000171. Except for the scapula and humerus, none of the left bones – which show no pathologies – are therefore not considered in the descriptions below. Nevertheless, their measurements have been retained in the supplementary information (Table S2). The vertebral column and the pelvis are not retained either in the following description, since there is no corresponding femur allowing us to verify whether they belong to this animal.

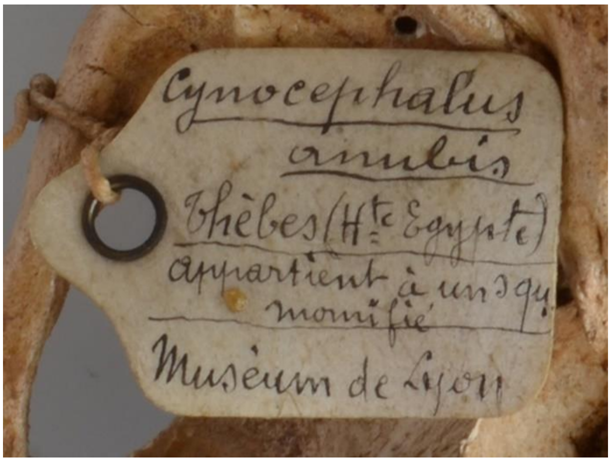

Figure SI-91: Old label attached to skull number MHNL 51000171 that we re-identified as *Papio hamadryas*.

Shoulder girdle and upper arm

Heavy deformations are observed at the level of the articulation of the scapulae and the proximal humeri and at the level of the articulation of the scapulae with the claviculae. Both the left and right scapula show heavy muscular ridges. On the glenoid surfaces and on their margins, osteophytes are observed, resulting in a rugose surface of the articulations. The articulation of the acromion with the clavicula shows periarticular osteophytes. Peri- and intra-articular osteophyte formation strongly suggest that the scapula and its coracoid process articulated loosely with the humerus and coracoid respectively. The humeral heads are collapsed and directed downward, exhibiting osteophytes at the level of the articulation and around their margins.

The diaphyses of both left and right humerus are bowed and most of the cortical bone surface of the humerus shafts is reticular. In the proximal third, the muscular attachments are very pronounced, especially for the musculus deltoideus, which was extended due to the strain exerted by the tilting of the long bone axis. The distal parts of the humeri appear unaltered, but a heavily developed lateral epicondyle crest with few osteophytes was noticed. The medial epicondyle exhibits osteophyte formation in the attachments of the ligaments. However, no deviation is observed of the longitudinal axis of the articulation with the radius and ulna.

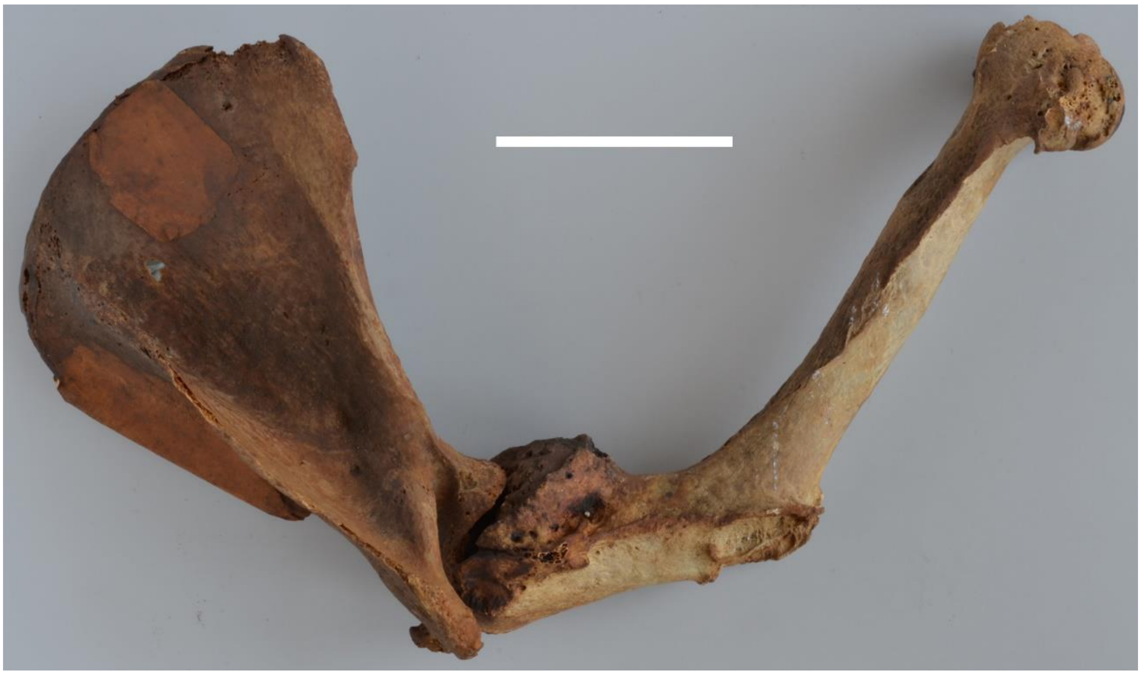

Figure SI-92: Medial view of the left scapula and humerus of *Papio* sp. male MHNL 51000171, showing the bowed aspect of the humeral shaft and the strong muscular insertions. Scale bar is 5 cm.

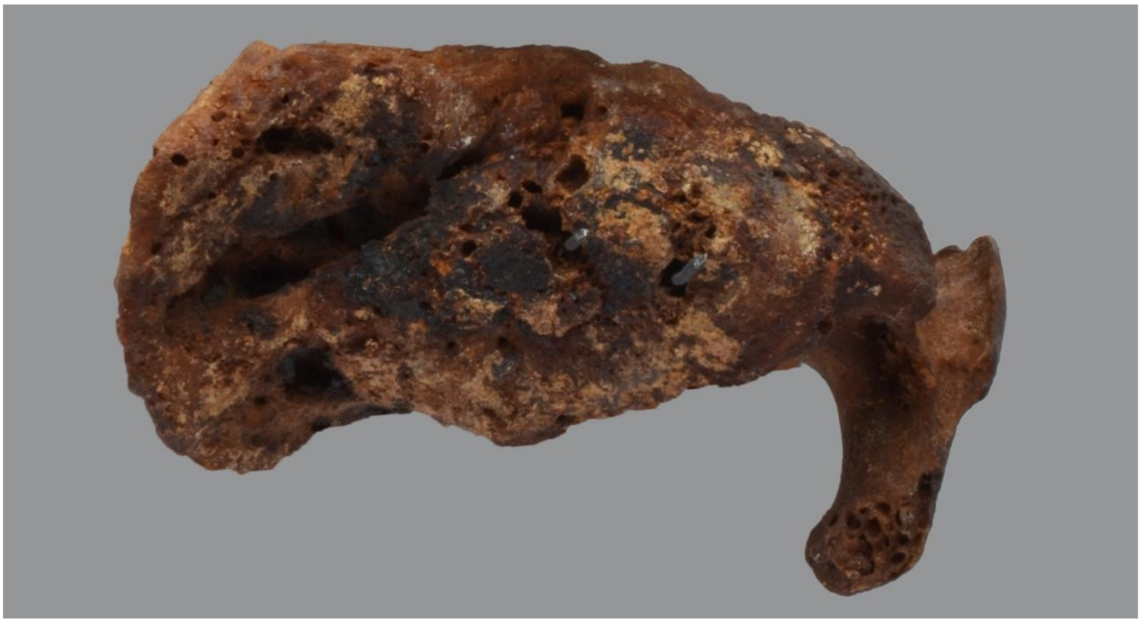

Figure SI-93: The glenoid surface and of the acromion of the right scapula of *Papio* sp. male MHNL 51000171, showing periarticular osteophytes and the rugose surface of the articulations.

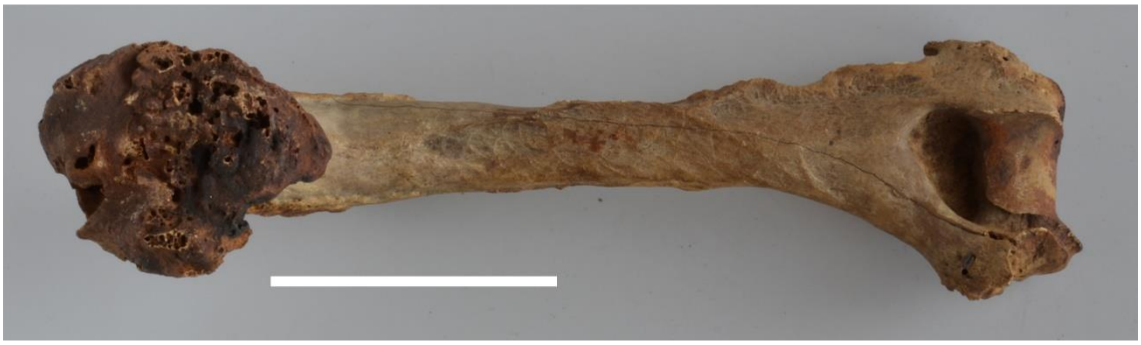

Figure SI-94: Caudal view of the right humerus of *Papio* sp. male MHNL 51000171, showing the head that is collapsed and directed downward. Scale bar is 5 cm.

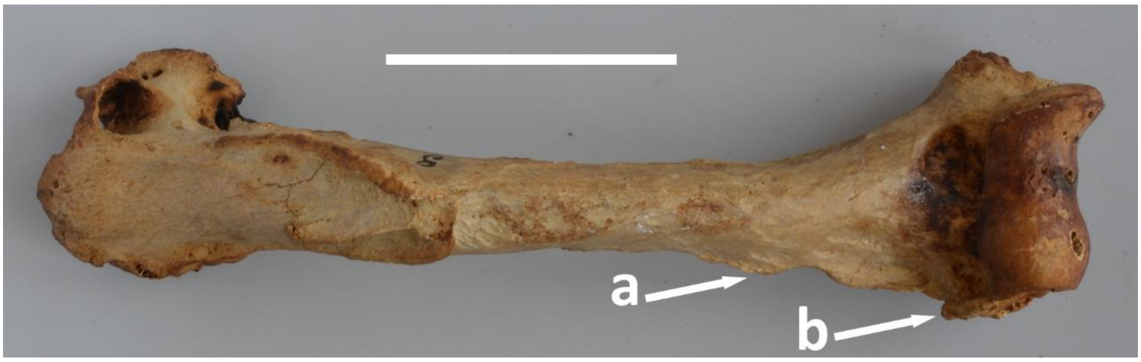

Figure SI-95: Cranial view of the right humerus of *Papio* sp. male MHNL 51000171, showing the thin, sharp-edged crista supracondylaris lateralis (a), the bony extension on the lateral epicondyle (b) and the reticular cortical bone surface. Scale bar is 5 cm.

Radius and ulna

Both the radius and ulna show heavy muscular crests and the shaft of the radius is slightly bowed. Instead of being smooth, the bone surface on both the lateral and the medial side of the ulna head has a reticular appearance on the medial side and with small depressions on the lateral side, occasionally with a sharp margin. All this is again indicating heavy strain at the level of the ligaments of the joint.

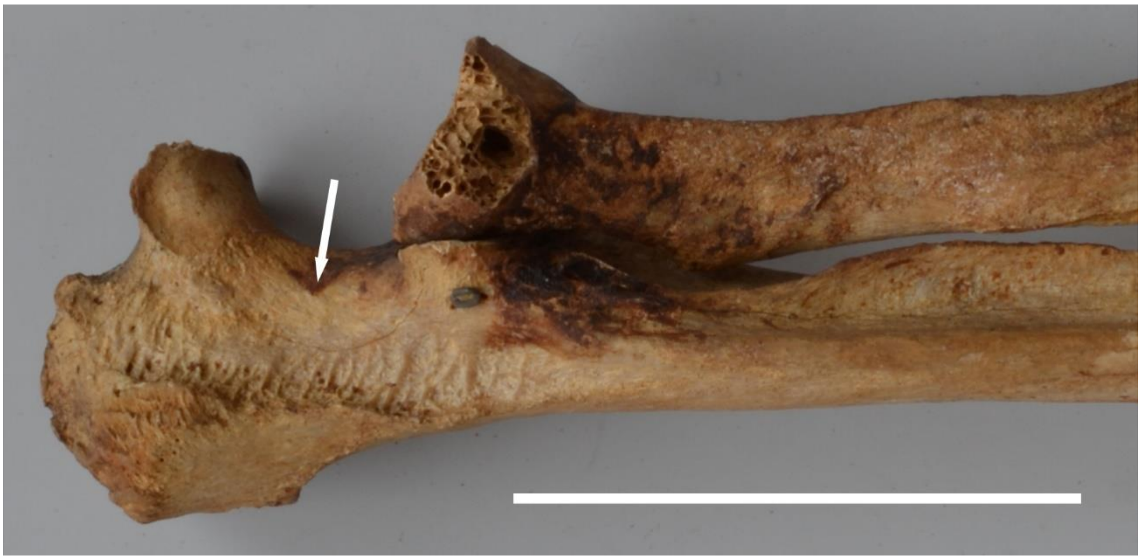

Figure SI-96: Medial view of the right proximal ulna and radius of *Papio* sp. male MHNL 51000171, showing the ulnar head that is reticulated. Scale bar is 5 cm.

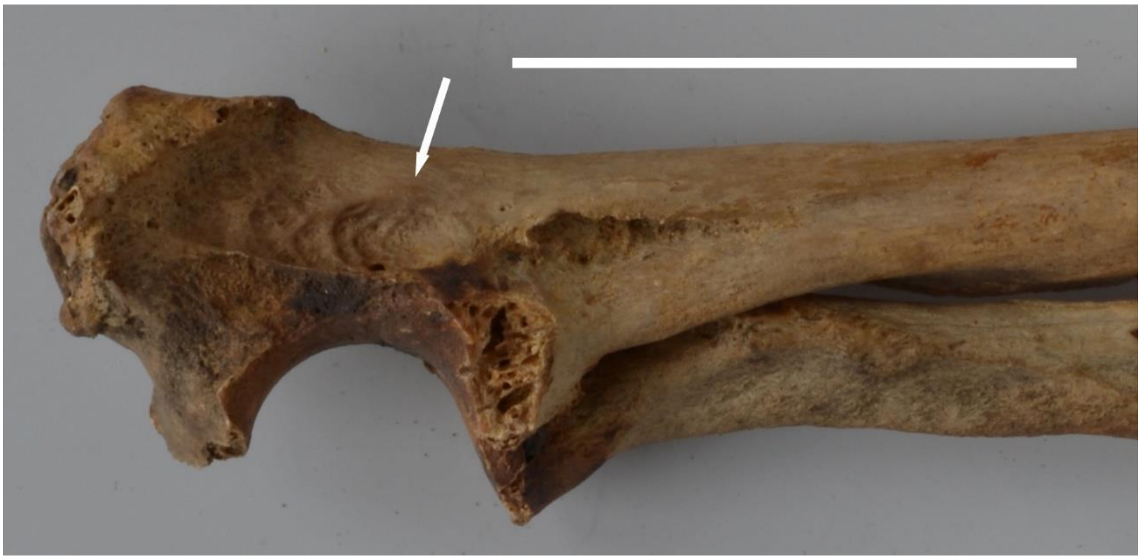

Figure SI-97: Lateral view of the right proximal ulna and radius of *Papio* sp. male MHNL 51000171, showing the ulnar head that exhibits hollows. Scale bar is 5 cm.

Tibia

The overall built of the tibia does not differ from that in healthy individuals. While pathologies are absent distally, the proximal tibia shows periarticular osteophytes surrounding nearly the entire articular surface. The tuberositas crista tibiae is irregular and its surface is pitted.

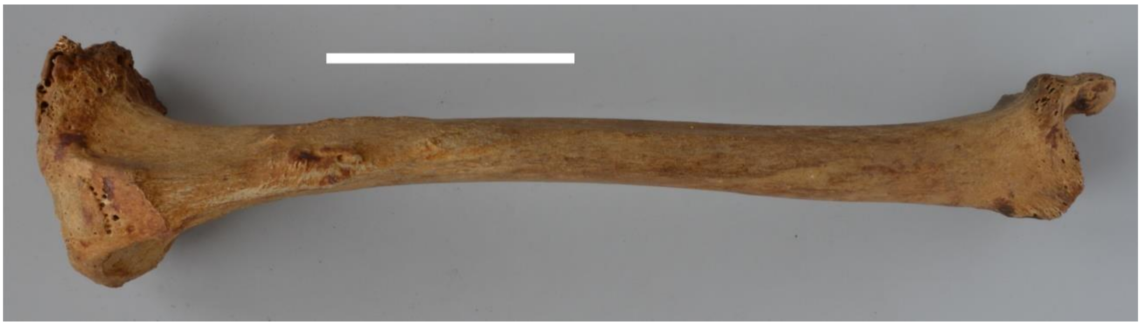

Figure SI-98: Dorsal view of the right tibia of *Papio* sp. male MHNL 51000171. Scale bar is 5 cm.

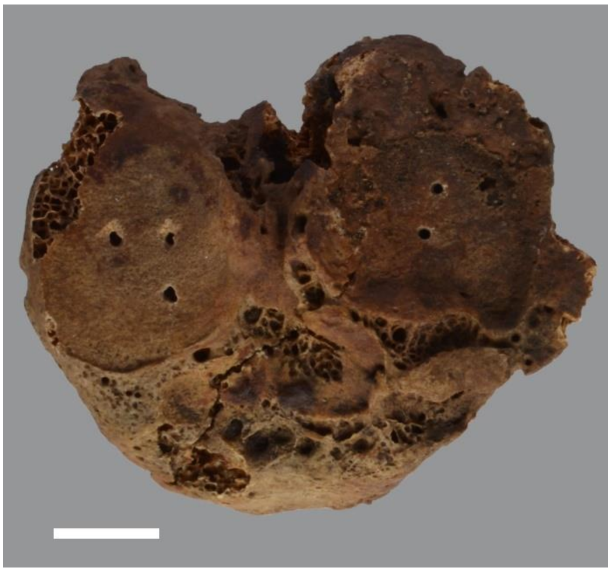

Figure SI-99: View of the proximal articulation of the right tibia of *Papio* sp. male MHNL 51000171. Periarticular osteophytes can be seen on almost the entire articular surface. The circular holes in the two articular facets are artefacts. Scale bar is 1 cm.

Skeleton MHNL 51000328B *Papio* sp. female; not in Lortet & Gaillard

The skeletal remains with this register number consist of the left and right fore- and hindlimbs that seem to belong to a single individual, with the exception of the pelvis that is clearly of a larger, probably male individual. The humeri and femurs are marked with a number "2", in the case of the left femur it reads "n°2 1906". As far as we can tell, these skeletal elements are not mentioned in Lortet and Gaillard (1907). The sole specimen marked as number 2 in their work is a skull of a female *Papio hamadryas*, now registered as MHNL 51000179. The measurements of the skeletal elements of MHNL 51000328B are to some extent comparable to those of the female *Papio hamadryas* MHNL 51000172, which corresponds to skeleton 1 of Lortet and Gaillard (see S2 Table). Maybe the skeletal elements were at some stage believed to belong to the same individual as skull #2, but the authors may have been hesitant to finally retain this conclusion.

The left and right humerus of this individual are heavily bent and the proximal articulations are deformed. The caput has collapsed and is deviated in a downward direction.

The left and right femurs are bent as well. The caput is deformed – especially in the left specimen – and bent downward, whereas the distal articulations seem unaffected.

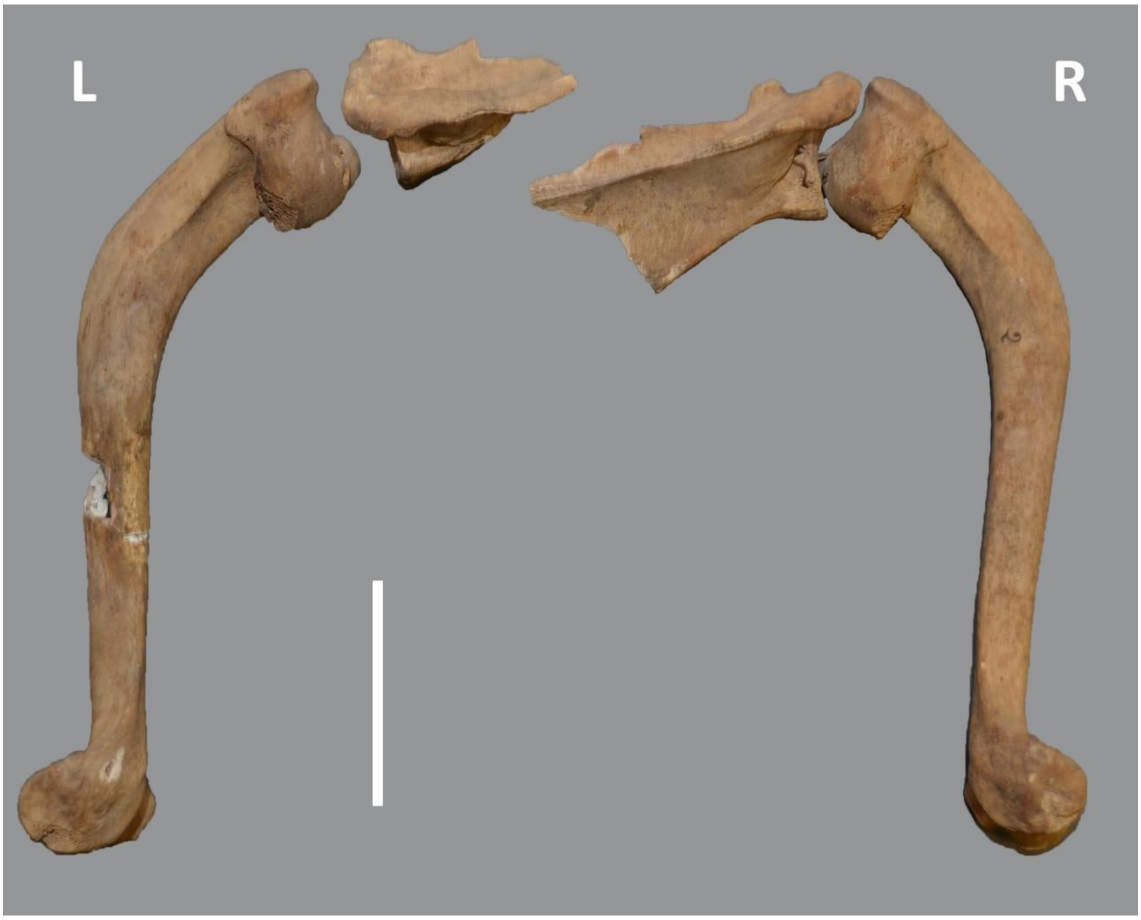

Figure SI-100: Left and right scapula and humerus of *Papio* sp. female MHNL 51000328B, showing the bent humeral shafts and the downward deviated humeral heads. Scale bar is 5 cm.

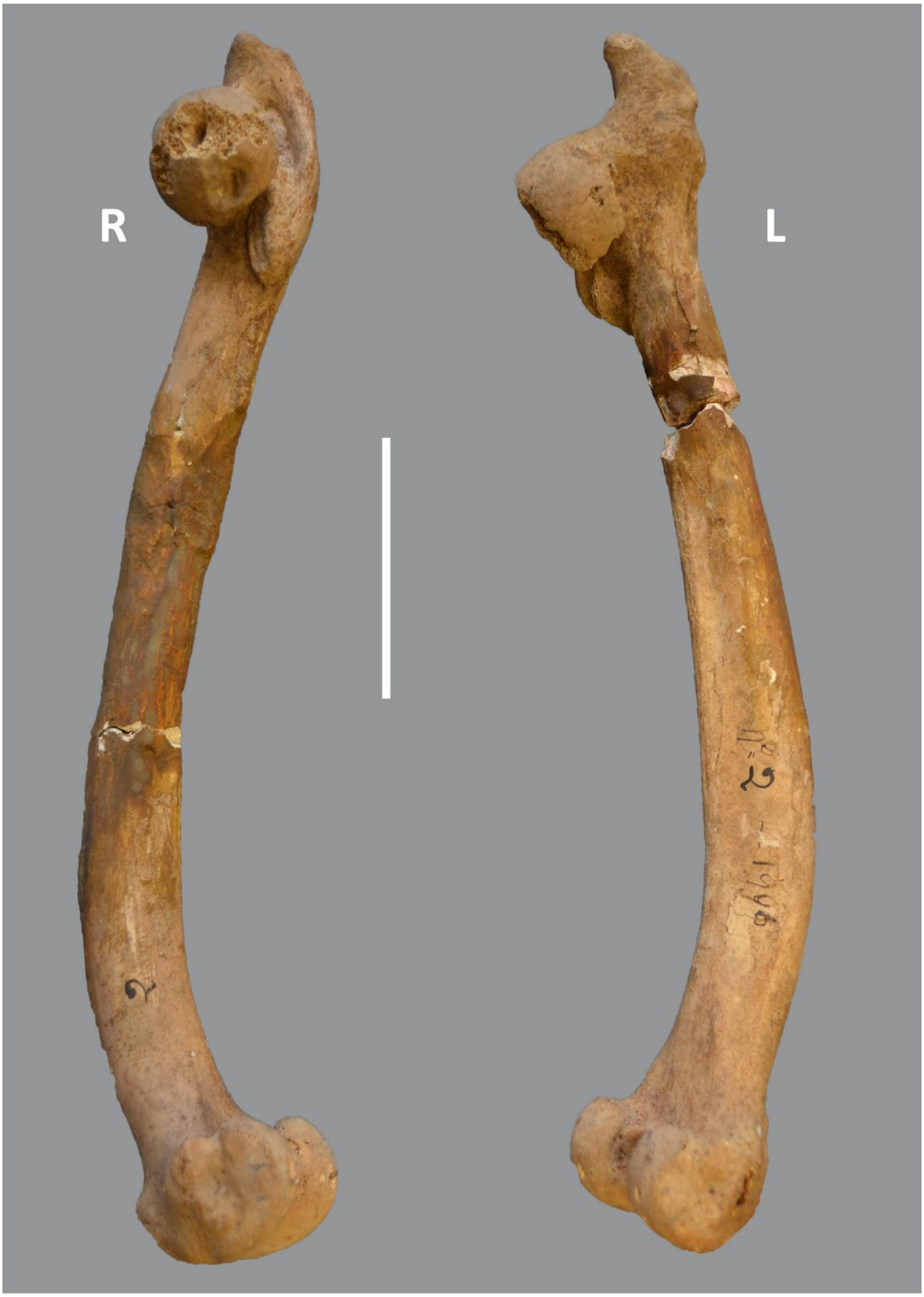

Figure SI-101: Left and right femur of *Papio* sp. female MHNL 51000328B, showing the bent shafts and the deformation of the caput. Scale bar is 5 cm.

Skeleton MHNL 51000324 *Papio* sp.; not in Lortet & Gaillard

The registernumber MHNL 51000324 refers to a series of bones that include several of the pathological specimens figured in Lortet and Gaillard (1907: figs. 92-95) and that were described by Dr Poncet (in Lortet & Gaillard 1907: 28-31).

Eight skeletal elements that are all heavily deformed belong to a single individual that developed a heavy case of rickets, a diagnosis that was already given in the initial description for the long bones (Lortet and Gaillard 1907: 28-29). All elements have intact cortical surfaces but are heavily bowed and their epiphyses are disproportionate. Besides these elements dealt with below, there still are a number of isolated bones from other individuals that were given the same register number MHNL 51000324. Those elements are described separately under a different heading (3. Isolated pathological bones).

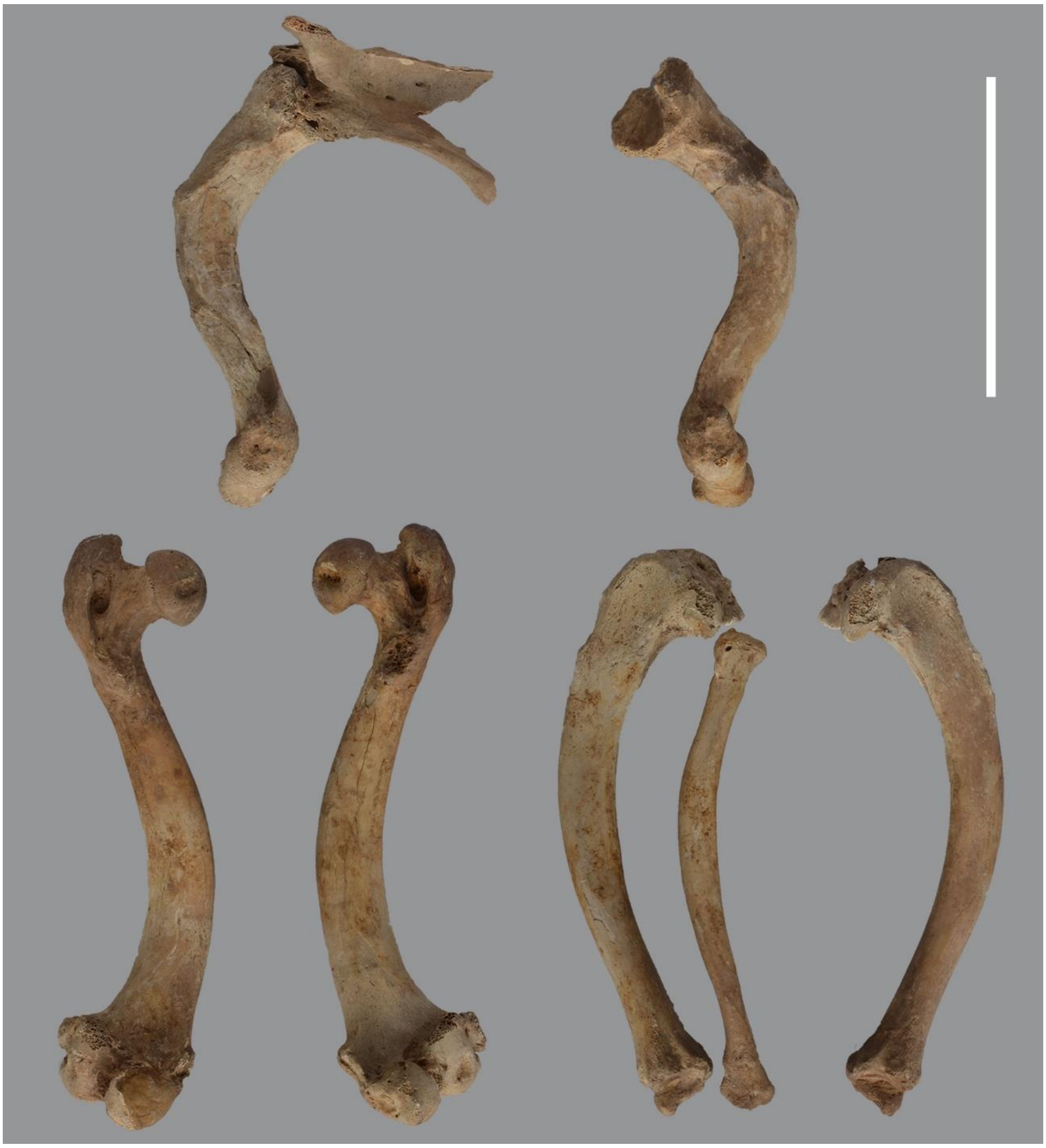

Figure SI-102: Overview of the long bones of *Papio* sp. MHNL 51000324 showing signs of rickets: left scapula and left and right humerus (top). Left and right femur (bottom left) and left tibia and right tibia and fibula (bottom right). Scale bar is 10 cm.

Scapula and humerus

Below the glenohumeral joint of the left scapula, at the level of the *Tuberositas infraglenoidalis* some osteophytes and a heavy muscular insertion occur. Three articular surfaces are observed in the distal scapula instead of the usual one. In this specimen, the glenoid cavity consists of two articular facets supplemented by a third that lies cranially. These newly formed facets constitute the scapular articulation; their irregular and rugose surfaces indicate remodelling with reactive new bone formation.

The left and right humerus are heavily bowed in an identical way. The proximal epiphysis has a flattened head that is deviated downward. Osteophytic development is noticed on the entire humeral head. The proximal articulation of the humerus has an almost concave aspect and consists of three articular surfaces, corresponding to the situation in the scapula. Unlike the scapula, however, the articular surfaces are rather smooth. No doubt a process of ankylosis had already set in, causing reduced mobility of the joint. The distal end shows a heavily developed medial epicondyle, possibly the result of constraints exerted by the situation in the proximal end. The *Crista supracondylaris lateralis* has a sharp-edged aspect and stepped distal end.

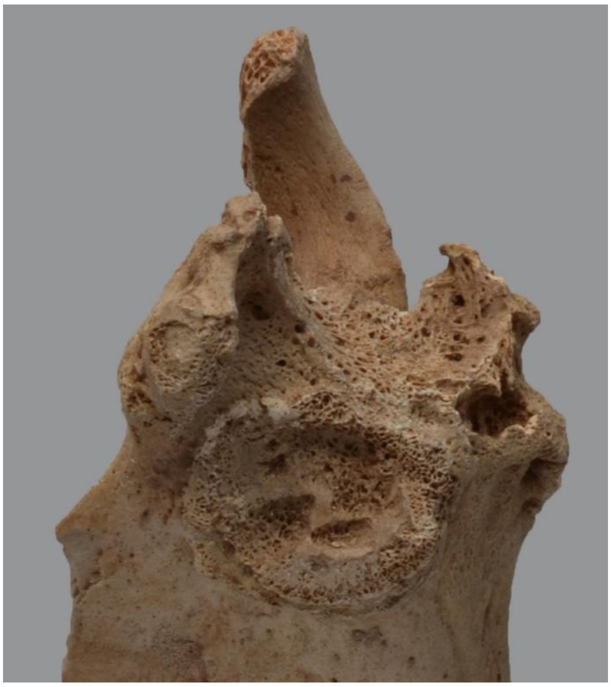

Figure SI-103: Distal view of the left scapula of *Papio* sp. MHNL 51000324, showing the three articular facets with their rugose and irregular appearance.

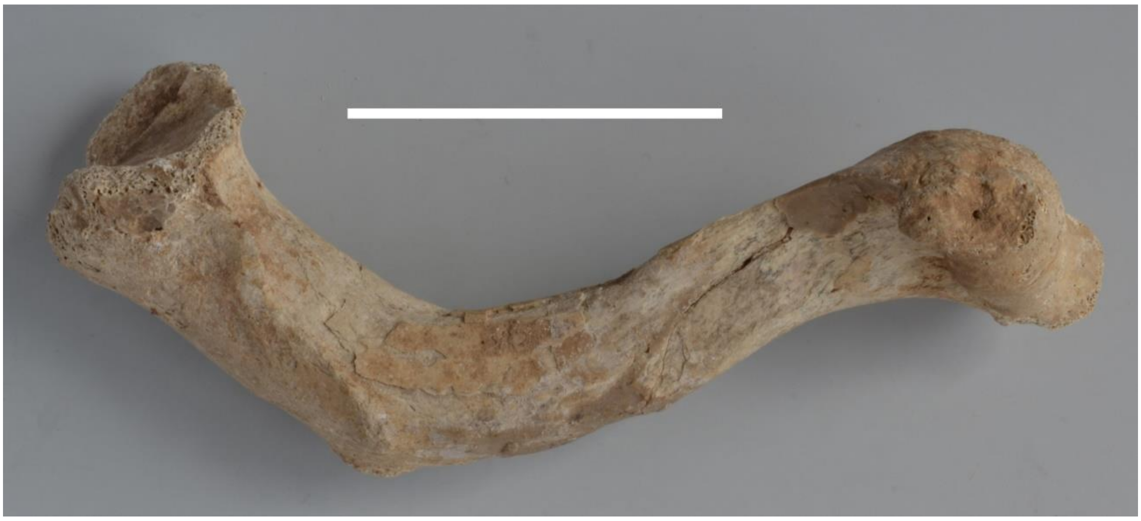

Figure SI-104: Lateral view of the left humerus of *Papio* sp. MHNL 51000324, showing the bent shaft and the nearly concave humeral head. Scale bar is 5 cm.

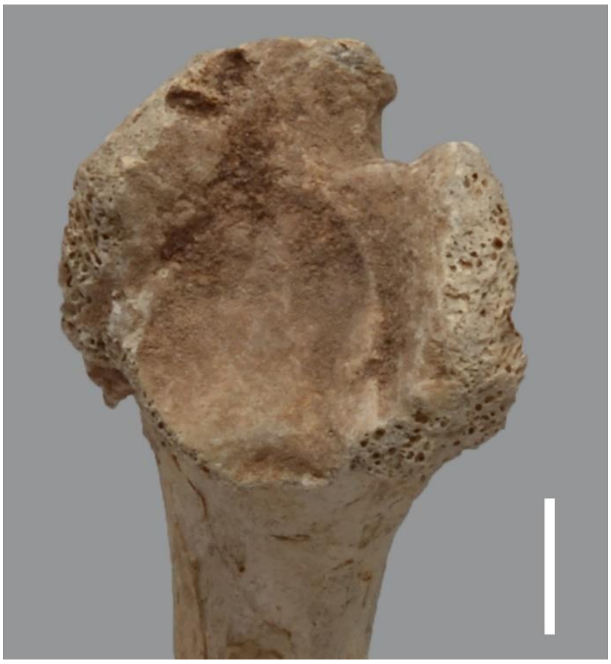

Figure SI-105: Proximal view of the left humerus of *Papio* sp. MHNL 51000324, showing the articulation composed of three facet surfaces. Scale bar is 1 cm.

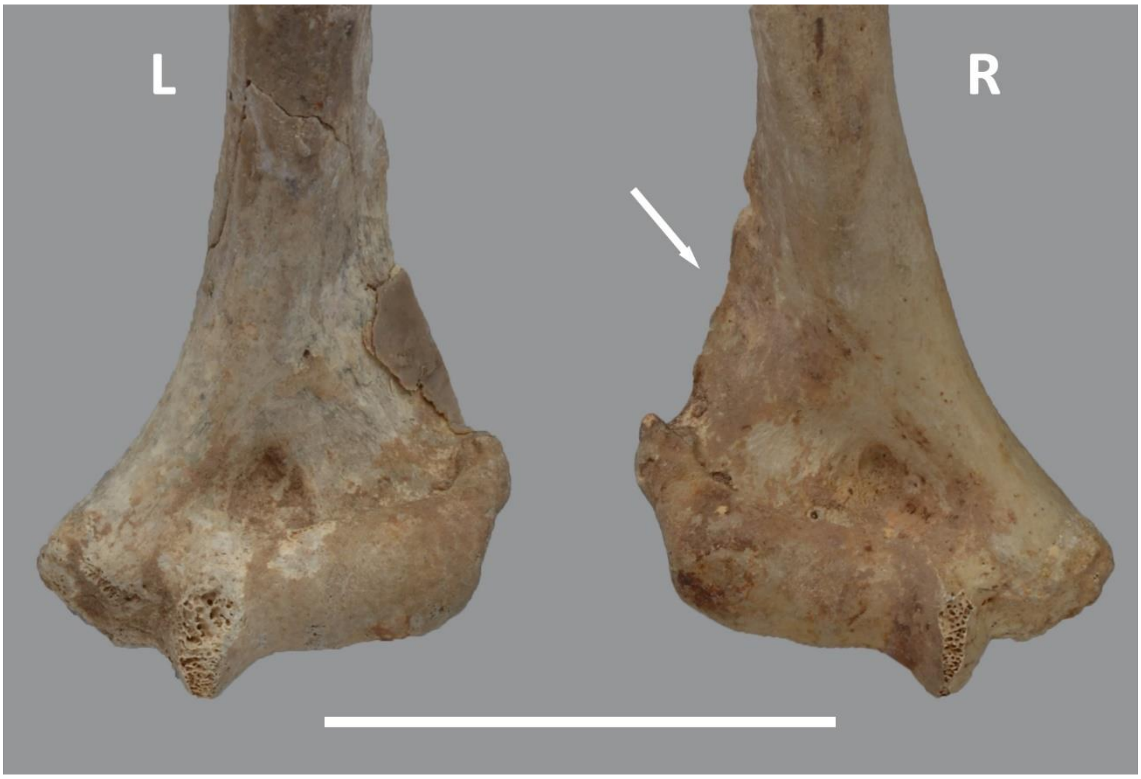

Figure SI-106: Cranial view of the distal end of the left and right humerus of *Papio* sp. MHNL 51000324, showing the disproportinately large medial epicondyle. Also note the sharp-edged aspect and stepped distal end of the *Crista supracondylaris lateralis* (arrow). Scale bar is 5 cm.

Femur

The left and right femur shafts show a comparable, severe bowing and in both specimens the cortical surface is normal. The proximal ends show a heavy development of both the trochanters major and minor; and the neck is shortened. The distal ends deviate in a lateral direction. Osteophytes of various sizes are observed throughout the distal region, although they are less developed in the medial condyles. The trochlea patellaris is only faintly indicated and its surface almost entirely eroded away.

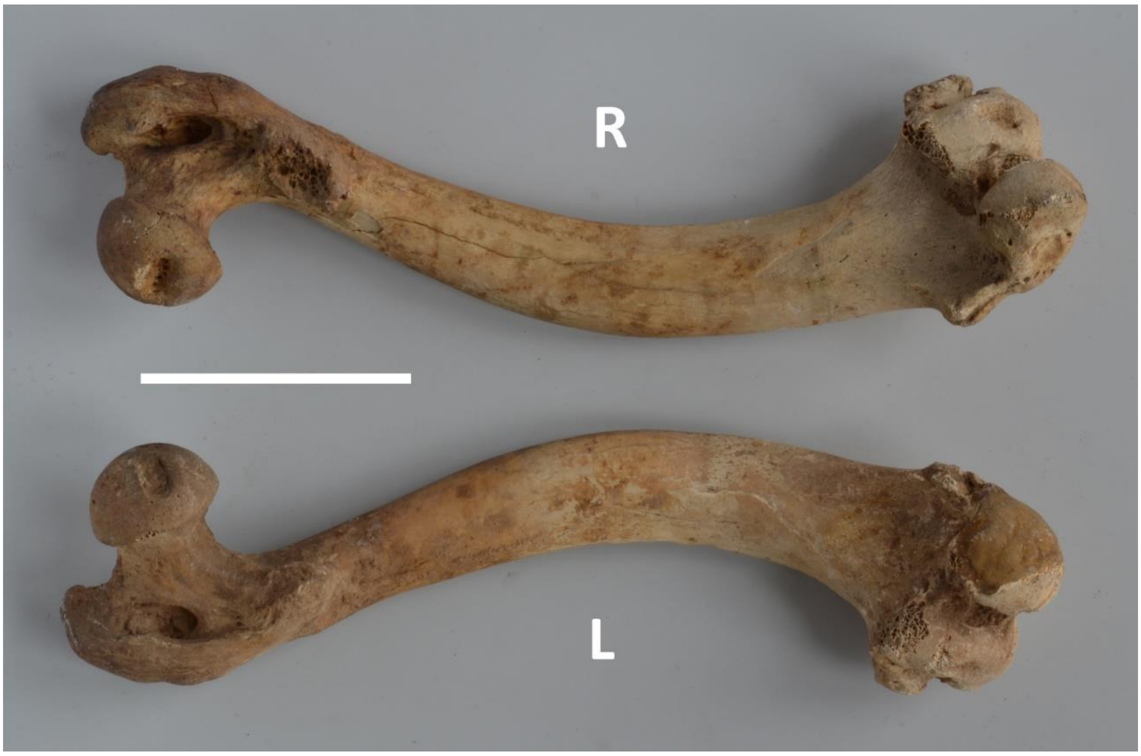

Figure SI-107: Caudal view of the right and left femur of *Papio* sp. MHNL 51000324, showing the bent shaft, the deviating distal ends, and the short femoral neck. Scale bar is 5 cm.

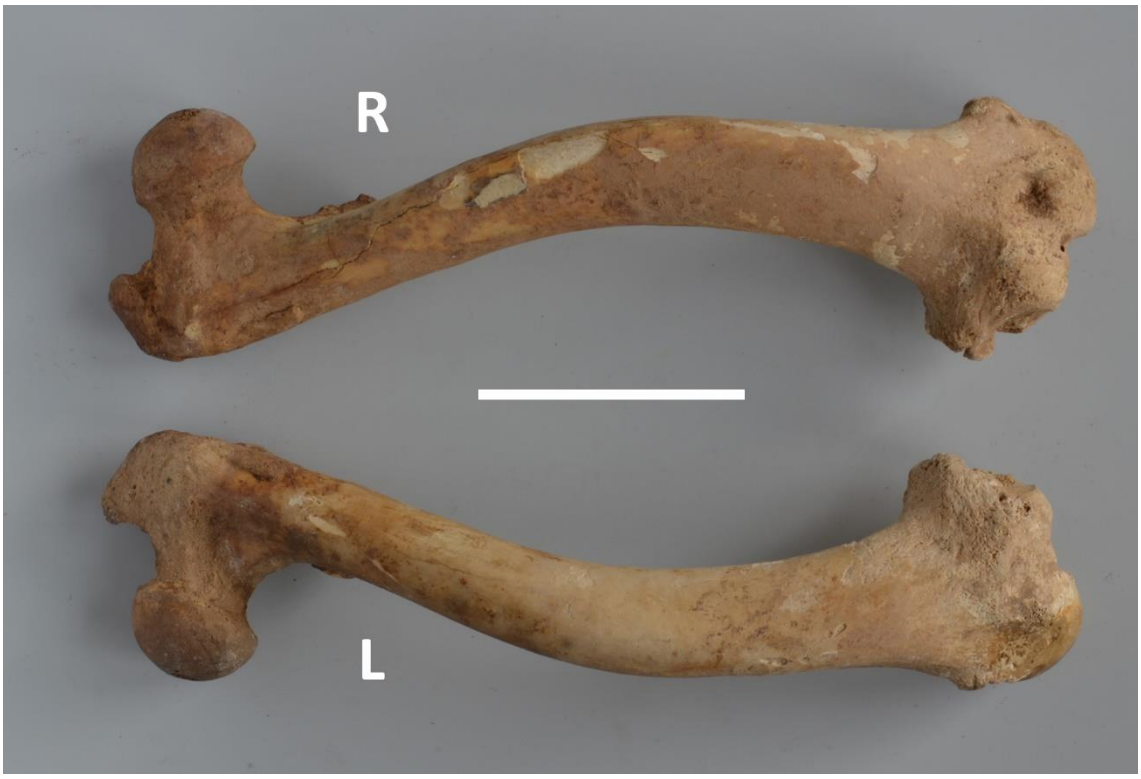

Figure SI-108: Cranial view of the right and left femur of *Papio* sp. MHNL 51000324, showing the bent shaft, the deviating distal ends, the short femoral neck, and the poorly indicated trochlea patellaris. Scale bar is 5 cm.

Tibia and fibula

Both tibiae and the sole preserved fibula are heavily bowed. On the fibula, no other deformations can be observed. The proximal epiphyses of the tibiae present erosive lesions with formation of reactive new bone all around the articular surface. The proximal articular surface shows cavities with an osteoporotic aspect. The tuberositas crista tibiae is eroded away. The distal part of the tibia appears anatomically unaltered but shows heavy muscular attachments.

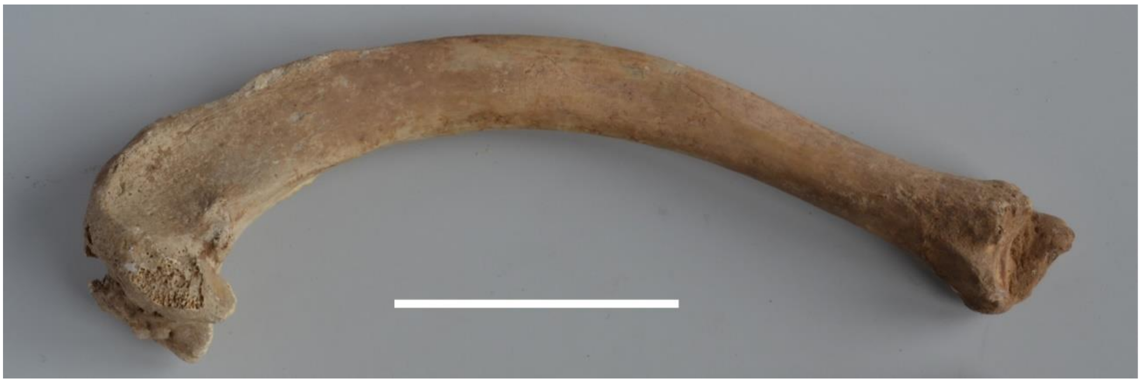

Figure SI-109: Lateral view of the right tibia of *Papio* sp. MHNL 51000324, with bowing of the shaft. Scale bar is 5 cm.

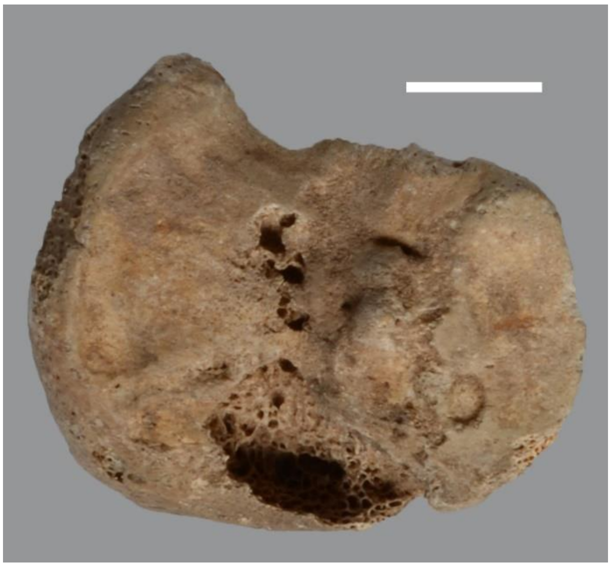

Figure SI-110: View of the proximal articulation of the right tibia of *Papio* sp. MHNL 51000324, showing erosive lesions, new bone formation around the articular surface and a pitted tibial plateau. Scale bar is 1 cm.

3. Isolated pathological bones

Besides the skulls and the more or less complete skeletons described above, the Lyon collections also contain numerous isolated bones, some of which show deformations. Several of these received the same registernumber MHNL 51000324, e.g., the rachitic individual mentioned above. In addition we found a few specimens with pathologies in the material that is stored by skeletal element under separate registry numbers.

Right radius MHNL 51000324

An isolated right radius has a bowed shaft while its proximal epiphysis is shaped normally. In the distal part, a minor swelling of the shaft is visible in its distal quarter on both the lateral and medial sides at respectively 3 cm and 1.5 cm.

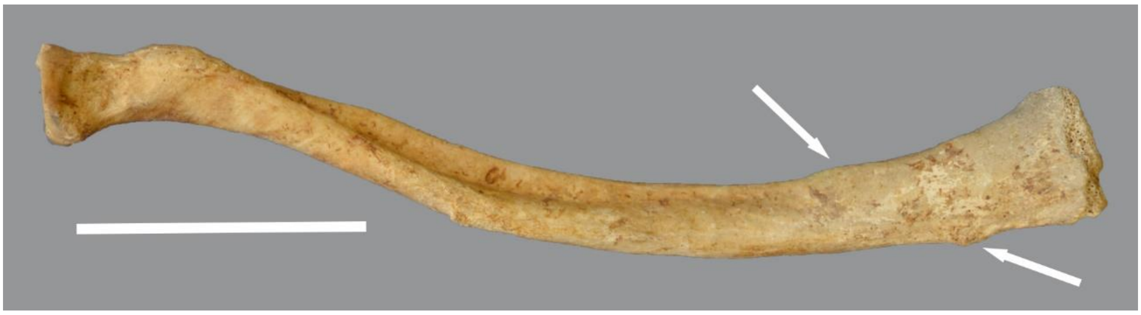

Figure SI-111: Palmar view of a right radius of *Papio* sp. MHNL 51000324, showing the bowed shaft and minor swelling of the shaft both laterally and medially. Scale bar is 5 cm.

Left humerus MHNL 51000324

The shaft of this humerus shows no bending. The proximal part of the bone has a cribriform aspect and the caput humeri has disappeared. The two tubercles are strongly eroded but are still partly preserved and they exhibit irregular surfaces. In the proximal part, the sulcus intertubercularis (bicipital groove) can be seen as well as some parts of the tuberculum minus and majus. On the medial side of the diaphysis, an ossified tendon occurs adjacent to the bicipital groove. Although the distal end is heavily weathered, it appears that the surface of the fossa coronoidea is slightly undulating and that it exhibits a small, circular perforation. The epicondyles appear swollen and the fossa ulnaris is not clearly delineated anymore.

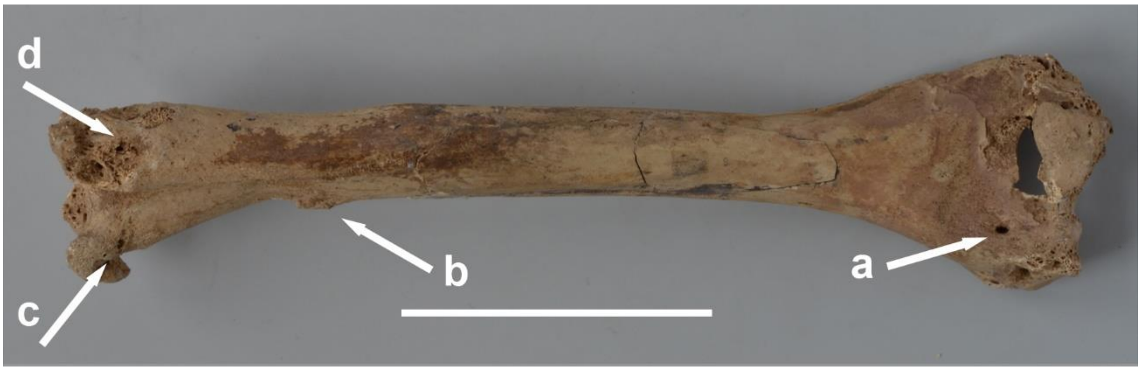

Figure SI-112: Cranial view of a left humerus of *Papio* sp. MHNL 51000324, showing the cribriform aspect of the proximal part and absence of the articular head. The arrows indicate the circular perforation in the fossa coronoidea (a), the position of the ossified tendon (b) near the bicipital groove and the approximate positions of the tuberculum minus (c) and majus (d). Scale bar is 5 cm.

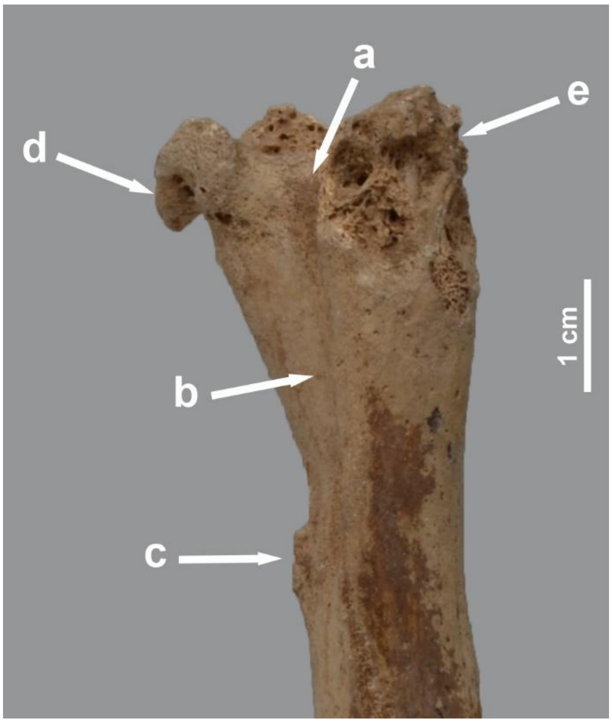

Figure SI-113: Cranial view of the proximal part of the left humerus of *Papio* sp. MHNL 51000324, showing the sulcus intertubercularis (a) and the bicipital groove (b), the ossified tendon (c), as well as some parts of the tuberculum minus (d) and majus (e).

Two lumbar vertebrae MHNL 51000324

Bony bridges connect the ventral bodies of two lumbar vertebrae, a condition diagnosed as spondylosis deformans.

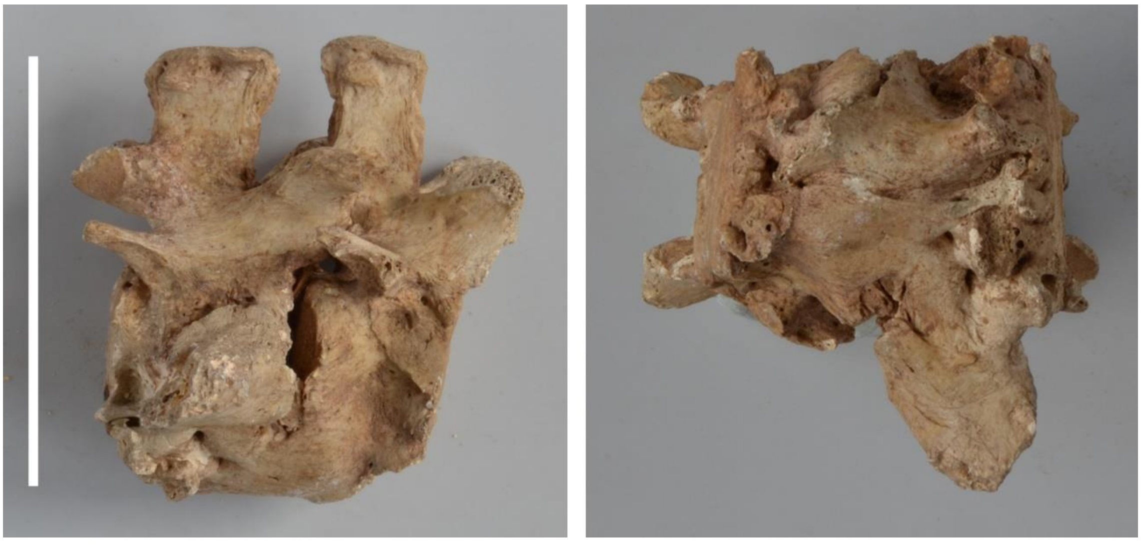

Figure SI-114: Left lateral and ventral view of two ankylosed lumbar vertebrae of *Papio* sp. MHNL 51000324. Scale bar is 5 cm.

Vertebral column MHNL 51000171

Some osteophytes are noted in the latero-ventral part of the centra of several vertebrae, but they are rather small and not yet projecting far towards adjacent vertebrae. This lipping was seen on the caudal end of the last three thoracal vertebrae, the cranial part of the first lumbar, the caudal part of the third lumbar and the articulating cranial part of the fourth lumbar, and the caudal part of the fifth lumbar and the cranial part of the articulating sacrum. The ventral part of the first three caudal vertebrae has numerous small osteophytes that are, however, restricted to each single vertebra and they do not have a tendency to build bony bridges as in the aforementioned vertebrae. Generally speaking, the vertebral bodies have an osteoporotic aspect.

Right radius & ulna MHNL 51000324

The pathology on a right radius and ulna was diagnosed by Poncet as being a “sarcome périostique ossifiant” (Lortet & Gaillard 1907: 31). Nerlich et al. (1993: 197) suggested that it could rather be an “exuberant (post-traumatic) callus formation rather than a malignant tumor”. We made a radiograph that at first sight seems to indicate the presence of a fracture underneath the callus. However, most likely we are dealing with a posterior incident that occurred during or after the excavation. The general, outer appearance of the ulna and in particular the radius is very irregular and in case one would argue in favour of a callus, the fracture must have occurred shortly before the baboon’s death. However, the radiograph that we made shows no evidence for a fracture. In fact, the disruption visible on the image is caused by a fresh, modern break that can be seen with the naked eye and that has been glued.

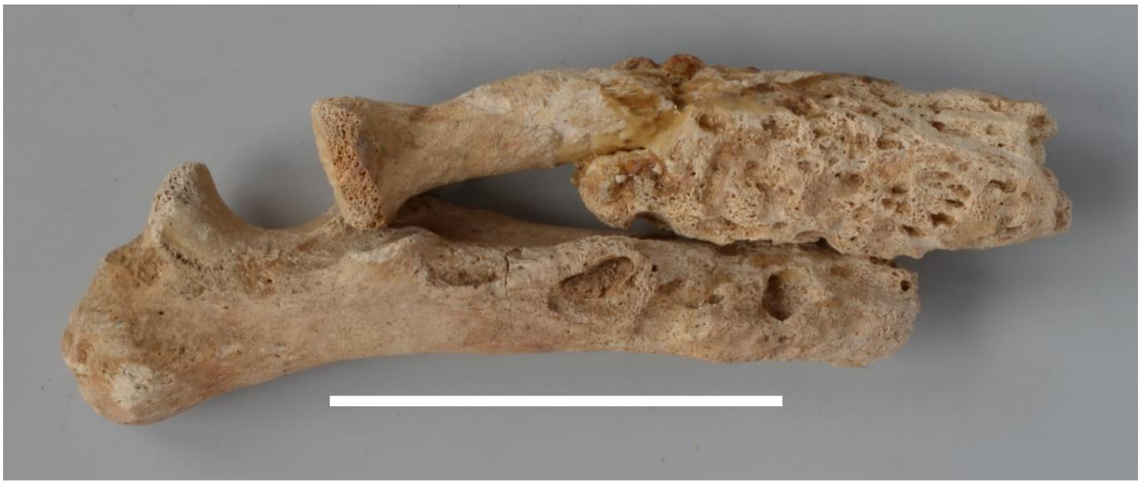

Figure SI-115: Lateral view of the right radius and ulna of *Papio* sp. MHNL 51000324 diagnosed as a “sarcome périostique ossifiant” in Lortet and Gaillard (1907: 31). The glue that was used to mend the recent break of the radius can be clearly seen. Scale bar is 5 cm.

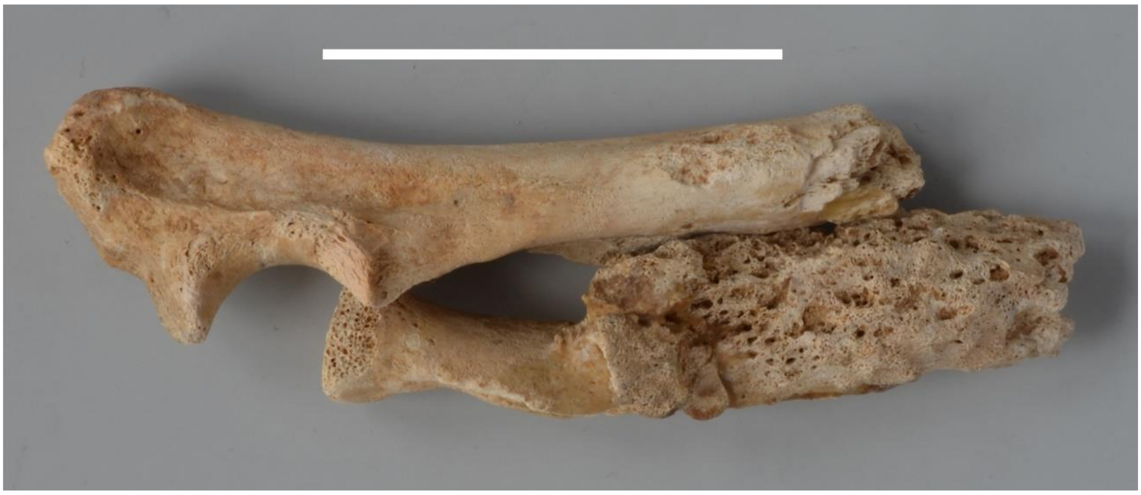

Figure SI-116: Medial view of the right radius and ulna of *Papio* sp. MHNL 51000324 diagnosed as a “sarcome périostique ossifiant” in Lortet and Gaillard (1907: 231). Scale bar is 5 cm.

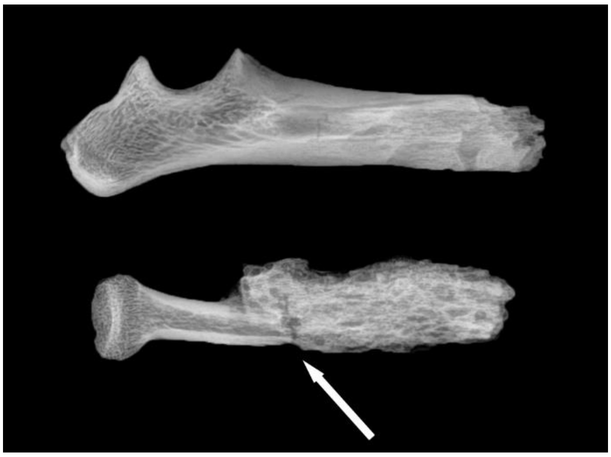

Figure SI-116: X-ray of the right radius and ulna of *Papio* sp. MHNL 51000324 diagnosed as a “sarcome périostique ossifiant” in Lortet and Gaillard (1907: 231) The arrow on the radius seems to indicate a transversal fracture but is in fact an artefact, due to a recent break.

Humeri MHNL 51000332

Among the isolated humeri, registered under number MHNL 51000332, are the left and right element of the same individual (GL right 164 mm; GL left 161 mm; see supplementary information S5 Table) of which the caput has the same pathological condition as the *Papio* cf. *anubis* MHNL 51000173. The humeral head is collapsed and directed downward. A right humerus with a GL of 167.5 mm has a similarly deformed caput.

Radii and ulnae

Eleven isolated radii are registered under the numbers MHNL 51000294, MHNL 51000331 and MHNL 51000333. A right radius from MHNL 51000331 with a GL of 180 mm is heavily bent.

None of the seven ulnae registered under the numbers MHNL 51000314, MHNL 51000331 and MHNL 51000333 show pathologies.

Femurs and tibiae

Thirteen isolated femurs are registered under the numbers MHNL 51000292, MHNL 51000294, MHNL 51000327 and MHNL 51000333. One of the specimens with number MHNL 51000294, a right femur of GL 193 mm, has an exostosis on the trochanter major comparable to what is seen in the skeleton labeled as *Papio* sp. male individual MHNL 51000170.

Twenty-four isolated tibiae are registered under the numbers MHNL 51000292 (2 left tibiae), MHNL 51000294 (1 left tibia) and MHNL 51000326 (9 left and 12 right tibiae). None of these bones show pathologies. From this assemblage, all 12 right tibia plus a single left tibia of baboon that was much larger, have been sampled for radiocarbon dating, stable isotope and possible future aDNA analysis.

Hand and foot bones

In the assemblage with registernumber MHNL 51000334, 75 metapodals and 12 first phalanges were counted. No fractures or any other pathologies were observed on these bones.

Mandibles

Besides a number of mandibles that belong to the skulls mentioned above, there are also 13 additional partial mandibles that are not associated to a particular skull. This material has been registered under eleven different numbers, in two cases it appeared that two mandible fragments with a different number belonged to the same individual (see S4 Table). In six cases, the mandibles come from adult animals, but there are also three individuals of which the M1 is the last erupted tooth and three of which the M2 is the last erupted tooth. None of these mandibles show pathologies, but in four of them calculus occurs that has been sampled for metagenomic analysis (Otttoni et al. 2019).

Femur MHNL 51000171

The right hind leg of the skeleton labelled MNHL 51000171 consists of a femur and tibia that are obviously not from the same individual. Both long bones almost equal each other in greatest length, i.e. 208 mm (femur) and 213 mm (tibia), unlike the situation that is seen in the other skeletons, where the femur is always markedly longer than the tibia (Supplementary information, S2 Table).

The shaft of the right femur is not bend, but the bone appears to be rather sturdy. At the cranial side, the diaphysis shows in its proximal half a rugose zone of 4 by 2 cm that is probably the result of a growth disorder. At the proximal end a heavy muscular relief is observed, in particular at the level of the trochanter major. The margin of the neck shows a sharp, irregular ridge. The transition zone linking the caput to the neck is also rugose. The fovea also seems pitted. The trochanter minor is irregularly enlarged.

The distal end of the femur is heavily deformed with the condyles clearly tilted in a medial direction. Periarticular osteophytes are observed on the trochlea and at the level of both condyles in particular on the medial one that is in addition enlarged. On the cranial side of the shaft, the area of the fossa suprapatellaris is pitted. The fossa patellaris itself is irregularly shaped and has a rugose surface.

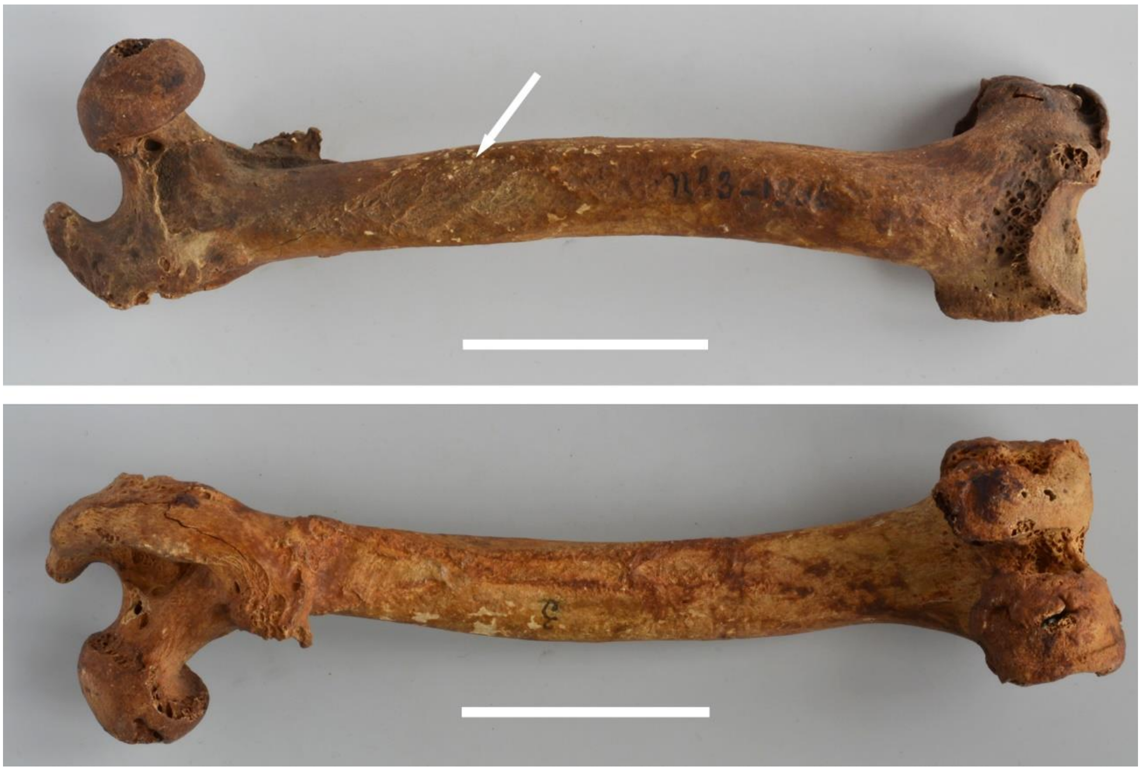

Figure SI-118: Cranial (above) and caudal (below) view of the right femur of *Papio* sp. male MHNL 51000171, showing the sturdy appearance of the bone and the presence of a rugose zone on the cranial part of the shaft. Scale bars are 5 cm.

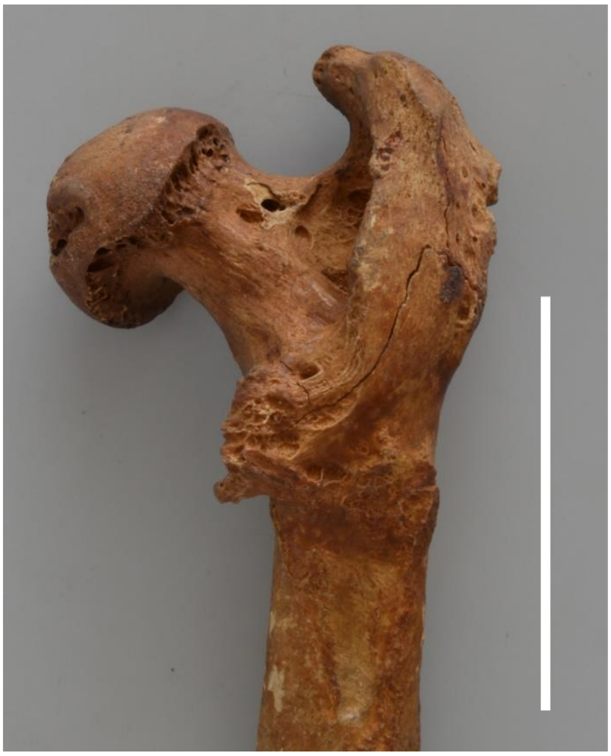

Figure SI-119: Caudal view of the proximal end of the right femur of *Papio* sp. male MHNL 51000171, showing the sharp, irregular ridge at the margin of the neck, the rugose transition zone linking the caput to the neck, and the irregularly enlarged trochanter minor. Scale bar is 5 cm.

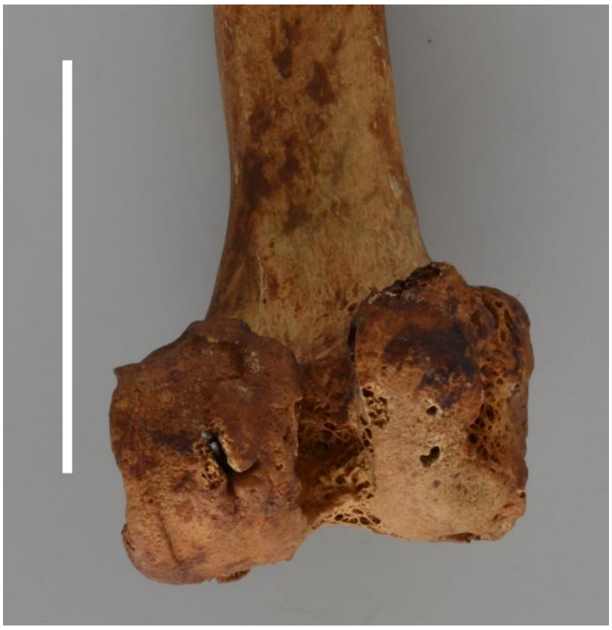

Figure SI-120: Caudal view of the distal end of the right femur of *Papio* sp. male MHNL 51000171, showing the osteophytes on the condyles. Scale bar is 5 cm.

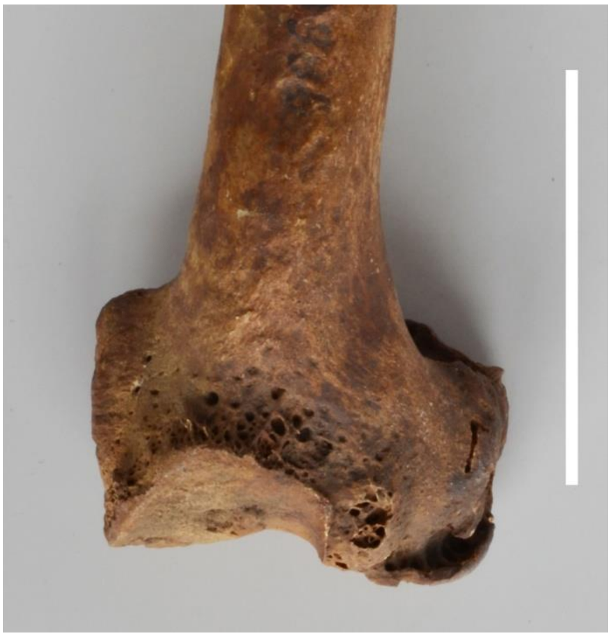

Figure SI-121: Cranial view of the distal end of the right femur of *Papio* sp. male MHNL 51000171, showing the tilting on a medial direction, the irregular shape of the fossa patellaris and the pitting above that fossa. Scale bar is 5 cm.

4. Bibliography

Lortet L., Gaillard Cl. 1907. La faune momifiée de l'ancienne Egypte. Archives du Muséum d'histoire naturelle de Lyon 9. Georg: Lyon.

Lortet, L. & Cl. Gaillard. 1909. La faune momifiée de l'ancienne Égypte et recherches anthropologiques III. Archives du Muséum d'histoire naturelle de Lyon 10. – Lyon, Henri Georg.

Ottoni C., Guellil M., Ozga A., Stone A., Kersten O., Bramanti B., Porcier S., Van Neer W., 2019. Metagenomic analysis of dental calculus in ancient Egyptian baboons Scientific Reports, 9(1):19637.

Reed OM. 1965. Studies on the dentition and eruption patterns in the San Antonio baboon colony. In The Baboon in Medical Research. Vol. 1, Vagtborg H (ed.). University of Texas Press: Austin; 167-180.

Reed OM 1967. Cephalometric studies of the growth, development, and eruption pattern of the baboon. In The Baboon in Medical Research. Vol. 2, Vagtborg H (ed.). University of Texas Press: Austin; 181-186.
