# Supplementary material for: Palaeopathological and demographic data reveal conditions of keeping of the ancient baboons at Gabbanat el-Qurud (Thebes, Egypt)
Source: PLoS One. 2023 Dec 6;18(12):e0294934. doi: 10.1371/journal.pone.0294934 (PMC10699651; doi:10.1371/journal.pone.0294934)
Supplement: S1 Table — Indicated are the registernumbers of the Royal Belgian Institute of Natural Sciences (RBINS), the species, sex and provenance of the animals. (PDF) [file pone.0294934.s002.pdf]

S1 Table. List of modern baboon specimens consulted for comparison.

RBINS#	species	sex	provenance	last erupted tooth	attrition stage
5491	*Papio anubis*	male	Maniema, Congo DR	M3	22
8879	*Papio anubis*	male	Kivu, Congo DR	M3	19
16175	*Papio anubis*	male	Kivu, Congo DR	M3	21
34934	*Papio anubis*	female	Kisangani, Congo DR	M3	20
34935	*Papio anubis*	male	Kisangani, Congo DR	M3	19
811B	*Papio hamadryas*	male	Antwerpen Zoo	M3	12
811D	*Papio hamadryas*	male	Antwerpen Zoo	M3	20
4288	*Papio hamadryas*	male	Antwerpen Zoo	M3	24
4264	*Papio hamadryas*	male	Antwerpen Zoo	M3	19
4266	*Papio hamadryas*	male	Antwerpen Zoo	M3	20
4271	*Papio hamadryas*	male	Antwerpen Zoo	M3	12
4412	*Papio hamadryas*	male	Antwerpen Zoo	M3	20
40233	*Papio hamadryas*	male	Antwerpen Zoo	M2	8
