# Supplementary material for: Palaeopathological and demographic data reveal conditions of keeping of the ancient baboons at Gabbanat el-Qurud (Thebes, Egypt)
Source: PLoS One. 2023 Dec 6;18(12):e0294934. doi: 10.1371/journal.pone.0294934 (PMC10699651; doi:10.1371/journal.pone.0294934)
Supplement: S2 Table — Indicated are the MHNL registration numbers, the corresponding numbers of Lortet and Gaillard [19], the species name and the sex. Measuring distances and their abbreviations are as in von den Driesch [36].The skeletons MHNL 51000172, MHNL 51000173 and MHNL 51000325 each have measurements that are coherent for one individual. As skeleton MHNL 51000328B is from a rachitic individual, measurements of the greatest length of humerus and femur are not provided. The three other skeletons are clearly reconstructed from more than one individual, but for completeness, we also give the measurements of these bones. In the case of skeleton MHNL 51000170, the dimensions of the arms show that bones from at least one additional individual were used to compose a more or less complete skeleton. Specimen MHNL 51000171, is partly composed of bones from a pathological specimen whose measurements are indicated in bold. MHNL 90002100, a skeleton thought to belong to a baboon found in a jar, is also composed of skeletal elements from more than one individual, as shown by the differences in left and right values for several bones. The distal epiphysis of the left radius has not yet fused, unlike all other long bones. This means that this skeleton consists of at least three individuals. (PDF) [file pone.0294934.s003.pdf]

S2 Table. Measurements, in mm, of the long bones of seven baboon skeletons from Gabbanat al-Gurud.

	51000172		51000173		51000325		51000328B		51000170		51000171		90002100A	
	skeleton 1 of Lortet & Gaillard		skeleton 8 of Lortet & Gaillard						mixed		mixed		mixed	
	P. hamadryas		*Papio* sp.		*Papio* sp.		*Papio* sp.		*Papio* sp.		*Papio* sp.		*Papio* sp.	
	female		male		male		female		male		male		?	
	left	right	left	right	left	right	left	right	left	right	left	right	left	right
**scapula**														
HS	-	113	122.5	127	-	-	-	-	133	-	-	-	-	-
SLC	-	21.9	30.3	28.4	-	-	24.0	-	28.9	30.2	-	-	25.9	-
GLP	-	33.5	40.1	41.1	-	-	32.6	-	41.7	39.0	-	-	34.0	33.8
LG	-	22.2	32.6	31.7	-	-	24.7	-	29.1	28.0	-	-	23.3	24.4
BG	-	17.4	22.1	22.3	-	-	17.6	-	17.6	18.9	-	-	16.8	15.8
**humerus**														
GL	200	198	195	195	-	220	-	-	197	-	-	-	178	179
Bp	33.6	34.5	39.4	41.3	-	35.5	33.5	31.5	31.1	33.5	-	-	28.3	27.5
Dp	37.1	35.8	36.1	34.5	-	35.8	32.8	-	-	35.3	-	-	30.3	30.5
SD	15.1	15.6	14.6	14.6	-	17.0	-	13.0	15.9	12.6	-	-	13.5	13.9
Bd	38.7	40.6	45.8	45.5	-	45.6	39.3	38.6	38.3	-	-	-	33.4	33.7
BT	29.6	30.2	34.5	34.2	-	34.2	-	28.6	29.4	-	-	-	27.0	-
**radius**														
GL	197.5	199	205	206	233	230	203	205	208	215	207	**207**	(206)	201
Bp	17.7	18.5	20.6	20.8	20.4	20.5	18.5	18.7	18.1	17.2	19.2	**21.5**	17.3	18.3
Bd	24.8	25.0	28.6	28.6	27.6	28.5	23.4	23.5	23.1	23.6	27.0	**29.2**	24.0	23.6
**ulna**														
GL	220	220	230	229	258	257	-	227	227	235	224	-	-	-
BPC	24.7	25.8	25.9	25.6	30.0	28.8	22.1	-	22.7	23.1	25.0	-	24.4	22.9
DPA	28.6	27.7	29.5	30.7	30.8	31.6	28.7	28.6	27.7	27.8	28.9	-	27.1	29.4
**pelvis**														
GL	174	172	193	139.5	-	191	-	-	193	192.5	204	207	182	-
LA	25.3	25.2	30.5	29.2	32.6	28.1	-	-	30.8	30.1	33.6	32.2	29.6	30.6
**femur**														
GL	205	206	231	233	259	258	-	-	231	233	223	208	218	223
Bp	40.4	41.7	50.8	51.0	50.6	51.3	-	-	47.0	47.5	44.8	53.4	48.3	49.2
DC	19.0	19.1	24.6	24.7	25.4	25.3	-	-	22.6	22.7	22.1	26.5	22.5	22.5
SD	14.8	15.0	17.0	16.4	17.6	18.6	14.1	-	15.5	16.3	16.6	16.5	16.5	16.6
Bd	32.1	31.2	46.1	49.7	42.2	41.8	40.0	40.6	39.3	37.6	38.5	52.8	40.0	40.1
**patella**														
GB	-	-	23.4	27.1	24.7	23.8	-	-	-	-	-	-	-	-
GL	-	-	21.5	21.5	18.6	18.4	-	-	-	-	-	-	-	-
**tibia**														
GL	196	195	210	211	225	223	198	-	200	199	230	**213**	203	204.5
Bp	37.8	37.8	43.9	47.5	43.6	43.2	38.8	37.6	39.0	38.7	43.4	**42.5**	39.2	38.6
SD	11.4	11.1	12.1	12.6	13.2	13.2	11.2	11.8	17.0	11.8	11.6	**12.1**	11.2	11.5
Bd	26.4	25.4	27.1	27.0	29.1	27.7	-	26.1	25.5	25.0	-	**31.7**	24.7	24.1
Dd	20.4	19.3	25.6	24.7	-	-	-	-	19.0	19.0	-	-	19.6	19.7
**fibula**														
GL	180	178	188	190	203	204	-	-	184	184	-	**188**	-	-
Dp	18.3	17.1	20.0	19.4	18.3	17.5	-	-	14.7	14.5	-	**18.3**	-	-
Dd	18.0	17.5	16.9	16.5	17.2	18.0	-	-	15.8	15.1	-	**19.6**	-	-
**calcaneus**														
GL	44.4	43.4	52.0	55.0	-	-	46.5	-	45.4	43.5	-	-	38.5	39.4
GB	22.2	(20)	-	-	-	-	21.8	-	24.4	24.4	-	-	21.4	19.8
**astragalus**														
GL	-	24.6	31.4	31.9	-	-	29.7	-	-	29.8	-	-	-	-
