# Supplementary material for: Palaeopathological and demographic data reveal conditions of keeping of the ancient baboons at Gabbanat el-Qurud (Thebes, Egypt)
Source: PLoS One. 2023 Dec 6;18(12):e0294934. doi: 10.1371/journal.pone.0294934 (PMC10699651; doi:10.1371/journal.pone.0294934)
Supplement: S3 Table — Indicated are the MHNL registration numbers, the corresponding numbers of Lortet and Gaillard [19], the species name, the sex, the last erupted tooth and the attrition stage of the dentition. Measuring distances, including the numbers assigned to them, are those of von den Driesch et al. [17]. The only additional measuring distance, defined by us, is indicated with an asterisk. Measurements in brackets are approximate. The attrition stages are according to Reed [33, 34]. NA: not available. (PDF) [file pone.0294934.s004.pdf]

S3 Table. Measurements, in mm, of the baboon skulls and associated mandibles from Gabbanat al-Gurud.

**MHNL number**	51000170	51000171	51000172	51000173	51000174	51000175	51000176	51000177	51000178	51000179	51000180	51000181	51000182	51000183	51000318	51000319	51000320c	51000321	51000323A	51000323B	90002100A
**Lortet & Gaillard number**	3		1	8		5	9	10	6	2	12			7			4				11
**species**	*P. hamadryas*	*P. hamadryas*	*P. hamadryas*	*P. anubis*	*P. hamadryas*	*P. anubis*	*P. anubis*	*P. anubis*	*P. anubis*	*P. hamadryas*	*Papio* sp.	*Papio* sp.	*P. anubis*	*P. hamadryas*	*Papio* sp.	*Papio* sp.	*P. anubis*	*Papio* sp.	*Papio* sp.	*Papio* sp.	*P. anubis*
**sex**	female	male	female	male	male	female	female	female	female	female	male		male	male		male	female		male		female
**SKULLS**																					
**last erupted tooth**	M3	M3	M3	M3	M3	M3	M2; M3 pierced	M3	M3	M3	M2; P3 & P4 in crypt	M3	NA	M3	NA	NA	M3 in crypt	M2 in crypt	NA	M3 in crypt	M3
**attrition stage**	12	15	16	17	18	17	8	12	19	16	-	?	-	17	-	-	-	-	-	-	10-11
1) Total length: Akrokranion - Prostion	169	198	171	211	201	-	163	176.5	191	158	(161)	-	-	220	-	-	175	-	-	-	173
2) Condylobasal length	132	151.5	132	166.5	147	-	123.5	136	142.5	120	(120)	-	-	164	-	-	126.5	-	-	-	131
3) Basal length	118	138	120	152	-	-	112	125.5	131	108.5	(107)	-	-	150	-	-	117	-	-	-	118
4) Basion - Staphylion	45.2	50.5	44.8	51.8	-	42.1	40.0	45.5	45.1	44.5	42.8	-	-	55.5	-	-	44.1	-	-	-	43.5
5) Median palatal length: Staphylion - Prostion	72.0	87.3	76.0	101.2	85.5	-	71.1	77.6	85.6	63.3	(64)	-	-	92.5	-	-	73.7	-	-	-	70.2
6) Upper neurocranial length: Akrokranion - Supraorbitale	102.1	113.5	107.2	105.6	122.1	108.0	103.1	108.3	111	106.4	112	-	-	(130)	122	112	103.5	-	123.5	-	107.7
7) Facial length: Supraorbitale - Prostion	99.5	115	99.0	139.5	120.5	-	106	103	124	85.1	105	-	-	(131)	-	-	114.3	-	-	-	105.4
8) Greatest breadth of the skull: Zygion - Zygion	100.8	-	107.5	-	-	102.6	98.5	111.5	115.5	97.0	93.5	-	-	-	-	-	103.7	-	-	-	102.8
9) Dental length: Postdentale - Prostion	71.2	86.1	75.5	90.0	84.6	-	77.5	77.5	76.7	64.8	-	-	-	91.5	-	-	-	-	-	-	76.3
10) Length from the oral border of C to the aboral border of M3	55.1	65.8	58.5	69.5	67.3	52.0	62.2	62.0	59.6	56.3	-	-	-	71.6	-	-	-	-	-	-	61.3
11) Alveolar length of cheecktooth row (P3-M3)	48.3	53.8	47.8	54.0	52.8	48.4	52.8	52.4	50.0	47.0	-	-	-	50.1	-	-	-	-	-	-	53.6
12) Length of molar row (M1-M3)	33.1	37.5	32.7	38.5	38.5	33.6	37.1	35.5	34.5	32.3	-	38.1	-	40.6	-	-	-	-	-	-	38.7
13) Length of premolar row (P3-P4)	13.5	15.0	13.6	15.9	14.2	13.8	15.3	14.5	13.8	13.3	-	-	-	16.1	-	-	18.4	-	-	-	14.0
14) Length of canine alveolus	8.7	15.0	10.4	19.9	17.3	6.8	11.0	11.2	8.4	9.1	-	-	-	16.5	-	-	-	-	-	-	8.9
15) Greatest palatal breadth, measured across the outer borders of alveoli	52.0	56.0	53.8	59.6	57.9	52.4	51.7	53.6	50.1	50.7	-	-	-	60.0	-	-	52.6	-	-	-	52.8
16) Greatest breadth across the alveoli of the canines	40.8	51.5	43.6	56.7	56.1	-	40.2	41.6	41.6	37.8	-	-	-	55.3	-	-	-	-	-	-	40.3
17) Greatest breadth of the occipital condyles	27.4	31.8	27.8	30.6	-	27.2	27.6	29.7	31.5	27.2	28.1	34.4	-	32.5	-	-	27.7	-	-	-	29.6
18) Greatest mastoid breadth: Otion - Otion	79.2	99.0	80.2	100.0	-	84.2	79.6	85.9	85.6	77.6	80.5	90.6	-	119.8	-	98.2	78.8	-	92.7	-	83.2
19) Least frontal breadth	54.2	56.3	57.8	52.8	54.8	55.3	56.1	57.2	56.6	56.6	58.5	64.1	57.5	(61)	62.6	51.4	55.9	53.2	58.9	53.5	51.5
20) Height of occipital triangle: Akromion - Basion	56.6	65.5	54.7	67.8	-	58.1	53.1	57.4	64.1	53.8	54.5	63.8	-	72.1	-	68.5	63.5	-	-	-	58.1
21) Greatest inner height of the orbit	25.2	25.2	23.8	23.2	26.8	24.9	25.5	25.7	27.2	23.8	23.8	28.6	22.5	23.0	-	27.2	26.2	-	-	-	27.1
22) Greatest inner breadth of the orbit	28.5	32.2	26.3	28.6	30.4	25.7	28.5	31.0	29.2	25.2	28.1	30.6	28.9	32.5	-	32.0	29.7	-	-	-	28.8
23*) Interorbital breadth (breadth between both orbits)	9.0	13.5	9.5	15.5	12.5	8.5	10.4	9.8	8.8	7.6	12.3	11.5	11.5	18.2	-	15.0	11.0	-	-	-	7.9
**MANDIBLES**																					
**last erupted tooth**	M3	M3	M3	M3	M3	M3	M3 piercing. not at level	M3	NA	M3	M2 piercing ± at level	NA	NA	NA	NA	NA	M3 just pierced	NA	NA	NA	M3
**attrition stage**		15	15	?	17	15?	9?	?	-	17	-	-	-	-	-	-	-	-	-	-	10
1) Greatest length: aboral border of condyle - Infradentale	122.4	143.5	120.5	150	145.5	131.5	122.5	-		110.8	-						123.5				121
2) Length of horizontal ramus: aboral border of alveolus of M3 - Infradentale	71.4	84.2	73.8	89.5	88.2	78.0	-	-		69.1	-						74.4				78.2
3) Length from aboral border of condyle to aboral border of alveolus of M3	53.9	61.5	50.5	61.4	57.7	51.4	-	-		42.8	-						48.9				44.2
4) Length from aboral border of alveolus of M3 to oral border of C (C-M3)	62.4	76.7	63.0	80.9	79.9	64.2	-	-		62.1	-						-				67.5
5) Length of cheecktooth row (P3-M3)	55.1	69.4	53.3	72.0	68.2	56.0	-	-		54.6	-						58.6				59.8
6) Length of molar row (M1-M3)	38.6	41.7	35.5	42.7	41.4	35.5	-	(41.5)		36.8	-						37.4				41.0
7) Height of vertical ramus	51.6	58.0	58.0	52.0	60.4	57.7	56.1	-		57.6	-						-				-
8) Height of mandible behind M3	27.6	30.2	29.5	31.6	32.1	26.8	-	-		25.3	-						29.5				-
11) Length of the symphysis	37.2	50.0	33.9	49.4	47.1	41.1	33.1	-		33.8	39.8						39.3				38.3
