# Supplementary material for: Palaeopathological and demographic data reveal conditions of keeping of the ancient baboons at Gabbanat el-Qurud (Thebes, Egypt)
Source: PLoS One. 2023 Dec 6;18(12):e0294934. doi: 10.1371/journal.pone.0294934 (PMC10699651; doi:10.1371/journal.pone.0294934)
Supplement: S4 Table — Indicated are the MHNL registration numbers, the species name, the sex, the last erupted tooth and the attrition stage of the dentition. Measuring distances, including the numbers assigned to them, are those of von den Driesch et al. [17]. Additional measuring distances defined by us are indicated with an asterisk. Measurements in brackets are approximate. The attrition stages are according to Reed [33, 34]. (PDF) [file pone.0294934.s005.pdf]

S4 Table. Measurements, in mm, of baboon mandibles from Gabbanat al-Gurud that could not be associated with a skull.

**MHNL number**	51000304	51000305	51000322	51000323a	51000323d	51000323x	51000323y	51000506	51000508	51000510	51000315+504	51000505+507
**species**	*Papio* sp.	*Papio* sp.	*Papio* sp.	*Papio* sp.	*Papio* sp.	*Papio* sp.	*Papio* sp.	*Papio* sp.	*Papio* sp.	*Papio* sp.	*Papio* sp.	*Papio* sp.
**sex**	?	male	?	male ?	male	female	male	?	?	?	male	?
**last erupted tooth**	M3	M3	M1	M3	M3	M3	M3	Pd4	M2	M1	M2	M1
**attrition stage**	10-13	15	6	-	13-14	17-19	-	-	-	-	10	6
1) Greatest length: aboral border of condyle - Infradentale	-	146.0	-	-	-	118.5	-	-	-	-	129.0	111.0
2) Length of horizontal ramus: aboral border of alveolus of M3 - Infradentale	-	86.0	-	-	-	70.0	85.1	-	-	-	(79)	-
3) Length from aboral border of condyle to aboral border of alveolus of M3	-	58.5	-	51.8	-	47.8	-	-	-	-	-	-
4) Length from aboral border of alveolus of M3 to oral border of C (C-M3)	-	67.8	-	64.1	-	-	-	-	-	-	-	-
5) Length of cheecktooth row (P3-M3)	-	67.2	-	57.2	-	56.5	66.1	-	-	-	-	-
6) Length of molar row (M1-M3)	-	41.0	-	37.7	-	39.1	36.7	-	-	-	-	-
7) Height of vertical ramus	-	59.9	-	62.5	-	-	-	-	63.0	-	55.2	49.5
8) Height of mandible behind M3	26.8	31.0	-	33.4	-	26.0	34.1	-	-	-	28.6	-
11) Length of the symphysis	-	48.0	28.3	-	33.5	29.8	34.0	40.1	34.8	29.0	-	(31)
12*) Ramus between P3 and P4	-	41.3	-	-	35.6	29.3	37.8	-	-	-	-	-
13*) Ramus between P4 and M1	-	39.6	26.1	-	33.4	27.4	36.3	36.5	30.5	24.6	-	29.3
14*) Ramus between M1 and M2	-	37.0	23.8	-	32.2	28.6	35.6	31.8	31.7	22.1	-	25.7
15*) Ramus between M2 and M3	-	-	-	-	28.8	25.8	-	-	-	-	-	-
